# Trace benzene capture by decoration of structural defects in metal–organic framework materials

**DOI:** 10.1038/s41563-024-02029-1

**Published:** 2024-10-29

**Authors:** Yu Han, Wenyuan Huang, Meng He, Bing An, Yinlin Chen, Xue Han, Lan An, Meredydd Kippax-Jones, Jiangnan Li, Yuhang Yang, Mark D. Frogley, Cheng Li, Danielle Crawshaw, Pascal Manuel, Svemir Rudić, Yongqiang Cheng, Ian Silverwood, Luke L. Daemen, Anibal J. Ramirez-Cuesta, Sarah J. Day, Stephen P. Thompson, Ben F. Spencer, Marek Nikiel, Daniel Lee, Martin Schröder, Sihai Yang

**Affiliations:** 1 Department of Chemistry, University of Manchester, Manchester, UK; 2 College of Chemistry and Molecular Engineering, Beijing National Laboratory for Molecular Sciences, Peking University, Beijing, China; 3 College of Chemistry, Beijing Normal University, Beijing, China; 4 Department of Chemical Engineering, University of Manchester, Manchester, UK; 5 Diamond Light Source, Harwell Science and Innovation Campus, Didcot, UK; 6 Chemical and Engineering Materials Division (CEMD), Neutron Sciences Directorate, Oak Ridge National Laboratory, Oak Ridge, TN USA; 7 ISIS Neutron and Muon Source, STFC Rutherford Appleton Laboratory, Chilton, UK; 8 Photon Science Institute, University of Manchester, Manchester, UK; 9 Department of Materials, University of Manchester, Manchester, UK; 10 National Graphene Institute, University of Manchester, Manchester, UK

**Keywords:** Metal-organic frameworks, Porous materials

## Abstract

Capture of trace benzene is an important and challenging task. Metal–organic framework materials are promising sorbents for a variety of gases, but their limited capacity towards benzene at low concentration remains unresolved. Here we report the adsorption of trace benzene by decorating a structural defect in MIL-125-defect with single-atom metal centres to afford MIL-125-X (X = Mn, Fe, Co, Ni, Cu, Zn; MIL-125, Ti_8_O_8_(OH)_4_(BDC)_6_ where H_2_BDC is 1,4-benzenedicarboxylic acid). At 298 K, MIL-125-Zn exhibits a benzene uptake of 7.63 mmol g^−1^ at 1.2 mbar and 5.33 mmol g^−1^ at 0.12 mbar, and breakthrough experiments confirm the removal of trace benzene (from 5 to <0.5 ppm) from air (up to 111,000 min g^−1^ of metal–organic framework), even after exposure to moisture. The binding of benzene to the defect and open Zn(II) sites at low pressure has been visualized by diffraction, scattering and spectroscopy. This work highlights the importance of fine-tuning pore chemistry for designing adsorbents for the removal of air pollutants.

## Abstract (web summary)

Benzene is a genotoxic carcinogen with no safe level of exposure. Here, by creating and decorating a structural defect in a metal–organic framework to form MIL-125-Zn, a benzene uptake of 7.63 mmol g^–1^ at 1.2 mbar is observed due to binding to Zn(II) sites.

## Main

Benzene is known as a genotoxic carcinogen with no safe level of exposure as recommended by the World Health Organization^1,2^. The ubiquitous presence of benzene in both indoor and outdoor settings underscores the need to remove trace benzene for the protection of health and the environment. Currently, oxidation^3^ and biological treatment^4^ are widely used for the removal of benzene, but the formation of hazardous by-products and the low efficiency of these processes restrict their application. Physisorption of benzene has attracted growing interest due to the cost-effective regeneration of the sorbent^5–7^. However, the lack of order and/or structural tunability in conventional porous materials, such as activated carbons, mesoporous silica and zeolites, render the precise control of pore structure and sorption properties extremely difficult^8,9^.

Metal–organic framework (MOF) materials show great promise as sorbents for toxic gases owing to their high porosity and tunable pore enviroment^10–13^. A number of MOFs show high benzene uptakes that outperform commercialized sorbents, such as MIL-101 (16.7 mmol g^−1^ at 303 K and 80 mbar; MIL, Material of Institute Lavoisier), MOF-177 (16.8 mmol g^−1^ at 298 K and relative pressure *P*/*P*_0_ = 1) and MOF-5 (12.8 mmol g^−1^ at 298 K and *P*/*P*_0_ = 1; *P*_0_ refers to the vapour pressure of benzene at saturation; *P* refers to measured vapour pressure of benzene)^14–17^. However, these uptakes at a relatively high partial pressure do not guarantee optimal performance under real-world conditions at low partial pressures. Several MOFs have been tested for benzene adsorption at low pressure (*P* < 1.2 mbar or *P*/*P*_0_ < 0.01), but they often exhibit low capacities^18–25^. Crystal engineering and pore manipulation have proven effective in increasing benzene uptake in MOFs at low pressure, with notable examples including BUT-55 (3.28 mmol g^−1^ at 7.3 Pa and 298 K) and Cu^II^/UiO-66 (3.92 mmol g^−1^ at 1.2 mbar and 298 K)^20,21^. However, the effective control of pore chemistry to improve benzene capture remains a substantial challenge. Furthermore, direct observation of the dynamics of binding interactions of captured benzene molecules within the confined voids of MOF hosts is largely lacking, impeding the design of improved sorbent materials.

Here we report the control of pore chemistry by the decoration of structural defects (missing metal centres) in MIL-125-defect by introduction of divalent single-atom sites to afford a series of bimetallic materials, MIL-125-X (X = Mn, Fe, Co, Ni, Cu, Zn). At 298 K, MIL-125-Zn exhibits a benzene uptake of 7.63 mmol g^−1^ at 1.2 mbar and 5.33 mmol g^−1^ at 0.12 mbar, a defining adsorption for porous solids. Dynamic breakthrough experiments demonstrate the potential of MIL-125-Zn to remove benzene at parts per million levels, even after exposure to moisture. In situ experiments based upon synchrotron powder X-ray diffraction, neutron powder diffraction (NPD), inelastic neutron scattering (INS), solid-state nuclear magnetic resonance (NMR), electron paramagnetic resonance and Fourier transformed infrared (FTIR) microspectroscopy, coupled with density functional theory (DFT) calculations, directly visualize the binding domains of captured benzene molecules and the dynamics of host–guest interactions at low pressure. The study provides a strategy to fine-tune the pore chemistry in MOFs to achieve optimal capture of trace toxic volatile organic compounds to mitigate air pollution.

## Materials and characterization

**Fig. 1:**
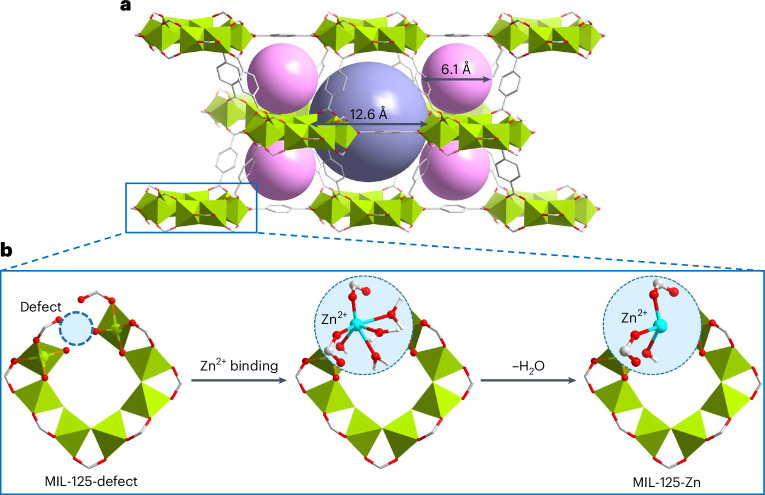
Schematic of decoration of defects by single-atom Zn(II) sites in MIL-125-defect. **a**, The crystal structure of MIL-125. The tetrahedral and octahedral cages are highlighted by pink and purple spheres, respectively, and hydrogen atoms are omitted for clarity. **b**, Schematic representation of the decoration of structural defects (missing metal centres) in the {Ti_7_O_7_(OH)_3_} cluster of MIL-125-defect by Zn(II) single-atom sites to afford a bimetallic {Ti_7_ZnO_7_(OH)_4_} cluster of MIL-125-Zn. The coordinated water molecules on Zn(II) were removed by activation under heating and vacuum to give unsaturated Zn(II) sites. Colour code: Ti, lime; Zn, turquoise; C, grey; O, red; H, white. Source data

MIL-125 possesses high stability and high porosity as well as tailorable pores. The robust structure of MIL-125 consists of ring-shaped {Ti_8_} titanium–oxo moieties connected by BDC^2−^ linkers, comprising octahedral and tetrahedral cages (dimensions of ~12.6 and 6.1 Å, respectively) interlinked by triangular apertures^26,27^ (Fig. 1a). The introduction of metal vacancies into the 12-connected {Ti_8_} ring using a deficient Ti source, accompanied with uncoordinated carboxylate oxygens, results in the formation of MIL-125-defect. Moreover, a secondary M(II) ion (M = Mn, Fe, Co, Ni, Cu, Zn) can be attached onto the metal vacancies to produce bimetallic MIL-125-X (Fig. 1b). Powder X-ray diffraction (Supplementary Figs. 1 and 2) of these MOFs confirms their phase purity and retention of structure after activation and long-term air exposure. Thermogravimetric analysis (Supplementary Fig. 3) confirms their thermal stability up to ∼620 K. Desolvated MOFs display a Brunauer–Emmett–Teller surface area of 1,462–1,866 m^2^ g^−1^ (Supplementary Fig. 4), comparable with that of MIL-125, reported previously^26^.

The averaged crystal structures of disordered MIL-125-defect and MIL-125-Zn were determined by Rietveld refinements of the NPD data. The refined Ti(IV) occupancy of 0.894(5) in MIL-125-defect (Ti_7.1_O_7.1_(OH)_3.6_BDC_5.3_(H_2_BDC)_0.7_) confirms the absence of, on average, nearly one Ti atom per {Ti_8_} ring, along with three disordered terminal carboxylate –O/–OH groups (O_defect_ atoms) surrounding the defect site. In MIL-125-Zn, the Zn(II) site occupies the Ti(IV) vacancy, resulting in bimetallic {Ti_7_Zn} rings with a Zn/Ti ratio of 1.04:6.95, confirmed by inductively coupled plasma–optical emission spectrometry and thermogravimetric analysis (Supplementary Section 1.1 and Supplementary Fig. 3). The anchored Zn(II) ion is stabilized through coordination to two carboxylate oxygen atoms (O_carb_) from the linker and one terminal hydroxyl group with Zn–O bonds of typically 2.0–2.1 Å, leaving a terminal carboxylate oxygen. The atomic dispersion of Zn(II) sites was also confirmed through X-ray absorption spectroscopy (Supplementary Fig. 5) and X-ray photoelectron spectroscopy (Supplementary Fig. 6). Transmission electron microscopy, scanning electron microscopy images and energy dispersive X-ray spectroscopy element mapping confirm an even distribution of the secondary metal ion within the MIL-125-X structure (Supplementary Fig. 7), and the refinement of high-resolution synchrotron X-ray diffraction data suggests that MIL-125-X (X = Mn, Fe, Co, Ni, Cu) incorporates a similar {Ti_7_X_1_} ring (Supplementary Table 3).

## Benzene adsorption and removal from air

**Fig. 2:**
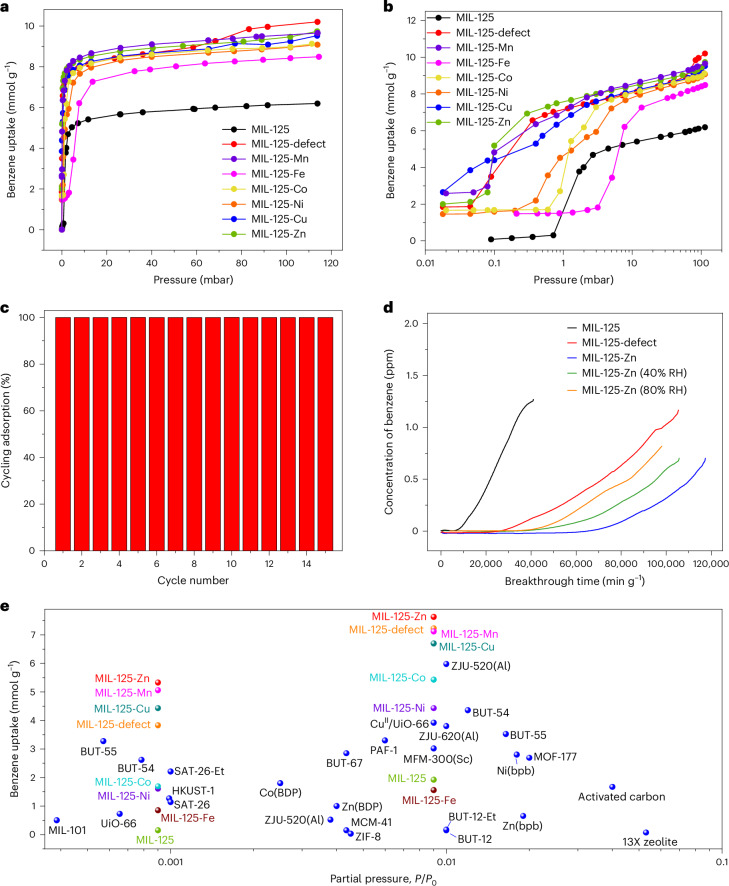
Benzene adsorption and breakthrough data. **a**, Adsorption isotherms for benzene in MIL-125, MIL-125-defect and MIL-125-X (X = Mn, Co, Ni, Cu, Zn) at 298 K (desorption data are shown in Supplementary Information for clarity). **b**, Logarithmic-scale plots of *P*/*P*_0_ to view the adsorption of benzene at low partial pressure. **c**, Comparison of benzene uptake capacities of MIL-125-Zn over 15 cycles at 298 K. At the 15th cycle, MIL-125-Zn retained over 99.99% of its initial benzene adsorption capacity, confirming the excellent stability of this material. **d**, Breakthrough curves of 0.0005% benzene (5 ppm) diluted in air through a fixed bed packed with MIL-125, MIL-125-defect, MIL-125-Zn and MIL-125-Zn pre-saturated with water under different humidities (40–80% relative humidity (RH)) at 298 K and 1 bar. **e**, Comparison of the benzene uptakes of MIL-125, MIL-125-defect and MIL-125-X with all leading sorbents reported to date. Source data

Single-component adsorption isotherms of benzene were collected for desolvated MIL-125, MIL-125-defect and MIL-125-X at 298–323 K (Fig. 2a,b and Supplementary Figs. 8 and 9). In contrast to the characteristic type-I isotherms observed for MIL-125 and MIL-125-X (X = Mn, Co, Ni, Cu, Zn), the adsorption isotherms of MIL-125-defect and MIL-125-Fe exhibit type-IV profiles likely due to the presence of structural voids and of slight structural flexibility, respectively. MIL-125-Zn shows a benzene uptake of 7.63 mmol g^−1^ at 298 K and 1.2 mbar, higher than that of MIL-125 (1.92 mmol g^−1^) and MIL-125-defect (7.23 mmol g^−1^) and reported values obtained under the same conditions for ZJU-520(Al) (5.98 mmol g^−1^)^25^, BUT-54 (4.31 mmol g^−1^)^20^, Carboxen 1000 (2.25 mmol g^−1^)^19^ and MCM-41 (0.45 mmol g^−1^)^19^. More importantly, compared with MIL-125 (0.15 mmol g^−1^) and MIL-125-defect (3.83 mmol g^−1^), MIL-125-Zn exhibits a notably higher adsorption capacity of 5.33 mmol g^−1^ at 298 K and 0.12 mbar (Supplementary Table 1), exceeding the leading sorbent, BUT-55 (3.39 mmol g^−1^)^20^. The performance of state-of-the-art porous materials for benzene adsorption at low pressure is summarized in Supplementary Table 2 and Fig. 2e. Notably, the introduction of defect sites and, more importantly, the presence of atomically dispersed Zn(II) sites greatly boost the adsorption of benzene at low pressure. The slightly higher adsorption of benzene by MIL-125-Zn compared with other MIL-125-X materials can be partially attributed to the higher charge density of Zn(II) ions that promotes stronger electrostatic interactions with benzene molecules. However, softer Cu(II) and Mn(II) ions can form enhanced Lewis acid–base interactions with benzene molecules^28^. Despite the high uptake at low pressure, adsorbed benzene can be readily removed upon desorption at *P*/*P*_0_ = 0, thus regenerating the free MOF and confirming the strong yet reversible adsorption affinity of benzene in these materials. MIL-125-Zn shows full retention of crystallinity and sorption capacity after 15 cycles of benzene adsorption–desorption (Fig. 2c). The isosteric heats of adsorption (*Q*_st_) analysis and toluene/cyclohexane adsorption measurements confirm the strong preferential affinity of MIL-125-Zn for aromatic compounds (Supplementary Section 15.1 and Supplementary Figs. 10–12).

To evaluate the efficiency of MIL-125-Zn for the capture of trace benzene from ambient air, we performed dynamic breakthrough experiments (Fig. 2d and Supplementary Fig. 13). Given the typically low concentrations of benzene present in air and industrial gas streams, an air mixture containing benzene (5 ppm) was passed through a column packed with ~10 mg of desolvated MOF at a flow rate of 200 ml min^−1^. Benzene started to break through the column of MIL-125-Zn after ~72,000 min g^−1^ of MOF (at 1% breakthrough time, that is, when the outlet concentration of benzene reaches 1% of the inlet concentration), corresponding to a dynamic adsorption capacity of ~3.21 mmol g^−1^, surpassing other reported values (2.14 mmol g^−1^ for BUT-55 at 10 ppm of benzene and 10 ml min^−1^ of gas flow rate)^20^. Thus, for an inlet feed of 5 ppm benzene, the benzene concentration can be purified to <0.05 ppm until breakthrough. Furthermore, the ability of MIL-125-Zn to capture benzene in the presence of moisture has been demonstrated by breakthrough experiments using MIL-125-Zn pre-saturated with water. Under different humidities (40–80% relative humidity), slightly reduced dynamic capacities are observed: 51,000 min g^−1^ and 2.27 mmol g^−1^ at 40% relative humidity, and 47,000 min g^−1^ and 2.09 mmol g^−1^ at 80% relative humidity, reflecting the competitive adsorption of water. MIL-125-Zn can be fully regenerated post-adsorption without loss of crystallinity or porosity (Supplementary Figs. 14–17). In addition, the breakthrough of benzene from MIL-125-defect shows a breakthrough time of ~40,200 min g^−1^ and benzene capacity of 1.79 mmol g^−1^, much higher than those of MIL-125 (10,800 min g^−1^ and 0.48 mmol g^−1^), indicating that the missing metal site plays a key role in the adsorption of trace benzene.

While no recommended safe level of exposure exists for airborne benzene concentrations, as determined by the World Health Organization, a number of agencies including the UK Health and Safety Executive, US Occupational Safety and Health Administration and European Union have established a threshold exposure limit for benzene at 0.5–1 ppm as an 8 h time-weighted average exposure^29^. In this context, a 10% breakthrough time (the time at which the outlet benzene concentration reaches 10% of the inlet concentration, that is, 0.5 ppm) was recorded. MIL-125-Zn demonstrates a 10% breakthrough time of 111,000 min g^−1^, which slightly decreases to 95,400 min g^−1^ on exposure to air, outperforming MIL-125-defect (76,200 min g^−1^) and MIL-125 (22,800 min g^−1^). These results indicate that MIL-125-Zn is capable of removing benzene from air to meet the most demanding criteria. The potential of MIL-125-Zn was further validated through gas separation experiments involving mixed matrix membranes composed of MIL-125-Zn and a polymer binder (Supplementary Section 1.3 and Supplementary Figs. 18 and 19).

## Determination of the binding domains for adsorbed benzene

**Fig. 3:**
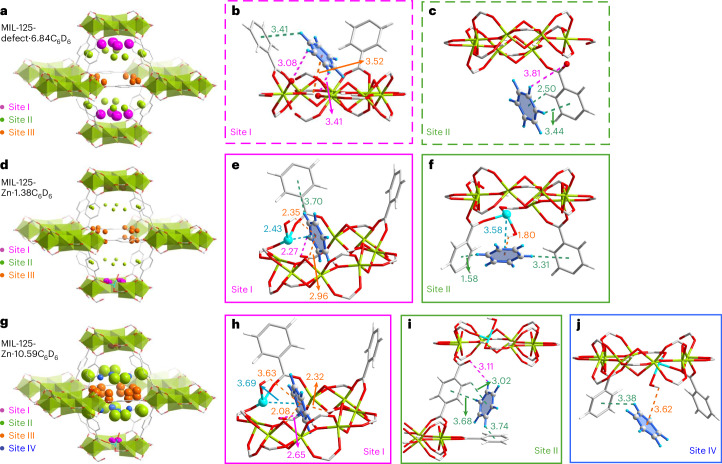
Structural models for the adsorption domains of benzene-d_6_ in MIL-125-defect and MIL-125-Zn determined from NPD data. **a**,**d**,**g**, Distribution of adsorbed benzene-d_6_ molecules within the octahedral cages in MIL-125-defect·6.84C_6_D_6_, MIL-125-Zn·1.38C_6_D_6_ and MIL-125-Zn·10.59C_6_D_6_. The radii of the coloured balls represent different binding sites and are proportional to their crystallographic occupancies. **b**,**c**, Views of host–guest interactions with benzene at sites I and II in MIL-125-defect·6.84C_6_D_6_. **e**,**f**, Views of host–guest interactions with benzene at sites I and II in MIL-125-Zn·1.38C_6_D_6_. **h**–**j**, Views of host–guest interactions with benzene at sites I–III in MIL-125-Zn·10.59C_6_D_6_. The Zn^II^···*π*_benzene_ interactions, C–D_benzene_···O_defect_ interactions, O–H_framework_···*π*_benzene_ interactions and C–H···*π* (or C–D···*π*) interactions are highlighted in blue, pink, orange and sea green, respectively. The estimated standard deviation for the distances are typically within the range 0.01–0.09 Å. Colour code: Ti, lime; Zn, turquoise; O, red; C, grey; D, sky blue; H, white.

The binding domains of benzene were studied by high-resolution synchrotron X-ray diffraction and NPD experiments, with deuterated substrates used in the latter. Refinement of the NPD data for benzene-d_6_-loaded MIL-125, MIL-125-defect and MIL-125-Zn confirms the retention of the long-range order of these structures upon the inclusion of benzene (Supplementary Figs. 20–23). For MIL-125 and MIL-125-defect, four distinct binding sites (I–IV) were identified (Fig. 3a–c and Supplementary Figs. 24–27). The occupancy refers to benzene per {M_7_} or {M_8_} ring throughout this study. In MIL-125-defect, sites I–III (occupancies of 2.00, 1.39 and 1.26, respectively) are located in the octahedral cages, while site IV (occupancy of 2.21) resides in tetrahedral cages (Fig. 3a and Supplementary Figs. 24a and 26a,b). Site I is anchored through interactions with the phenyl rings of ligands (C–D_benzene_···*π*_framework_ = 3.35(6)–3.53(7) Å) and bridging hydroxyl groups (O–H_framework_···*π*_benzene_ = 3.46(2)–3.52(3) Å) (Fig. 3b and Supplementary Fig. 26c). Sites II–IV are stabilized by multiple C–D_benzene_···*π*_framework_ interactions (distances of 2.74(3)–3.93(3) Å) and C–H_framework_···*π*_benzene_ interactions (distances of 2.50(1)–3.08(1) Å; Fig. 3c and Supplementary Figs. 24b,c and 26d–f). Moreover, benzene molecules confined in MIL-125-defect also interact with the terminal carboxylate oxygens (O_defect_) at the defect sites (C–D_benzene_···O_defect_ = 1.97(3)–3.81(5) Å). The strong adsorption of benzene in MIL-125-defect was further confirmed by DFT calculations, which indicate a higher adsorption energy for MIL-125-defect (−227 kJ mol^−1^) compared with MIL-125 (−178 kJ mol^−1^) at the strongest binding site.

For MIL-125-Zn, five binding sites are observed. Four of them (sites I–IV with occupancies of 0.42, 3.63, 2.67 and 1.50, respectively) are located in octahedral cages, and site V (occupancy of 2.37) is within the tetrahedral cages (Fig. 3g–j and Supplementary Figs. 28 and 29). Compared with MIL-125-defect, an additional binding site (site I) is found partially embedded in the {Ti_7_Zn} ring, forming Zn^II^···*π*_benzene_ interactions (distances of 3.69(9) Å), accompanied by O–H_framework_···*π*_benzene_ interactions (distances of 2.08(3)–3.63(9) Å) and C–D_benzene_···O_defect_ interactions (distances of 2.65(9) Å; Fig. 3h). Sites II–V are anchored by terminal carboxylate oxygen sites (C–D_benzene_···O_defect_ = 3.11(3)–3.60(4) Å), hydroxyl groups (O–H_framework_···*π*_benzene_ = 3.62(9) Å) and neighbouring phenyl rings (C–D_benzene_···*π*_framework_ = 2.41(4)–3.74(3) Å and C–H_framework_···*π*_benzene_ = 3.02(1)–3.69(1) Å; Fig. 3i,j and Supplementary Fig. 28b,c).

To understand further the adsorption behaviour at low concentrations, an additional NPD experiment was carried out for MIL-125-Zn with a low loading of benzene-d_6_. Structural analysis reveals three binding sites (sites I–III with occupancies of 0.35, 0.22 and 0.38, respectively) in octahedral cages and one binding site (site IV with an occupancy of 0.42) in tetrahedral cages (Fig. 3d–f and Supplementary Figs. 30 and 31). Interestingly, site I is closer to the Zn(II) site and is immobilized by stronger Zn^II^···*π*_benzene_ interactions (distance of 2.43(9) Å; Fig. 3e). Furthermore, site II is observed in close proximity to the {Ti_7_Zn} ring, with interactions with the Zn(II) site (Zn^II^···*π*_benzene_ = 3.58(7) Å; Fig. 3f). DFT calculations suggest that the Zn(II) centre is a thermodynamically strong binding site for benzene. This demonstrates the pivotal role of Zn(II) sites in binding benzene molecules, especially at low concentrations, consistent with the observed capture of benzene at low concentrations. By contrast, MIL-125-X (X = Mn, Fe, Co, Ni, Cu) presents only four binding sites for benzene with weaker X^II^···*π*_benzene_ interactions (Supplementary Section 15.2, Supplementary Table 2 and Supplementary Figs. 32–41).

## Analysis of the host–guest binding dynamics

**Fig. 4:**
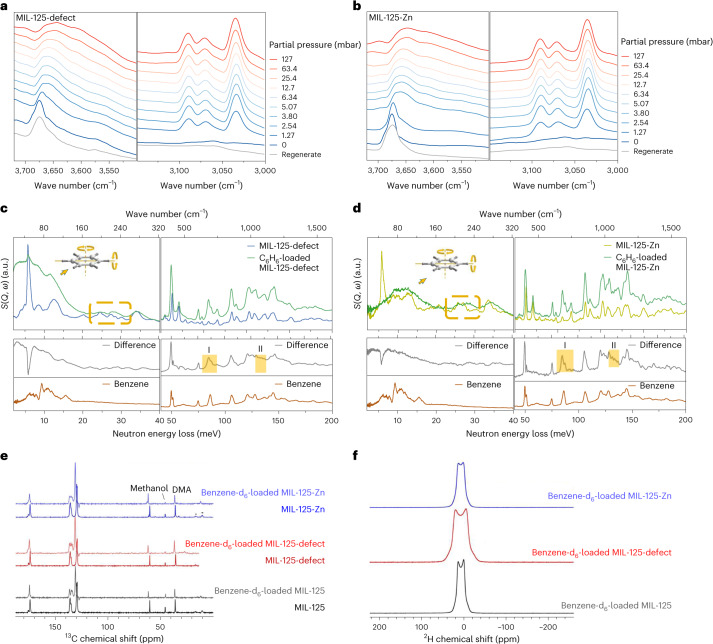
In situ FTIR, solid-state NMR and INS spectra for MIL-125-defect and MIL-125-Zn as a function of adsorption of benzene. **a**,**b**, In situ FTIR spectra of MIL-125-defect and MIL-125-Zn at partial pressures of benzene from 0 to 127 mbar (diluted in dry N_2_) and samples after regeneration at 353 K with a dry N_2_ flow. Bands at 3,720–3,500 cm^−1^ and 3,150–3,000 cm^−1^ correspond to *v*(O–H) and C–H stretching modes, respectively. **c**,**d**, INS spectra of bare and benzene-loaded MIL-125-defect and MIL-125-Zn. Difference spectra were obtained by subtraction of the INS spectra of the bare MOF from that of the benzene-loaded MOF. The INS spectra of solid benzene are also shown for comparison. The rotation of the benzene ring is present and marked in the spectra. *S*, dynamic structure factor; *Q*, momentum transfer; *ω*, energy transfer. **e**, The {^1^H}–^13^C cross-polarization MAS solid-state NMR spectra of MIL-125 (black, bottom), MIL-125-defect (red, middle) and MIL-125-Zn (blue, top), and benzene-d_6_-loaded counterparts (above corresponding spectra). Asterisks denote spinning side bands. DMA, dimethylamine. **f**, The ^2^H solid–echo solid-state NMR spectra of benzene-d_6_-loaded MIL-125 (black, bottom), MIL-125-defect (red, middle) and MIL-125-Zn (blue, top). All spectra were recorded at 9.4 T using a MAS frequency of 12 kHz. Source data

In situ FTIR microspectroscopy of MIL-125-defect and MIL-125-Zn as a function of benzene loading (Fig. 4a,b) shows depletion of the O–H stretching bands at 3,687 and 3,674 cm^−1^ together with a notable redshift (*Δ* = 16–31 cm^−1^), consistent with binding of benzene molecules to the –OH moiety from both bridging hydroxyl groups and defect sites (for MIL-125-defect). MIL-125-Zn exhibits smaller *v*(O–H) vibrational changes than MIL-125-defect at 1.27 mbar. This is consistent with the role of Zn(II) sites as dedicated binding sites for benzene under low pressure conditions. In addition, the perturbation of the framework C–H band (3,060 cm^−1^) and continuous growth of benzene C–H band (3,090 cm^−1^) reflect the presence of interactions between benzene and phenyl rings of the framework. The substantial change observed at 1.27 mbar aligns with the sharp adsorption of benzene at low pressure. By contrast, upon benzene adsorption, MIL-125 shows only a perturbation in one O–H stretching band (Supplementary Fig. 42), consistent with the presence of a single type of hydroxyl group within MIL-125.

INS experiments were conducted for MIL-125-defect and MIL-125-Zn to gain further insights into the dynamics of framework vibrations and the translational and rotational motion of adsorbed benzene. Simulated INS spectra were obtained via DFT calculations based on the above structural models and show excellent agreement with experimental spectra (Supplementary Fig. 43). This allows the assignment of observed INS intensities to the corresponding vibrational modes. Solid benzene shows bands at 50.2–64.0 cm^−1^, assigned to rotation along the *C*_6_ axis; at 75.7 cm^−1^ for rotation at one of the *C*_2_ axes; and at 84.6–96.8 cm^−1^ for another *C*_2_ axis. Although the adsorbed benzene exhibits relatively broad features in this frequency range, a more detailed analysis can be performed by examining the underlying vibrational modes based on DFT calculations. The analysis reveals that the wave number difference between *C*_6_ and *C*_2_ rotations increases to *Δ* = ~40 and ~70 cm^−1^ for benzene adsorbed at site I, indicating a marked energy discrepancy between *C*_6_ and *C*_2_ rotations. This substantial energy difference, coupled with the presence of new peaks in the low-frequency domain (10–40 cm^−1^) and the splitting of vibrational peaks in region I demonstrate robust and directional host–guest interactions, consistent with the NPD results. Meanwhile, the decrease near 46.4 cm^−1^ and the redshift in the C–H bending region (region II) in both MOFs suggest restricted ligand phenyl ring rotation along its *C*_2_ axis due to guest molecules. Peaks observed at 150–250 cm^−1^ (framed in Fig. 4c,d) can be attributed to the hydroxyl groups on the Ti cluster and attached Zn(II) centre in MIL-125-defect and MIL-125-Zn, respectively. The broadening of these peaks is due to the emergence of new vibration modes induced by additional benzene molecules at site I.

To probe the structural changes of the host framework upon benzene adsorption, magic angle spinning (MAS) NMR spectra were recorded for MIL-125, MIL-125-defect and MIL-125-Zn (Supplementary Section 15.3, Fig. 4e and Supplementary Fig. 44)^30^. After doping the MOFs with benzene-d_6_, all the ^13^C resonances shift, except for the methanol peak at *δ*{^13^C} = 45 ppm. This indicates that the framework undergoes a slight structural change to accommodate the benzene molecules. Interestingly, this change is the same for all the studied MOFs. The most notable change is in the overlapping peaks at *δ*{^13^C} ≈ 135 ppm, which split into three discrete peaks upon the loading of benzene. This suggests that the phenyl ring in the organic ligand has twisted away from its original quasi-planar position. The shift of the methanol peak at *δ*{^13^C} = 60 ppm demonstrates that it is part of the framework, likely as {Ti–MeOH} moieties. The absence of a large shift of the carboxylate ^13^C resonances upon benzene adsorption highlights that the MIL-125 framework remains similar, without major breathing effects^31^, although twisting of the ligands within the framework seems likely.

To investigate further the host–guest interactions of benzene molecules in MIL-125, MIL-125-defect and MIL-125-Zn, static ^2^H NMR spectra were recorded at ambient temperature for a low loading of benzene-d_6_, at less than one molecule per pore to minimize guest–guest interactions (Fig. 4f). The ^2^H NMR spectra all display similar characteristic motionally averaged quadrupolar line shapes. This indicates that the motion of benzene molecules is anisotropic for all MIL-125 materials at ambient temperature and, as such, provides further evidence of the strong interaction of the framework with benzene. This is contrary to what is observed for benzene in a mesoporous silica (SBA-15; ref. ^32^) and in the MOF UiO-66(Zr) (ref. ^33^), where isotropic motion is observed at ambient temperature. Therefore, the MIL-125 framework interacts with benzene, with the introduction of defects in MIL-125-defect and the formation of MIL-125-Zn providing higher capacity, with the framework retaining a robust structure that allows local movements (rotation of ligands) to maximize host–guest interactions. This underpins the observed high uptake of benzene at low partial pressures.

**Fig. 5:**
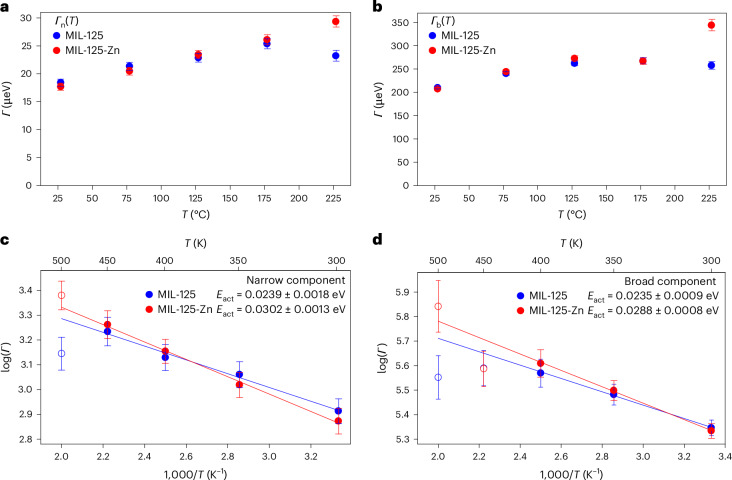
QENS analysis for adsorbed benzene molecules in MIL-125 and MIL-125-Zn. **a**,**b**, Fitted values of the width of the Lorentzian curve (*Γ*) for the narrow (*Γ*_n_) and broad (*Γ*_b_) components. **c**,**d**, Arrhenius plots for the narrow and broad components. In both cases (that is, narrow and broad components), the activation energies (*E*_act_) for the processes are very similar. *Γ*_n_(*Q*) and *Γ*_b_(*Q*) are the widths of the Lorentzian curves; these are set to a constant value independent of *Q*, and the subscripts n and b refer to a narrow and broad component, respectively. Data points represent mean values with error bars indicating the standard deviation (s.d.) derived from the fitting method described in Supplementary Section 14 against *Q* at five temperatures (*T*) of 27, 77, 127, 177 and 227 °C. The lines are the Arrhenius fitting result to determine the activation energies. The empty circles are excluded from the Arrhenius fitting process. Source data

Quasi-elastic neutron scattering (QENS) has been employed to analyse the impact of Zn(II) sites on host–guest binding in MIL-125 and MIL-125-Zn (Supplementary Section 14). QENS is a powerful technique to study the diffusion of guest molecules in porous materials, but has been rarely applied to study benzene adsorption in MOFs^33–35^. Fitting of the QENS data collected at 300–500 K, using an equation and model that includes a narrow and broad component (Fig. 5a,b and Supplementary Figs. 45 and 46), suggests that MIL-125-Zn has a higher averaged activation energy (0.0295 ± 0.0013 eV) compared with MIL-125 (0.0237 ± 0.0018 eV; Fig. 5c,d). Thus, the QENS analysis confirms the important role of Zn(II) sites in promoting the binding of benzene molecules, fully consistent with the superior adsorption at low pressure in MIL-125-Zn.

## Outlook

The enhancement of trace benzene capture through the decoration of structural defects in MIL-125-defect with single-atom sites demonstrates a promising strategy for tackling air pollution. The highly efficient removal of trace benzene by MIL-125-Zn under ambient conditions underscores its potential for practical indoor air quality management. The principles of defect engineering and single-atom site decoration could be extended beyond benzene capture, offering adaptability for targeting other volatile organic compounds and gaseous pollutants. Additionally, the multifaceted characterization in this study provides a valuable framework for probing host–guest interactions in porous materials, potentially accelerating the discovery and optimization of sorbents for various environmental and industrial applications. This work highlights the critical importance of fine-tuning pore chemistry in the design of viable and efficient sorbents.

## Methods

### Gas adsorption isotherms and breakthrough experiments

Measurements of the benzene adsorption isotherm (relative pressure of 0–0.9) were performed using an Intelligent Gravimetric Analyzer (Hiden Isochema). Methanol-exchanged samples were loaded into the system and degassed at 443 K and high dynamic vacuum (10^−10^ bar) for 12 h to give fully desolvated samples. Breakthrough experiments were carried out in a 3-mm-diameter fixed bed tube of 50 mm length packed with ~10 mg of the samples. The sample was heated at 150 °C under a flow of He overnight for complete activation. The fixed bed was then cooled to room temperature and the breakthrough experiment was performed with a calibration gas (5 ppm benzene balanced with air) at atmospheric pressure and room temperature. The flow rate of the entering gas was maintained at 200 ml min^−1^, and the concentration of benzene at the outlet determined by mass spectrometry. For the breakthrough experiment for moisture-exposed MIL-125-Zn, no activation was conducted before the measurement^13,36^. The breakthrough was defined as 1% of initial concentration (that is, 0.05 ppm) detected in the eluted stream. This is higher than the detection limit of the mass spectrometer (<0.01 ppm) and is recorded as 1% breakthrough time. Mixed matrix membrane preparation and gas separation experiments are detailed in the Supplementary Information.

### Structure determination from NPD and high-resolution synchrotron X-ray diffraction experiments

NPD experiments were carried out on WISH at the ISIS Neutron and Muon Source at Rutherford Appleton Laboratory, UK (bare MIL-125-defect, low-C_6_D_6_-loaded MIL-125-Zn) and on POWGEN at the Oak Ridge National Laboratory, US (C_6_D_6_-loaded MIL-125, bare MIL-125-Zn and high-C_6_D_6_-loaded MIL-125-Zn). The methanol-exchanged MOF samples were activated at 423 K and 10^−10^ mbar for 12 h, noted as bare samples, before exposure to benzene-d_6_ vapour at room temperature for 12 h to give benzene-d_6_-loaded samples. The powder was then transferred into cylindrical vanadium sample cells in a glove box. All NPD patterns were collected at 7 K. Rietveld refinements were carried out on NPD patterns of the bare MOF sample and samples dosed with C_6_D_6_ using the TOPAS v.5.0 package. Structure-independent Pawley refinements were first carried out to extract the peak shape and background parameters, which were used and initially fixed in the following Rietveld refinement. The structural model of the initial MIL-125 framework was obtained from the reported crystal structure. For the bare bimetallic MIL-125-Zn, the positions of Zn and the terminal OH were initially determined based on the Fourier difference map followed by full-profile Rietveld refinement including the positions of metal and linkers, the occupancy of atom sites, thermal parameters (*U*_iso_) and lattice parameters. Residual solvent methanol was described by the rigid-body model, where the position and orientation were refined based on the Fourier map. Chemically reasonable restraints on bond distance and angle were applied on the organic linker to keep the molecule geometry. In addition, the phenyl ring of the BDC linker was allowed to rotate along its *C*_2_ axis to reflect the influence of host–guest interactions on the framework structure. All other non-structural parameters, including that of the background, peak shape and peak broadening correction function, were released and refined at the final stage. For the structure of C_6_D_6_-loaded samples, rigid bodies were used to define the molecular geometry of the C_6_D_6_ molecule with refinable C–C and C–H bond distance and fixed bond angles. Structural models of the framework were taken from the previous refinement. The initial centres of mass and orientations of C_6_D_6_ molecules in the pore were chosen by simulated annealing. At the final stage, all structure parameters including atomic coordinates, thermal parameters and occupancies and the non-structural parameters were refined with restraints on the linker geometry. All structure solutions were accepted with good agreement factors of the refinement.

High-resolution synchrotron powder X-ray diffraction patterns for C_6_H_6_-loaded MIL-125-X (X = Mn, Fe, Co, Ni, Cu) were collected at beamline i11 of the Diamond Light Source using a position sensitive detector and monochromated radiation (wavelength *λ* = 0.824388 Å). The methanol-exchanged MOF samples were loaded into capillary tubes of 0.7 mm diameter, which were treated at 443 K and 10^−10^ bar for 12 h followed by exposure to saturated benzene atmosphere at room temperature for 12 h to give benzene-loaded samples. Diffraction patterns were then collected at room temperature. Rietveld structure refinement on synchrotron powder X-ray diffraction patterns were carried out using the TOPAS package. Pawley refinement was conducted first to obtain the refined non-structural parameters, including background, peak shape and lattice parameters. Structural models from the NPD results were used and fixed at the beginning of refinement. A rigid-body model was used to define the geometry of benzene molecules. The initial centre of mass values and orientations of the benzene molecules were guessed using the simulated annealing method. All parameters were released to be refined in the final stage, and structure solutions were accepted with good agreement factors.

All crystal structures determined reflect a set of averaged structures due to the presence of multi-fold disorders from both the framework and the distribution of guest molecules. The physical properties reported in this paper are merely the average values of the assembly of microcrystals.

### In situ FTIR spectroscopy

In situ FTIR microspectroscopy was carried out at the Multimode InfraRed Imaging and Microspectroscopy (MIRIAM) beamline at the Diamond Light Source. The in situ FTIR spectroscopy was performed according to ref. ^21^. Samples were desolvated under dry N_2_ flow at 423 K for 2 h, cooled to 298 K and dosed with benzene vapour at different partial pressures (0–127 mbar).

### INS

INS spectra were recorded on the VISION spectrometer at the Spallation Neutron Source, Oak Ridge National Laboratory and on the TOSCA Facility at Rutherford Appleton Laboratory. The methanol-exchanged MOF samples were activated at 423 K and 10^−10^ mbar for 12 h, noted as bare samples, before exposure to benzene vapour at room temperature for 12 h to give benzene-loaded samples. The powder was then transferred into cylindrical vanadium sample cells in a glove box. The temperature was kept below 10 K during the scattering measurements to minimize the thermal motion of the framework host and the adsorbed benzene molecules. Background spectra (sample can plus bare MOF) were subtracted to obtain the difference spectra.

### QENS

QENS experiments were carried out on four samples, including bare and benzene-loaded MIL-125 and MIL-125-Zn, on the neutron spectrometer (IRIS) at the ISIS Pulsed Neutron and Muon Source, Chilton, UK. IRIS is a time-of-flight inverted-geometry neutron spectrometer with a single-crystal array of pyrolytic graphite as an energy analyser that makes use of Bragg reflections close to backscattering geometry (Bragg angle *θ*_B_ = 175°) to analyse the energy of scattered neutrons. The detector array that counts those scattered neutrons covers a scattering angle (2*θ*) range of 27–158°, yielding a wave vector (*Q*) range of 0.4–1.8 Å^−1^. In each measurement, IRIS performed in the PG(002) configuration, which offers an energy resolution of 17 μeV and an energy window (*ħω*, where *ħ* is the reduced Planck constant) of −0.5–0.5 meV. The sample (~1.2 g) was packed into an annular aluminium container with an appropriate thickness (0.5 mm) to minimize multi-scattering effects. Data collection was made at every 50 K in the temperature range of 300–500 K. Fitting and analysis of the QENS data are detailed in the Supplementary Information.

### DFT calculations

The DFT calculations were performed according to ref. ^37^. The DFT calculations in this work were conducted via a simplified structural model to assign the experimentally observed INS peaks to their corresponding vibrational modes so that the measured changes upon benzene dosing can be fully interpreted with less ambiguity and in more detail.

### Solid-state NMR

Solid-state NMR spectroscopy was carried out on a Bruker 9.4 T (400 MHz ^1^H Larmor frequency) Avance III spectrometer equipped with a 4 mm HFX MAS probe. The {^1^H}–^13^C cross-polarization MAS NMR experiments were acquired at ambient temperature using a MAS frequency of 12 kHz. ^1^H and ^13^C pulses of 100 and 50 kHz were used, respectively, and ^13^C spin-locking at 50 kHz was applied for 2 ms, with corresponding ramped (70–100%) ^1^H spin-locking at ∼73 kHz for cross-polarization experiments with 100 kHz of SPINAL-64 heteronuclear ^1^H decoupling^38^ used throughout signal acquisition. The ^1^H MAS NMR spectra were recorded after an echo of two rotor periods total duration. The ^2^H NMR experiments were acquired under static sample conditions at ambient temperature using a solid–echo pulse sequence (90*x*–*τ*–90*y*–*τ*–acquisition) with π/2 pulses of 4.5 μs duration and half-echo delays (*τ*) of 10 μs. Samples were packed into 4-mm-outer-diameter zirconia rotors and activated (423 K and 10^−2^ mbar for 12 h) and were subsequently exposed to benzene-d_6_ at room temperature before being sealed with a Kel-F rotor cap. The benzene-d_6_-loaded samples were exposed to vacuum (10^−2^ mbar) at 298 K for 5 min to give low-loading samples.

## Online content

Any methods, additional references, Nature Portfolio reporting summaries, source data, extended data, supplementary information, acknowledgements, peer review information; details of author contributions and competing interests; and statements of data and code availability are available at 10.1038/s41563-024-02029-1.

## Supplementary information

Supplementary Information Experimental section, Supplementary Figs. 1–46, Tables 1–3 and additional Discussion.

Supplementary Data 1 Crystallographic data (11 CIFs) and checkCIF reports.

Supplementary Data 2 Optimized computational models for DFT calculations (2 CIFs).

## Source data

Source Data Figs. 2a–d, 4a–d and 5 Source data.

## Data Availability

All relevant data are available from the corresponding authors and/or are included in Supplementary Information. The crystallographic data of the MOFs reported in this work have been deposited in the Cambridge Crystallographic Data Centre (CCDC) under accession numbers CCDC 2301737 (MIL-125-defect), 2301736 (MIL-125-Zn), 2301732 (C_6_D_6_-loaded MIL-125), 2301735 (C_6_D_6_-loaded MIL-125-defect), 2301734 (C_6_D_6_-loaded MIL-125-Zn-high), 2301733 (C_6_D_6_-loaded MIL-125-Zn-low), 2301739 (C_6_H_6_-loaded MIL-125-Mn), 2301738 (C_6_H_6_-loaded MIL-125-Fe), 2301740 (C_6_H_6_-loaded MIL-125-Co), 2301741 (C_6_H_6_-loaded MIL-125-Ni) and 2301742 (C_6_H_6_-loaded MIL-125-Cu). These data can be obtained free of charge from the CCDC via www.ccdc.cam.ac.uk/data_request/cif. All other data supporting the findings of this study are available within the article and its Supplementary Information. Source data are provided with this paper.
