# Supplementary material for: Trace benzene capture by decoration of structural defects in metal–organic framework materials
Source: Nat Mater. 2024 Oct 29;23(11):1531–8. doi: 10.1038/s41563-024-02029-1 (PMC11525167; doi:10.1038/s41563-024-02029-1)
Supplement: Supplementary file 1 — Experimental section, Supplementary Figs. 1–46, Tables 1–3 and additional Discussion. [file 41563_2024_2029_MOESM1_ESM.pdf]

nature materials

Supplementary information https://doi.org/10.1038/s41563-024-02029-1

Trace benzene capture by decoration of structural defects in metal–organic framework materials

In the format provided by the authors and unedited

Index for Supplementary Information

1. Experimental section

1.1. MOF synthesis

1.2 Characterisation of materials

1.3. Preparation of mixed-matrix membranes and gas separation experiments

2. PXRD, TGA and N_2 isotherms

3. X-ray absorption spectroscopy (XAS) and XPS

4. Scanning electron microscopy and energy-dispersive X-ray spectroscopic elemental mapping

5. Gas sorption isotherms

6. Breakthrough curves

7. Membrane-based separation

8. Rietveld refinement of NPD and SPXRD patterns

9. Crystallographic data for crystal structures

10. Views of crystal structures

11. *In situ* FTIR spectra

12. DFT-simulated INS spectra

13. ssNMR spectra

14. QENS analysis

15. Additional discussion

15.1. Q_{st} and toluene/cyclohexane adsorption

15.2 Binding domains for adsorbed benzene

15.3. ssNMR

16. References

1. Experimental Section

1.1. MOF synthesis

All the reagents were used as received from commercial suppliers without further purification. MIL-125 was synthesised according to the reported method[1] but with modifications. MIL-125-defect and MIL-125-X (X = Mn, Fe, Co, Ni, Cu, Zn) were synthesised according to the reported methodology[2] but with modifications as described below.

Synthesis of MIL-125. Terephthalic acid (3.0 g, 18.1 mmol) and N,N-dimethylacetamide (DMF) (54 mL, 0.58 mmol) were mixed under stirring for 10 mins, and methanol (6 mL, 0.15 mmol) and tetrabutyl titanate (1.56 mL, 4.58 mmol) were added. The mixture was heated at 130 °C for 20 h under reflux. After cooling down to room temperature, the resulting product was collected by centrifugation and washed with DMF and methanol several times.

Synthesis of MIL-125-defect. The same procedure was used as for the synthesis of MIL-125 except from that 1.2 mL (3.52 mmol) of tetrabutyl titanate was used. The use of an insufficient amount of Ti(IV) with respect to the metal/linker ratio allows a fraction of the carboxylates to be not fully coordinated, resulting in defect sites. These coordinatively free oxygen centers can be utilised to anchor a secondary X(II) (X = Mn, Fe, Co, Ni, Cu, Zn) ion at the Ti vacancies to obtain MIL-125-X. The molar ratio of metal/ligand was determined as 1.06 using inductively coupled plasma-optical emission spectrometry (ICP-OES).

Synthesis of MIL-125-X (X = Mn, Fe, Co, Ni, Cu, Zn). MIL-125-defect (0.5 g, 0.33 mmol) was dispersed in methanol (35 mL, 0.86 mmol), and 0.33 mmol of metal salt [manganese(II) chloride, iron(II) chloride, cobalt chloride, nickel dichloride, copper(II) chloride, zinc chloride] was added and stirred for 3 h. The resulting product was collected by centrifugation and washed with methanol. The loading of X(II) in resultant materials was determined by ICP-OES and the X(II):Ti molar ratio lies in the range of 0.9-1.1:7). We attempted to incorporate double and triple quantities of $ZnCl_2$ (i.e., 0.66 and 0.99 mmol, respectively) during the preparation of MIL-125-Zn. However, the Ti/Zn ratio in the resulting MOFs remained at approximately 1:7 in all cases (as determined by ICP). This observation is attributed to the missing-metal defects in MIL-125-defect being the controlling factor to the incorporation of $ZnCl_2$.

1.2 Characterisation of materials

Powder X-ray diffraction (PXRD) data was collected in flat plate mode over the 2θ range 5−50° on an X'pert multipurpose diffractometer using Cu-Kα radiation (λ = 1.54056 Å, 40 kV/30 mA). Thermo-gravimetric analysis (TGA) was carried out on a SDTQ600 TA instrument under air flow with a heating rate of 5 °C min^{-1}. ICP-OES analysis was performed on a Thermo Scientific iCAP 6300 Duo.

Characterisation of porosity. The as-synthesised MOF samples were soaked in methanol at room temperature for 3 days, with the solution exchanged with fresh acetone every day. The resulting solvent-exchanged samples were dried in air followed by degassing at 443 K and 10^{-10} bar for 12 h to afford the fully desolvated samples,

which were loaded in a Tristar II PLUS (Micrometrics company) for porosity analysis. The BET surface areas were calculated using the N_2 isotherms measured at 77 K for MIL-125, MIL-125-defect and MIL-125-X (X = Mn, Fe, Co, Ni, Cu, Zn).

X-ray photoelectron spectroscopy (XPS). X-ray photoelectron spectroscopy (XPS) was carried out using a Kratos Axis Ultra Hybrid spectrometer using Al Kα X-rays (1486.6 eV, 150 W at 15 kV bias); a charge neutraliser was used to replenish the electron supply at the surface. Survey spectra were measured using a pass energy of 80 eV and high-resolution core levels with a pass energy of 20 eV. Charge (binding energy scale) calibration was performed using C 1s at 284.8 eV, and data were analysed using CASAXPS (www.casaxps.com). Voigt peak shape was used for all 2p.

Scanning electron microscopy (SEM) and energy dispersive spectroscopy (EDS). SEM and EDS images were captured using a FEI Quanta 650 system equipped with a Bruker Quantax energy dispersive spectrometer. Sample powder was sprayed onto adhesive carbon tapes attached to aluminium sample stages. All samples were further coated with carbon to avoid surface charging. The SEM images and EDS mapping were captured with a working distance of 10 mm and an accelerating voltage of 15 kV with a spot size of 5 μm.

Transmission electron microscopy (TEM). Samples for scanning transmission electron microscopy (STEM) were prepared by drop-casting the nanoparticle dispersion in methanol directly onto a gold grid coated with an amorphous holey carbon film. STEM images were collected using a JEOL ARM300F equipped with a Cs probe aberration corrector, XMAX 100 EDX detector (Oxford Instruments), high-angle annular dark-field and segmented detectors. The system was operated at 300kV.

X-Ray absorption spectroscopy (XAS). Zn-K edge X-ray absorption for MIL-125-Zn was collected at Diamond Light Source on beamline B18 in transmission mode to illustrate both the oxidation state and local structure. The X-ray beam was monochromated using a Si [3 1 1] monochromater. A metallic Zn foil standard was used as a reference for energy calibration and was measured simultaneously with experimental samples. Data were processed using the Athena and Artemis programs of the IFEFFIT package based on FEFF 6. Prior to merging, spectra from transmission mode were calibrated against the reference spectra and aligned to the first peak in the smoothed first derivative of the absorption spectrum, with background removed. Spectra were processed to obtain a normalized unit edge step. Refinement was performed by optimizing an amplitude factor S_0^2 and energy shift ΔE_0, which are common to all paths, in addition to parameters for bond length (ΔR) and Debye-Waller factor (σ^2). All XAS plots are represented without phase correction. Therefore, the radial distances observed in the EXAFS plots are ~0.4–0.5 Å below their actual value.

1.3. Preparation of mixed-matrix membrane and gas separation experiments

The 15 wt.% MIL-125-Zn/6FDA-Durene membrane was prepared by a solvent evaporation method. A solution

containing 0.35g of combined MOF particles in 6FDA-Durene polymer was prepared by dispersing desolvated MIL-125-Zn powder (52.5 mg) in THF (5 mL) followed by sonication for 2 hours. Dried 6FDA-Durene polymer powder (297.5 mg) was added to the dispersion in portions and the mixture vigorous stirred for 4 h. The solution was sonicated for 15 mins sonication and stirred for a further 15 min. This was further repeated twice. The cast solution was then poured into a clean petri dish laid on a flat surface and the THF slowly evaporated. The membrane was then peeled off and activated under dynamic vacuum at 393K overnight to remove any residual THF.

The potential of MIL-125-Zn as the porous filler in membrane-based benzene/air separation was evaluated. Membrane separation experiments were carried out using a customised rig in full-permeation mode (Figure S34). The activated membrane was cut and placed on a porous stainless-steel support, and clamped between O-rings in a flange. The flow rate of the benzene/air mixture (containing 5 ppm in benzene) was set to 40 standard cubic centimetre per minute (SCCM) controlled by a Alicat digital mass flow controller (MFC). The permeate was swept by 20 SCCM helium, and the composition was monitored using an in-line connected Hiden Analytics mass spectrometer. The experiment was conducted at room temperature for up to 24 h and no signal of continuous benzene permeation could be observed.

2. PXRD, TGA and N_2 isotherms

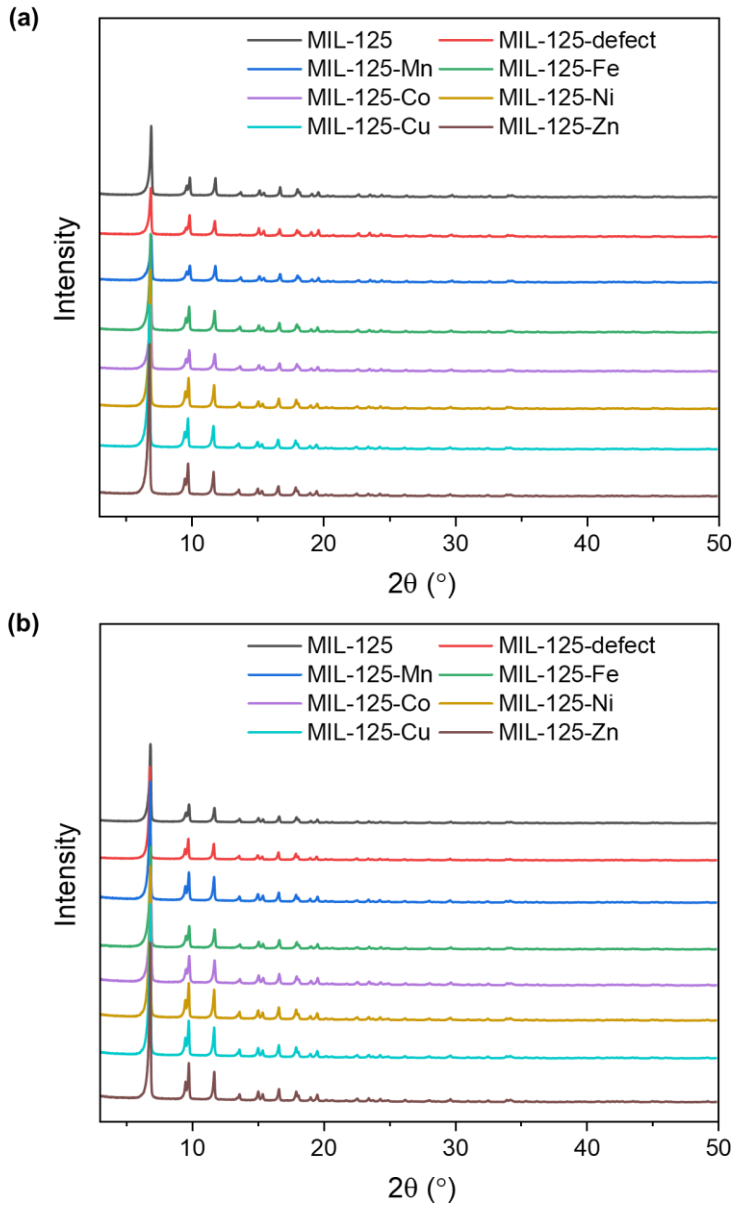

Figure S1. PXRD patterns (λ = 1.54056 Å) for MIL-125, MIL-125-defect and MIL-125-X (X = Mn, Fe, Co, Ni, Cu, Zn) (a) as-synthesised and (b) post activation.

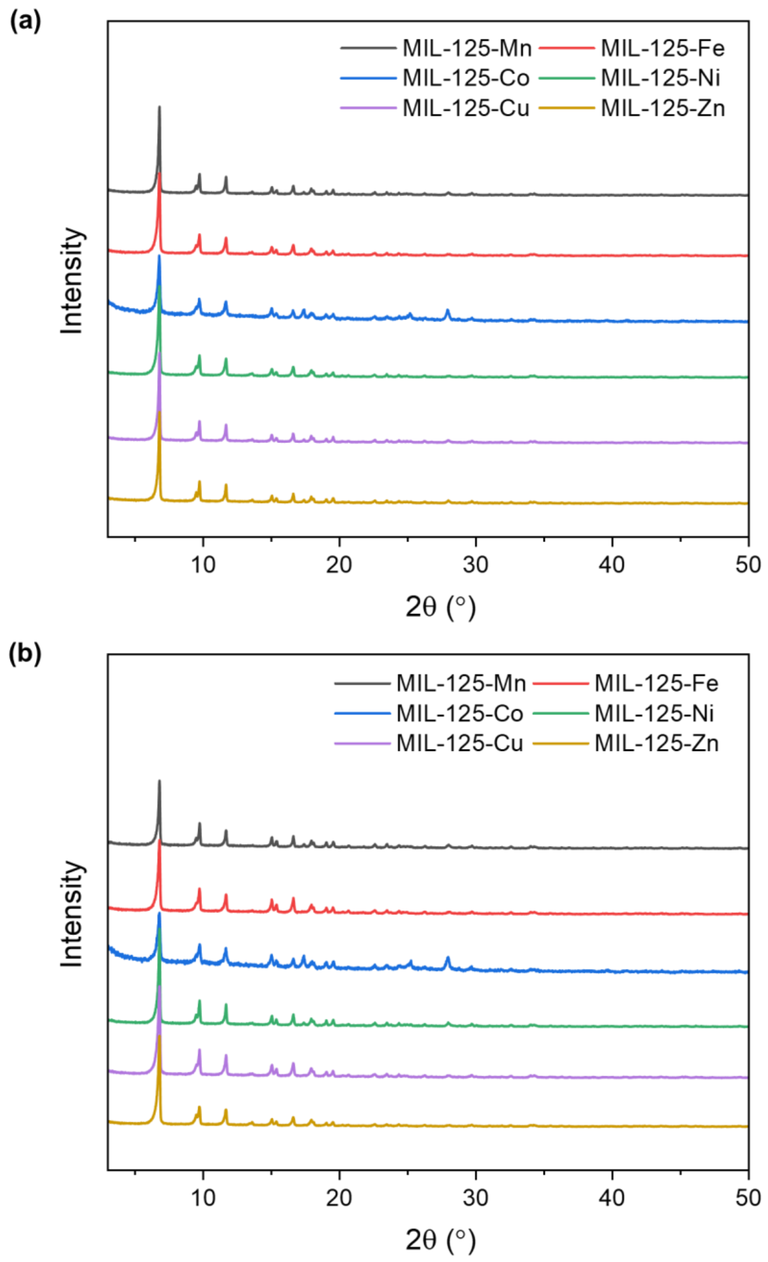

Figure S2. PXRD patterns (λ = 1.54056 Å) for MIL-125-X (X = Mn, Fe, Co, Ni, Cu, Zn) (a) after being soaked in liquid benzene for 60 days, and (b) after being exposed in air for 1 year. These results confirm the excellent stability of MIL-125-X following the introduction of metal sites.

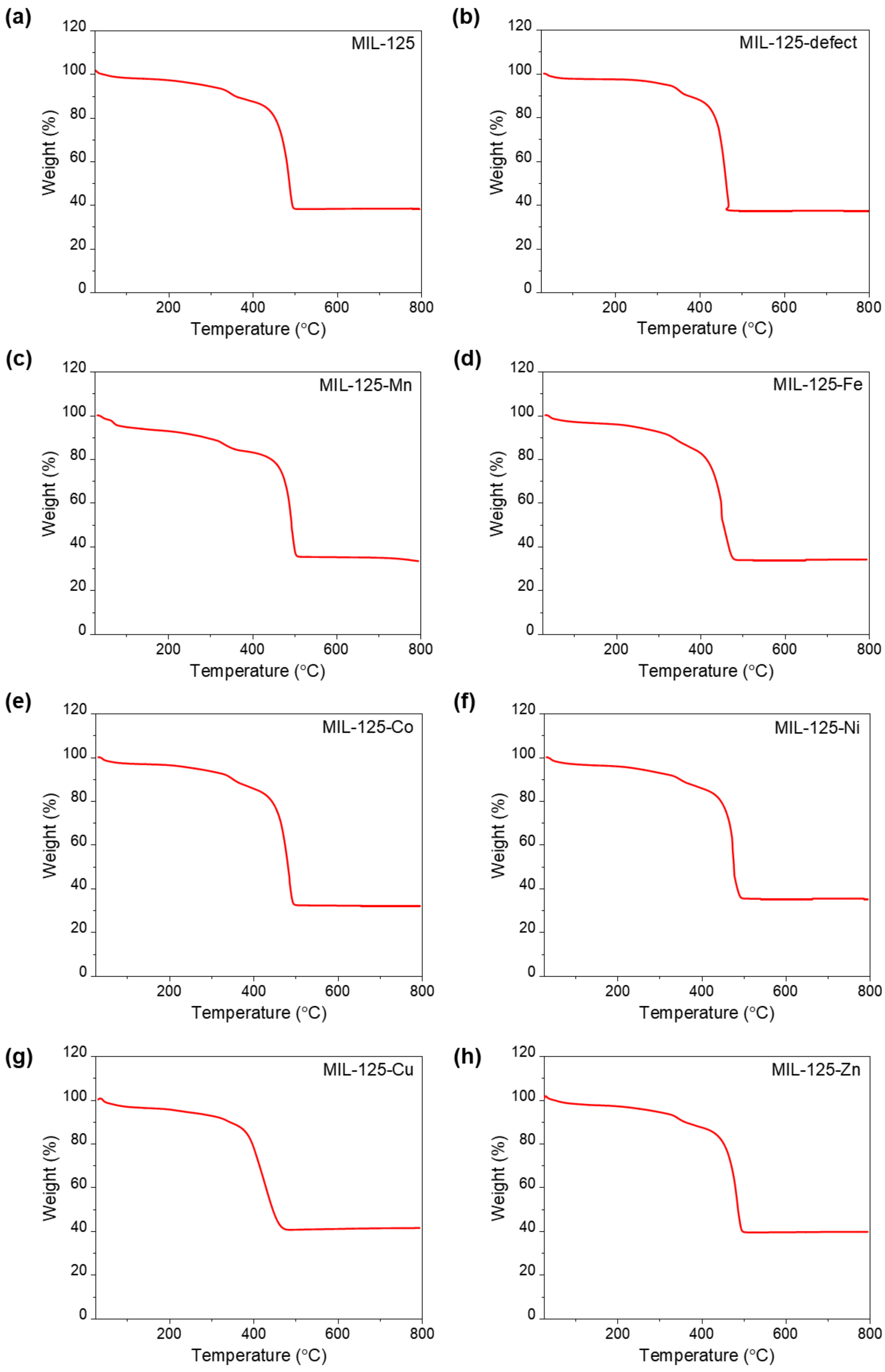

Figure S3. TGA curves of MIL-125, MIL-125-defect and MIL-125-X (X = Mn, Fe, Co, Ni, Cu, Zn). The 62.9% weight loss observed in MIL-125-defect corresponds to a metal-to-ligand molar ratio of 1.14 (theorical value of 1.16), consistent with the ICP-OES results.

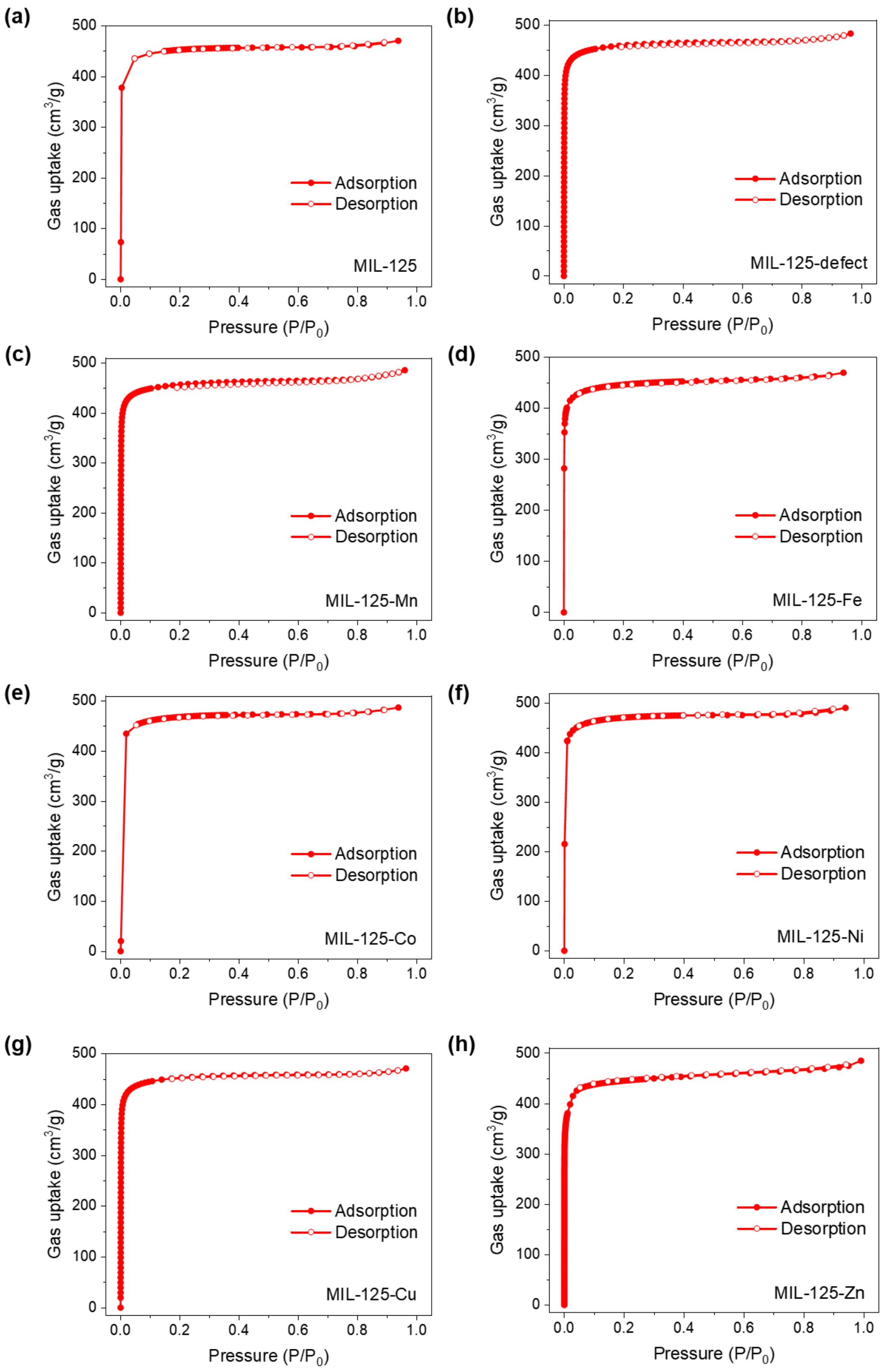

Figure S4. N_2 isotherms for MIL-125, MIL-125-defect and MIL-125-X (X = Mn, Fe, Co, Ni, Cu, Zn) at 77 K (adsorption, solid symbols; desorption, open symbols).

3. X-ray absorption spectroscopy (XAS) and XPS

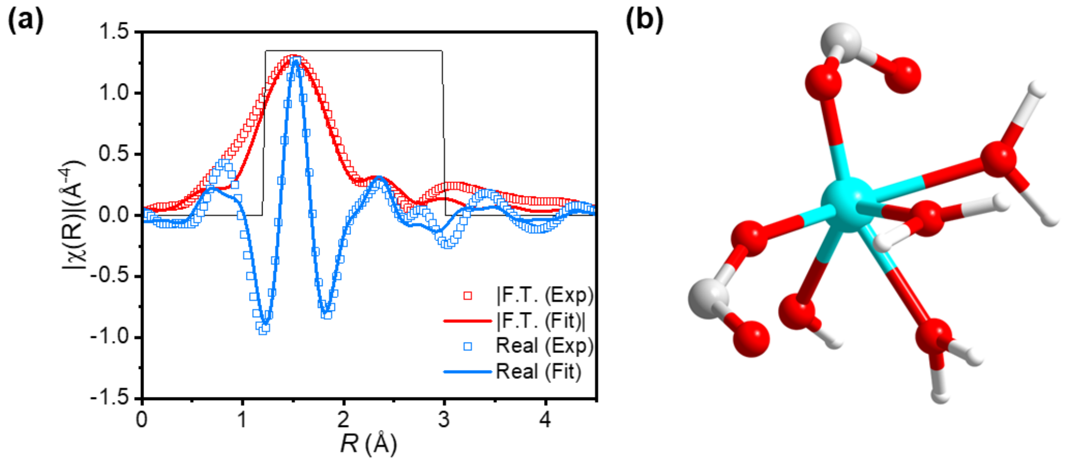

Figure S5. (a) EXAFS spectra and fitted spectra using the model in R-space (1.2-3.0 Å, black lines) at Zn K-edge with the magnitude (hollow squares, red) and real components (hollow squares, blue) of the Fourier transforms. (b) The coordination model of Zn. (Colour code for atoms: Zn, turquoise; O, red; C, grey; H, white.) The EXAFS fitting agrees well with the single crystal model (b) with a Zn–O_{2g} bond length of 1.93 ± 0.003 Å (×1), Zn–O_{2m} bond lengths of 1.98 ± 0.003 Å (×3) and Zn–O_{19a} bond length of 2.15 ± 0.003 Å (×1). Fitting parameters: $3 < k < 8.2$, independent points: 5.73, Number of variables:3, R factor: 0.0195, $\Delta E_o = 8.84 \pm 2.63$, $S_0^2 = 0.973$; $\sigma^2 = 0.003 \pm 0.001$.

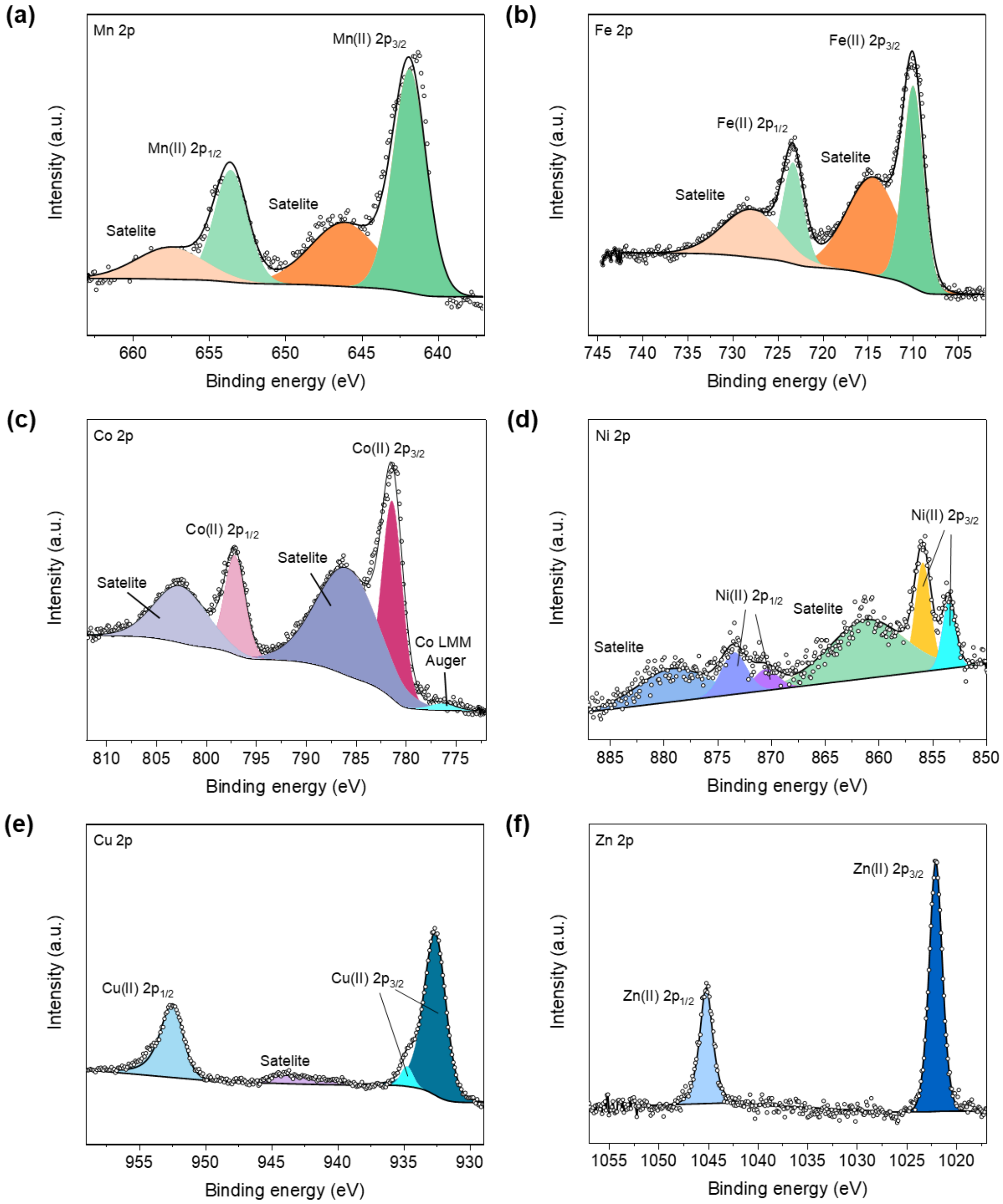

Figure S6. X-ray photoelectron spectra (XPS) of MIL-125-X (X = Mn, Fe, Co, Ni, Cu, Zn). XPS analysis is consistent with the divalent secondary metal sites, and charge calibration was conducted with C 1s at BE 248.8 eV. All element spectra adopted the Shirley or linear type background depending on the background slope, and fittings of the peaks were done with CasaXPS software.

4. Scanning electron microscopy images and energy-dispersive X-ray spectroscopic elemental mapping

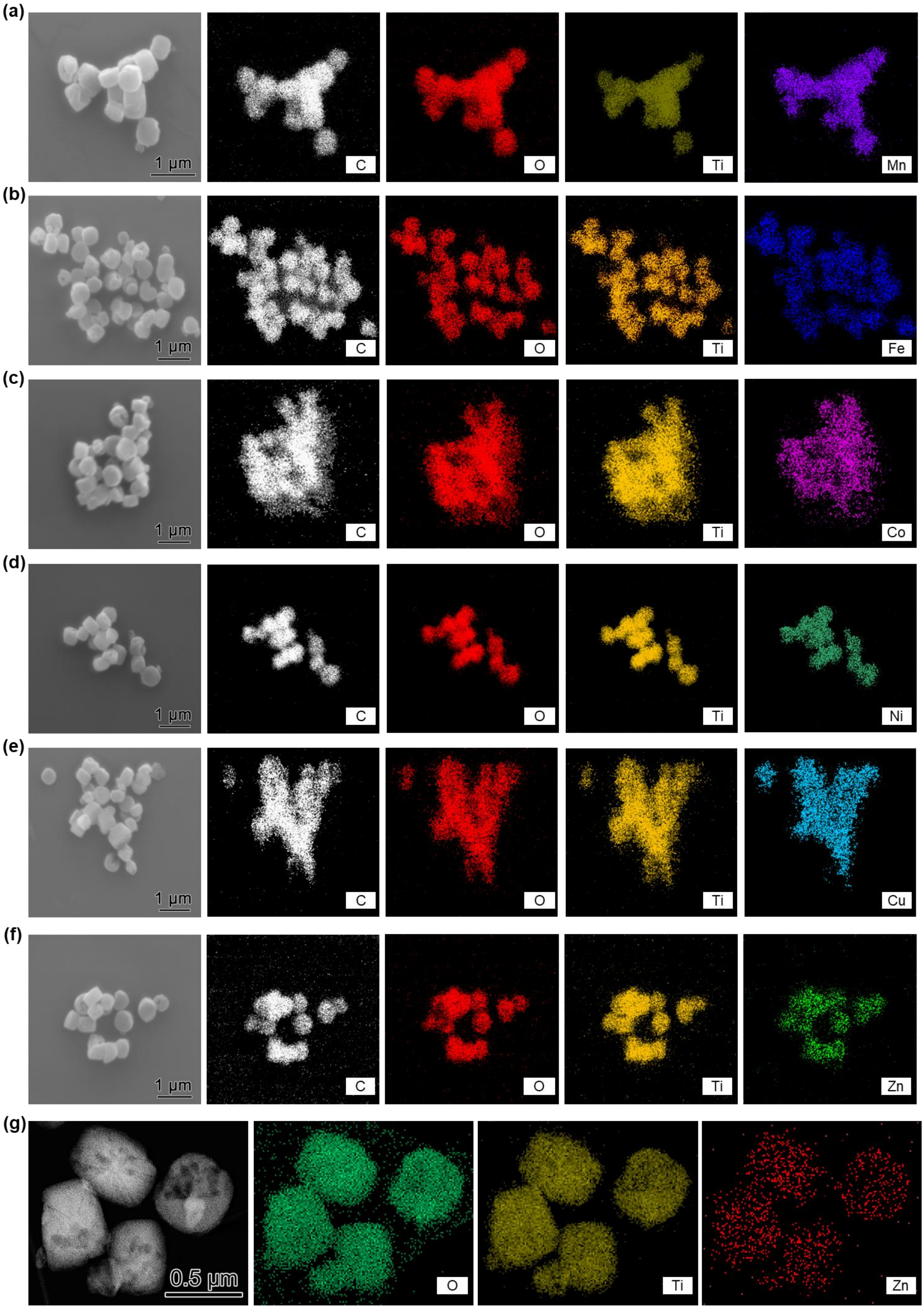

Figure S7. (a-e) SEM images and energy dispersive X-ray spectroscopic (EDS) elemental mapping of MIL-125-X (X = Mn, Fe, Co, Ni, Cu, Zn). (g) STEM images and EDS elemental mapping of MIL-125-Zn.

5. Gas sorption isotherms

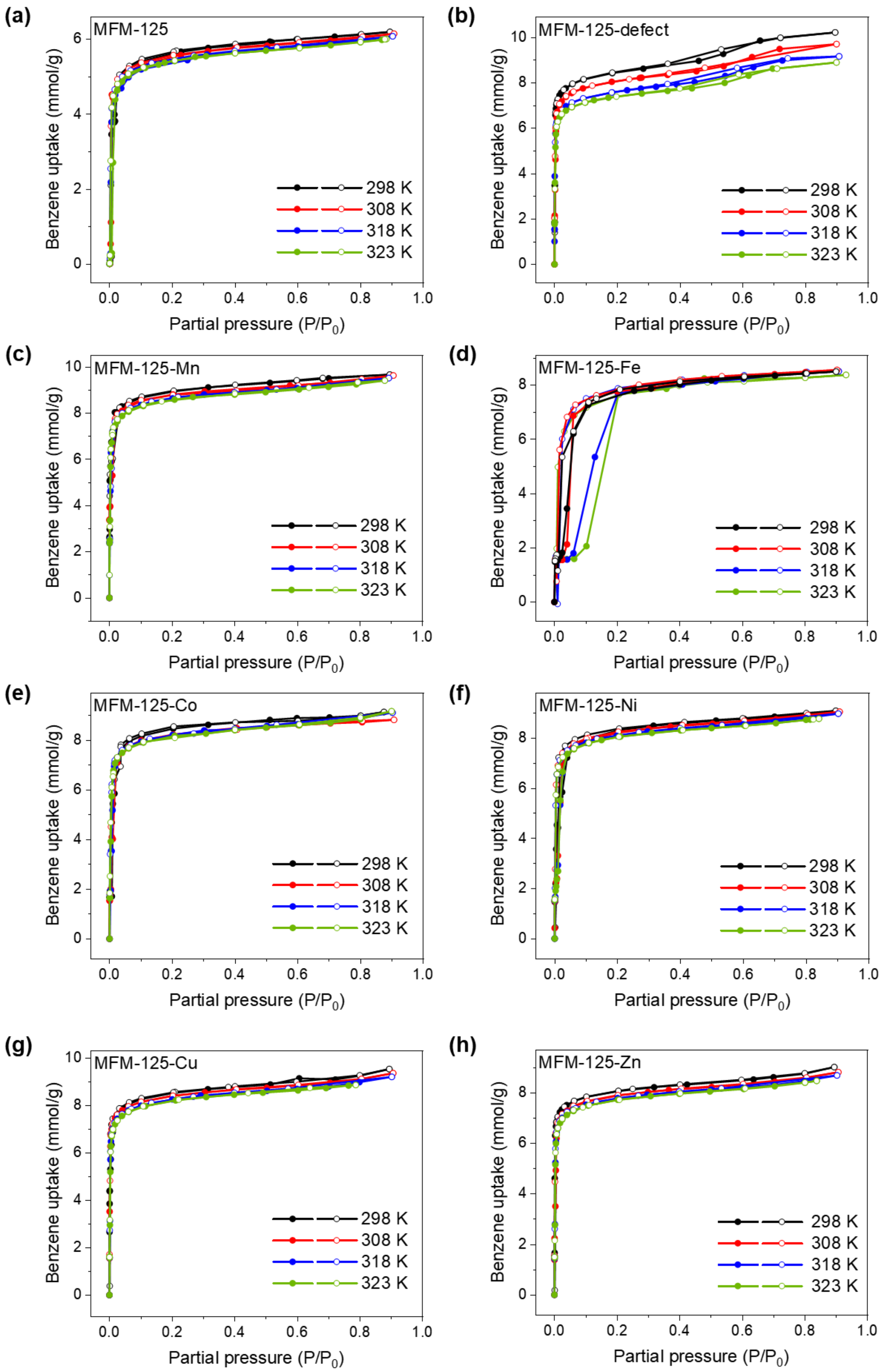

Figure S8. Adsorption isotherms for benzene in MIL-125, MIL-125-defect and MIL-125-X (X = Mn, Fe, Co, Ni, Cu, Zn) at 298-323 K (adsorption, solid symbols; desorption, open symbols).

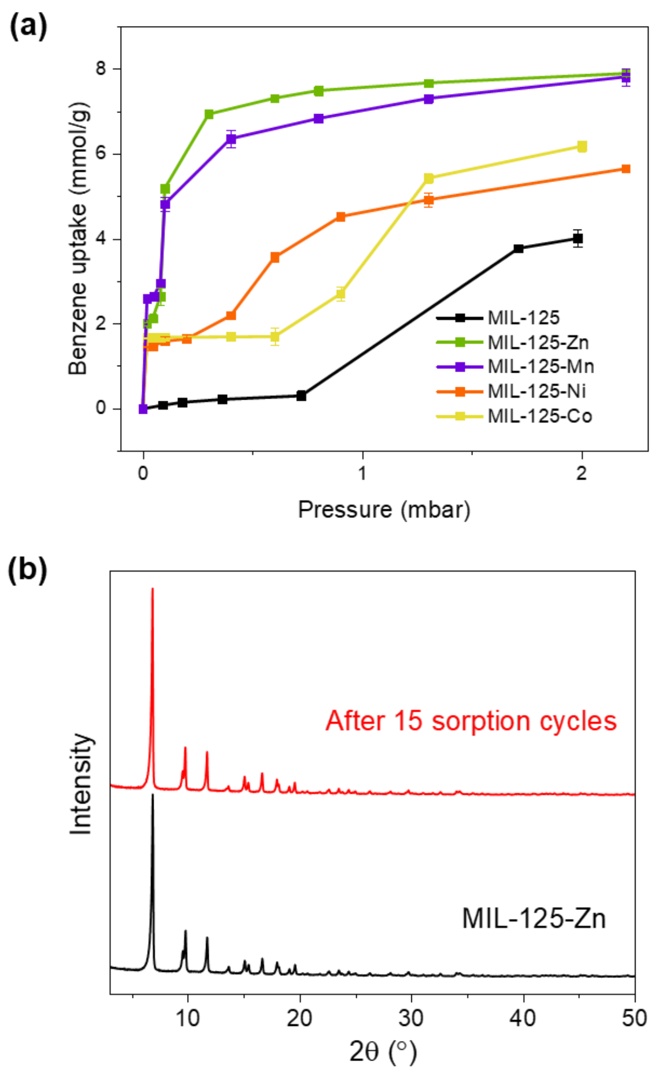

Figure S9. (a) Benzene adsorption isotherms for MIL-125, MIL-125-Mn, MIL-125-Zn, MIL-125-Co and MIL-125-Ni at 298 K and 0-2 mbar. Data points are presented as mean values with error bars indicating standard deviation from three measurements (n = 3). (b) PXRD patterns for MIL-125-Zn samples before and after 15 cycles of adsorption/desorption of benzene at 298 K.

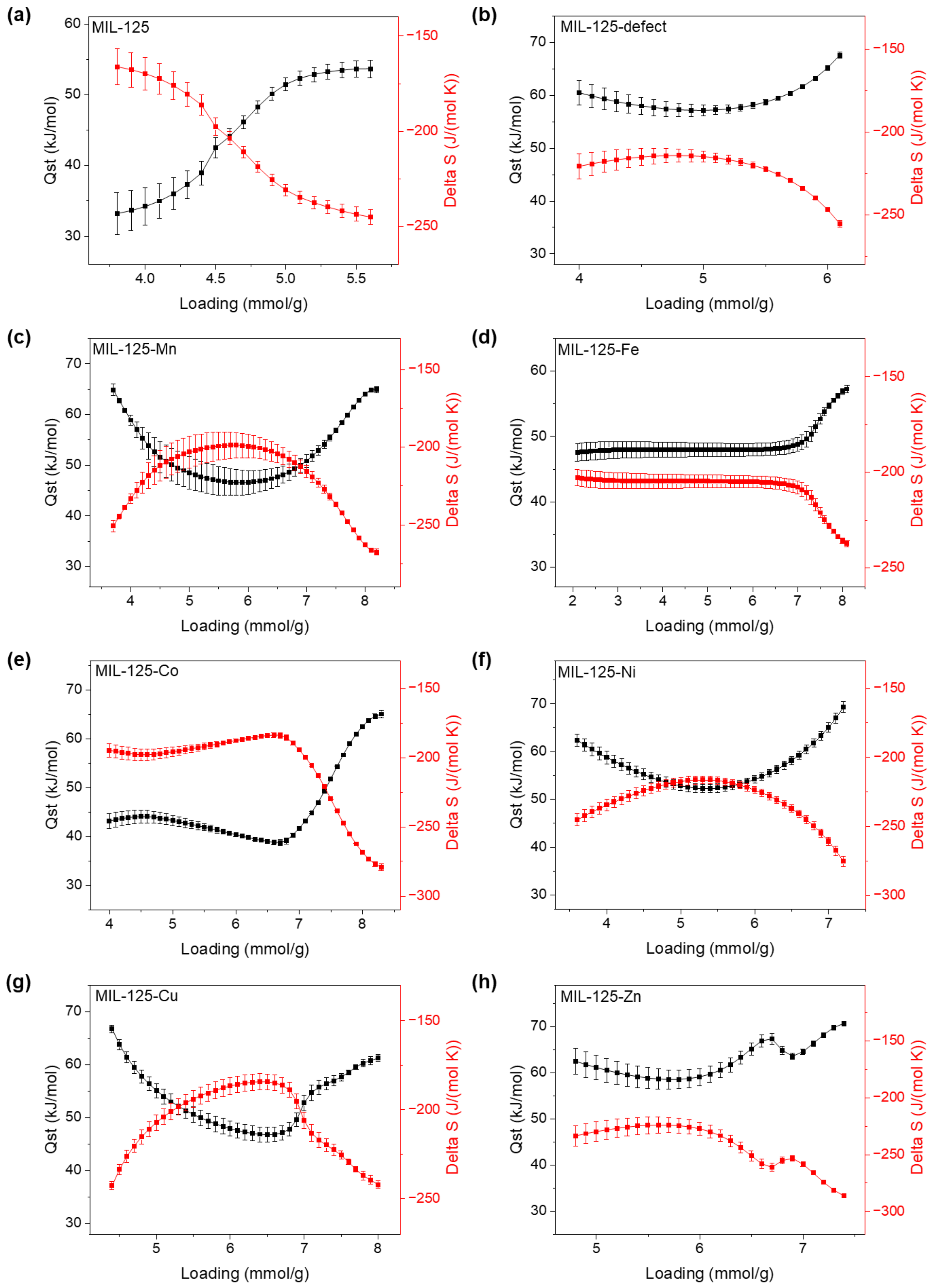

Figure S10. Isosteric heats of adsorption (Q_{st}) and entropy of adsorption of benzene in MIL-125, MIL-125-defect and MIL-125-X (X = Mn, Fe, Co, Ni, Cu, Zn). Data points (Q_{st} and Delta S) are presented as mean values with error bars indicating standard deviation from fitting of all four isotherms at 298, 308, 318 and 323 K to the van't Hoff equation: $ln(p) = \Delta H/RT - \Delta S/R$, where p is the pressure, T is the temperature (K) and R is the real gas constant.

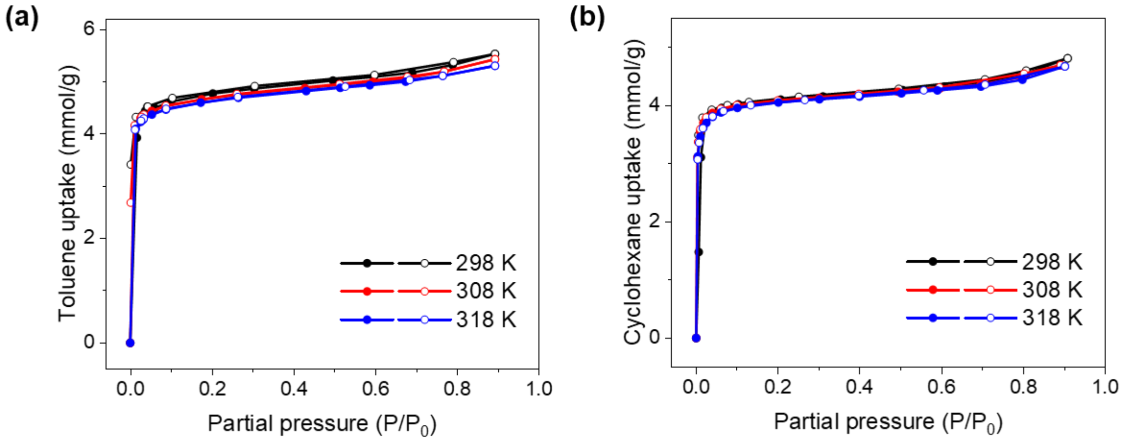

Figure S11. Adsorption isotherms for (a) toluene and (b) cyclohexane in MIL-125-Zn at 298-318 K (adsorption, solid symbols; desorption, open symbols).

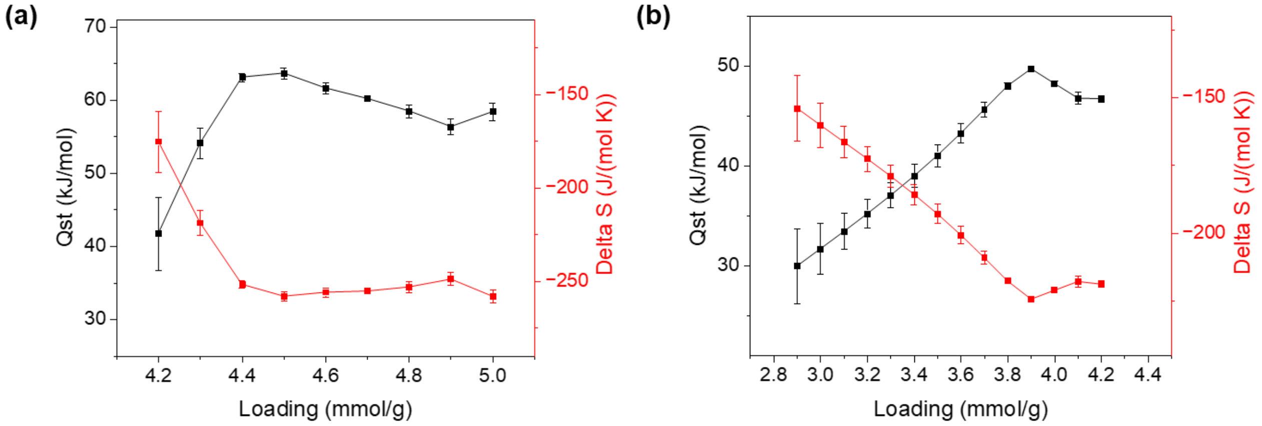

Figure S12. Isosteric heats of adsorption (Q_{st}) and entropy of adsorption for (a) toluene and (b) cyclohexane in MIL-125-Zn. Data points (Q_{st} and Delta S) are presented as mean values with error bars indicating standard deviation from fitting of all three isotherms at 298, 308 and 318 K to the van't Hoff equation: $ln(p) = \Delta H/RT - \Delta S/R$, where p is the pressure, T is the temperature (K) and R is the real gas constant.

Table S1. Summary of benzene adsorption data.

Materials	BET surface area (m^2/g)	Benzene uptake at 298 K, 100 mbar (mmol/g)	Benzene uptake at 298 K, 1.2 mbar (mmol/g)	Benzene uptake at 298 K, 0.12 mbar (mmol/g)
MIL-125	1462	6.14	1.92	0.15
MIL-125-defect	1866	10.05	7.23	3.83
MIL-125-Mn	1854	9.53	7.12	5.06
MIL-125-Fe	1700	8.41	1.56	0.85
MIL-125-Co	1777	8.98	5.43	1.69
MIL-125-Ni	1766	8.94	4.43	1.61
MIL-125-Cu	1831	9.19	6.70	4.43
MIL-125-Zn	1847	9.45	7.63	5.33

Table S2. Comparison of benzene adsorption data reported for solid adsorbent.

No.	Adsorbent name, chemical composition	Structural characteristics: S_{BET} (m^2 g^{-1})	Structural characteristics: Pore size (Å)	Structural characteristics: Structural defect	Adsorption sites and interaction mechanism	Benzene partial pressure at 298K (P/P_0)	Uptake (mmol g^{-1})	Ref.
1	MIL-101(Cr), $Cr_3O(OH)(BDC)_3$	2925	30-40 Å for cages; 12-16 Å for windows.	no	n/a	3.8×10^{-4}	0.50	3
2	UiO-66, $Zr_6O_4(OH)_4(BDC)_6$	1180 – 1240	11 Å for octahedral cage; 8 Å for tetrahedral cage; window aperture of 4.5 Å.[9]	no	Adsorption in the tetrahedral cage *via* π–π and C–H···π interaction.[9]	6.5×10^{-4}	0.73	3
3	HKUST-1, $Cu_3(BTC)_2$	1568.5	Small pore size of 5 Å with window aperture of 3.7 Å; Large pore size of 11 and 14 Å with window aperture of 7 Å.[9]	no	Adsorption at the open metal sites and tetrahedral pores.	9.8×10^{-4}	1.27	4
4	BUT-54, Co(DPN)	1128	Channels of ~ 10.0 Å, and adsorption cavities of ~ 3.7 Å.[9]	no	Adsorption at the wall-to-wall cavities *via* C–H (MOF)···π (benzene), C–H (benzene)···N (MOF) hydrogen bond, C–H (benzene)···π (MOF) interaction. Adsorption in the channel *via* C–H (benzene)···π (MOF) interaction, C–H (benzene)–N (MOF) hydrogen bond.	7.8×10^{-4}<br>1.2×10^{-2}	2.62<br>4.36	4
5	Co(BDP)	n/a	Rectangular 1D channels of ~ 13.2 Å.	no	n/a	2.5×10^{-3}	1.8	4
6	BUT-67 $Zr_6O_4(\mu_3\text{-}OH)_4(NDB)_6$	984	Channel of 7.0 Å, and adsorption cavities of 5.5 Å.[9]	no	n/a	4.3×10^{-3}	2.85	3
7	PAF-1, $[C_{25}H_{16}]$	5600	~7-12 Å 3D pore system.	no	n/a	6.0×10^{-3}	3.3	3
8	Cu^{II}/UiO-66, $Zr_6O_4(OH)_4(BDC)_{5.60}(\text{defect-}Cu^{II})_{0.40}$	1250	Octahedral cage of 11 Å, and tetrahedral cage of 8 Å, window aperture of 4.5 Å. Larger pore at the missed linker and/or cluster sites.[9]	yes	Adsorption in the tetrahedral pore *via* C–H (benzene)···π (MOF), Cu^{II} (MOF)···π (benzene) and –OH (MOF)···π (benzene) interactions. Adsorption in the octahedral pore *via* C–H (MOF)···π (benzene) interaction.	6.0×10^{-3}	3.92	5
9	ZJU-620(Al), $Al_3O_3(HCOO)_3(TMTA)$	1347[9]	Channels of 8.2 Å.	no	Strong adsorption sites at the triangle edge of the channel with C–H (MOF)···π (benzene), and π···π and Al (MOF)···π (benzene) interaction. Weak adsorption site at the trapezoid edge of the channel with C–H (MOF)···π (benzene), and Al (MOF)···π (benzene) interaction.	1.0×10^{-2}	3.8	6
10	BUT-55, Co(BDP)	873	Channels of 8.4 Å, and adsorption cavities of ~ 3.7 Å.	no	Adsorption at the wall-to-wall cavities *via* C–H (MOF)···π (benzene), C–H (benzene)–N (MOF) hydrogen bond, C–H (benzene)···π (MOF) interaction. Adsorption in the channel *via* C–H (benzene)···π (MOF) interaction, C–H (benzene)–N (MOF) hydrogen bond.	5.7×10^{-4}<br>1.6×10^{-2}	3.28<br>3.53	4

11	MFM-300(Sc), $Sc_2(OH)_2(BPTC)$	1228	Channels of 8.1 Å.	no	Adsorption in the channel *via* T-shaped interaction fashion through multiple C–H··· π interactions between benzene and the framework.	6.0×10^{-3}	3.02	5
12	Ni(BPB)	1600[10]	Rhombic channels	no	n/a	1.8×10^{-2}	2.8	4
13	MOF-177, $Zn_4O(BTB)_2$	2970[9]	3D channels with window of ~ 10 Å.[9]	no	n/a	2×10^{-2}	2.69	4
14	Zn(BDP)	n/a	Rectangular 1D channels of ~ 13.2 Å.	no	n/a	4×10^{-3}	1	4
15	MCM-41	1139	3-5 nm 1D mesopores	no	n/a	4.3×10^{-3}	0.15	3
16	ZIF-8, $Zn(MIM)_2$	1510	~12.5 Å for cages<br>~3.3 Å for windows	no	n/a	4.5×10^{-3}	0.03	3
17	Zn(bpb)	2200[10]	Square channels	no	n/a	1.9×10^{-2}	0.65	4
18	ZJU-520(Al), Al(OH)(DBP)	2235	Double-walled channels with pore size distribution (PSD) in the range of 9.26–12.99 Å centred at 10.96 Å.	no	Adsorption at the inorganic node *via* Al – π interactions and C – H···X interactions.<br>Adsorption in the channels *via* C – H···N interactions	3.8×10^{-3}<br>1×10^{-2}	0.52<br>5.98	7
19	BUT-12, $Zr_6O_4(OH)_8(H_2O)_4(L^1)_{8/3}$	3734	Cages of ~13 Å and 1D channels of ~20 Å; PSD of 10 and 20 Å.	no	n/a	1×10^{-2}<br>~1	0.15<br>14.41	8
20	BUT-12-Et, $Zr_6O_4(OH)_8(H2O)_4(L^2)_{8/3}$	3016	Isostructure of BUT-12 with PSD of 6 and 17 Å.	no	n/a	1×10^{-2}<br>~1	0.17<br>13.07	8
21	STA-26, $Zr_6O_4(OH)_8(H_2O)_4(L^1)_{8/3}$	1071	2-fold interpenetrated structure of BUT-12; PSD of ~7 Å.	no	n/a	1×10^{-3}<br>1×10^{-2}<br>~1	1.14<br>4.20<br>4.93	8
22	STA-26-Et, $Zr_6O_4(OH)_8(H2O)_4(L^2)_{8/3}$	710	Isostructure of STA-12 with PSD of ~5 Å.	no	n/a	1×10^{-3}<br>1×10^{-2}<br>~1	2.21<br>4.13<br>4.95	8
	Activated carbon	n/a	n/a	no	n/a	4×10^{-2}	1.67	4
	13X Zeolite	n/a	n/a	no	n/a	5.3×10^{-2}	0.07	4
	MIL-125	1462	Octahedral cage of ~12.6 Å; Tetrahedral cage of 6.1 Å; Triangular windows.	no	Adsorption in the octahedral/tetrahedral cages *via* multiple C–H···π interactions between the framework and benzene; Adsorption in the octahedral cage with extra O–H (framework)···π (benzene).	9×10^{-4}<br>9×10^{-3}	0.15<br>1.92	This work
	MIL-125-defect	1866	MIL-125 with missing metal defects on the $\{Ti_8\}$-ring.	yes	Interactions includes those in MIL-125 and an extra C–H (benzene)···O (terminal carboxylate) interaction.	9×10^{-4}<br>9×10^{-3}	3.83<br>7.23	
	MIL-125-Zn	1847	MIL-125 with Zn^{II}-doped $\{Ti_7Zn\}$-ring.	yes	Interactions includes those in MIL-125-defect and an extra Zn^{II}···π (benzene) interaction.	9×10^{-4}<br>9×10^{-3}	5.33<br>7.63	
	MIL-125-Mn	1854	MIL-125 with Mn^{II}-doped $\{Ti_7Zn\}$-ring.	yes	Interactions includes those in MIL-125-defect and an extra Mn^{II}···π (benzene) interaction	9×10^{-4}<br>9×10^{-3}	5.06<br>7.12	
	MIL-125-Cu	1831	MIL-125 with Cu^{II}-doped $\{Ti_7Zn\}$-ring.	yes	Interactions includes those in MIL-125-defect and an extra Cu^{II}···π (benzene) interaction	9×10^{-4}<br>9×10^{-3}	4.43<br>6.7	
	MIL-125-Co	1777	MIL-125 with Co^{II}-doped $\{Ti_7Zn\}$-ring.	yes	Interactions includes those in MIL-125-defect and an extra Co^{II}···π (benzene) interaction	9×10^{-4}<br>9×10^{-3}	1.69<br>5.43	
	MIL-125-Ni	1766	MIL-125 with Ni^{II}-doped $\{Ti_7Zn\}$-ring.	yes	Interactions includes those in MIL-125-defect and an extra Ni^{II}···π (benzene) interaction	9×10^{-4}<br>9×10^{-3}	1.61<br>4.43	

	MIL-125-Fe	1700	MIL-125 with Fe^{II}-doped {Ti_7Zn}-ring.	yes	Interactions includes those in MIL-125-defect and an extra $Fe^{II}\cdots\pi$ (benzene) interaction	9×10^{-4}<br>9×10^{-3}	0.85<br>1.56	

BDC^{2-} = benzene-1,4-dicarboxylate; BTC^{3-} = benzenetricarboxylate; DPN^{2-} = 2,7-di(1H-pyrazolate-4-yl)naphthalene; NDB^{2-} = 4,4′-(naphthalene-2,7-diyl)dibenzoate; $TMTA^{3-}$ = 4,4′,4″-(2,4,6-trimethylbenzene-1,3,5-triyl) tribenzoate; $BPTC^{4-}$ = biphenyl-3,3′,5,5′-tetracarboxylate; BPB^{2-} = 1,2-bis(pyridine-2-carboxamido)benzoate; BTB = 1,3,5-benzenetribenzoate; MIM^{-1} = 2-methylimidazole; DBP^{2-} = 4,6-di(4-carboxylatephenyl)pyrimidine; H_3L^1 = 5′-(4-carboxyphenyl)-2′,4′,6′-trimethyl-[1,1′:3′,1″-terphenyl]-4,4″-dicarboxylic acid; H_3L^2 = 5′-(4-carboxylatephenyl)-2′,4′,6′-triethyl-(1,1′:3′,1″-terphenyl)-4,4″-dicarboxylic acid.

6. Breakthrough curves

To evaluate the performance of MIL-125-Zn for the capture of trace benzene, breakthrough experiments were conducted under both dry and wet conditions. An air stream containing benzene (5 ppm) was flowed through a fixed-bed of MIL-125-Zn (approximately 10 mg) at a flow rate of 200 mL min^{-1} at atmospheric pressure and room temperature. The concentration of benzene in the eluted stream was assessed using a calibration curve obtained by flowing various benzene/air mixtures diluted with helium (Fig. S38). The breakthrough was defined as 1% of the initial concentration (i.e., 0.05 ppm) being detected in the eluted stream, recorded as 1% breakthrough time. The same experiments were repeated with MIL-125-Zn pre-saturated with water under different humidities (40% and 80% RH), in which minor reductions in the breakthrough time were observed due to the presence of competitive adsorption with water (Fig. 2d).

The regeneration of MIL-125-Zn post benzene adsorption was achieved under a flow of dry helium at 40 mL min^{-1} at atmospheric pressure and room temperature, and complete desorption was observed after 7 h (Fig. S39). PXRD patterns and N_2 isotherms at 77 K were collected for the regenerated MIL-125-Zn, confirming the retention of crystallinity and porosity (Fig. S40 and S41). It is worth noting that the regeneration conditions used here are considerably milder compared with those employed in literature, which often use high temperatures (up to 120 °C).[4] The full regeneration of MIL-125-Zn after benzene adsorption can be achieved in only 10 mins by heating at 80 °C in a flow of dry N_2, as confirmed by *in situ* FTIR spectra (Fig. 4b). Moreover, the isothermal desorption of benzene from MIL-125-Zn has been recorded at 298 K under pressure-swing conditions after initial adsorption (Fig. S42). MIL-125-Zn can be readily regenerated post benzene adsorption upon reduction of pressure at 298 K for 30 minutes, consistent with its excellent reversibility and rapid kinetics for benzene sorption (Fig. 2c).

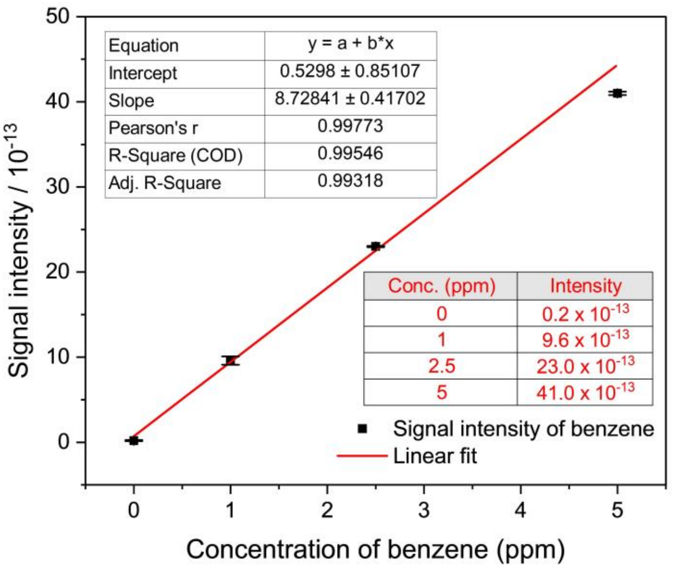

Figure S13. Standard curve for the concentration of benzene. Data points are presented as mean values with error bars indicating standard deviation from three measurements by mass spectrometry after reaching steady state.

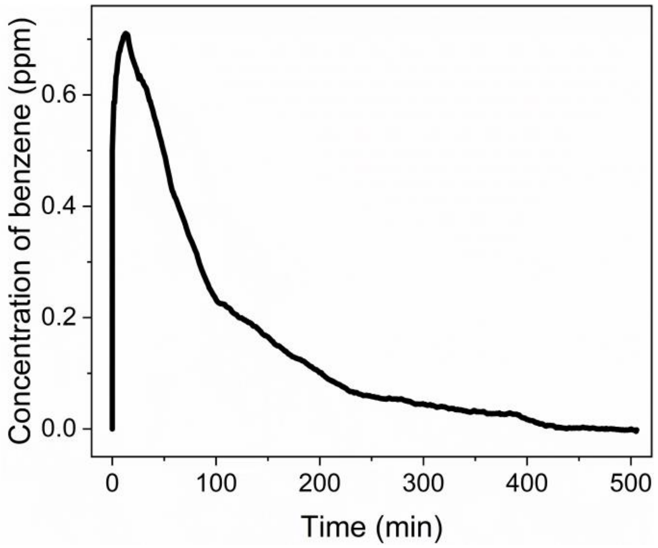

Figure S14. Benzene desorption curve for MIL-125-Zn under a flow of pure He at a rate of 40 mL/min at 298 K and atmospheric pressure.

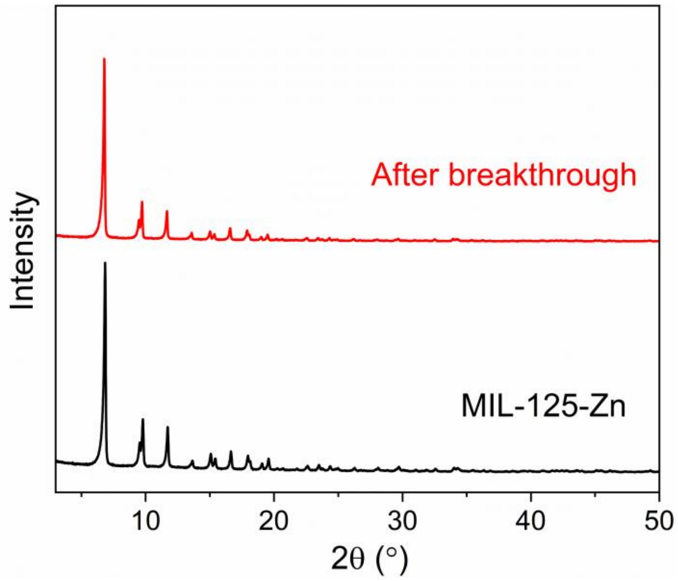

Figure S15. PXRD patterns of MIL-125-Zn before and after the breakthrough experiment.

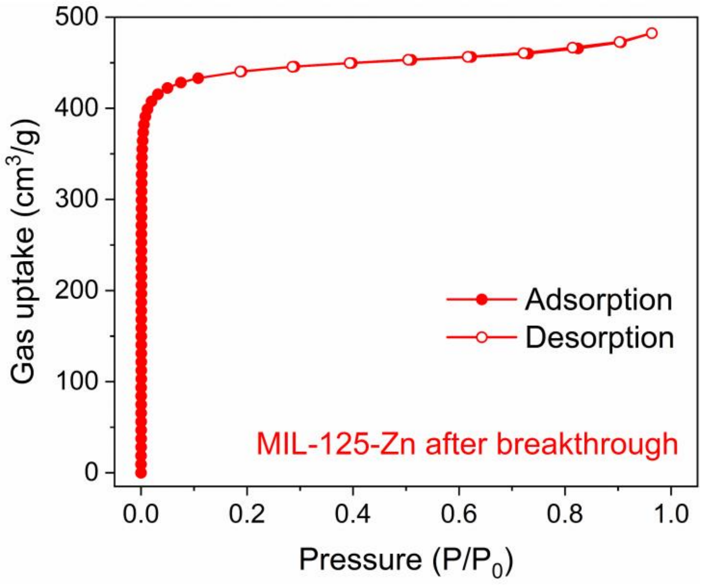

Figure S16. N_2 isotherms at 77 K for regenerated MIL-125-Zn post breakthrough experiments (adsorption, solid symbols; desorption, open symbols).

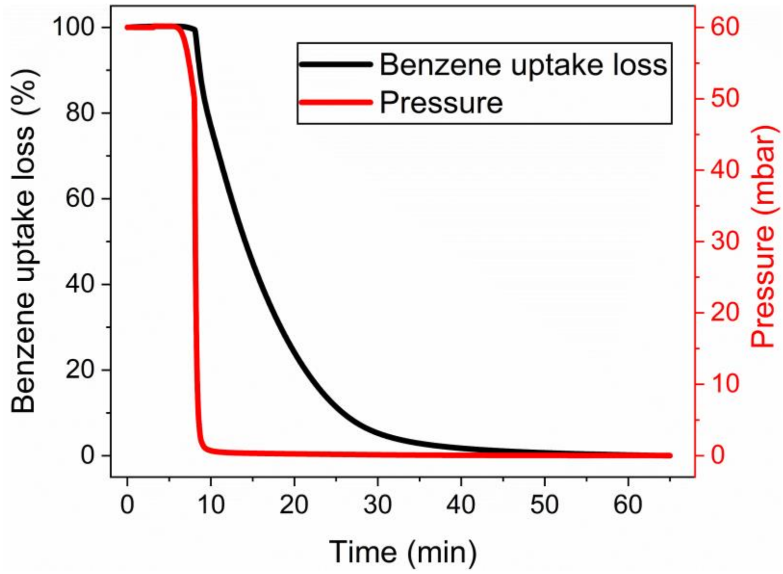

Figure S17. The isothermal desorption profiles of benzene from MIL-125-Zn, observed post-adsorption at 298 K, confirming that MIL-125-Zn can be readily regenerated using pressure-swing conditions.

7. Membrane-based separation

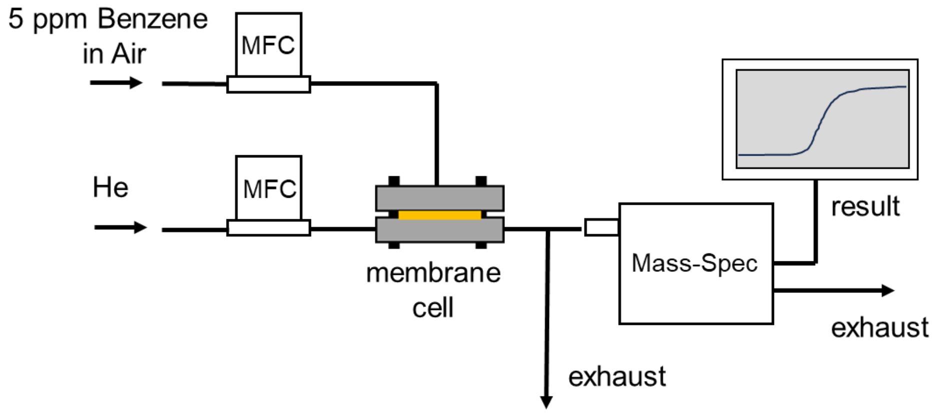

Figure S18. Schematic of the membrane separation test rig.

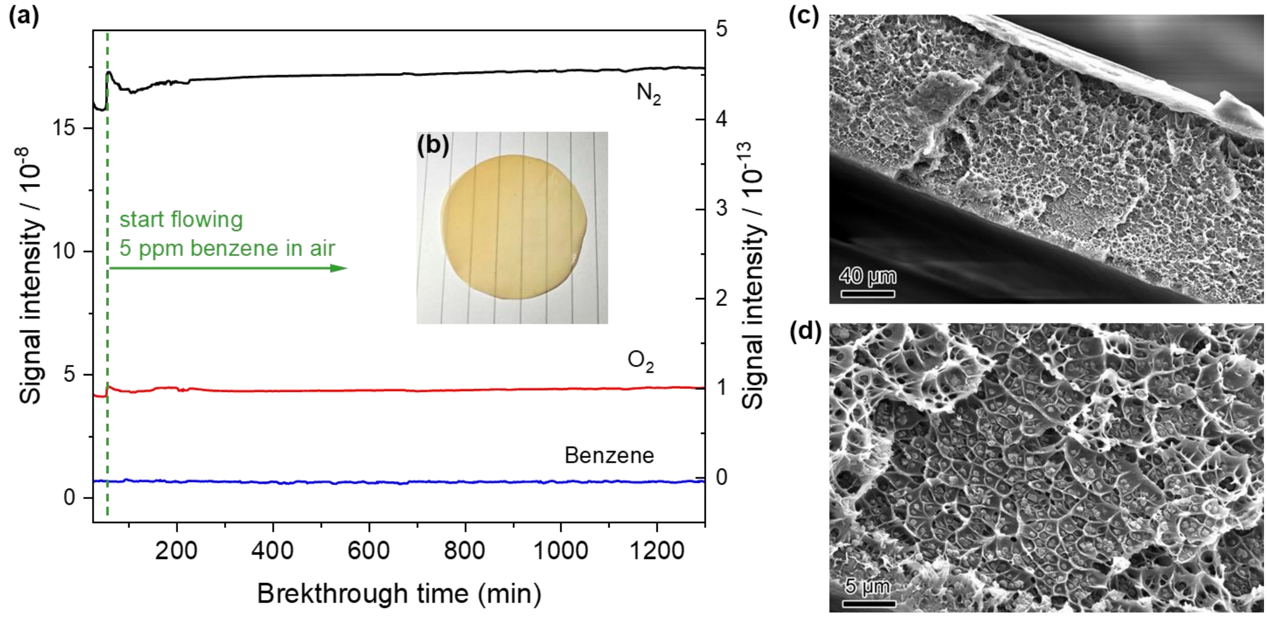

Figure S19. Separation experiments and cross-section SEM images of 15 wt.% MIL-125-Zn/6FDA-Durene membrane. (a) Mass spectrometric signals of permeate after flow through membrane. (b) Photograph of the membrane. (c, d) Cross-section SEM images of the membrane at different magnifications.

8. Rietveld refinement of NPD and SPXRD patterns

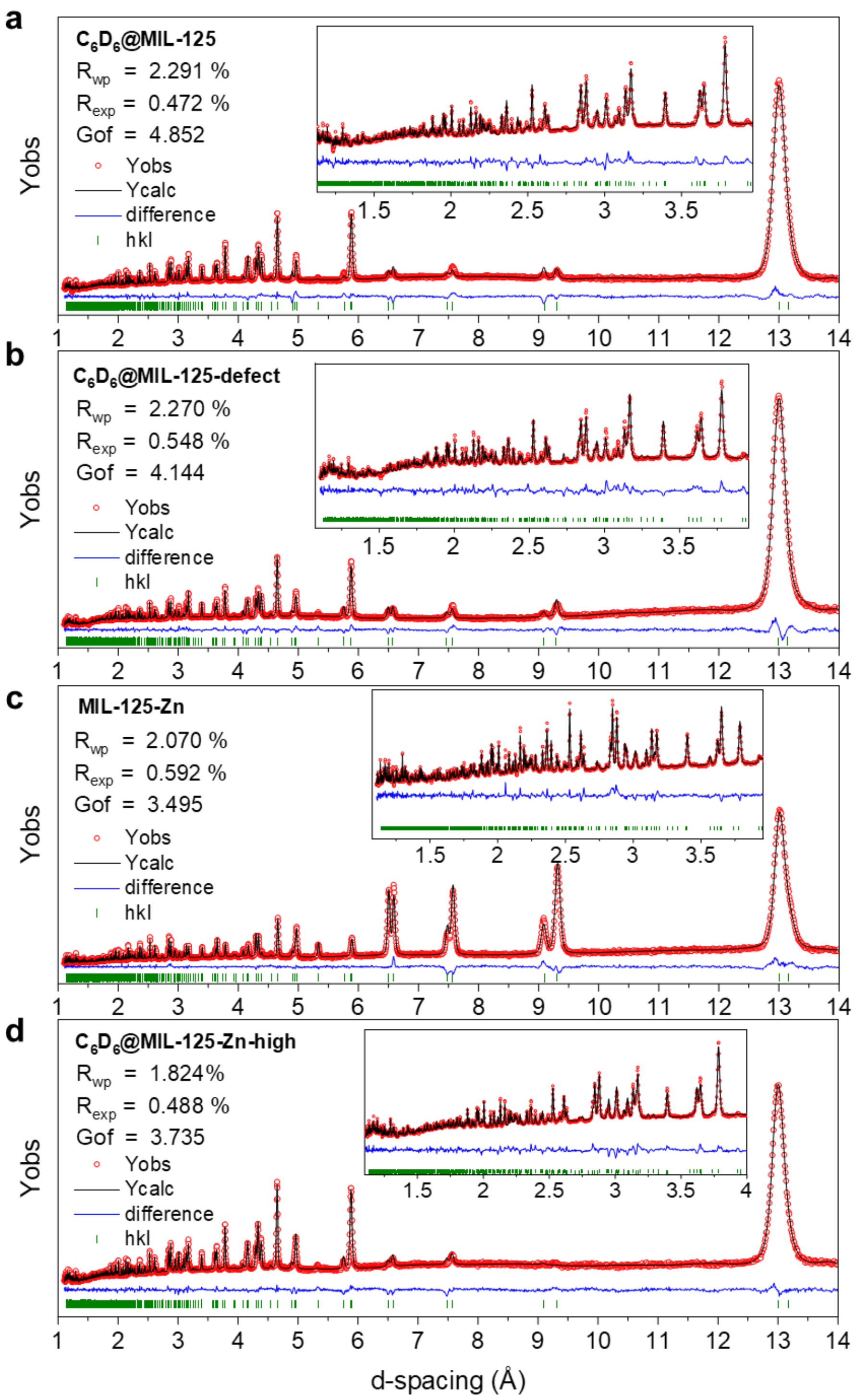

Figure S20. Rietveld refinement of NPD patterns of (a) C_6D_6@MIL-125, (b) C_6D_6@MIL-125-defect, (c) MIL-125-Zn and (d) C_6D_6@MIL-125-Zn-high. Insets are enlarged fitting range of 1-4 Å.

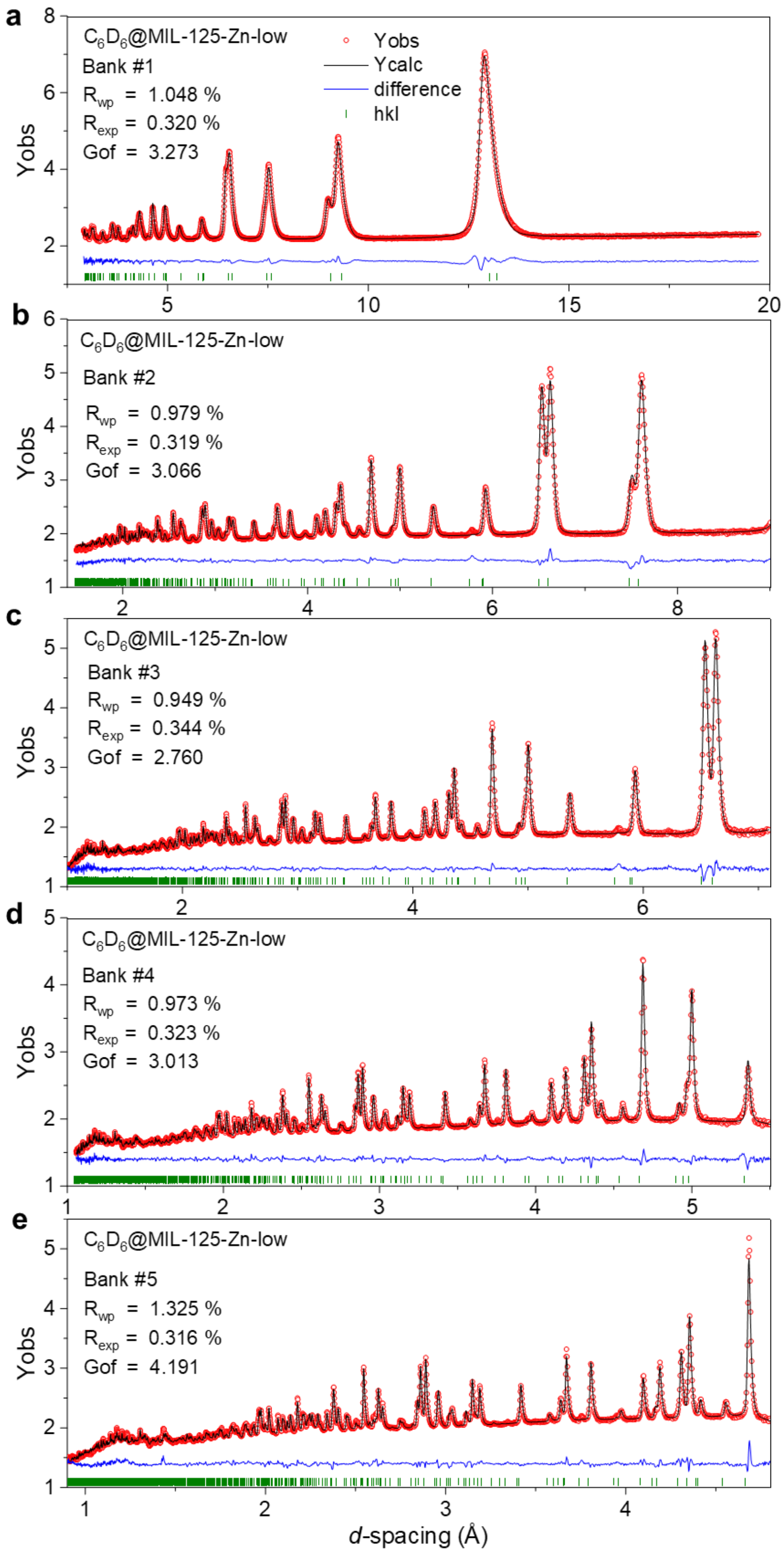

Figure S21. Rietveld refinement of of NPD patterns of C_6D_6@MIL-125-Zn-low.

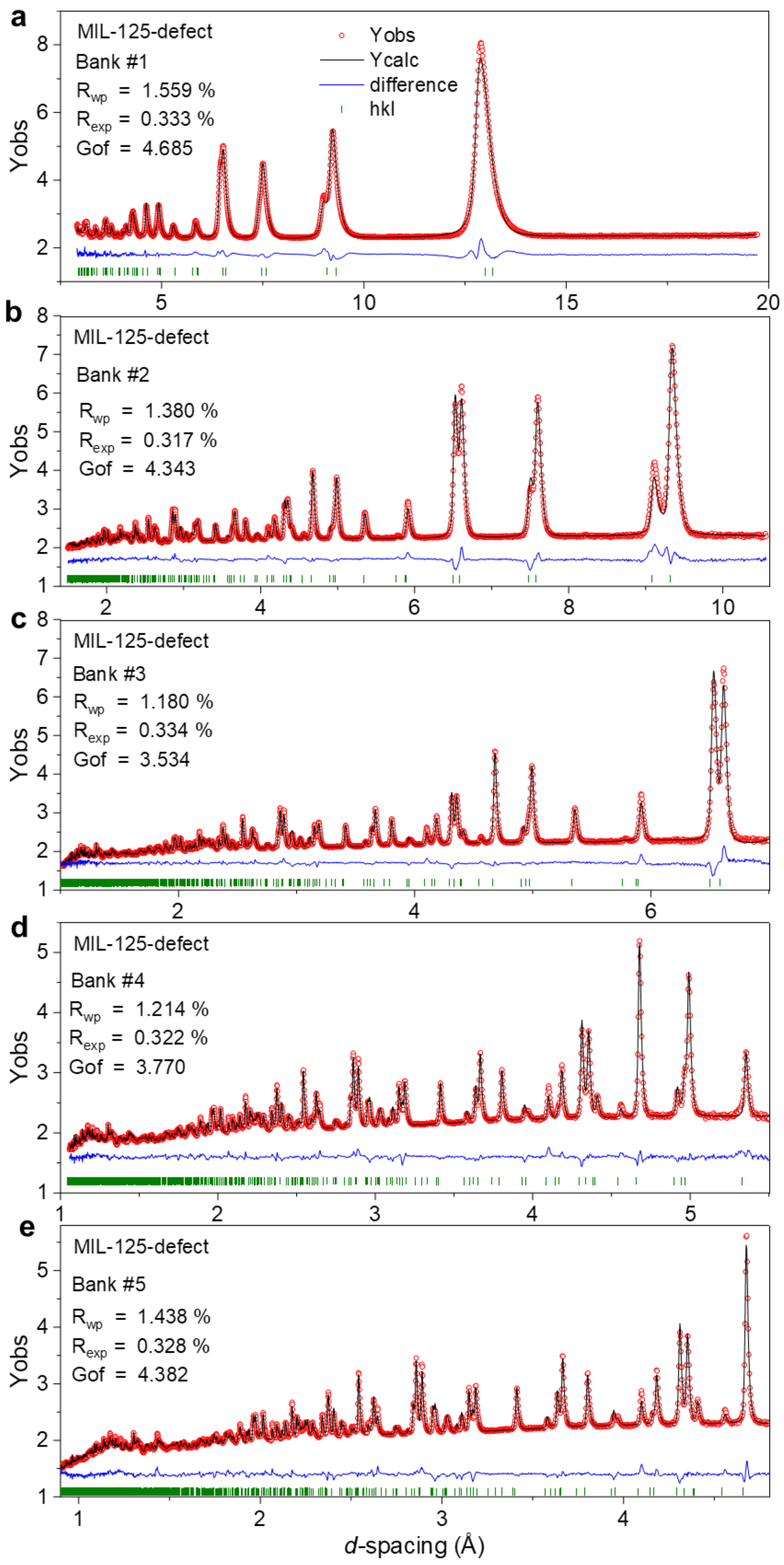

Figure S22. Rietveld refinement of NPD patterns of MIL-125-defect.

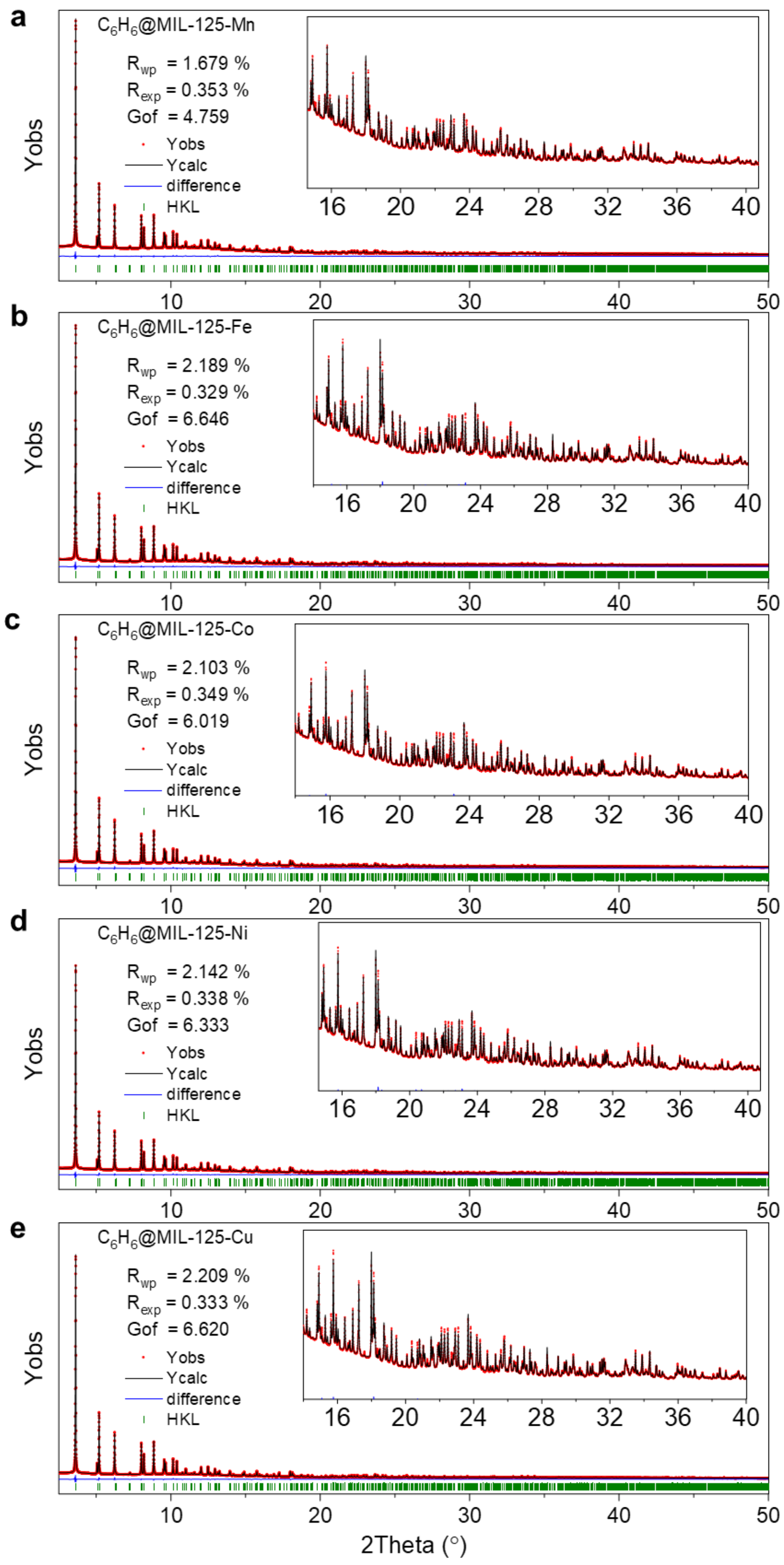

Figure S23. Rietveld refinement of SPXRD (λ = 0.824388 Å) patterns of C_6H_6-loaded MIL-125-X (X = Mn, Fe, Co, Ni, Cu).

9. Crystallographic data for crystal structures

Table S3. Crystallographic details of structure from Rietveld refinement on NPD and SPXRD patterns.

**Samples**	**C_6D_6@MIL-125**	**C_6D_6@MIL-125-defect**	**MIL-125-defect**	**MIL-125-Zn**
CCDC	2301732	2301735	2301737	2301736
Formula	$[Ti_8O_8(OH)_4BDC_6](C_6D_6)_{7.38}$	$[(Ti_8O_8(OH)_4)_{0.87}(defect)_{0.13}BDC_6](C_6D_6)_{6.84}$	$[(Ti_8O_8(OH)_4)_{0.89}(defect)_{0.11}\ BDC_6](CH_3OH)_{3.02}$	$[(Ti_8O_8(OH)_4)_{0.90}Zn_{0.1}(OH)_{0.1}BDC_6](CH_3OH)_{2.0}$
Crystal system	Tetragonal	Tetragonal	Tetragonal	Tetragonal
Space group	$I4_1$/mmm (139)	$I4_1$/mmm (139)	$I4_1$/mmm (139)	$I4_1$/mmm (139)
a, *b* (Å)	18.6015(9)	18.5706(11)	18.6200(1)	18.6250(1)
c (Å)	18.1692(9)	18.1366(11)	18.1533(2)	18.1596(3)
Vol. (Å^3)	6286.8(7)	6254.7(8)	6293.8(1)	6299.4(2)
ρ (calc) g/cm^3	1.130	1.075	0.845	0.862
Diffractometer	POWGEN, ORNL	POWGEN, ORNL	WISH, ISIS	POWGEN, ORNL
Radiation	Neutron	Neutron	Neutron	Neutron
Method	Time of flight	Time of flight	Time of flight	Time of flight
Temperature	10 K	10 K	10 K	10 K
Refinement *d*-spacing range	1-14 Å	1-14 Å	0.7-20 Å	1-14 Å
$R_{wp}/R_{exp}/R_p$ (%)	2.291/0.472/1.899	2.270/0.548/2.093	1.352/0.328/1.090	2.070/0.592/2.004
GoF (χ^2)	4.853	4.144	4.115	3.495
**Samples**	**C_6D_6@MIL-125-Zn-high**	**C_6D_6@MIL-125-Zn-low**	**C_6H_6@MIL-125-Mn**	**C_6H_6@MIL-125-Fe**
CCDC	2301734	2301733	2301739	2301738
Formula	$[(Ti_8O_8(OH)_4)_{0.83}Zn_{0.17}(OH)_{0.7}BDC_6](C_6D_6)_{10.59}$	$[(Ti_8O_8(OH)_4)_{0.87}Zn_{0.13}(OH)_{0.3}BDC_6](C_6D_6)_{1.38}$	$[(Ti_8O_8(OH)_4)_{0.89}Mn_{0.11}(OH)_{0.11}BDC_6](C_6H_6)_{8.928}$	$[(Ti_8O_8(OH)_4)_{0.89}Fe_{0.11}(OH)_{0.11}BDC_6](C_6H_6)_{10.06}$
Crystal system	Tetragonal	Tetragonal	Tetragonal	Tetragonal
Space group	$I4_1$/mmm (139)	$I4_1$/mmm (139)	$I4_1$/mmm (139)	$I4_1$/mmm (139)
a, *b* (Å)	18.6048(6)	18.6461(1)	18.6539(1)	18.6554(1)
c (Å)	18.1660(6)	18.1348(2)	18.2054(1)	18.2026(1)
Vol. (Å^3)	6288.0(4)	6305.0(1)	6293.8(1)	6334.9(1)
ρ (calc) g/cm^3	1.270	0.886	1.185	1.232
Diffractometer	POWGEN, ORNL	POWGEN, ORNL	I11, DLS	I11, DLS
Radiation	Neutron	Neutron	Synchrotron X-ray	Synchrotron X-ray
Method	Time of flight	Time of flight	Capillary, PSD detector	Capillary, PSD detector
Temperature	10 K	10 K	298 K	298 K
Refinement range	1-14 Å	1-14 Å	2.5-50° (λ = 0.824388 Å)	2.5-50° (λ = 0.824388 Å)
$R_{wp}/R_{exp}/R_p$ (%)	1.824/0.488/1.613	2.270/0.548/2.093	1.679 /0.328/1.177	2.189/0.329/1.470
GoF (χ^2)	3.735	4.144	4.759	6.646

Samples	C_6H_6@MIL-125-Co	C_6H_6@MIL-125-Ni	C_6H_6@MIL-125-Cu
CCDC	2301740	2301741	2301742
Formula	$[(Ti_8O_8(OH)_4)_{0.90}Co_{0.10}(OH)_{0.10}BDC_6](C_6H_6)_{8.91}$	$[(Ti_8O_8(OH)_4)_{0.89}Ni_{0.11}(OH)_{0.11}BDC_6](C_6H_6)_{9.28}$	$[(Ti_8O_8(OH)_4)_{0.89}Cu_{0.11}(OH)_{0.11}BDC_6](C_6H_6)_{9.71}$
Crystal system	Tetragonal	Tetragonal	Tetragonal
Space group	$I4_1$/mmm (139)	$I4_1$/mmm (139)	$I4_1$/mmm (139)
a, *b* (Å)	18.6497(1)	18.6512(1)	18.6554(1)
c (Å)	18.1942(1)	18.2022(1)	18.2052(1)
Vol. (Å^3)	6328.2(1)	6331.9(1)	6335.8(1)
ρ (calc) g/cm^3	1.187	1.188	1.221
Diffractometer	I11, DLS	I11, DLS	I11, DLS
Radiation	Synchrotron X-ray	Synchrotron X-ray	Synchrotron X-ray
Method	Capillary, PSD detector	Capillary, PSD detector	Capillary, PSD detector
Temperature	298 K	298 K	298 K
Refinement range	2.5-50° (λ = 0.824388 Å)	2.5-50° (λ = 0.824388 Å)	2.5-50° (λ = 0.824388 Å)
$R_{wp}/R_{exp}/R_p$(%)	2.104/0.349/1.376	2.142/0.338/1.440	2.209/0.334/1.484
GoF (χ^2)	6.019	6.333	6.620

10. Views of crystal structures

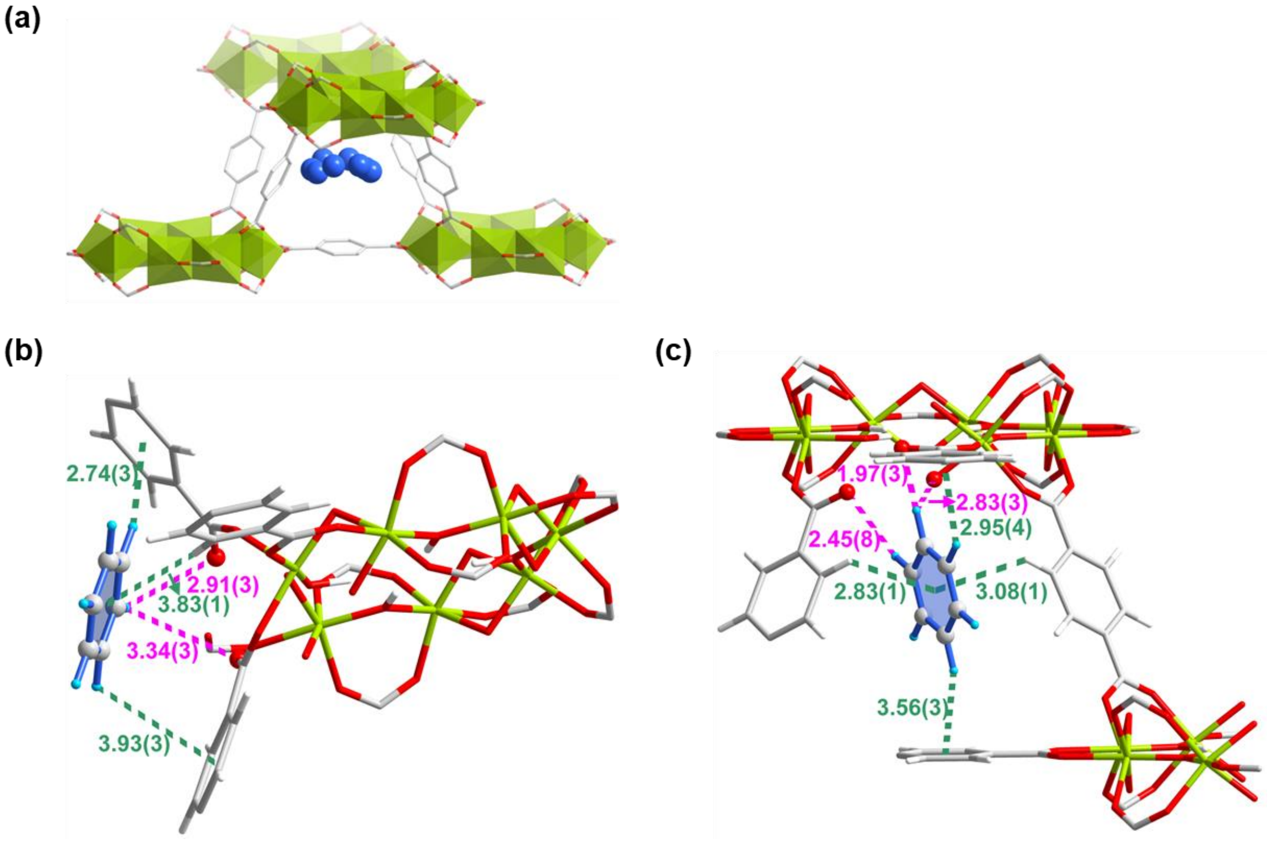

Figure S24. The structure of MIL-125-defect·6.84C_6D_6 with CCDC 2301735. Views of the structural models for the adsorption of d_6-benzene in MIL-125-defect determined from NPD data. (a) Distribution of adsorbed d_6-benzene molecules within the tetrahedral cages in MIL-125-defect. (b,c) Views of host-guest interactions with benzene at site III and site IV in MIL-125-defect. The C–$\underline{D}_{benzene}\cdots O_{defect}$ interactions and C–$\underline{H}\cdots\pi$ interactions are highlighted in pink and sea green, respectively. Color code for atoms: Ti, lime; O, red; C, gray; D, sky blue; H, white.

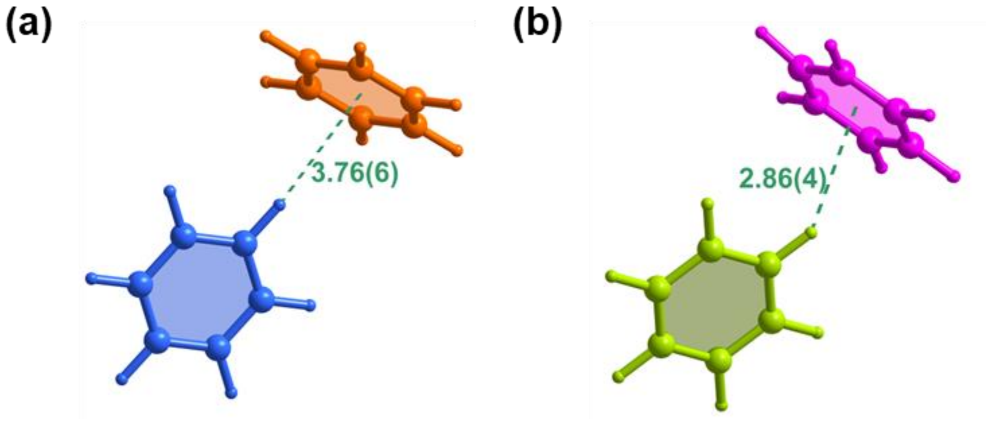

Figure S25. The structure of MIL-125-defect·6.84C_6D_6 (CCDC 2301735). Views of interactions between guest benzene molecules in MIL-125-defect determined from NPD data. Benzene at sites I–IV are colored in pink, green, orange and blue, respectively. The C–H···π interactions are highlighted in sea green.

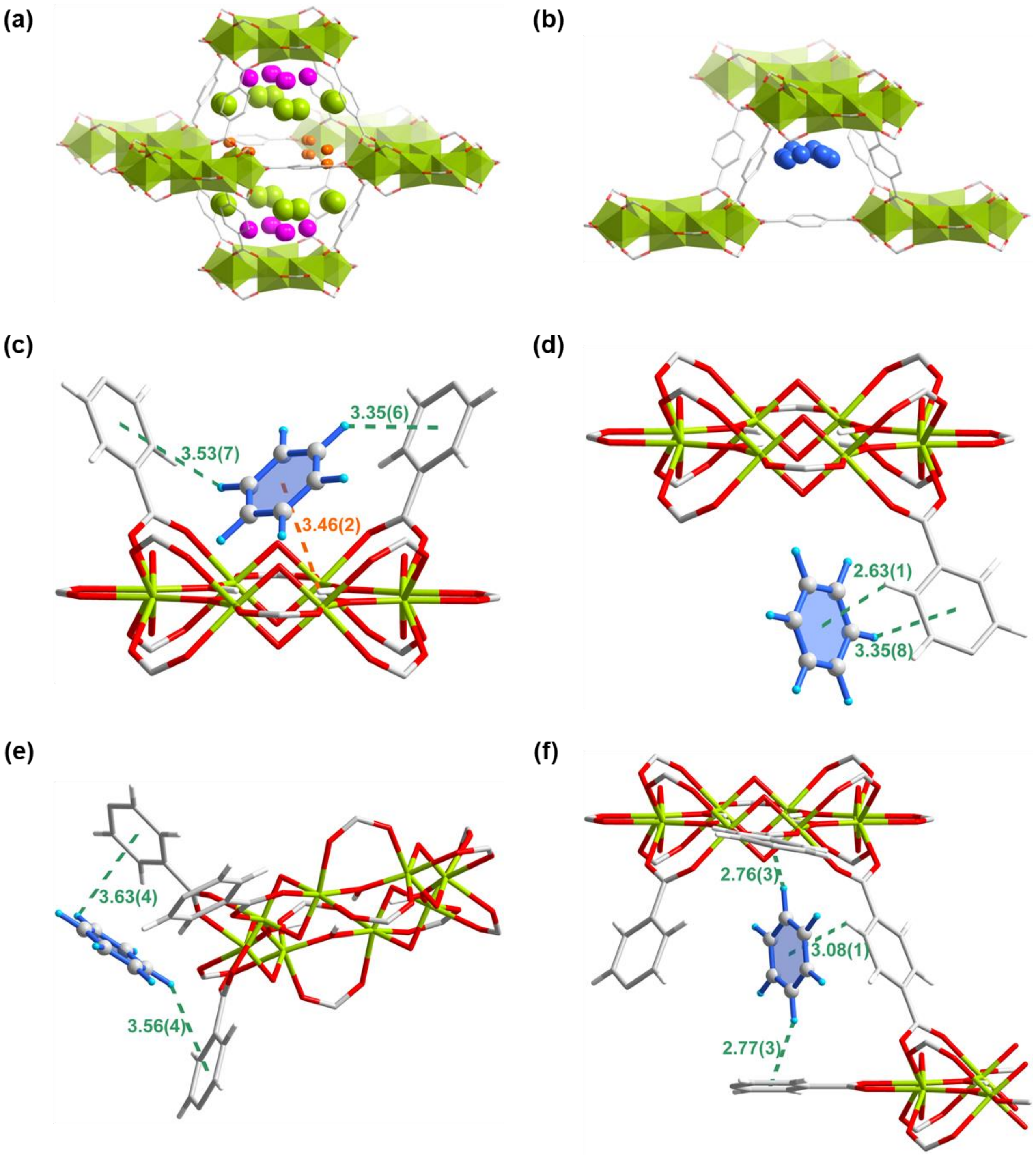

Figure S26. The structure of MIL-125·7.38C_6D_6 (CCDC 2301732). Views of the structural models for the adsorption of d_6-benzene in MIL-125 determined from NPD data. (a,b) Distribution of adsorbed d_6-benzene molecules within the (a) octahedral and (b) tetrahedral cages in MIL-125. Sites I–IV are colored in pink, green, orange and blue, respectively. The radii of the colored balls of different binding sites are proportional to their crystallographic occupancies (Sites I–IV have occupancies of 1.90, 2.59, 1.10 and 1.78 for benzene per {Ti_8} cluster in MIL-125, respectively). (c-f) Views of host-guest interactions with benzene at site I (c), II (d), III (e) and IV (f) in MIL-125. The O–$\underline{H}_{framework}\cdots\pi_{benzene}$ interactions and C–$\underline{H(or\ D)}\cdots\pi$ interactions are highlighted in orange and sea green, respectively. Color code for atoms: Ti, lime; O, red; C, gray; D, sky blue; H, white.

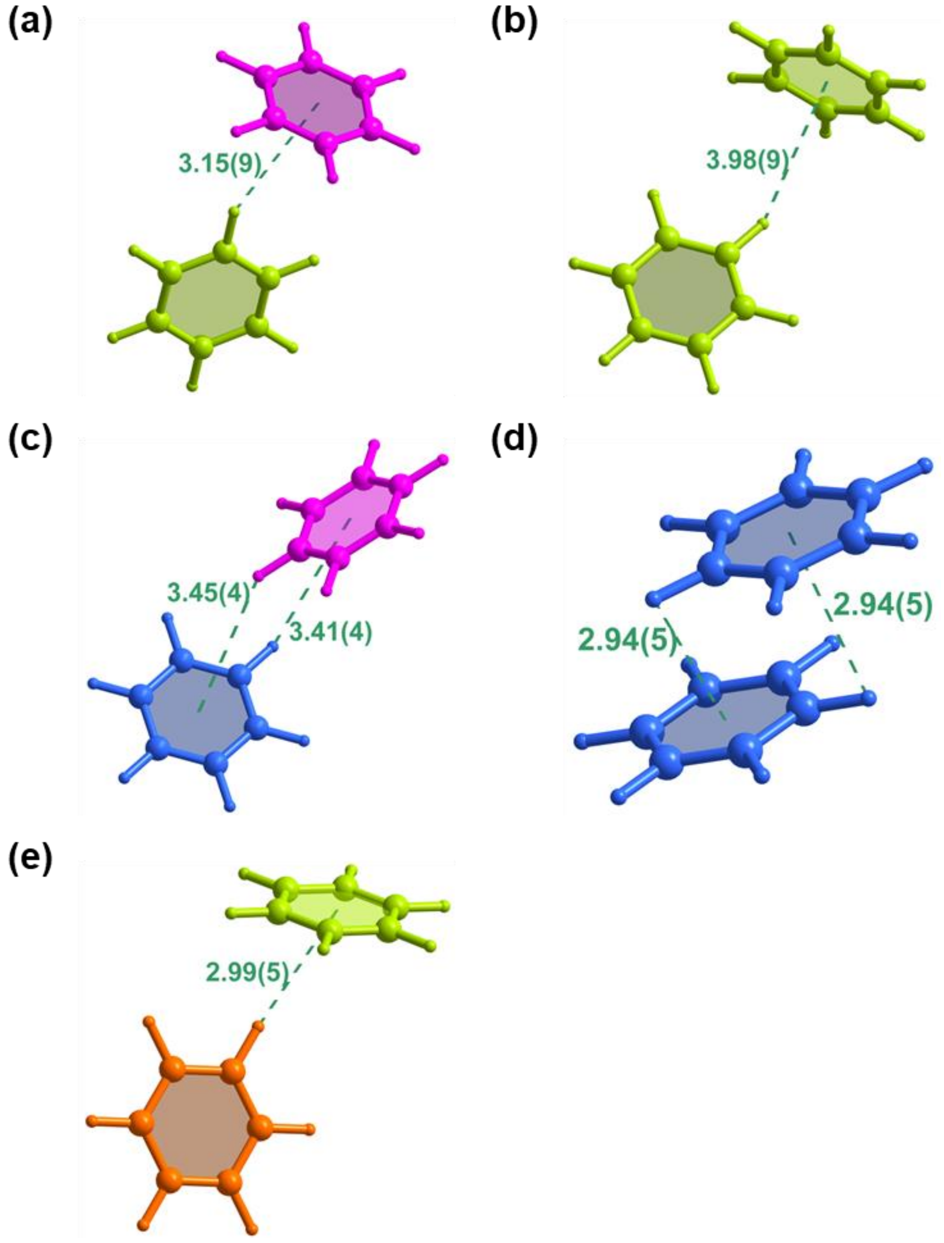

Figure S27. The structure of MIL-125·7.38C_6D_6 (CCDC 2301732). Views of interactions between guest benzene molecules in MIL-125 determined from NPD data. Benzene at sites I–IV are colored in pink, green, orange and blue, respectively. The C–H···π interactions are highlighted in sea green.

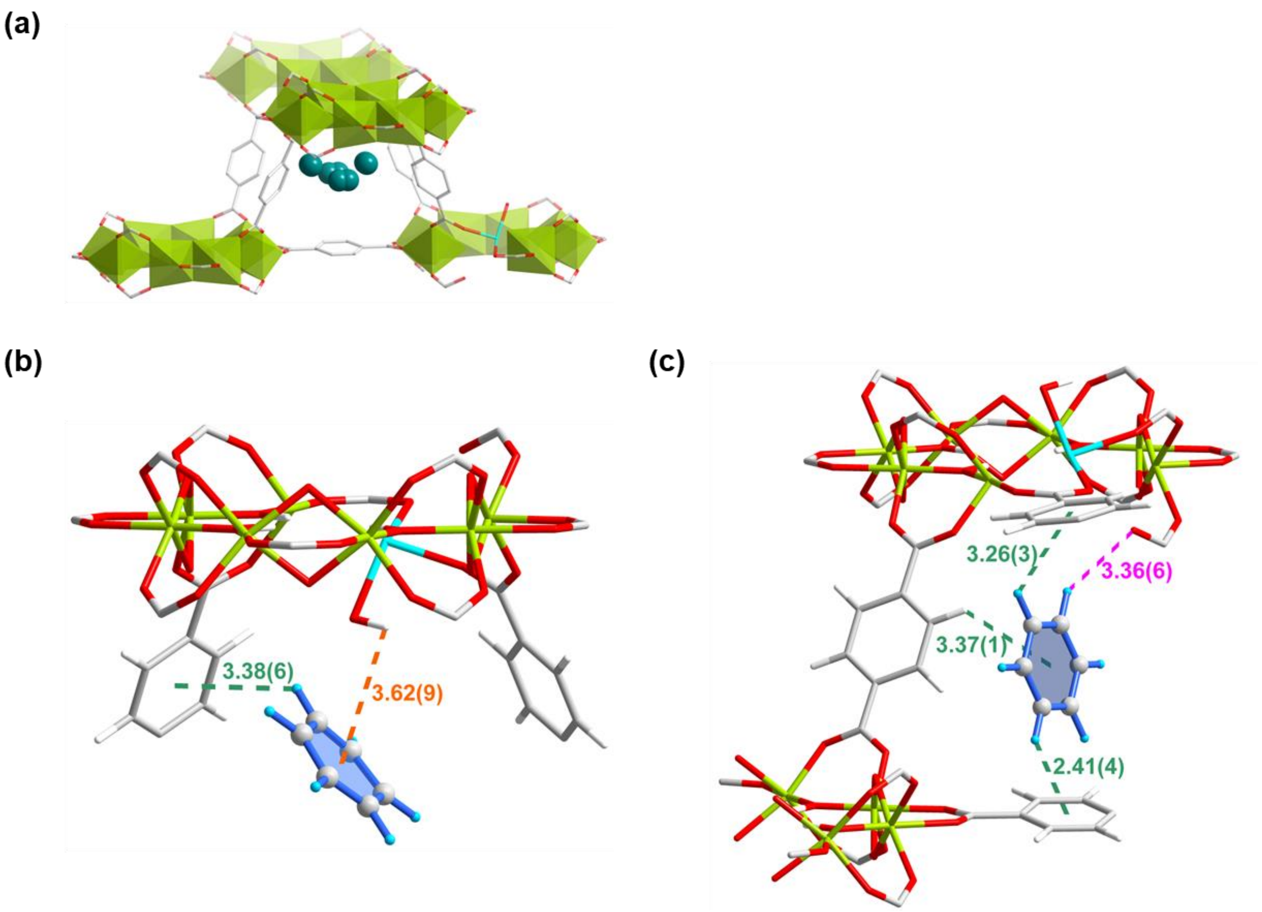

Figure S28. The structure of MIL-125-Zn·10.59C_6D_6 (CCDC 2301734). Views of the structural models for the adsorption of d_6-benzene in MIL-125-Zn determined from NPD data. (a) Distribution of adsorbed d_6-benzene molecules within the tetrahedral cages in MIL-125-Zn. (b,c) Views of host-guest interactions with benzene at site IV and site V in MIL-125-defect. The C–$\underline{D}_{benzene}\cdots O_{defect}$ interactions, O–$\underline{H}_{framework}\cdots\pi_{benzene}$ interactions and C–<u>H(or D)</u>$\cdots\pi$ interactions are highlighted in pink, orange and sea green, respectively. Color code for atoms: Ti, lime; Zn, turquoise; O, red; C, gray; D, sky blue; H, white.

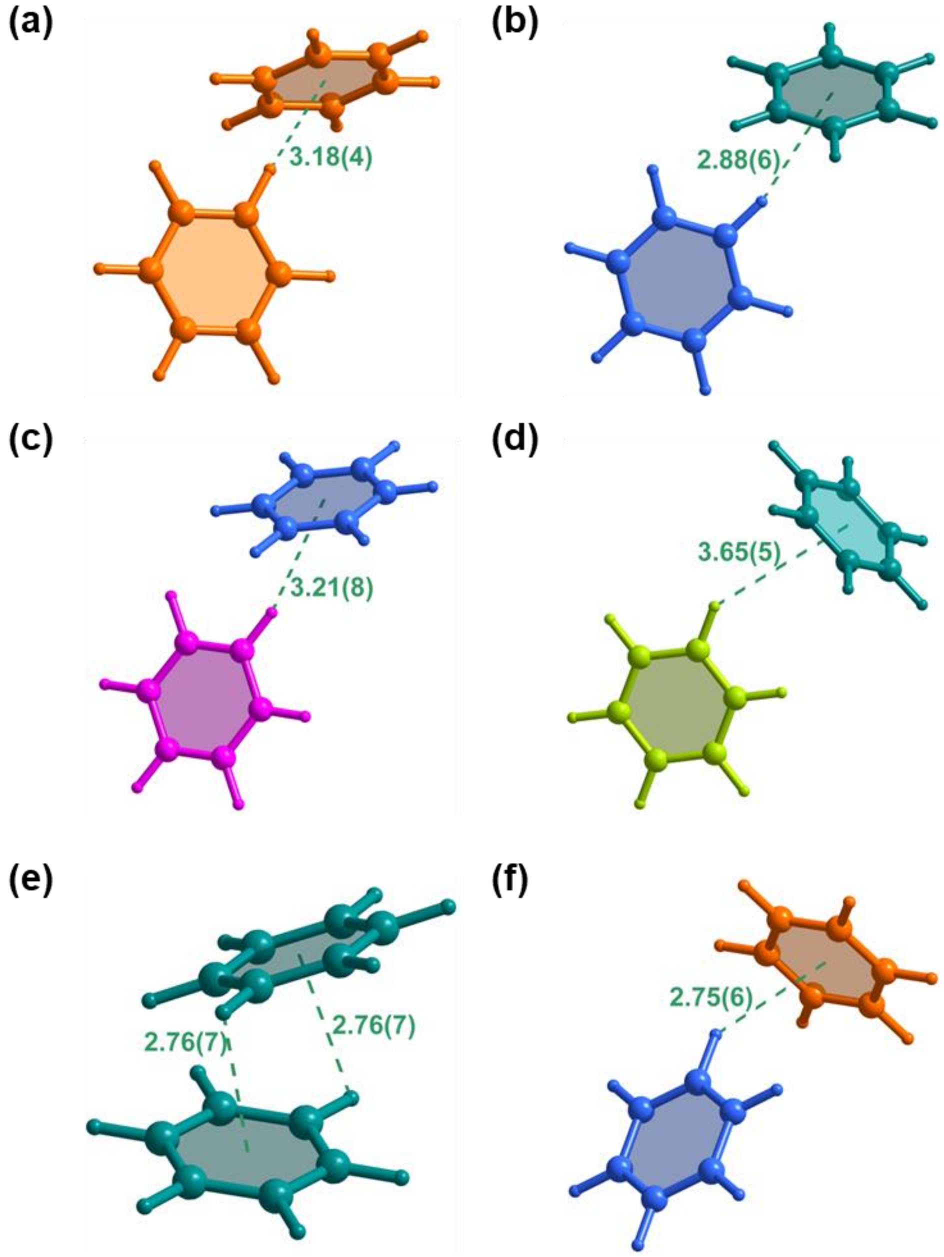

Figure S29. The structure of MIL-125-Zn·10.59C_6D_6 (CCDC 2301734). Views of interactions between guest benzene molecules in MIL-125-Zn determined from NPD data. Benzene at sites I–V are colored in pink, green, orange, blue and teal, respectively. The C–D···π interactions are highlighted in sea green.

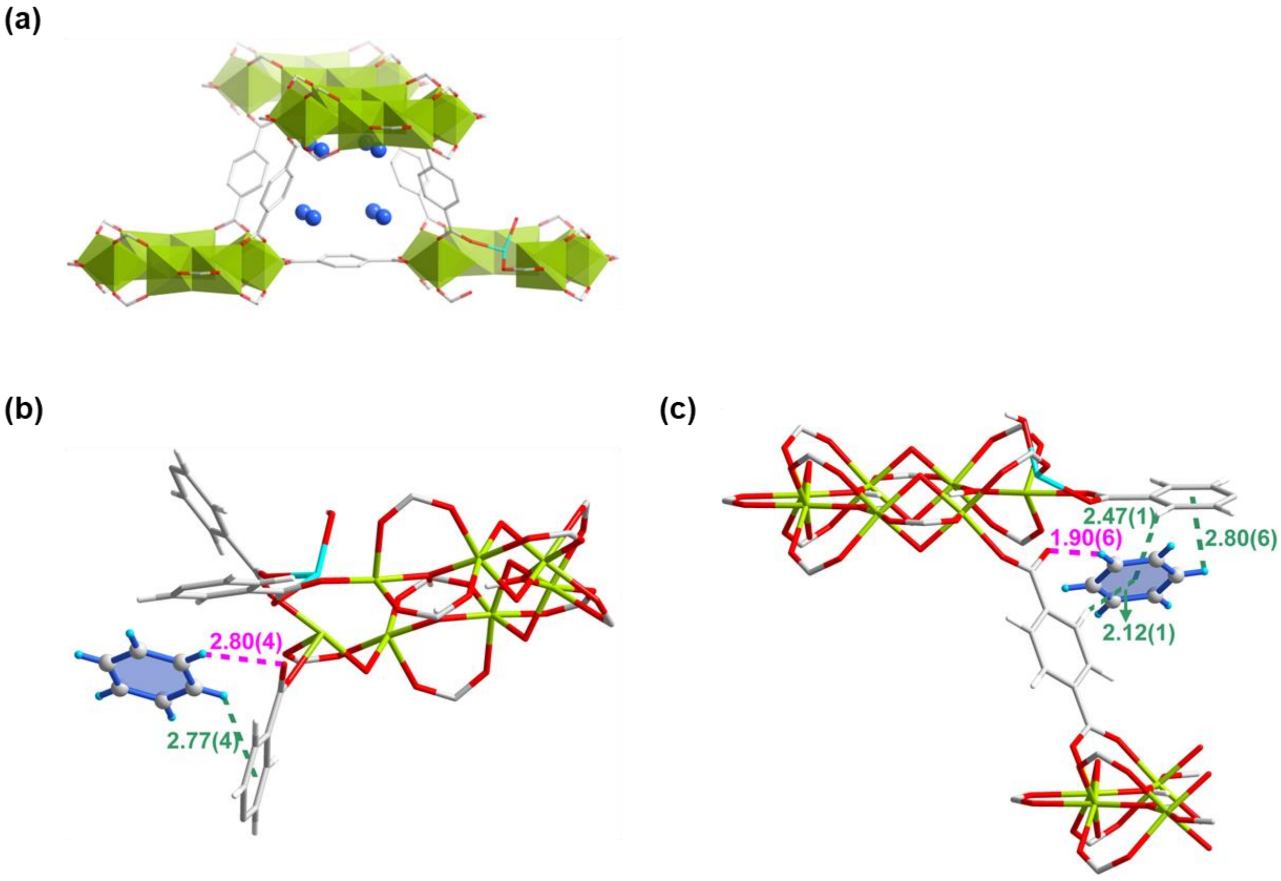

Figure S30. The structure of MIL-125-Zn·1.38C_6D_6 (CCDC 2301733). Views of the structural models of the adsorption of d_6-benzene in MIL-125-Zn·1.38C_6D_6 determined from NPD data. (a) Distribution of adsorbed d_6-benzene molecules within the tetrahedral cages in MIL-125-Zn·1.38C_6D_6. (b,c) Views of host-guest interactions with benzene at site III (b) and IV (c) in MIL-125-Zn. The C–$D_{benzene}\cdots O_{defect}$ interactions and C–H(or D)$\cdots\pi$ interactions are highlighted in pink and sea green, respectively. Color code for atoms: Ti, lime; Zn, turquoise; O, red; C, gray; D, sky blue; H, white.

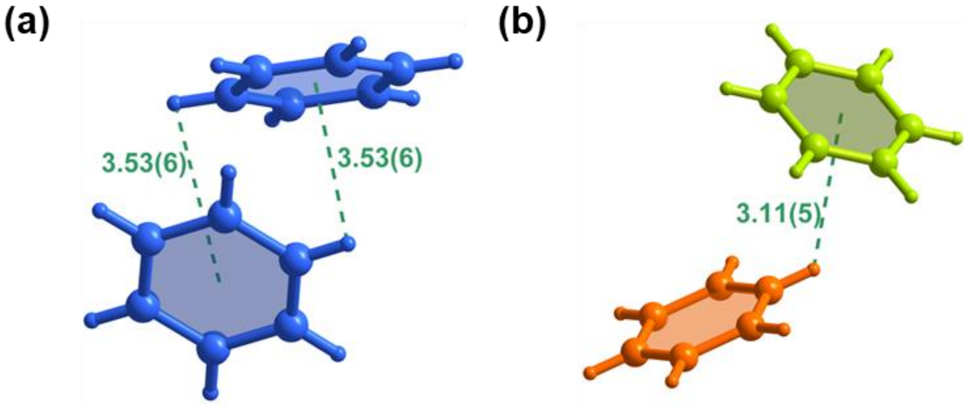

Figure S31. The structure of MIL-125-Zn·1.38C_6D_6 (CCDC 2301733). Views of interactions between guest benzene molecules in MIL-125-Zn·1.38C_6D_6 determined from NPD data. Benzene at site I–IV are colored in pink, green, orange and blue, respectively. The C–D···π interactions are highlighted in sea green.

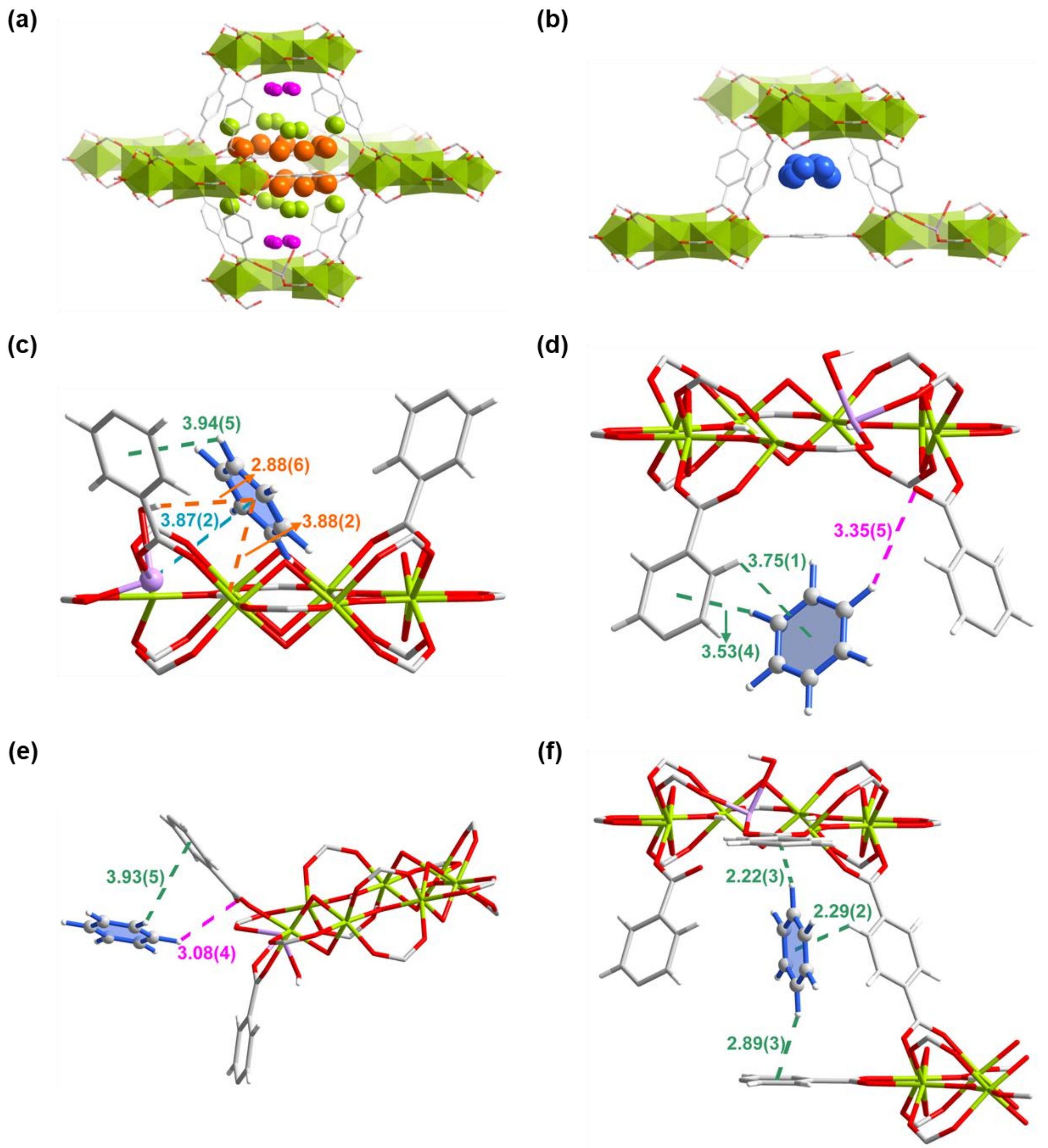

Figure S32. The structure of MIL-125-Mn·8.93C_6H_6 (CCDC 2301739). Views of the structural models for the adsorption of benzene in MIL-125-Mn determined from SPXRD data. (a,b) Distribution of adsorbed benzene molecules within the (a) octahedral and (b) tetrahedral cages in MIL-125-Mn. Sites I–IV are colored in pink, green, orange and blue, respectively. The radii of the colored balls of different binding sites are proportional to their crystallographic occupancies (Sites I–IV have occupancies of 1.34, 2.22, 2.66 and 2.70 for benzene per {Ti_7Mn} cluster in MIL-125-Mn, respectively). (c-f) Views of host-guest interactions with benzene at site I (c), II (d), III (e) and IV (f) in MIL-125-Mn. The $Mn^{II}\cdots\pi_{benzene}$ interactions, C–$\underline{H}_{benzene}\cdots O_{defect}$ interactions, O–$\underline{H}_{framework}\cdots\pi_{benzene}$ interactions and C–$\underline{H}\cdots\pi$ interactions are highlighted in blue, pink, orange and sea green, respectively. Color code for atoms: Ti, lime; Mn, lavender; O, red; C, gray; H, white.

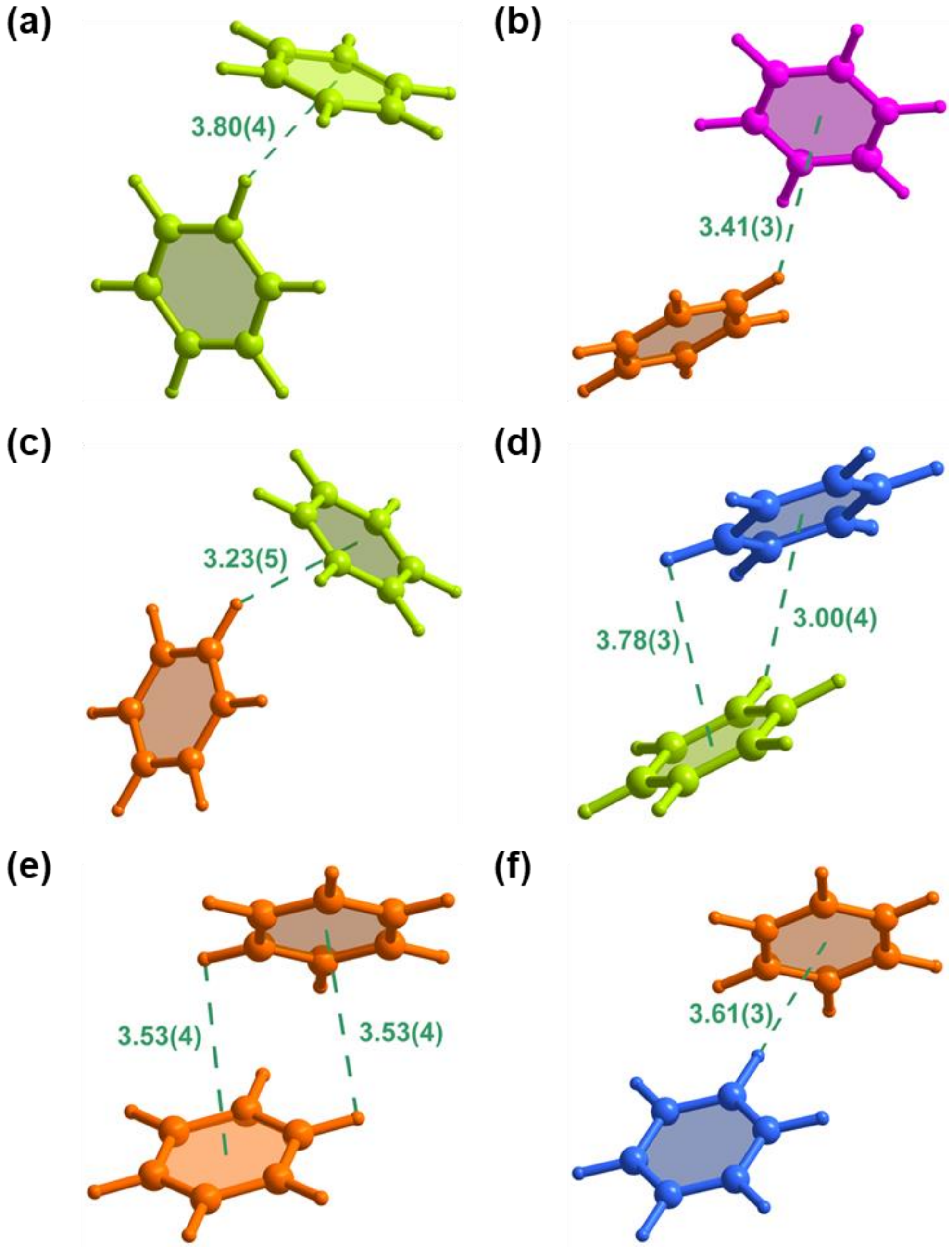

Figure S33. The structure of MIL-125-Mn·8.93C_6H_6 (CCDC 2301739). Views of interactions between guest benzene molecules in MIL-125-Mn determined from SPXRD data. Benzene at sites I–IV are colored in pink, green, orange and blue, respectively. The C–$\underline{H}\cdots\pi$ interactions are highlighted in sea green.

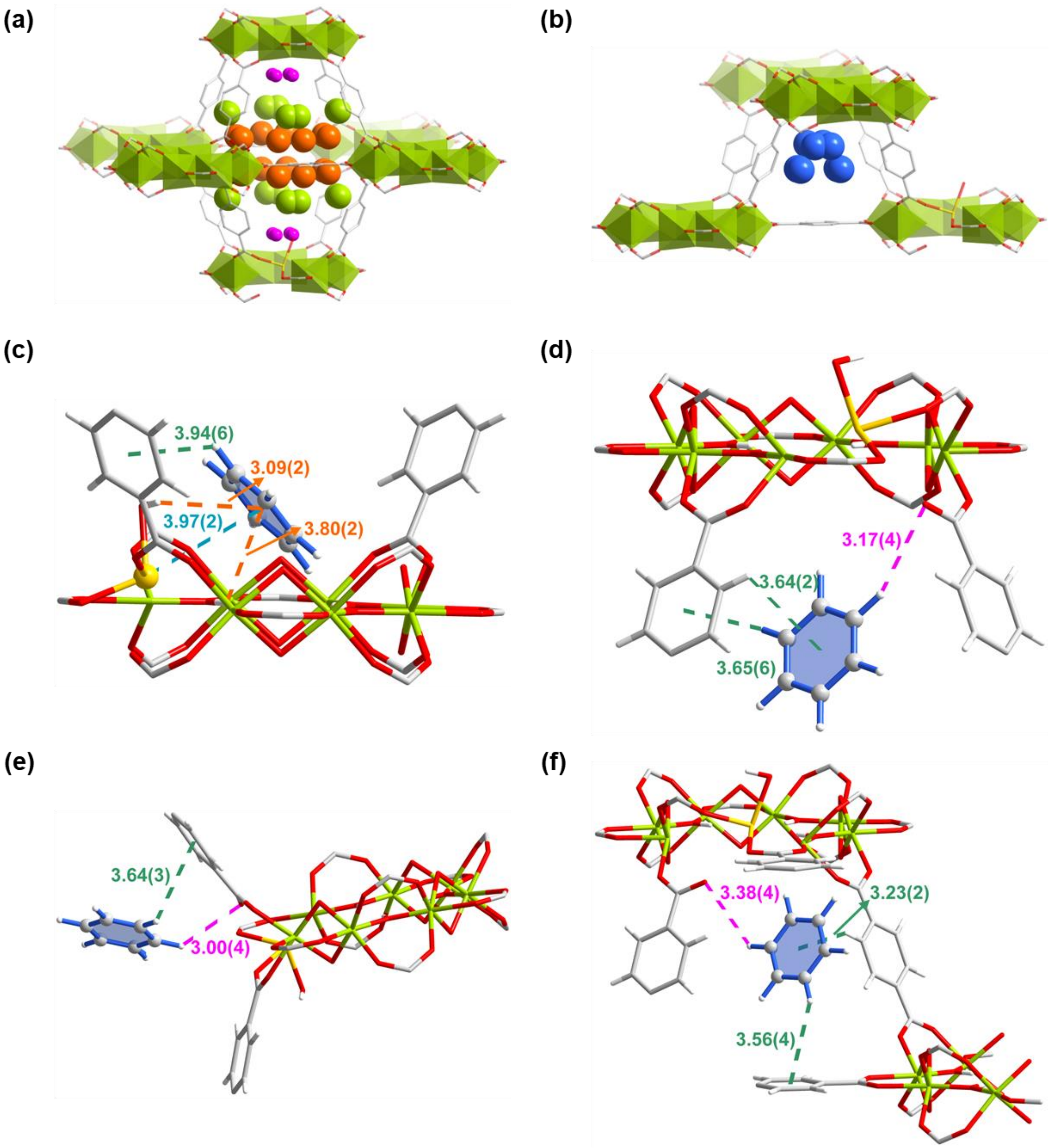

Figure S34. The structure of MIL-125-Fe·10.06C_6H_6 (CCDC 2301738). Views of the structural models for the adsorption of benzene in MIL-125-Fe determined from SPXRD data. (a,b) Distribution of adsorbed benzene molecules within the (a) octahedral and (b) tetrahedral cages in MIL-125-Fe. Sites I–IV are colored in pink, green, orange and blue, respectively. The radii of the colored balls of different binding sites are proportional to their crystallographic occupancies (Sites I–IV have occupancies of 1.28, 2.96, 2.91 and 2.91 for benzene per {Ti_7Fe} cluster in MIL-125-Fe, respectively). (c-f) Views of host-guest interactions with benzene at site I (c), II (d), III (e) and IV (f) in MIL-125-Fe. The $Fe^{II}\cdots\pi_{benzene}$ interactions, C–$\underline{H}_{benzene}\cdots O_{defect}$ interactions, O–$\underline{H}_{framework}\cdots\pi_{benzene}$ interactions and C–$\underline{H}\cdots\pi$ interactions are highlighted in blue, pink, orange and sea green, respectively. Color code for atoms: Ti, lime; Fe, gold; O, red; C, gray; H, white.

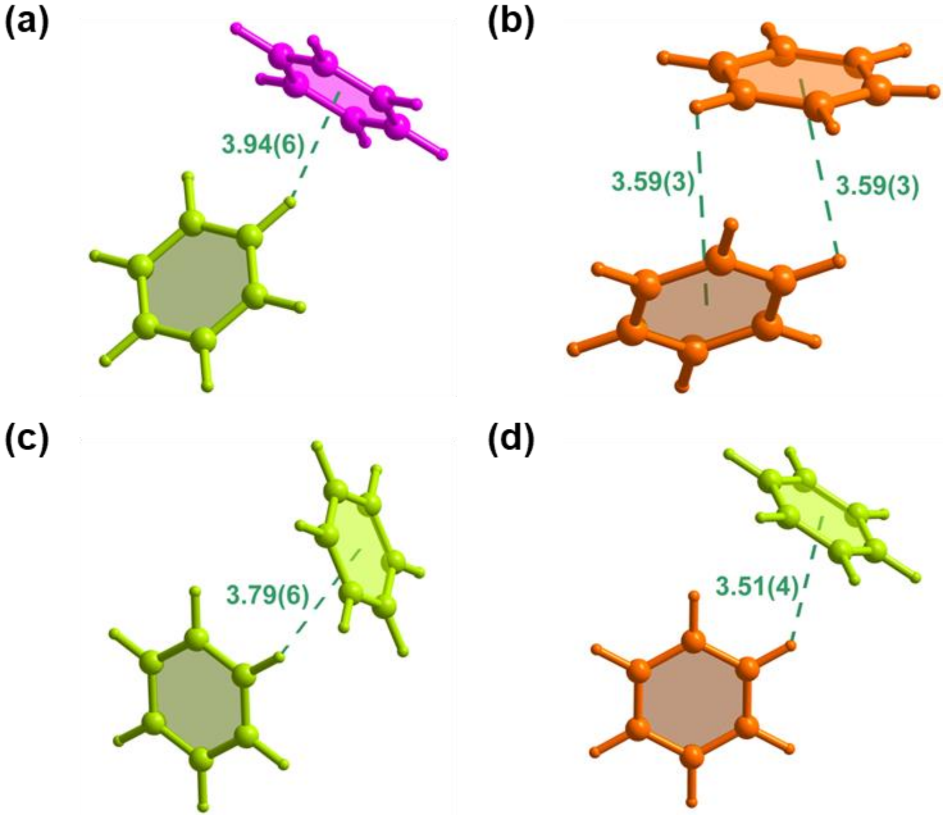

Figure S35. The structure of MIL-125-Fe·10.06C_6H_6 (CCDC 2301738). Views of interactions between guest benzene molecules in MIL-125-Fe determined from SPXRD data. Benzene at site I–IV are colored in pink, green, orange and blue, respectively. The C–H···π interactions are highlighted in sea green.

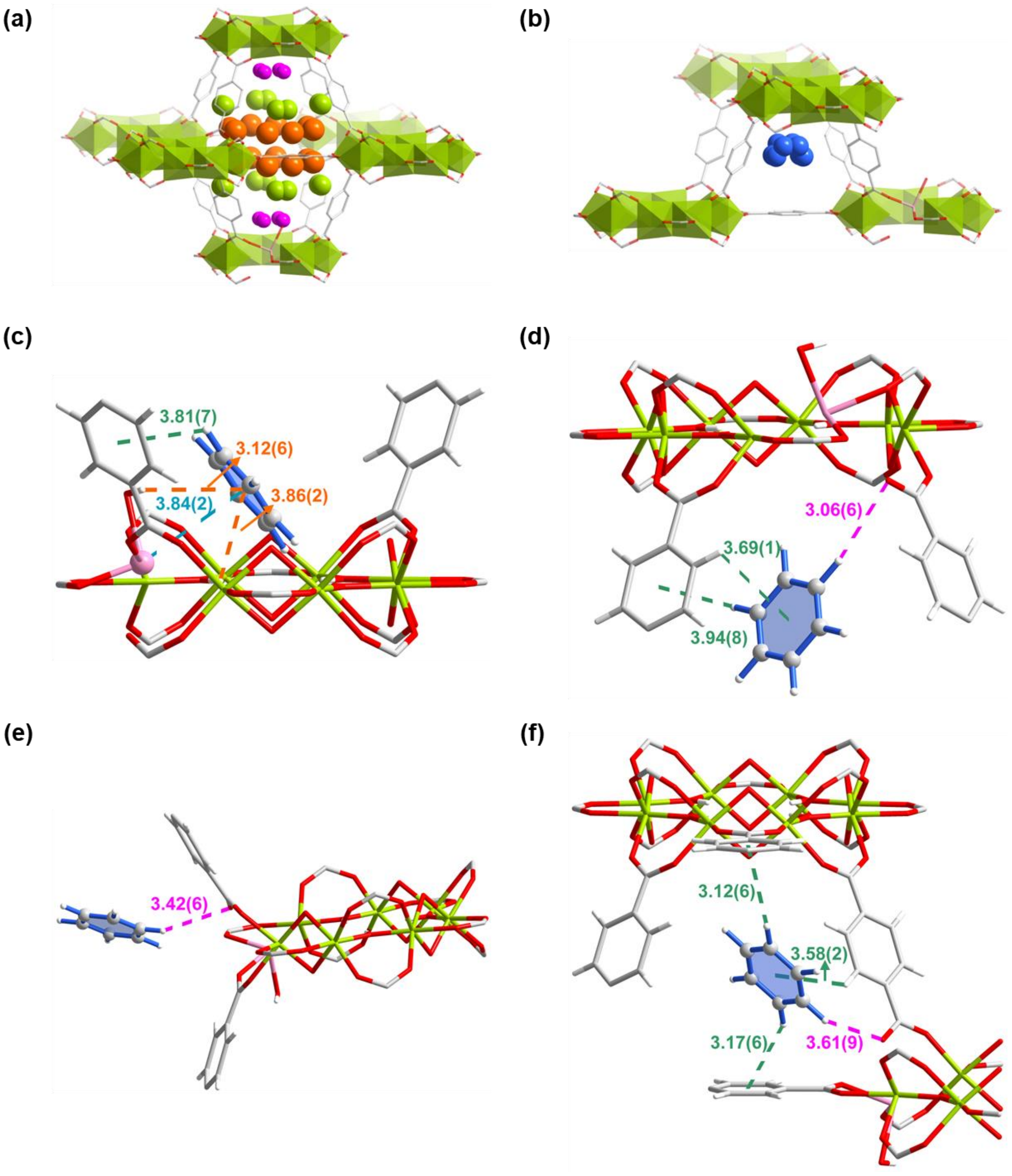

Figure S36. The structure of MIL-125-Co·8.91C_6H_6 (CCDC 2301740). Views of the structural models for the adsorption of benzene in MIL-125-Co determined from SPXRD data. (a,b) Distribution of adsorbed benzene molecules within the (a) octahedral and (b) tetrahedral cages in MIL-125-Co. Sites I–IV are colored in pink, green, orange and blue, respectively. The radii of the colored balls of different binding sites are proportional to their crystallographic occupancies (Sites I–IV have occupancies of 1.62, 2.50, 2.72 and 2.08 for benzene per {Ti_7Co} cluster in MIL-125-Co, respectively). (c-f) Views of host-guest interactions with benzene at site I (c), II (d), III (e) and IV (f) in MIL-125-Co. The $Co^{II}\cdots\pi_{benzene}$ interactions, C–$\underline{H}_{benzene}\cdots O_{defect}$ interactions, O–$\underline{H}_{framework}\cdots\pi_{benzene}$ interactions and C–$\underline{H}\cdots\pi$ interactions are highlighted in blue, pink, orange and sea green, respectively. Color code for atoms: Ti, lime; Co, rose; O, red; C, gray; H, white.

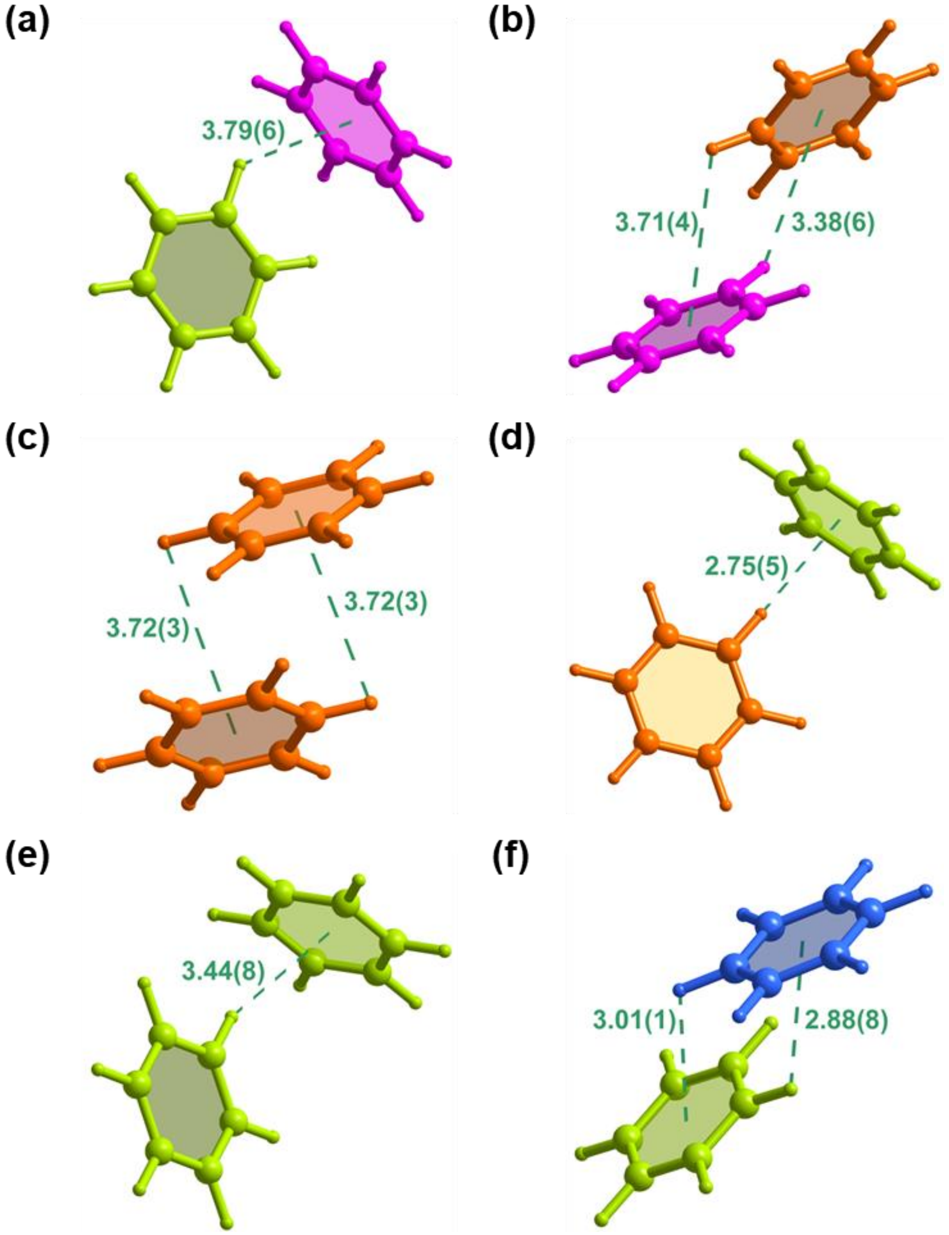

Figure S37. The structure of MIL-125-Co·8.91C_6H_6 (CCDC 2301740). Views of interactions between guest benzene molecules in MIL-125-Co determined from SPXRD data. Benzene at sites I–IV are colored in pink, green, orange and blue, respectively. The C–H···*π* interactions are highlighted in sea green.

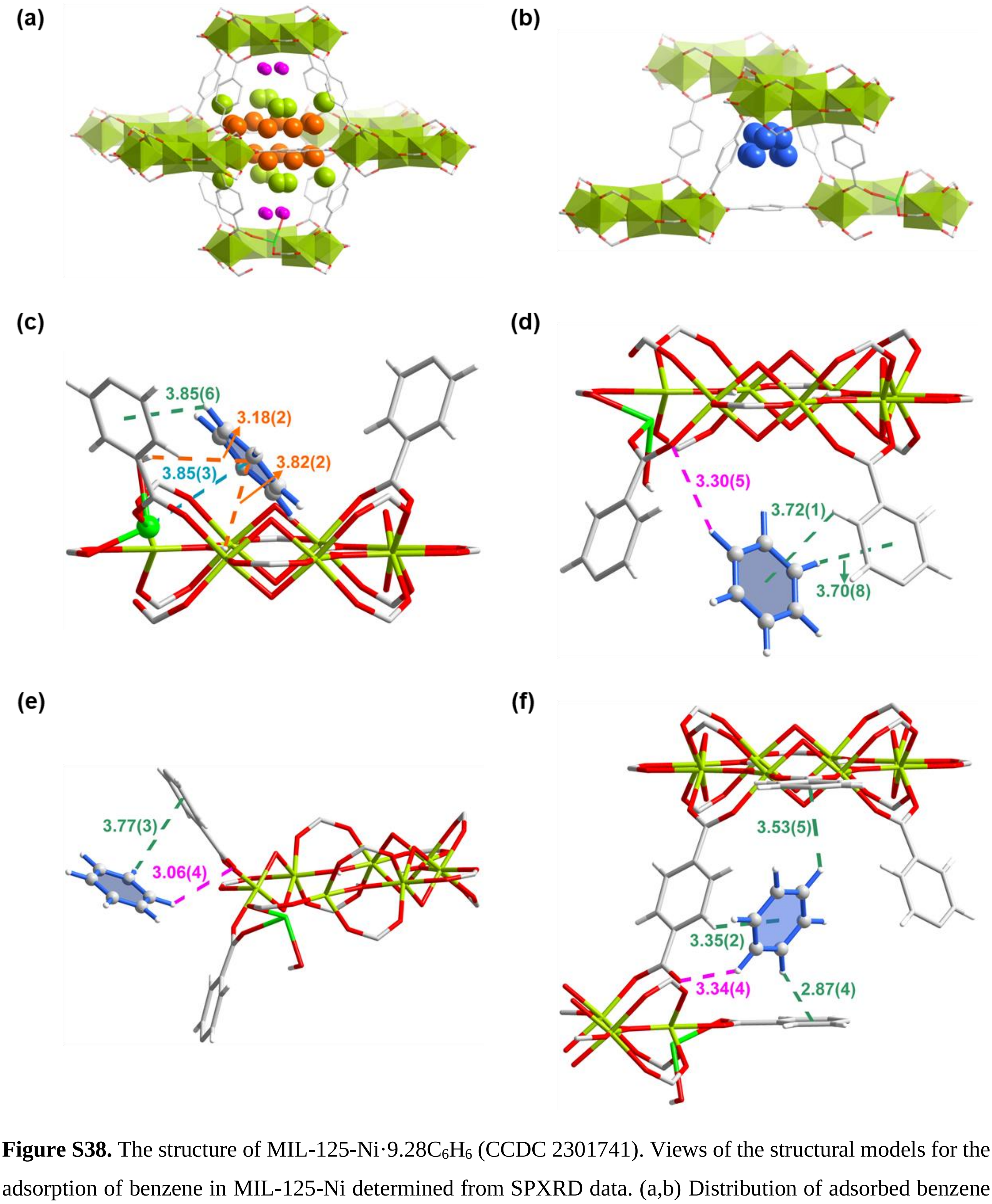

Figure S38. The structure of MIL-125-Ni·9.28C_6H_6 (CCDC 2301741). Views of the structural models for the adsorption of benzene in MIL-125-Ni determined from SPXRD data. (a,b) Distribution of adsorbed benzene molecules within the (a) octahedral and (b) tetrahedral cages in MIL-125-Ni. Sites I–IV are colored in pink, green, orange and blue, respectively. The radii of the colored balls of different binding sites are proportional to their crystallographic occupancies (Sites I–IV have occupancies of 1.36, 2.61, 2.64 and 2.67 for benzene per {Ti_7Ni} cluster in MIL-125-Ni, respectively). (c-f) Views of host-guest interactions with benzene at site I (c), II (d), III (e) and IV (f) in MIL-125-Ni. The $Ni^{II}\cdots\pi_{benzene}$ interactions, C–$\underline{H}_{benzene}\cdots O_{defect}$ interactions, O–$\underline{H}_{framework}\cdots\pi_{benzene}$ interactions and C–$\underline{H}\cdots\pi$ interactions are highlighted in blue, pink, orange and sea green, respectively. Color code for atoms: Ti, lime; Ni, bright green; O, red; C, gray; H, white.

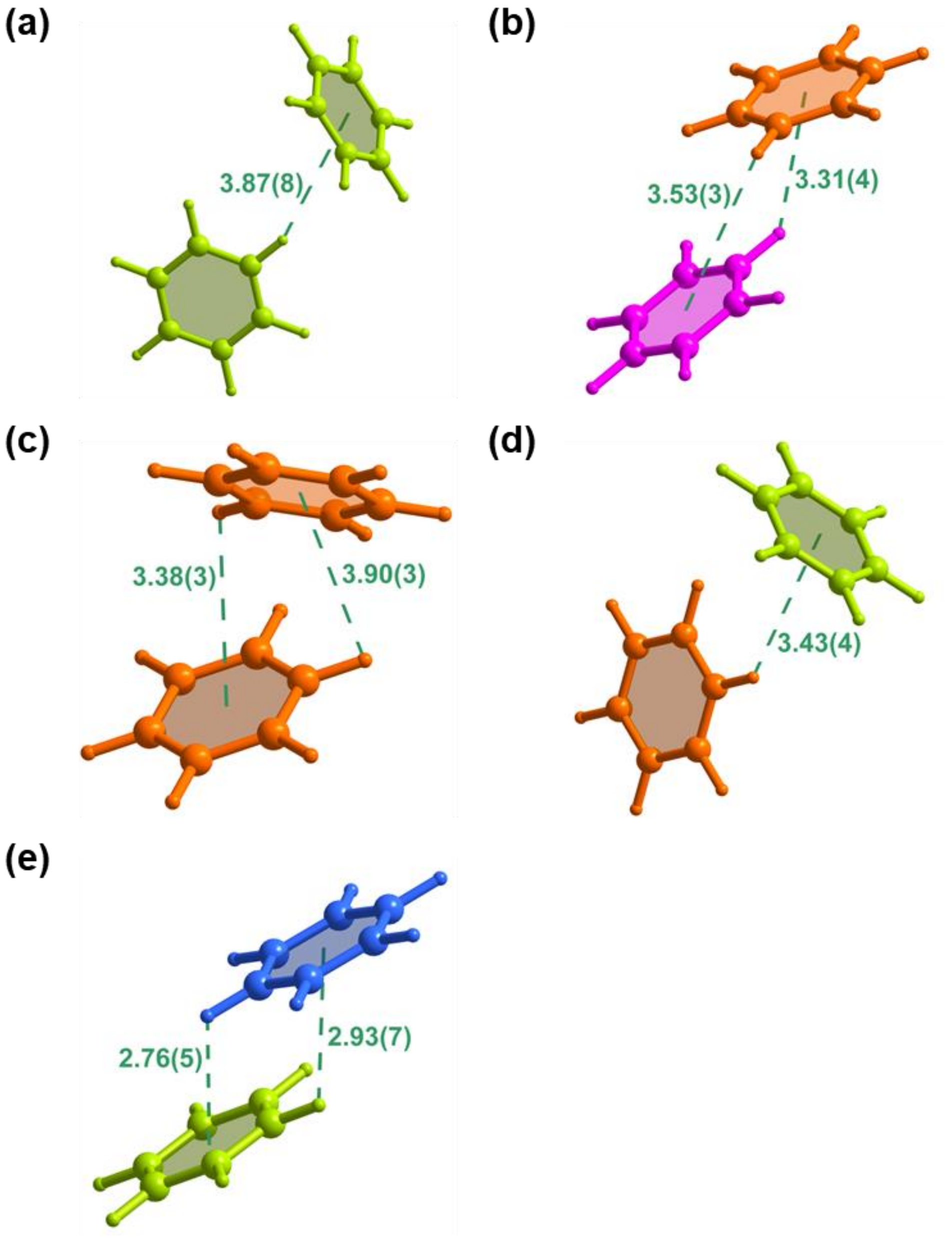

Figure S39. The structure of MIL-125-Ni·9.28C_6H_6 (CCDC 2301741). Views of interactions between guest benzene molecules in MIL-125-Ni determined from SPXRD data. Benzene at sites I–IV are colored in pink, green, orange and blue, respectively. The C–H···π interactions are highlighted in sea green.

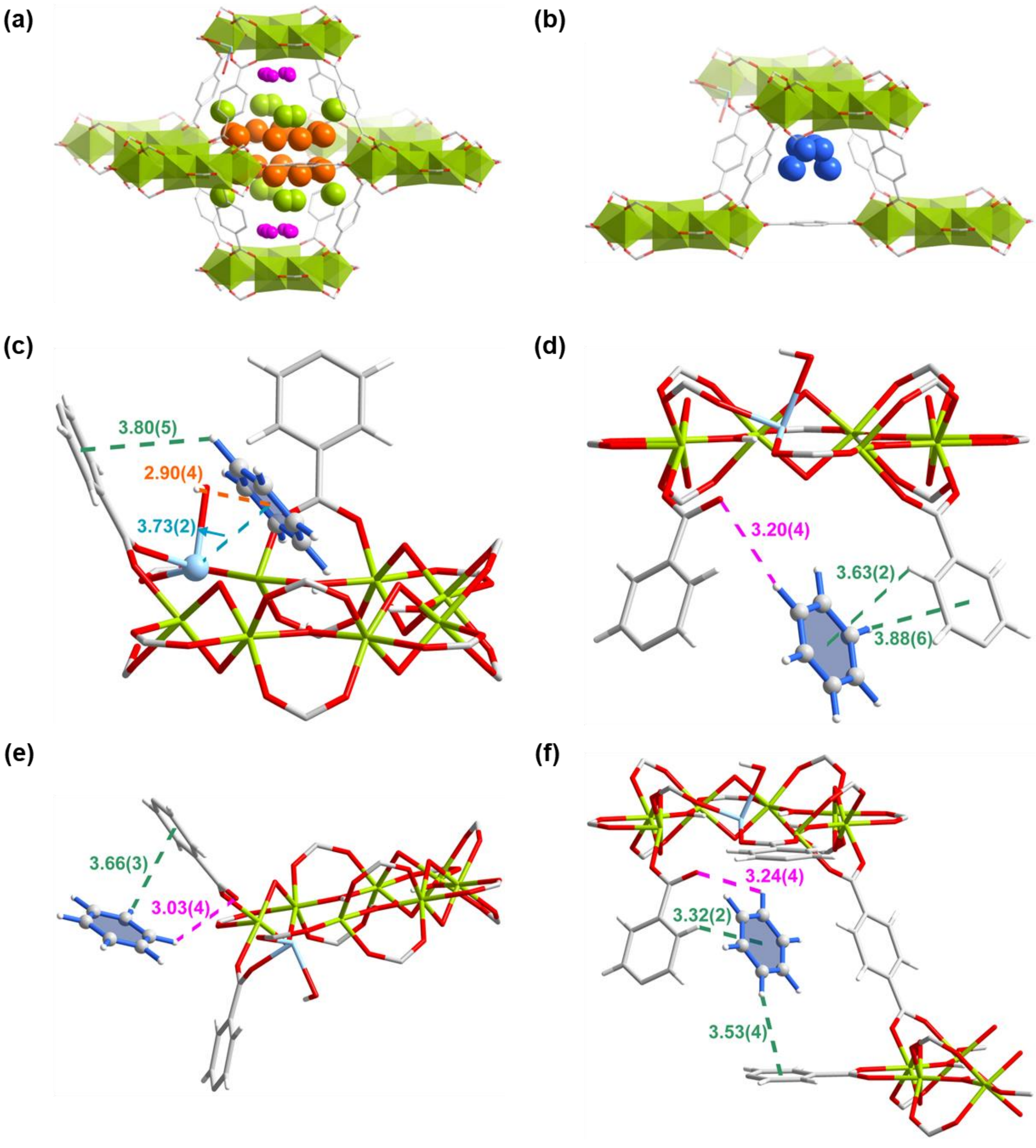

Figure S40. The structure of MIL-125-Cu·9.71C_6H_6 (CCDC 2301742). Views of the structural models for the adsorption of benzene in MIL-125-Cu determined from SPXRD data. (a,b) Distribution of adsorbed benzene molecules within the (a) octahedral and (b) tetrahedral cages in MIL-125-Cu. Sites I–IV are colored in pink, green, orange and blue, respectively. The radii of the colored balls of different binding sites are proportional to their crystallographic occupancies (Sites I–IV have occupancies of 1.42, 2.78, 2.90 and 2.61 for benzene per {Ti_7Cu} cluster in MIL-125-Cu, respectively). (c-f) Views of host-guest interactions with benzene at site I (c), II (d), III (e) and IV (f) in MIL-125-Cu. The $Cu^{II}\cdots\pi_{benzene}$ interactions, C–$\underline{H}_{benzene}\cdots O_{defect}$ interactions, O–$\underline{H}_{framework}\cdots\pi_{benzene}$ interactions and C–$\underline{H}\cdots\pi$ interactions are highlighted in blue, pink, orange and sea green, respectively. Color code for atoms: Ti, lime; Cu, pale blue; O, red; C, gray; H, white.

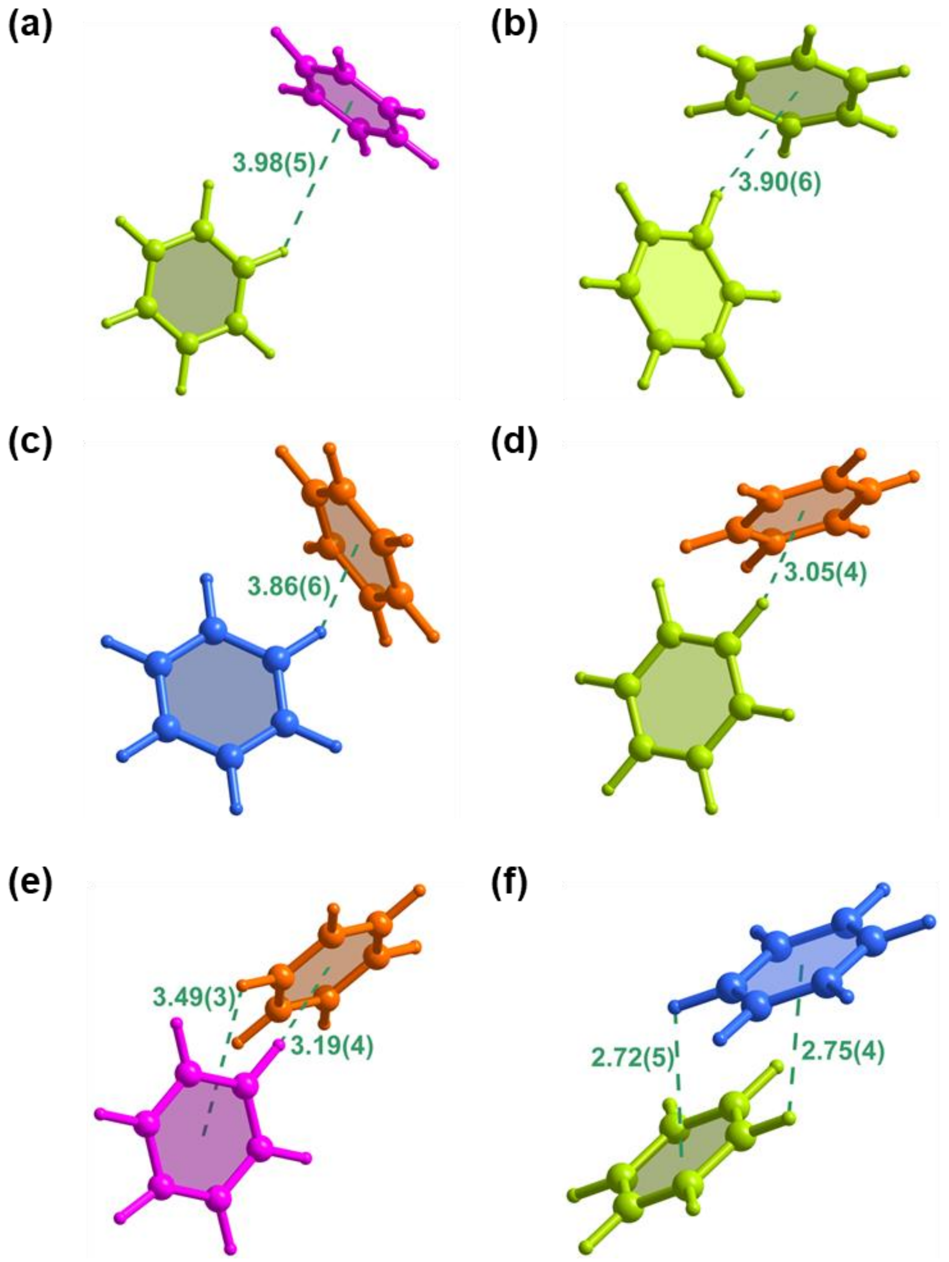

Figure S41. The structure of MIL-125-Cu·9.71C_6H_6 (CCDC 2301742). Views of interactions between guest benzene molecules in MIL-125-Cu determined from SPXRD data. Benzene at sites I–IV are colored in pink, green, orange and blue, respectively. The C–H···π interactions are highlighted in sea green.

11. *In situ* FTIR Spectra

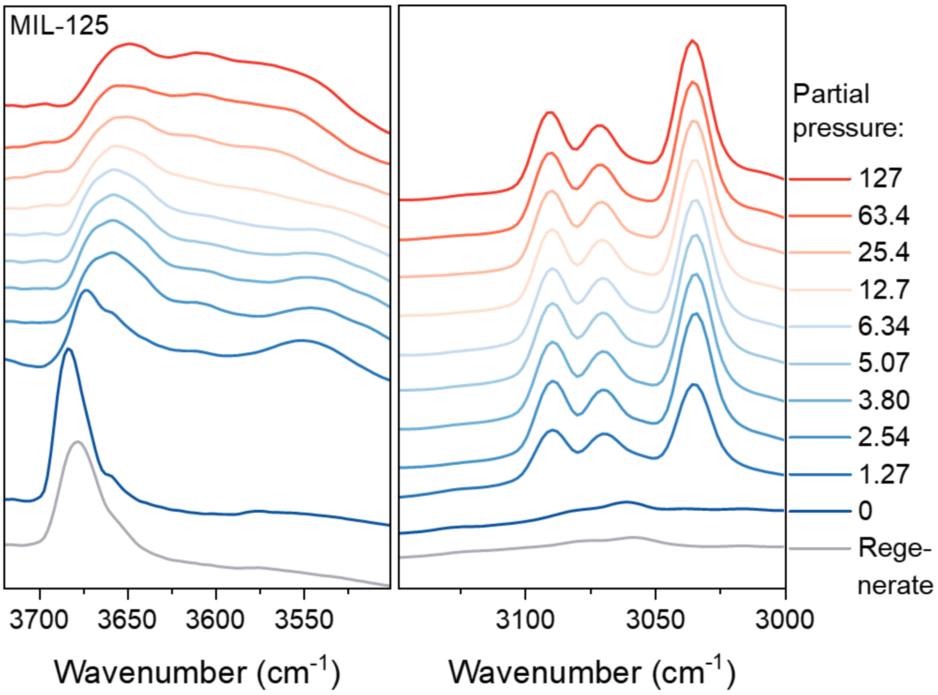

Figure S42. *In situ* FTIR spectra of MIL-125 at partial pressures of benzene from 0–127 mbar (diluted in dry N_2) and after regeneration at 353 K with dry N_2 flow. Wavenumber of 3720–3500 cm^{-1} and 3150–3000 cm^{-1} corresponds to the v(OH) and CH stretching region, respectively.[11] The FTIR spectra of MIL-125-defect and MIL-125-Zn (Fig. 4a, 4b in the main text) exhibit two distinct O–H stretching bands (3687 and 3674 cm^{-1}), indicating the presence of two types of hydroxyl groups with one originating from defects. In contrast, MIL-125 shows only a single O–H stretching band, corresponding to a single type of hydroxyl group. Therefore, the FTIR results directly corroborate our Rietveld Refinement analysis.

12. DFT-simulated INS Spectra

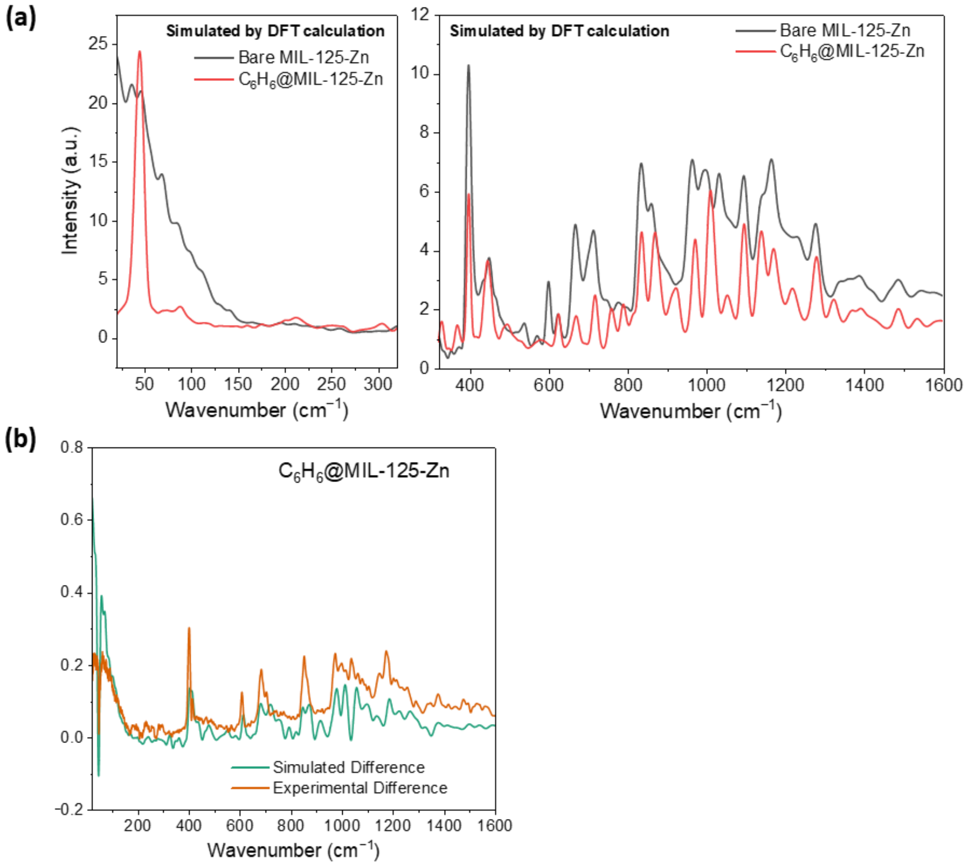

Figure S43. (a) DFT-simulated INS spectra for bare and C_6H_6-loaded MIL-125-Zn. (b) Comparison of the difference plots for experimental and DFT-calculated INS spectra of bare and C_6H_6-loaded MIL-125-Zn.

13. ssNMR spectra

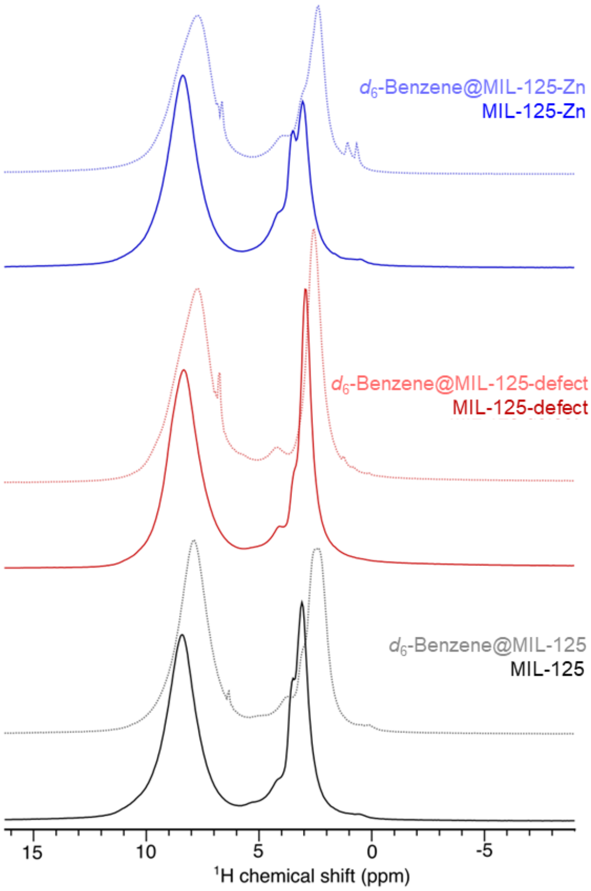

Figure S44. ^{1}H Hahn-echo MAS NMR spectra of MIL-125 (bottom, black), MIL-125-defect (middle, red), and MIL-125-Zn (top, blue), and d_6-benzene-loaded counterparts (light-colored lines). All spectra were recorded at 9.4 T using a MAS frequency of 12 kHz.

14. QENS analysis

QENS data was collected at the IRIS spectrometer at the ISIS facility at Rutherford Appleton Laboratory (UK). The experimental data consisted of a series of backgrounds (resolution function $R(\mathrm{Q},\omega)$) at each temperature for desolvated MIL-125 and MIL-125-Zn. A total of five temperatures were measured, 27, 77, 127,177 and 227 °C (300, 350, 400, 450, 500 K). The data $I(\mathrm{Q},\omega)$ contains contribution from the 'MOF + benzene' at the same temperatures. The data were fitted using the Qclimax module (ICE-MAN)[12], and a global minimisation was obtained for the following equation:

$$I(\mathrm{Q},\omega) = \left[f_n(\mathrm{Q})\frac{1}{\pi}\frac{\Gamma_n(\mathrm{Q})}{\Gamma_n(\mathrm{Q})^2+\omega^2} + f_b(\mathrm{Q})\frac{1}{\pi}\frac{\Gamma_b(\mathrm{Q})}{\Gamma_b(\mathrm{Q})^2+\omega^2} + f_\delta(\mathrm{Q})\delta\right] \otimes R(\mathrm{Q},\omega) + C_1(\mathrm{Q})\omega + C_1$$

The variables, $\Gamma_n(\mathrm{Q})$ and $\Gamma_b(\mathrm{Q})$ are the widths of the Lorentzian curves: these were set to a constant value independent of Q; the n and b referred to a narrow and a broad component, respectively; the δ referred to contribution of elastic scattering. The center of all fitting functions is the same at each Q. The equation then became:

$$I(\mathrm{Q},\omega) = \left[f_n(\mathrm{Q})\frac{1}{\pi}\frac{\Gamma_n}{{\Gamma_n}^2+\omega^2} + f_b(\mathrm{Q})\frac{1}{\pi}\frac{\Gamma_b}{{\Gamma_b}^2+\omega^2} + f_\delta(\mathrm{Q})\delta\right] \otimes R(\mathrm{Q},\omega) + linear\ background$$

A representative overall fit is shown in Fig. S45, and the amplitudes for each component were fitted against Q at all five temperature points (Fig. S46).

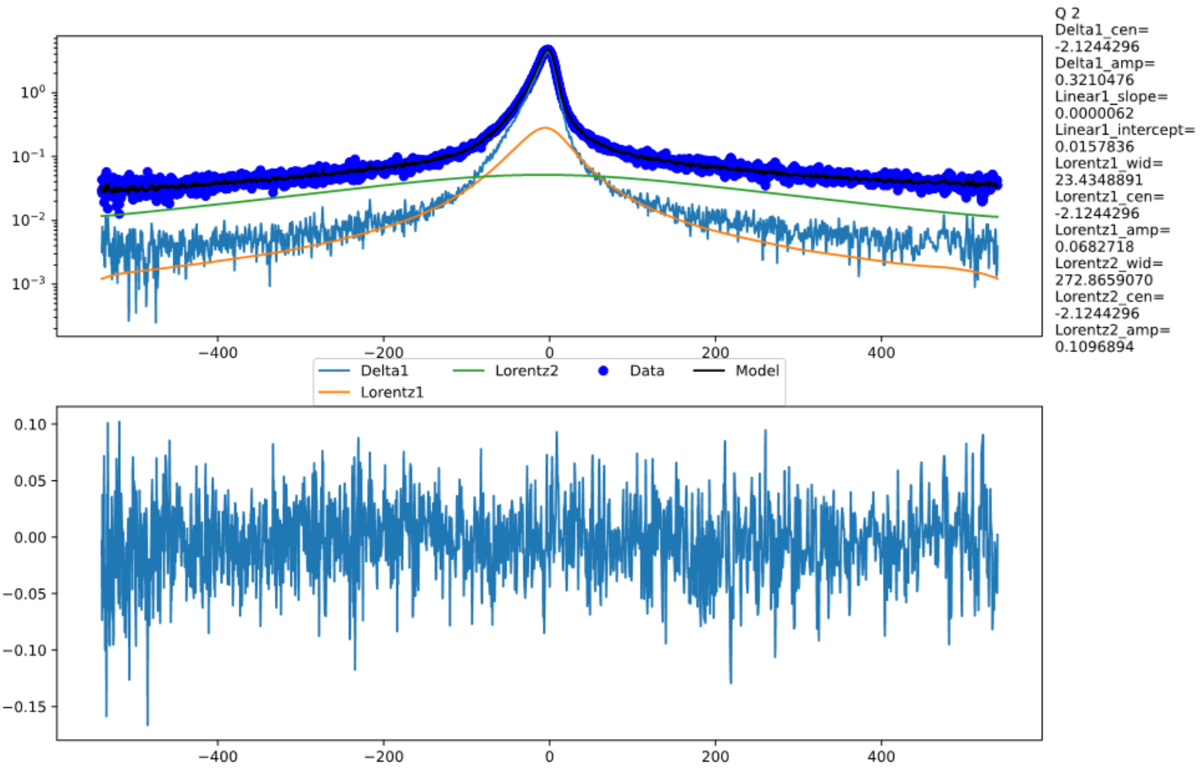

Figure S45. A representative overall fit of the QENS spectra. Only raw data points and fitted curves from different components of the model are shown.

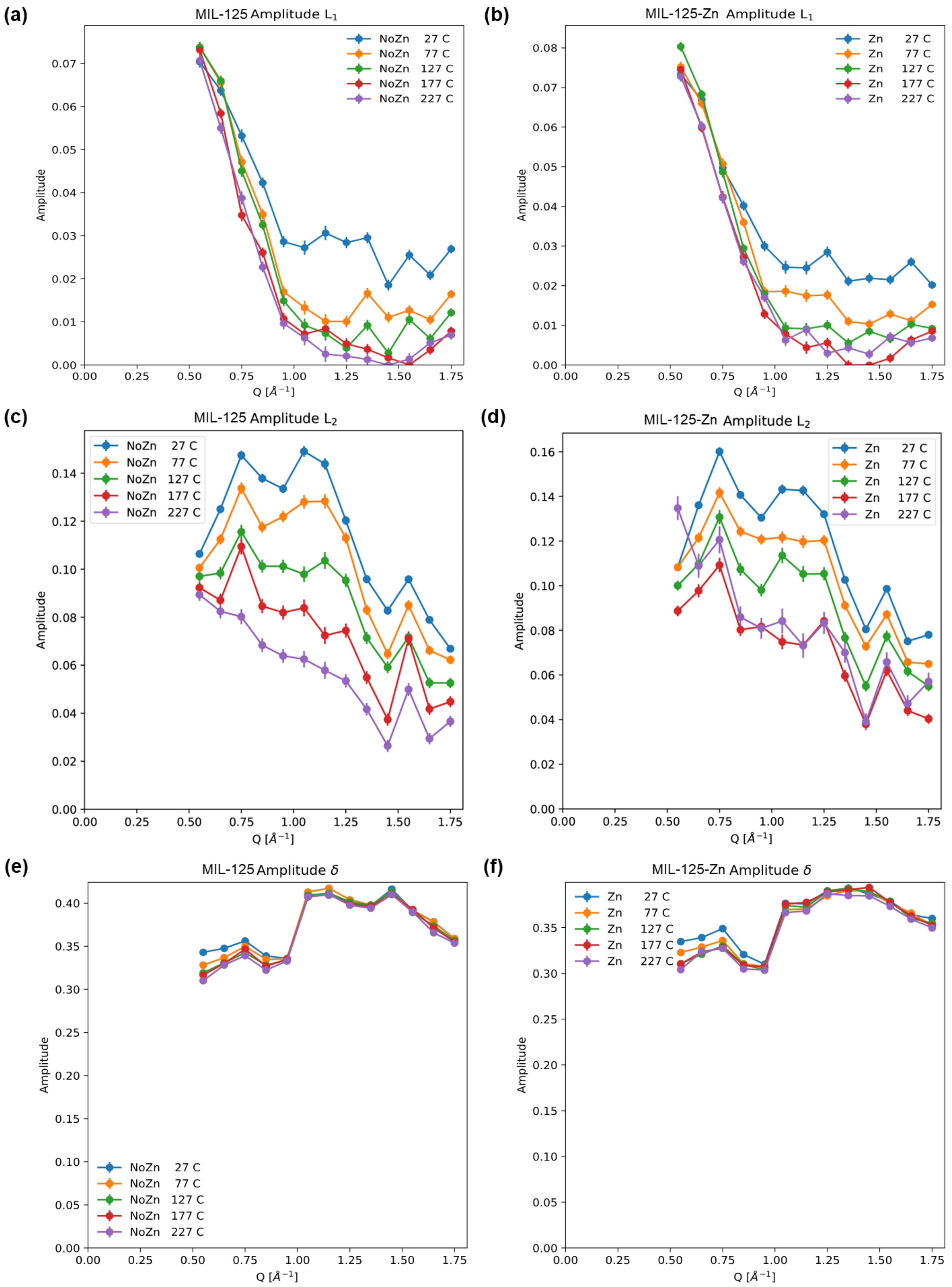

Figure S46. The fitted amplitudes for the (a, b) narrow components, (c, d) broad components and (e, f) delta components for MIL-125 and MIL-125-Zn. The different Q dependence of the Lorentzian amplitudes points to different motions, for example, one could be a tumbling while the other could be a rotation around an axis. Data points are shown as mean values with error bars indicating standard deviation for the fitting against Q at temperatures of 27, 77, 127, 177, 227 °C.

15. Additional discussion

15.1. Q_{st} and toluene/cyclohexane adsorption

The isosteric heats of adsorption (Q_{st}) for benzene uptake in MIL-125-Zn are 58−62 kJ mol^{-1} at surface coverage up to 5.8 mmol g^{-1}, indicating strong host-guest interactions, consistent with the sharp adsorption at low pressure. These are further increased to 69 kJ mol^{-1} at 7.4 mmol g^{-1} owing to additional guest-guest interactions (Fig. S10). Similar trends are observed for MIL-125-defect and other MIL-125-X materials, except for MIL-125-Fe, which shows lower Q_{st} (47−48 kJ mol^{-1}) at surface coverage up to 5.1 mmol g^{-1}, consistent with its slightly lower adsorption. Adsorption isotherms of toluene and cyclohexane were also recorded at 298−318 K for MIL-125-Zn (Fig. S11). At 298 K and relative pressure P/P_0 = 0.01, MIL-125-Zn exhibits higher uptakes for toluene (3.92 mmol g^{-1}) and benzene (7.63 mmol g^{-1}) compared with cyclohexane (1.48 mmol g^{-1}), indicating favoured adsorption affinity to aromatic compounds, consistent with the higher Q_{st} for toluene and benzene (Fig. S12).

15.2. Binding domains for adsorbed benzene

MIL-125-X (X = Mn, Fe, Co, Ni, Cu) all present only four binding sites for benzene, sites I–III in octahedral cages and site IV in tetrahedral cages (Fig. S32-S41). Likewise, $X^{II}\cdots\pi_{benzene}$ interactions are observed at site I, but at longer distances [3.72(2)–3.97(2) Å] than that observed for MIL-125-Zn [2.43(9)–3.69(9) Å]. This weaker interaction between benzene and these metal centers compared with $Zn^{II}\cdots\pi_{benzene}$ is consistent with their lower uptake at low pressure. Apart from this, MIL-125-X (X = Mn, Fe, Co, Ni, Cu) all exhibit analogous host-guest interactions with benzene (Fig. S32-S41). In addition, C–$\underline{H}$(or $\underline{D}$)$_{benzene}\cdots\pi_{benzene}$ interactions are found between guest benzene molecules [Supplementary Section 10]. It is worth noting that the π···cation interactions observed in MIL-125-X play an important role in promoting its adsorption performance compared with other leading sorbents, such as BUT-55[4] and ZJU-520(Al)[7], for which the adsorbed benzene molecules are primarily stabilised by π···π interactions accompanied by hydrogen bonding (Table S2).

15.3. ssNMR

Magic angle spinning (MAS) NMR spectra were recorded for MIL-125, MIL-125-defect and MIL-125-Zn. The {^{1}H-}^{13}C cross-polarisation (CP) MAS NMR spectra (Fig. 4e), as well as the ^{1}H MAS NMR spectra (Fig. S44), show that the bare MOFs are all very similar in the structural organisation of their frameworks as they share the same resonances. Notably, along with the expected ^{13}C resonances for carboxylate (d{^{13}C} = 173 ppm) and aromatic (d{^{13}C} = 130 and 135 ppm) carbons, resonances from aliphatic carbons can be observed in all MOFs. These have been assigned to residual methanol and dimethylamine (DMA) species; the latter arise as a product of DMF degradation during MOF synthesis[13]. The presence of these species after sample activation suggests that they are framework-bound and that even the pristine MIL-125 contains defect sites.

16. References

1. Dan-Hardi, M. et al. A new photoactive crystalline highly porous titanium (IV) dicarboxylate. *J. Am. Chem. Soc.* **131**, 10857-10859 (2009).
2. Zhang, Y. et al. Single-atom Cu anchored catalysts for photocatalytic renewable H_2 production with a quantum efficiency of 56%. *Nat. Commun.* **13**, 58 (2022).
3. Xie, L. H., Liu, X. M., He, T. & Li, J. R. Metal-organic frameworks for the capture of trace aromatic volatile organic compounds. *Chem* **4**, 1911-1927 (2018).
4. He, T. et al. Trace removal of benzene vapour using double-walled metal-dipyrazolate frameworks. *Nat. Mater.* **21**, 689-695 (2022).
5. Han, Y. et al. Control of the pore chemistry in metal-organic frameworks for efficient adsorption of benzene and separation of benzene/cyclohexane. *Chem* **9**, 739-754 (2023).
6. Hu, L. et al. A novel aluminum-based metal-organic framework with uniform micropores for trace BTEX adsorption. *Angew. Chem., Int. Ed.* **62**, e202215296 (2023).
7. Hu, L. et al. Double-walled Al-based MOF with large microporous specific surface area for trace benzene adsorption. *Nat. Commun*. **15**, 1–8 (2024).
8. Lv, J.-A. *et al.* Interior and exterior surface modification of Zr-based metal–organic frameworks for trace benzene removal. *Inorg. Chem.* **63**, 4249–4259 (2024).
9. Tu, T. N. *et al.* Metal–organic frameworks for aromatic-based VOC capture. *Sep. Purif. Technol.* **333**, 125883 (2024).
10. Galli, S. *et al.* Adsorption of harmful organic vapors by flexible hydrophobic bis-pyrazolate based MOFs. *Chem. Mater.* **22**, 1664–1672 (2010).
11. Tripathi, A.K. et al. QENS and FTIR studies on binding states of benzene molecules adsorbed in zeolite HZSM-5 at room temperature. *Phys. Chem. Chem. Phys.* **3**, 4449-4455 (2001).
12. Ramirez-Cuesta, A., Smith, R., Mamontov, E. & Cheng, Y. ICE-MAN the integrated computational environment for modeling and analysis for neutrons at ORNL. *EPJ Web Conf.* **272**, 01013 (2022).
13. Fu, Y. et al. Solvent-derived defects suppress adsorption in MOF-74. *Nat. Commun.* **14**, 2386 (2023).
