# Supplementary material for: Trace benzene capture by decoration of structural defects in metal–organic framework materials
Source: Nat Mater. 2024 Oct 29;23(11):1531–8. doi: 10.1038/s41563-024-02029-1 (PMC11525167; doi:10.1038/s41563-024-02029-1)

checkCIF/PLATON report

You have not supplied any structure factors. As a result the full set of tests cannot be run.

THIS REPORT IS FOR GUIDANCE ONLY. IF USED AS PART OF A REVIEW PROCEDURE FOR PUBLICATION, IT SHOULD NOT REPLACE THE EXPERTISE OF AN EXPERIENCED CRYSTALLOGRAPHIC REFEREE.

No syntax errors found. CIF dictionary Interpreting this report

Datablock: C6D6@MIL-125-defect

Bond precision: C-C = 0.0140 A Wavelength=0

Cell:	a=18.5706(11)	b=18.5706(11)	c=18.1366(11)
	alpha=90	beta=90	gamma=90
Temperature:	10 K		

	Calculated	Reported
Volume	6254.7(8)	6254.7(8)
Space group	I 4/m m m	I4/mmm
Hall group	-I 4 2	-I 4 2
Moiety formula	8(C6 H3 O4.31 Ti0.87), 0.079(C96 D96), 0.45(C48 D48), C11, 3.33	8(C6 H3.22 D0.22 O4.31 Ti0.87), 6.573(C6 D6), 0.286(C6 H6)
Sum formula	C88.16 H27.34 D40.30 O34.46 Ti6.98	C89.16 D41.16 H27.48 O34.46 Ti6.98
Mr	2052.79	2066.87
Dx,g cm-3	1.090	1.097
Z	2	2
Mu (mm-1)	0.000	0.000
F000	1858.8	0.0
F000'	2051.41	
h,k,lmax		
Nref		
Tmin,Tmax		
Tmin'		

Correction method= Not given

Data completeness= Theta(max)=

R(reflections)= wR2(reflections)=

S = Npar=

The following ALERTS were generated. Each ALERT has the format
test-name_ALERT_alert-type_alert-level.
Click on the hyperlinks for more details of the test.

Alert level A

PLAT770_ALERT_2_A Suspect C-H Bond in CIF: C_3 --H_3_1_H . 1.47 Ang.

Author Response: The close contact of this alert could be due to the disordered guest molecules within the framework.

PLAT770_ALERT_2_A Suspect C-H Bond in CIF: C_3 --H_1_1_H . 1.52 Ang.

Author Response: The close contact of this alert could be due to the disordered guest molecules within the framework.

PLAT770_ALERT_2_A Suspect C-H Bond in CIF: C_3 --H_3_1_H . 1.57 Ang.

Author Response: The close contact of this alert could be due to the disordered guest molecules within the framework.

PLAT770_ALERT_2_A Suspect C-H Bond in CIF: C_4_1 --H_1_1_H . 1.48 Ang.

Author Response: The close contact of this alert could be due to the disordered guest molecules within the framework.

PLAT770_ALERT_2_A Suspect C-H Bond in CIF: C_5_1 --H_5_1_H . 1.36 Ang.

Author Response: The close contact of this alert could be due to the disordered guest molecules within the framework.

PLAT770_ALERT_2_A Suspect C-H Bond in CIF: C_5_1 --H_1_1_H . 1.45 Ang.

Author Response: The close contact of this alert could be due to the disordered guest molecules within the framework.

PLAT770_ALERT_2_A Suspect C-H Bond in CIF: C_6_1 --H_6_1_H . 1.51 Ang.

Author Response: The close contact of this alert could be due to the disordered guest molecules within the framework.

PLAT770_ALERT_2_A Suspect C-H Bond in CIF: C_6_1 --H_1_1_H . 1.52 Ang.

Author Response: The close contact of this alert could be due to the disordered guest molecules within the framework.

```
PLAT770_ALERT_2_A Suspect C-H Bond in CIF:  C_6_1    --H_6_1_H   .       1.55 Ang.
```

Author Response: The close contact of this alert could be due to the disordered guest molecules within the framework.

```
PLAT770_ALERT_2_A Suspect C-H Bond in CIF:  C_6_1    --H_6_1_H   .       1.58 Ang.
```

Author Response: The close contact of this alert could be due to the disordered guest molecules within the framework.

```
PLAT770_ALERT_2_A Suspect C-H Bond in CIF:  C_6_1    --H_6_1_H   .       1.60 Ang.
```

Author Response: The close contact of this alert could be due to the disordered guest molecules within the framework.

```
PLAT770_ALERT_2_A Suspect C-H Bond in CIF:  H_1_1_H  --C_5_1     .       1.45 Ang.
```

Author Response: The close contact of this alert could be due to the disordered guest molecules within the framework.

```
PLAT770_ALERT_2_A Suspect C-H Bond in CIF:  H_3_1_H  --C_6_3     .       1.31 Ang.
```

Author Response: The close contact of this alert could be due to the disordered guest molecules within the framework.

```
PLAT770_ALERT_2_A Suspect C-H Bond in CIF:  H_3_1_H  --C_5_3     .       1.37 Ang.
```

Author Response: The close contact of this alert could be due to the disordered guest molecules within the framework.

```
PLAT770_ALERT_2_A Suspect C-H Bond in CIF:  H_3_1_H  --C_3       .       1.47 Ang.
```

Author Response: The close contact of this alert could be due to the disordered guest molecules within the framework.

```
PLAT770_ALERT_2_A Suspect C-H Bond in CIF:  H_3_1_H  --C_3       .       1.57 Ang.
```

Author Response: The close contact of this alert could be due to the disordered guest molecules within the framework.

PLAT770_ALERT_2_A Suspect C-H Bond in CIF: H_5_1_H --C_5_1 . 1.36 Ang.

Author Response: The close contact of this alert could be due to the disordered guest molecules within the framework.

PLAT770_ALERT_2_A Suspect C-H Bond in CIF: H_6_1_H --C_6_1 . 1.58 Ang.

Author Response: The close contact of this alert could be due to the disordered guest molecules within the framework.

PLAT770_ALERT_2_A Suspect C-H Bond in CIF: H_6_1_H --C_6_1 . 1.60 Ang.

Author Response: The close contact of this alert could be due to the disordered guest molecules within the framework.

PLAT770_ALERT_2_A Suspect C-H Bond in CIF: C_5_3 --H_3_1_H . 1.37 Ang.

Author Response: The close contact of this alert could be due to the disordered guest molecules within the framework.

PLAT770_ALERT_2_A Suspect C-H Bond in CIF: C_6_3 --H_3_1_H . 1.31 Ang.

Author Response: The close contact of this alert could be due to the disordered guest molecules within the framework.

Alert level C

CELLK01_ALERT_1_C Check that the cell measurement temperature is in Kelvin.
Value of measurement temperature given = 10.000

Author Response: The structure is obtained from neutron powder diffraction using cold neutron source at 10K from POWGEN, BL-11A, SNS, ORNL.

PLAT041_ALERT_1_C Calc. and Reported SumFormula Strings Differ Please Check

Author Response: This is likely caused by the high symmetry and the considered decimal points of occupancy when calculating the formula.

PLAT042_ALERT_1_C Calc. and Reported MoietyFormula Strings Differ Please Check

Author Response: This is likely caused by the high symmetry and the considered decimal points of occupancy when calculating the formula.

```
PLAT043_ALERT_1_C Calculated and Reported Mol. Weight Differ by ..      14.08 Check
```

Author Response: This is likely caused by the high symmetry and the considered decimal points of occupancy when calculating the formula.

```
PLAT341_ALERT_3_C Low Bond Precision on  C-C Bonds ..............      0.014 Ang.
```

Author Response: Structure are obtained from powder diffraction refinement. Low precision could be due to insufficient resolution.

```
PLAT601_ALERT_2_C Unit Cell Contains Solvent Accessible VOIDS of .         97 Ang**3
```

Author Response: The void is not occupied likely due to kinetic hindrance

---

Alert level G

```
FORMU01_ALERT_1_G  There is a discrepancy between the atom counts in the
            _chemical_formula_sum and _chemical_formula_moiety. This is
            usually due to the moiety formula being in the wrong format.
            Atom count from _chemical_formula_sum:   C89.15999 H27.48 D41.16 O34.4
            Atom count from _chemical_formula_moiety:C89.15400 H27.476 D41.19799 O
PLAT004_ALERT_5_G Polymeric Structure Found with Maximum Dimension          3 Info
PLAT164_ALERT_4_G Nr. of Refined C-H H-Atoms in Heavy-Atom Struct.          2 Note
PLAT301_ALERT_3_G Main Residue  Disorder .............(Resd  1  )        31% Note
PLAT301_ALERT_3_G Main Residue  Disorder .............(Resd  2  )       100% Note
PLAT301_ALERT_3_G Main Residue  Disorder .............(Resd  3  )       100% Note
PLAT301_ALERT_3_G Main Residue  Disorder .............(Resd  5  )       100% Note
PLAT302_ALERT_4_G Anion/Solvent/Minor-Residue Disorder (Resd  4  )       100% Note
PLAT411_ALERT_2_G Short Inter H...H Contact  H5       ..D_5_3    .       1.95 Ang.
                                                     x,y,z  =      1_555 Check
PLAT411_ALERT_2_G Short Inter H...H Contact  H5       ..H_4_1_H  .       2.14 Ang.
                                        1/2-y,1/2-x,-1/2+z  =     22_554 Check
PLAT411_ALERT_2_G Short Inter H...H Contact  H5       ..D_4_1    .       2.14 Ang.
                                        1/2-y,1/2-x,-1/2+z  =     22_554 Check
PLAT432_ALERT_2_G Short Inter X...Y Contact  O3       ..C_4_1    .       2.50 Ang.
                                        1/2-x,1/2-y,-1/2+z  =     18_554 Check
PLAT432_ALERT_2_G Short Inter X...Y Contact  O3       ..C_5_1    .       2.97 Ang.
                                        1/2-x,1/2-y,-1/2+z  =     18_554 Check
PLAT432_ALERT_2_G Short Inter X...Y Contact  C1       ..C_4_3    .       2.98 Ang.
                                                    y,-x,z  =     10_555 Check
PLAT432_ALERT_2_G Short Inter X...Y Contact  C1       ..C_4_3    .       2.98 Ang.
                                                   y,x,1-z  =     11_556 Check
PLAT432_ALERT_2_G Short Inter X...Y Contact  C1       ..C_4_3    .       2.98 Ang.
                                                     y,x,z  =     12_555 Check
PLAT432_ALERT_2_G Short Inter X...Y Contact  C1       ..C_4_3    .       2.98 Ang.
                                                  y,-x,1-z  =      9_556 Check
PLAT432_ALERT_2_G Short Inter X...Y Contact  C4       ..C_4_1    .       2.93 Ang.
                                        1/2-y,1/2-x,-1/2+z  =     22_554 Check
PLAT432_ALERT_2_G Short Inter X...Y Contact  C4       ..C_4_1    .       2.93 Ang.
                                        1/2-x,1/2-y,-1/2+z  =     18_554 Check
PLAT432_ALERT_2_G Short Inter X...Y Contact  C4       ..C_3_2    .       3.17 Ang.
```

```
                                              x,1-y,z  =     14_565 Check
PLAT432_ALERT_2_G Short Inter X...Y Contact  C4       ..C_3_2    .       3.17 Ang.
                                              1-y,x,z  =      8_655 Check
PLAT432_ALERT_2_G Short Inter X...Y Contact  C6       ..C_5_3    .       2.77 Ang.
                                                x,y,z  =      1_555 Check
PLAT432_ALERT_2_G Short Inter X...Y Contact  C6       ..C_5      .       2.86 Ang.
                                   -1/2+y,-1/2+x,-1/2+z  =     28_444 Check
PLAT432_ALERT_2_G Short Inter X...Y Contact  C6       ..C_5      .       2.94 Ang.
                                          1-x,1-y,1-z  =     16_666 Check
PLAT432_ALERT_2_G Short Inter X...Y Contact  C6       ..C_4      .       2.95 Ang.
                                          1-x,1-y,1-z  =     16_666 Check
PLAT432_ALERT_2_G Short Inter X...Y Contact  C6       ..C_6      .       3.11 Ang.
                                          1-x,1-y,1-z  =     16_666 Check
PLAT432_ALERT_2_G Short Inter X...Y Contact  C6       ..C_3      .       3.15 Ang.
                                          1-x,1-y,1-z  =     16_666 Check
PLAT432_ALERT_2_G Short Inter X...Y Contact  C6       ..C_6      .       3.18 Ang.
                                   -1/2+y,-1/2+x,-1/2+z  =     28_444 Check
PLAT720_ALERT_4_G Number of Unusual/Non-Standard Labels .........          55 Note
PLAT764_ALERT_4_G Overcomplete CIF Bond List Detected (Rep/Expd) .       1.85 Ratio
PLAT773_ALERT_2_G Check long C-C Bond in CIF: C_1      --C_1_3           1.72 Ang.
PLAT773_ALERT_2_G Check long C-C Bond in CIF: C_1      --C_3_3           2.03 Ang.
PLAT773_ALERT_2_G Check long C-C Bond in CIF: C_1      --C_2_3           2.03 Ang.
PLAT773_ALERT_2_G Check long C-C Bond in CIF: C_2      --C_3             1.79 Ang.
PLAT773_ALERT_2_G Check long C-C Bond in CIF: C_2      --C_6_3           1.88 Ang.
PLAT773_ALERT_2_G Check long C-C Bond in CIF: C_2      --C_4_3           1.99 Ang.
PLAT773_ALERT_2_G Check long C-C Bond in CIF: C_2      --C_6_3           2.03 Ang.
PLAT773_ALERT_2_G Check long C-C Bond in CIF: C_3      --C_2             1.79 Ang.
PLAT773_ALERT_2_G Check long C-C Bond in CIF: C_3      --C_3             1.98 Ang.
PLAT773_ALERT_2_G Check long C-C Bond in CIF: C_3      --C_5_3           1.98 Ang.
PLAT773_ALERT_2_G Check long C-C Bond in CIF: C_3      --C_1_1           2.04 Ang.
PLAT773_ALERT_2_G Check long C-C Bond in CIF: C_6      --C_5_3           1.73 Ang.
PLAT773_ALERT_2_G Check long C-C Bond in CIF: C_6      --C_6             1.79 Ang.
PLAT773_ALERT_2_G Check long C-C Bond in CIF: C_6      --C_2_3           2.04 Ang.
PLAT773_ALERT_2_G Check long C-C Bond in CIF: C_1_1    --C_4_1           1.87 Ang.
PLAT773_ALERT_2_G Check long C-C Bond in CIF: C_1_1    --C_3             2.04 Ang.
PLAT773_ALERT_2_G Check long C-C Bond in CIF: C_2_1    --C_1_1           1.71 Ang.
PLAT773_ALERT_2_G Check long C-C Bond in CIF: C_2_1    --C_2_1           2.06 Ang.
PLAT773_ALERT_2_G Check long C-C Bond in CIF: C_3_1    --C_5_3           1.97 Ang.
PLAT773_ALERT_2_G Check long C-C Bond in CIF: C_3_1    --C_5_3           1.97 Ang.
PLAT773_ALERT_2_G Check long C-C Bond in CIF: C_4_1    --C_2             1.87 Ang.
PLAT773_ALERT_2_G Check long C-C Bond in CIF: C_6_1    --C_5_1           1.91 Ang.
PLAT773_ALERT_2_G Check long C-C Bond in CIF: C_6_1    --C_6_1           1.92 Ang.
PLAT773_ALERT_2_G Check long C-C Bond in CIF: C_1_2    --C_5_2           1.88 Ang.
PLAT773_ALERT_2_G Check long C-C Bond in CIF: C_1_2    --C_6_2           1.90 Ang.
PLAT773_ALERT_2_G Check long C-C Bond in CIF: C_1_2    --C_3_2           2.05 Ang.
PLAT773_ALERT_2_G Check long C-C Bond in CIF: C_2_2    --C_4_2           1.71 Ang.
PLAT773_ALERT_2_G Check long C-C Bond in CIF: C_2_2    --C_4_2           1.92 Ang.
PLAT773_ALERT_2_G Check long C-C Bond in CIF: C_3_2    --C_4_2           1.71 Ang.
PLAT773_ALERT_2_G Check long C-C Bond in CIF: C_3_2    --C_1_2           2.05 Ang.
PLAT773_ALERT_2_G Check long C-C Bond in CIF: C_4_2    --C_2_2           1.71 Ang.
PLAT773_ALERT_2_G Check long C-C Bond in CIF: C_4_2    --C_3_2           1.71 Ang.
PLAT773_ALERT_2_G Check long C-C Bond in CIF: C_4_2    --C_6_2           1.81 Ang.
PLAT773_ALERT_2_G Check long C-C Bond in CIF: C_4_2    --C_2_2           1.92 Ang.
PLAT773_ALERT_2_G Check long C-C Bond in CIF: C_5_2    --C_5_2           1.88 Ang.
PLAT773_ALERT_2_G Check long C-C Bond in CIF: C_5_2    --C_1_2           1.88 Ang.
PLAT773_ALERT_2_G Check long C-C Bond in CIF: C_5_2    --C_5_2           1.96 Ang.
PLAT773_ALERT_2_G Check long C-C Bond in CIF: C_6_2    --C_6_2           1.77 Ang.
```

```
PLAT773_ALERT_2_G Check long C-C Bond in CIF: C_6_2    --C_4_2           1.81 Ang.
PLAT773_ALERT_2_G Check long C-C Bond in CIF: C_6_2    --C_1_2           1.90 Ang.
PLAT773_ALERT_2_G Check long C-C Bond in CIF: C_1_3    --C_1             1.72 Ang.
PLAT773_ALERT_2_G Check long C-C Bond in CIF: C_2_3    --C_2_3           1.78 Ang.
PLAT773_ALERT_2_G Check long C-C Bond in CIF: C_2_3    --C_1             2.03 Ang.
PLAT773_ALERT_2_G Check long C-C Bond in CIF: C_2_3    --C_6             2.04 Ang.
PLAT773_ALERT_2_G Check long C-C Bond in CIF: C_3_3    --C_1             2.03 Ang.
PLAT773_ALERT_2_G Check long C-C Bond in CIF: C_4_3    --C_5_3           1.80 Ang.
PLAT773_ALERT_2_G Check long C-C Bond in CIF: C_4_3    --C_2             1.99 Ang.
PLAT773_ALERT_2_G Check long C-C Bond in CIF: C_5_3    --C_6             1.73 Ang.
PLAT773_ALERT_2_G Check long C-C Bond in CIF: C_5_3    --C_4_3           1.80 Ang.
PLAT773_ALERT_2_G Check long C-C Bond in CIF: C_5_3    --C_6_3           1.81 Ang.
PLAT773_ALERT_2_G Check long C-C Bond in CIF: C_5_3    --C_3             1.98 Ang.
PLAT773_ALERT_2_G Check long C-C Bond in CIF: C_6_3    --C_5_3           1.81 Ang.
PLAT773_ALERT_2_G Check long C-C Bond in CIF: C_6_3    --C_2             1.88 Ang.
PLAT773_ALERT_2_G Check long C-C Bond in CIF: C_6_3    --C_2             2.03 Ang.
PLAT778_ALERT_2_G Check O..H..X Bond in CIF: H_5_1_H  --O4              1.45 Ang.
PLAT778_ALERT_2_G Check O..H..X Bond in CIF: O4       --H_5_1_H         1.45 Ang.
PLAT778_ALERT_2_G Check O..H..X Bond in CIF: O4       --H_5_1_H         1.45 Ang.
PLAT778_ALERT_2_G Check O..H..X Bond in CIF: O4       --H_5_1_H         1.45 Ang.
PLAT778_ALERT_2_G Check O..H..X Bond in CIF: O4       --H_5_1_H         1.45 Ang.
PLAT811_ALERT_5_G No ADDSYM Analysis: Too Many Excluded Atoms ....          ! Info
```

```
 21 ALERT level A = Most likely a serious problem - resolve or explain
  0 ALERT level B = A potentially serious problem, consider carefully
  6 ALERT level C = Check. Ensure it is not caused by an omission or oversight
 90 ALERT level G = General information/check it is not something unexpected

  5 ALERT type 1 CIF construction/syntax error, inconsistent or missing data
101 ALERT type 2 Indicator that the structure model may be wrong or deficient
  5 ALERT type 3 Indicator that the structure quality may be low
  4 ALERT type 4 Improvement, methodology, query or suggestion
  2 ALERT type 5 Informative message, check
```

It is advisable to attempt to resolve as many as possible of the alerts in all categories. Often the minor alerts point to easily fixed oversights, errors and omissions in your CIF or refinement strategy, so attention to these fine details can be worthwhile. In order to resolve some of the more serious problems it may be necessary to carry out additional measurements or structure refinements. However, the purpose of your study may justify the reported deviations and the more serious of these should normally be commented upon in the discussion or experimental section of a paper or in the "special_details" fields of the CIF. checkCIF was carefully designed to identify outliers and unusual parameters, but every test has its limitations and alerts that are not important in a particular case may appear. Conversely, the absence of alerts does not guarantee there are no aspects of the results needing attention. It is up to the individual to critically assess their own results and, if necessary, seek expert advice.

Publication of your CIF in IUCr journals

A basic structural check has been run on your CIF. These basic checks will be run on all CIFs submitted for publication in IUCr journals (*Acta Crystallographica*, *Journal of Applied Crystallography*, *Journal of Synchrotron Radiation*); however, if you intend to submit to *Acta Crystallographica Section C* or *E* or *IUCrData*, you should make sure that full publication checks are run on the final version of your CIF prior to submission.

Publication of your CIF in other journals

Please refer to the *Notes for Authors* of the relevant journal for any special instructions relating to CIF submission.

PLATON version of 06/07/2023; check.def file version of 30/06/2023

Datablock C6D6@MIL-125-defect - ellipsoid plot

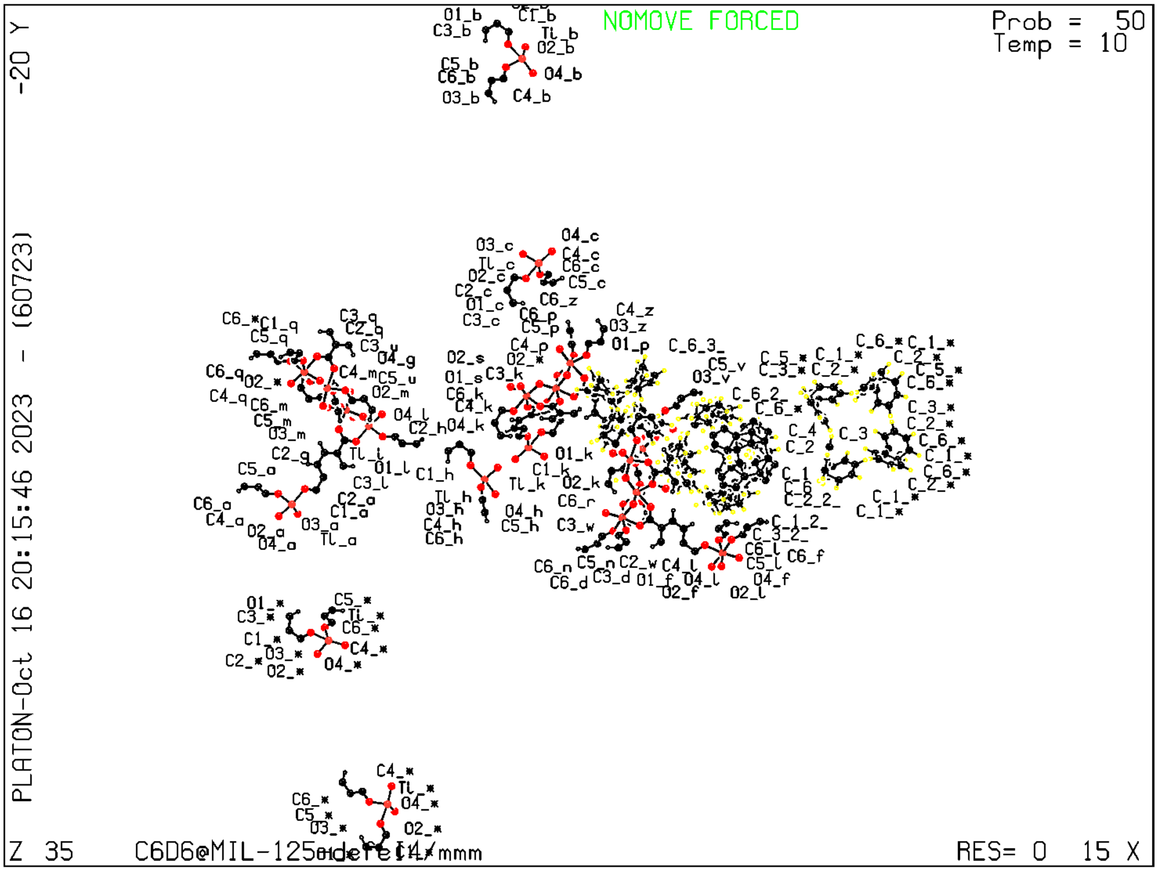

Supplement: Supplementary file 2 — Crystallographic data (11 CIFs) and checkCIF reports. [file 41563_2024_2029_MOESM2_ESM.zip › cifs and check cif reports/C6D6@MIL-125-defect_checkcif.pdf]
