# Supplementary material for: Trace benzene capture by decoration of structural defects in metal–organic framework materials
Source: Nat Mater. 2024 Oct 29;23(11):1531–8. doi: 10.1038/s41563-024-02029-1 (PMC11525167; doi:10.1038/s41563-024-02029-1)

checkCIF/PLATON report

You have not supplied any structure factors. As a result the full set of tests cannot be run.

THIS REPORT IS FOR GUIDANCE ONLY. IF USED AS PART OF A REVIEW PROCEDURE FOR PUBLICATION, IT SHOULD NOT REPLACE THE EXPERTISE OF AN EXPERIENCED CRYSTALLOGRAPHIC REFEREE.

No syntax errors found.   CIF dictionary   Interpreting this report

Datablock: C6D6-MIL-125-Zn-high

Bond precision: C-C = 0.0130 A   Wavelength=0

Cell:	a=18.6048(6)	b=18.6048(6)	c=18.1660(6)
	alpha=90	beta=90	gamma=90
Temperature:	10 K		

	Calculated	Reported
Volume	6288.0(5)	6288.0(4)
Space group	I 4/m m m	I 4/m m m
Hall group	-I 4 2	-I 4 2
Moiety formula	C48 H27.32 O33.96 Ti6.64 Zn1.36, 0.026(C80 D80), 0.167(C96 D88)	C48 H27.32 O33.96 Ti6.64 Zn1.36, 10.595(C6 D6)
Sum formula	C111.15 H28.68 D60.31 O35.32 Ti6.64 Zn1.36	C111.57 H27.32 D63.57 O33.96 Ti6.64 Zn1.36
Mr	2456.98	2456.98
Dx,g cm-3	1.298	1.298
Z	2	2
Mu (mm-1)	0.000	0.000
F000	2449.1	0.0
F000’	2450.38	
h,k,lmax		
Nref		
Tmin,Tmax		
Tmin’		

Correction method= Not given

Data completeness=   Theta(max)=

R(reflections)=   wR2(reflections)=

S =   Npar=

The following ALERTS were generated. Each ALERT has the format
test-name_ALERT_alert-type_alert-level.
Click on the hyperlinks for more details of the test.

Alert level A

PLAT770_ALERT_2_A Suspect C-H Bond in CIF: C_1_2 --H4O . 1.37 Ang.

Author Response: The framework structure contains disordered framework and disordered guest molecules within the framework. The close contact of this alert could be due to the mentioned disorder.

PLAT770_ALERT_2_A Suspect C-H Bond in CIF: C_4_2 --H4O . 1.47 Ang.

Author Response: The framework structure contains disordered framework and disordered guest molecules within the framework. The close contact of this alert could be due to the mentioned disorder.

PLAT770_ALERT_2_A Suspect C-H Bond in CIF: C_6_2 --H4O . 1.36 Ang.

Author Response: The framework structure contains disordered framework and disordered guest molecules within the framework. The close contact of this alert could be due to the mentioned disorder.

PLAT770_ALERT_2_A Suspect C-H Bond in CIF: H4O --C_6_2 . 1.36 Ang.

Author Response: The framework structure contains disordered framework and disordered guest molecules within the framework. The close contact of this alert could be due to the mentioned disorder.

PLAT770_ALERT_2_A Suspect C-H Bond in CIF: H4O --C_6_2 . 1.36 Ang.

Author Response: The framework structure contains disordered framework and disordered guest molecules within the framework. The close contact of this alert could be due to the mentioned disorder.

PLAT770_ALERT_2_A Suspect C-H Bond in CIF: H4O --C_6_2 . 1.36 Ang.

Author Response: The framework structure contains disordered framework and disordered guest molecules within the framework. The close contact of this alert could be due to the mentioned disorder.

PLAT770_ALERT_2_A Suspect C-H Bond in CIF: H4O   --C_6_2   .   1.36 Ang.

Author Response: The framework structure contains disordered framework and disordered guest molecules within the framework. The close contact of this alert could be due to the mentioned disorder.

PLAT770_ALERT_2_A Suspect C-H Bond in CIF: H4O   --C_1_2   .   1.37 Ang.

Author Response: The framework structure contains disordered framework and disordered guest molecules within the framework. The close contact of this alert could be due to the mentioned disorder.

PLAT770_ALERT_2_A Suspect C-H Bond in CIF: H4O   --C_1_2   .   1.37 Ang.

Author Response: The framework structure contains disordered framework and disordered guest molecules within the framework. The close contact of this alert could be due to the mentioned disorder.

PLAT770_ALERT_2_A Suspect C-H Bond in CIF: H4O   --C_1_2   .   1.37 Ang.

Author Response: The framework structure contains disordered framework and disordered guest molecules within the framework. The close contact of this alert could be due to the mentioned disorder.

PLAT770_ALERT_2_A Suspect C-H Bond in CIF: H4O   --C_1_2   .   1.37 Ang.

Author Response: The framework structure contains disordered framework and disordered guest molecules within the framework. The close contact of this alert could be due to the mentioned disorder.

PLAT770_ALERT_2_A Suspect C-H Bond in CIF: H4O   --C_4_2   .   1.47 Ang.

Author Response: The framework structure contains disordered framework and disordered guest molecules within the framework. The close contact of this alert could be due to the mentioned disorder.

PLAT770_ALERT_2_A Suspect C-H Bond in CIF: H4O   --C_4_2   .   1.47 Ang.

Author Response: The framework structure contains disordered framework and disordered guest molecules within the framework. The close contact of this alert could be due to the mentioned disorder.

PLAT770_ALERT_2_A Suspect C-H Bond in CIF: H4O --C_4_2 . 1.47 Ang.

Author Response: The framework structure contains disordered framework and disordered guest molecules within the framework. The close contact of this alert could be due to the mentioned disorder.

PLAT770_ALERT_2_A Suspect C-H Bond in CIF: H4O --C_4_2 . 1.47 Ang.

Author Response: The framework structure contains disordered framework and disordered guest molecules within the framework. The close contact of this alert could be due to the mentioned disorder.

Alert level B

PLAT420_ALERT_2_B D-H Bond Without Acceptor O4 --H4O . Please Check

Author Response: The guest molecule benzenen does not have a hydrogen bonding acceptor.

Alert level C

CELLK01_ALERT_1_C Check that the cell measurement temperature is in Kelvin.
Value of measurement temperature given = 10.000

Author Response: The structure is obtained from neutron powder diffraction using cold neutron source at 10K from POWGEN, BL-11A, SNS, ORNL.

CHEMW01_ALERT_1_C The difference between the given and expected weight for
compound is greater 1 mass unit. Check that all hydrogen
atoms have been taken into account.

Author Response: This is likely due to the decimal points considers in the calculation. The reported strings are consistent with the atom sites information.

PLAT041_ALERT_1_C Calc. and Reported SumFormula Strings Differ Please Check

Author Response: This is likely to the decimal points considers in the calculation. The reported strings are consistent with the atom sites information.

PLAT042_ALERT_1_C Calc. and Reported MoietyFormula Strings Differ Please Check

Author Response: This is likely due to the decimal points considers in the calculation. The reported strings are consistent with the atom sites information.

```
PLAT077_ALERT_4_C Unitcell Contains Non-integer Number of Atoms ..     Please Check
```

Author Response: The structure contains disordered framework and guest molecules.

```
PLAT341_ALERT_3_C Low Bond Precision on  C-C Bonds ...............      0.013 Ang.
```

Author Response: Structure are obtained from powder diffraction refinement.

Alert level G

```
FORMU01_ALERT_2_G  There is a discrepancy between the atom counts in the
            _chemical_formula_sum and the formula from the _atom_site* data.
            Atom count from _chemical_formula_sum:C111.57 H27.32 D63.57 O33.96 Ti6
            Atom count from the _atom_site data:  C111.5712 H28.68 D63.57121 O35.3
CELLZ01_ALERT_1_G Difference between formula and atom_site contents detected.
CELLZ01_ALERT_1_G ALERT: Large difference may be due to a
            symmetry error - see SYMMG tests
           From the CIF: _cell_formula_units_Z    2
           From the CIF: _chemical_formula_sum  C111.57 H27.32 D63.57 O33.96 Ti6.6
           TEST: Compare cell contents of formula and atom_site data

           atom    Z*formula  cif sites diff
           C        223.14    223.14   -0.00
           H         54.64     57.36   -2.72
           D        127.14    127.14   -0.00
           O         67.92     70.64   -2.72
           Ti        13.28     13.28    0.00
           Zn         2.72      2.72    0.00
PLAT300_ALERT_4_G Atom Site Occupancy of C3         Constrained at        0.5 Check
PLAT300_ALERT_4_G Atom Site Occupancy of C6         Constrained at        0.5 Check
PLAT300_ALERT_4_G Atom Site Occupancy of C66        Constrained at        0.5 Check
PLAT300_ALERT_4_G Atom Site Occupancy of H4         Constrained at        0.5 Check
PLAT300_ALERT_4_G Atom Site Occupancy of H5         Constrained at        0.5 Check
PLAT300_ALERT_4_G Atom Site Occupancy of H55        Constrained at        0.5 Check
PLAT301_ALERT_3_G Main Residue  Disorder ..............(Resd  1  )        58% Note
PLAT301_ALERT_3_G Main Residue  Disorder ..............(Resd  2  )       100% Note
PLAT301_ALERT_3_G Main Residue  Disorder ..............(Resd  3  )       100% Note
PLAT301_ALERT_3_G Main Residue  Disorder ..............(Resd  4  )       100% Note
PLAT301_ALERT_3_G Main Residue  Disorder ..............(Resd  5  )       100% Note
PLAT302_ALERT_4_G Anion/Solvent/Minor-Residue Disorder (Resd  6  )       100% Note
PLAT302_ALERT_4_G Anion/Solvent/Minor-Residue Disorder (Resd  7  )       100% Note
PLAT304_ALERT_4_G Non-Integer Number of Atoms in ..... (Resd  1  )     117.28 Check
PLAT304_ALERT_4_G Non-Integer Number of Atoms in ..... (Resd  2  )       4.19 Check
PLAT304_ALERT_4_G Non-Integer Number of Atoms in ..... (Resd  3  )      30.73 Check
PLAT304_ALERT_4_G Non-Integer Number of Atoms in ..... (Resd  4  )      14.21 Check
PLAT304_ALERT_4_G Non-Integer Number of Atoms in ..... (Resd  5  )      21.79 Check
PLAT304_ALERT_4_G Non-Integer Number of Atoms in ..... (Resd  6  )       8.27 Check
PLAT304_ALERT_4_G Non-Integer Number of Atoms in ..... (Resd  7  )       0.34 Check
```

```
PLAT432_ALERT_2_G Short Inter X...Y Contact  O3       ..C_4_2    .       2.98 Ang.
                                         1/2-x,1/2-y,-1/2+z  =     18_554 Check
PLAT432_ALERT_2_G Short Inter X...Y Contact  C2       ..C3       .       2.39 Ang.
                                                  1-x,y,1-z  =      3_656 Check
PLAT432_ALERT_2_G Short Inter X...Y Contact  C2       ..C3       .       2.39 Ang.
                                                    1-x,y,z  =      4_655 Check
PLAT432_ALERT_2_G Short Inter X...Y Contact  C2       ..C3       .       2.39 Ang.
                                                   1-x,-y,z  =      2_655 Check
PLAT432_ALERT_2_G Short Inter X...Y Contact  C2       ..C3       .       2.39 Ang.
                                                 1-x,-y,1-z  =     16_656 Check
PLAT432_ALERT_2_G Short Inter X...Y Contact  C2       ..C2       .       2.76 Ang.
                                                   1-x,-y,z  =      2_655 Check
PLAT432_ALERT_2_G Short Inter X...Y Contact  C3       ..C3       .       1.36 Ang.
                                                    1-x,y,z  =      4_655 Check
PLAT432_ALERT_2_G Short Inter X...Y Contact  C3       ..C3       .       1.43 Ang.
                                                  1-x,y,1-z  =      3_656 Check
PLAT432_ALERT_2_G Short Inter X...Y Contact  C3       ..C3       .       2.75 Ang.
                                                   1-x,-y,z  =      2_655 Check
PLAT432_ALERT_2_G Short Inter X...Y Contact  C3       ..C3       .       2.78 Ang.
                                                 1-x,-y,1-z  =     16_656 Check
PLAT432_ALERT_2_G Short Inter X...Y Contact  C4       ..C_4_2    .       3.17 Ang.
                                         1/2-y,1/2-x,-1/2+z  =     22_554 Check
PLAT432_ALERT_2_G Short Inter X...Y Contact  C4       ..C_4_2    .       3.17 Ang.
                                         1/2-x,1/2-y,-1/2+z  =     18_554 Check
PLAT432_ALERT_2_G Short Inter X...Y Contact  C5       ..C66      .       2.42 Ang.
                                          1/2-x,1/2-y,1/2-z  =     32_555 Check
PLAT432_ALERT_2_G Short Inter X...Y Contact  C5       ..C66      .       2.42 Ang.
                                          1/2-y,1/2-x,1/2-z  =     21_555 Check
PLAT432_ALERT_2_G Short Inter X...Y Contact  C5       ..C6       .       2.43 Ang.
                                          1/2-x,1/2-y,1/2-z  =     32_555 Check
PLAT432_ALERT_2_G Short Inter X...Y Contact  C5       ..C6       .       2.43 Ang.
                                          1/2-y,1/2-x,1/2-z  =     21_555 Check
PLAT432_ALERT_2_G Short Inter X...Y Contact  C5       ..C5       .       2.80 Ang.
                                          1/2-y,1/2-x,1/2-z  =     21_555 Check
PLAT432_ALERT_2_G Short Inter X...Y Contact  C6       ..C66      .       1.40 Ang.
                                          1/2-x,1/2-y,1/2-z  =     32_555 Check
PLAT432_ALERT_2_G Short Inter X...Y Contact  C6       ..C6       .       1.45 Ang.
                                          1/2-y,1/2-x,1/2-z  =     21_555 Check
PLAT432_ALERT_2_G Short Inter X...Y Contact  C6       ..C66      .       2.78 Ang.
                                          1/2-y,1/2-x,1/2-z  =     21_555 Check
PLAT432_ALERT_2_G Short Inter X...Y Contact  C6       ..C6       .       2.81 Ang.
                                          1/2-x,1/2-y,1/2-z  =     32_555 Check
PLAT432_ALERT_2_G Short Inter X...Y Contact  C66      ..C66      .       1.43 Ang.
                                          1/2-y,1/2-x,1/2-z  =     21_555 Check
PLAT432_ALERT_2_G Short Inter X...Y Contact  C66      ..C66      .       2.79 Ang.
                                          1/2-x,1/2-y,1/2-z  =     32_555 Check
PLAT720_ALERT_4_G Number of Unusual/Non-Standard Labels .........         60 Note
PLAT764_ALERT_4_G Overcomplete CIF Bond List Detected (Rep/Expd) .       3.36 Ratio
PLAT773_ALERT_2_G Check long C-C Bond in CIF: C_1      --C_2             1.72 Ang.
PLAT773_ALERT_2_G Check long C-C Bond in CIF: C_1      --C_6_3           1.72 Ang.
PLAT773_ALERT_2_G Check long C-C Bond in CIF: C_1      --C_5             1.95 Ang.
PLAT773_ALERT_2_G Check long C-C Bond in CIF: C_1      --C_5             2.06 Ang.
PLAT773_ALERT_2_G Check long C-C Bond in CIF: C_2      --C_1             1.72 Ang.
PLAT773_ALERT_2_G Check long C-C Bond in CIF: C_2      --C_1_3           1.79 Ang.
PLAT773_ALERT_2_G Check long C-C Bond in CIF: C_2      --C_4             1.85 Ang.
PLAT773_ALERT_2_G Check long C-C Bond in CIF: C_2      --C_4             1.93 Ang.
PLAT773_ALERT_2_G Check long C-C Bond in CIF: C_2      --C_6             2.02 Ang.
```

```
PLAT773_ALERT_2_G Check long C-C Bond in CIF: C_3      --C_1_3           1.99 Ang.
PLAT773_ALERT_2_G Check long C-C Bond in CIF: C_3      --C_1_3           2.01 Ang.
PLAT773_ALERT_2_G Check long C-C Bond in CIF: C_3      --C_6             2.06 Ang.
PLAT773_ALERT_2_G Check long C-C Bond in CIF: C_4      --C_4             1.75 Ang.
PLAT773_ALERT_2_G Check long C-C Bond in CIF: C_4      --C_2             1.85 Ang.
PLAT773_ALERT_2_G Check long C-C Bond in CIF: C_4      --C_2             1.93 Ang.
PLAT773_ALERT_2_G Check long C-C Bond in CIF: C_4      --C_4             1.94 Ang.
PLAT773_ALERT_2_G Check long C-C Bond in CIF: C_4      --C_6             1.98 Ang.
PLAT773_ALERT_2_G Check long C-C Bond in CIF: C_5      --C_6             1.73 Ang.
PLAT773_ALERT_2_G Check long C-C Bond in CIF: C_5      --C_1             1.95 Ang.
PLAT773_ALERT_2_G Check long C-C Bond in CIF: C_5      --C_6             2.04 Ang.
PLAT773_ALERT_2_G Check long C-C Bond in CIF: C_5      --C_5             2.04 Ang.
PLAT773_ALERT_2_G Check long C-C Bond in CIF: C_5      --C_5             2.04 Ang.
PLAT773_ALERT_2_G Check long C-C Bond in CIF: C_5      --C_1             2.06 Ang.
PLAT773_ALERT_2_G Check long C-C Bond in CIF: C_6      --C_5             1.73 Ang.
PLAT773_ALERT_2_G Check long C-C Bond in CIF: C_6      --C_4             1.98 Ang.
PLAT773_ALERT_2_G Check long C-C Bond in CIF: C_6      --C_2             2.02 Ang.
PLAT773_ALERT_2_G Check long C-C Bond in CIF: C_6      --C_5             2.04 Ang.
PLAT773_ALERT_2_G Check long C-C Bond in CIF: C_6      --C_3             2.06 Ang.
PLAT773_ALERT_2_G Check long C-C Bond in CIF: C_1_2    --C_4_2           1.80 Ang.
PLAT773_ALERT_2_G Check long C-C Bond in CIF: C_1_2    --C_4_2           1.82 Ang.
PLAT773_ALERT_2_G Check long C-C Bond in CIF: C_1_2    --C_5_2           1.88 Ang.
PLAT773_ALERT_2_G Check long C-C Bond in CIF: C_1_2    --C_3_2           1.89 Ang.
PLAT773_ALERT_2_G Check long C-C Bond in CIF: C_1_2    --C_5_2           1.90 Ang.
PLAT773_ALERT_2_G Check long C-C Bond in CIF: C_1_2    --C_5_2           1.92 Ang.
PLAT773_ALERT_2_G Check long C-C Bond in CIF: C_1_2    --C_3_2           1.92 Ang.
PLAT773_ALERT_2_G Check long C-C Bond in CIF: C_1_2    --C_5_2           1.95 Ang.
PLAT773_ALERT_2_G Check long C-C Bond in CIF: C_1_2    --C_5_2           2.04 Ang.
PLAT773_ALERT_2_G Check long C-C Bond in CIF: C_2_2    --C_6_2           1.71 Ang.
PLAT773_ALERT_2_G Check long C-C Bond in CIF: C_2_2    --C_6_2           1.72 Ang.
PLAT773_ALERT_2_G Check long C-C Bond in CIF: C_2_2    --C_6_2           1.72 Ang.
PLAT773_ALERT_2_G Check long C-C Bond in CIF: C_2_2    --C_4_2           1.92 Ang.
PLAT773_ALERT_2_G Check long C-C Bond in CIF: C_2_2    --C_4_2           1.96 Ang.
PLAT773_ALERT_2_G Check long C-C Bond in CIF: C_2_2    --C_4_2           1.96 Ang.
PLAT773_ALERT_2_G Check long C-C Bond in CIF: C_2_2    --C_4_2           2.02 Ang.
PLAT773_ALERT_2_G Check long C-C Bond in CIF: C_3_2    --C_1_2           1.89 Ang.
PLAT773_ALERT_2_G Check long C-C Bond in CIF: C_3_2    --C_3_2           1.90 Ang.
PLAT773_ALERT_2_G Check long C-C Bond in CIF: C_3_2    --C_1_2           1.92 Ang.
PLAT773_ALERT_2_G Check long C-C Bond in CIF: C_3_2    --C_6_2           2.06 Ang.
PLAT773_ALERT_2_G Check long C-C Bond in CIF: C_3_2    --C_6_2           2.06 Ang.
PLAT773_ALERT_2_G Check long C-C Bond in CIF: C_4_2    --C_1_2           1.80 Ang.
PLAT773_ALERT_2_G Check long C-C Bond in CIF: C_4_2    --C_1_2           1.82 Ang.
PLAT773_ALERT_2_G Check long C-C Bond in CIF: C_4_2    --C_2_2           1.92 Ang.
PLAT773_ALERT_2_G Check long C-C Bond in CIF: C_4_2    --C_6_2           1.93 Ang.
PLAT773_ALERT_2_G Check long C-C Bond in CIF: C_4_2    --C_6_2           1.93 Ang.
PLAT773_ALERT_2_G Check long C-C Bond in CIF: C_4_2    --C_2_2           1.96 Ang.
PLAT773_ALERT_2_G Check long C-C Bond in CIF: C_4_2    --C_2_2           1.96 Ang.
PLAT773_ALERT_2_G Check long C-C Bond in CIF: C_4_2    --C_2_2           2.02 Ang.
PLAT773_ALERT_2_G Check long C-C Bond in CIF: C_5_2    --C_1_2           1.88 Ang.
PLAT773_ALERT_2_G Check long C-C Bond in CIF: C_5_2    --C_6_2           1.90 Ang.
PLAT773_ALERT_2_G Check long C-C Bond in CIF: C_5_2    --C_1_2           1.90 Ang.
PLAT773_ALERT_2_G Check long C-C Bond in CIF: C_5_2    --C_6_2           1.92 Ang.
PLAT773_ALERT_2_G Check long C-C Bond in CIF: C_5_2    --C_1_2           1.92 Ang.
PLAT773_ALERT_2_G Check long C-C Bond in CIF: C_5_2    --C_6_2           1.93 Ang.
PLAT773_ALERT_2_G Check long C-C Bond in CIF: C_5_2    --C_6_2           1.94 Ang.
PLAT773_ALERT_2_G Check long C-C Bond in CIF: C_5_2    --C_1_2           1.95 Ang.
PLAT773_ALERT_2_G Check long C-C Bond in CIF: C_5_2    --C_6_2           1.95 Ang.
```

```
PLAT773_ALERT_2_G Check long C-C Bond in CIF: C_5_2    --C_6_2           1.96 Ang.
PLAT773_ALERT_2_G Check long C-C Bond in CIF: C_5_2    --C_6_2           1.97 Ang.
PLAT773_ALERT_2_G Check long C-C Bond in CIF: C_5_2    --C_6_2           1.99 Ang.
PLAT773_ALERT_2_G Check long C-C Bond in CIF: C_5_2    --C_1_2           2.04 Ang.
PLAT773_ALERT_2_G Check long C-C Bond in CIF: C_6_2    --C_2_2           1.71 Ang.
PLAT773_ALERT_2_G Check long C-C Bond in CIF: C_6_2    --C_2_2           1.72 Ang.
PLAT773_ALERT_2_G Check long C-C Bond in CIF: C_6_2    --C_2_2           1.72 Ang.
PLAT773_ALERT_2_G Check long C-C Bond in CIF: C_6_2    --C_5_2           1.90 Ang.
PLAT773_ALERT_2_G Check long C-C Bond in CIF: C_6_2    --C_5_2           1.92 Ang.
PLAT773_ALERT_2_G Check long C-C Bond in CIF: C_6_2    --C_4_2           1.93 Ang.
PLAT773_ALERT_2_G Check long C-C Bond in CIF: C_6_2    --C_4_2           1.93 Ang.
PLAT773_ALERT_2_G Check long C-C Bond in CIF: C_6_2    --C_5_2           1.93 Ang.
PLAT773_ALERT_2_G Check long C-C Bond in CIF: C_6_2    --C_5_2           1.94 Ang.
PLAT773_ALERT_2_G Check long C-C Bond in CIF: C_6_2    --C_5_2           1.95 Ang.
PLAT773_ALERT_2_G Check long C-C Bond in CIF: C_6_2    --C_5_2           1.96 Ang.
PLAT773_ALERT_2_G Check long C-C Bond in CIF: C_6_2    --C_5_2           1.97 Ang.
PLAT773_ALERT_2_G Check long C-C Bond in CIF: C_6_2    --C_5_2           1.99 Ang.
PLAT773_ALERT_2_G Check long C-C Bond in CIF: C_6_2    --C_3_2           2.06 Ang.
PLAT773_ALERT_2_G Check long C-C Bond in CIF: C_6_2    --C_3_2           2.06 Ang.
PLAT773_ALERT_2_G Check long C-C Bond in CIF: C_1_3    --C_2             1.79 Ang.
PLAT773_ALERT_2_G Check long C-C Bond in CIF: C_1_3    --C_1_4           1.81 Ang.
PLAT773_ALERT_2_G Check long C-C Bond in CIF: C_1_3    --C_1_4           1.83 Ang.
PLAT773_ALERT_2_G Check long C-C Bond in CIF: C_1_3    --C_3             1.99 Ang.
PLAT773_ALERT_2_G Check long C-C Bond in CIF: C_1_3    --C_3             2.01 Ang.
PLAT773_ALERT_2_G Check long C-C Bond in CIF: C_2_3    --C_2_4           1.92 Ang.
PLAT773_ALERT_2_G Check long C-C Bond in CIF: C_3_3    --C_1_4           1.72 Ang.
PLAT773_ALERT_2_G Check long C-C Bond in CIF: C_3_3    --C_6_5           2.02 Ang.
PLAT773_ALERT_2_G Check long C-C Bond in CIF: C_3_3    --C_1_5           2.03 Ang.
PLAT773_ALERT_2_G Check long C-C Bond in CIF: C_4_3    --C_4_5           1.71 Ang.
PLAT773_ALERT_2_G Check long C-C Bond in CIF: C_4_3    --C_3_5           1.72 Ang.
PLAT773_ALERT_2_G Check long C-C Bond in CIF: C_4_3    --C_1_4           1.78 Ang.
PLAT773_ALERT_2_G Check long C-C Bond in CIF: C_4_3    --C_1_4           1.92 Ang.
PLAT773_ALERT_2_G Check long C-C Bond in CIF: C_4_3    --C_5_5           1.94 Ang.
PLAT773_ALERT_2_G Check long C-C Bond in CIF: C_5_3    --C_6_3           1.75 Ang.
PLAT773_ALERT_2_G Check long C-C Bond in CIF: C_5_3    --C_5_3           1.93 Ang.
PLAT773_ALERT_2_G Check long C-C Bond in CIF: C_5_3    --C_4_5           1.94 Ang.
PLAT773_ALERT_2_G Check long C-C Bond in CIF: C_5_3    --C_1_4           1.98 Ang.
PLAT773_ALERT_2_G Check long C-C Bond in CIF: C_6_3    --C_1             1.72 Ang.
PLAT773_ALERT_2_G Check long C-C Bond in CIF: C_6_3    --C_5_3           1.75 Ang.
PLAT773_ALERT_2_G Check long C-C Bond in CIF: C_6_3    --C_1_4           2.00 Ang.
PLAT773_ALERT_2_G Check long C-C Bond in CIF: C_6_3    --C_1_4           2.02 Ang.
PLAT773_ALERT_2_G Check long C-C Bond in CIF: C_1_4    --C_3_3           1.72 Ang.
PLAT773_ALERT_2_G Check long C-C Bond in CIF: C_1_4    --C_4_3           1.78 Ang.
PLAT773_ALERT_2_G Check long C-C Bond in CIF: C_1_4    --C_1_3           1.81 Ang.
PLAT773_ALERT_2_G Check long C-C Bond in CIF: C_1_4    --C_1_3           1.83 Ang.
PLAT773_ALERT_2_G Check long C-C Bond in CIF: C_1_4    --C_4_3           1.92 Ang.
PLAT773_ALERT_2_G Check long C-C Bond in CIF: C_1_4    --C_5_3           1.98 Ang.
PLAT773_ALERT_2_G Check long C-C Bond in CIF: C_1_4    --C_6_3           2.00 Ang.
PLAT773_ALERT_2_G Check long C-C Bond in CIF: C_1_4    --C_6_3           2.02 Ang.
PLAT773_ALERT_2_G Check long C-C Bond in CIF: C_2_4    --C_6_4           1.78 Ang.
PLAT773_ALERT_2_G Check long C-C Bond in CIF: C_2_4    --C_2_3           1.92 Ang.
PLAT773_ALERT_2_G Check long C-C Bond in CIF: C_3_4    --C_4_4           1.74 Ang.
PLAT773_ALERT_2_G Check long C-C Bond in CIF: C_3_4    --C_4_4           1.81 Ang.
PLAT773_ALERT_2_G Check long C-C Bond in CIF: C_3_4    --C_5_4           1.82 Ang.
PLAT773_ALERT_2_G Check long C-C Bond in CIF: C_3_4    --C_3_4           2.01 Ang.
PLAT773_ALERT_2_G Check long C-C Bond in CIF: C_4_4    --C_3_4           1.74 Ang.
PLAT773_ALERT_2_G Check long C-C Bond in CIF: C_4_4    --C_5_4           1.80 Ang.
```

```
PLAT773_ALERT_2_G Check long C-C Bond in CIF: C_4_4    --C_3_4           1.81 Ang.
PLAT773_ALERT_2_G Check long C-C Bond in CIF: C_4_4    --C_4_4           1.93 Ang.
PLAT773_ALERT_2_G Check long C-C Bond in CIF: C_5_4    --C_4_4           1.80 Ang.
PLAT773_ALERT_2_G Check long C-C Bond in CIF: C_5_4    --C_3_4           1.82 Ang.
PLAT773_ALERT_2_G Check long C-C Bond in CIF: C_5_4    --C_1_5           1.94 Ang.
PLAT773_ALERT_2_G Check long C-C Bond in CIF: C_5_4    --C_6_4           2.04 Ang.
PLAT773_ALERT_2_G Check long C-C Bond in CIF: C_6_4    --C_2_5           1.75 Ang.
PLAT773_ALERT_2_G Check long C-C Bond in CIF: C_6_4    --C_2_4           1.78 Ang.
PLAT773_ALERT_2_G Check long C-C Bond in CIF: C_6_4    --C_6_5           1.81 Ang.
PLAT773_ALERT_2_G Check long C-C Bond in CIF: C_6_4    --C_5_4           2.04 Ang.
PLAT773_ALERT_2_G Check long C-C Bond in CIF: C_1_5    --C_6_5           1.91 Ang.
PLAT773_ALERT_2_G Check long C-C Bond in CIF: C_1_5    --C_5_4           1.94 Ang.
PLAT773_ALERT_2_G Check long C-C Bond in CIF: C_1_5    --C_3_3           2.03 Ang.
PLAT773_ALERT_2_G Check long C-C Bond in CIF: C_1_5    --C_2_5           2.04 Ang.
PLAT773_ALERT_2_G Check long C-C Bond in CIF: C_2_5    --C_6_4           1.75 Ang.
PLAT773_ALERT_2_G Check long C-C Bond in CIF: C_2_5    --C_3_5           1.94 Ang.
PLAT773_ALERT_2_G Check long C-C Bond in CIF: C_2_5    --C_1_5           2.04 Ang.
PLAT773_ALERT_2_G Check long C-C Bond in CIF: C_3_5    --C_4_3           1.72 Ang.
PLAT773_ALERT_2_G Check long C-C Bond in CIF: C_3_5    --C_4_5           1.76 Ang.
PLAT773_ALERT_2_G Check long C-C Bond in CIF: C_3_5    --C_2_5           1.94 Ang.
PLAT773_ALERT_2_G Check long C-C Bond in CIF: C_3_5    --C_3_5           2.00 Ang.
PLAT773_ALERT_2_G Check long C-C Bond in CIF: C_3_5    --C_6_5           2.05 Ang.
PLAT773_ALERT_2_G Check long C-C Bond in CIF: C_4_5    --C_4_3           1.71 Ang.
PLAT773_ALERT_2_G Check long C-C Bond in CIF: C_4_5    --C_6_5           1.72 Ang.
PLAT773_ALERT_2_G Check long C-C Bond in CIF: C_4_5    --C_3_5           1.76 Ang.
PLAT773_ALERT_2_G Check long C-C Bond in CIF: C_4_5    --C_5_3           1.94 Ang.
PLAT773_ALERT_2_G Check long C-C Bond in CIF: C_5_5    --C_4_3           1.94 Ang.
PLAT773_ALERT_2_G Check long C-C Bond in CIF: C_6_5    --C_4_5           1.72 Ang.
PLAT773_ALERT_2_G Check long C-C Bond in CIF: C_6_5    --C_6_4           1.81 Ang.
PLAT773_ALERT_2_G Check long C-C Bond in CIF: C_6_5    --C_1_5           1.91 Ang.
PLAT773_ALERT_2_G Check long C-C Bond in CIF: C_6_5    --C_3_3           2.02 Ang.
PLAT773_ALERT_2_G Check long C-C Bond in CIF: C_6_5    --C_3_5           2.05 Ang.
PLAT811_ALERT_5_G No ADDSYM Analysis: Too Many Excluded Atoms ....          ! Info
```

---

15 **ALERT level A** = Most likely a serious problem - resolve or explain
1 **ALERT level B** = A potentially serious problem, consider carefully
6 **ALERT level C** = Check. Ensure it is not caused by an omission or oversight
204 **ALERT level G** = General information/check it is not something unexpected

6 ALERT type 1 CIF construction/syntax error, inconsistent or missing data
195 ALERT type 2 Indicator that the structure model may be wrong or deficient
6 ALERT type 3 Indicator that the structure quality may be low
18 ALERT type 4 Improvement, methodology, query or suggestion
1 ALERT type 5 Informative message, check

It is advisable to attempt to resolve as many as possible of the alerts in all categories. Often the minor alerts point to easily fixed oversights, errors and omissions in your CIF or refinement strategy, so attention to these fine details can be worthwhile. In order to resolve some of the more serious problems it may be necessary to carry out additional measurements or structure refinements. However, the purpose of your study may justify the reported deviations and the more serious of these should normally be commented upon in the discussion or experimental section of a paper or in the "special_details" fields of the CIF. checkCIF was carefully designed to identify outliers and unusual parameters, but every test has its limitations and alerts that are not important in a particular case may appear. Conversely, the absence of alerts does not guarantee there are no aspects of the results needing attention. It is up to the individual to critically assess their own results and, if necessary, seek expert advice.

Publication of your CIF in IUCr journals

A basic structural check has been run on your CIF. These basic checks will be run on all CIFs submitted for publication in IUCr journals (*Acta Crystallographica*, *Journal of Applied Crystallography*, *Journal of Synchrotron Radiation*); however, if you intend to submit to *Acta Crystallographica Section C* or *E* or *IUCrData*, you should make sure that full publication checks are run on the final version of your CIF prior to submission.

Publication of your CIF in other journals

Please refer to the *Notes for Authors* of the relevant journal for any special instructions relating to CIF submission.

PLATON version of 06/07/2023; check.def file version of 30/06/2023

Datablock C6D6-MIL-125-Zn-high - ellipsoid plot

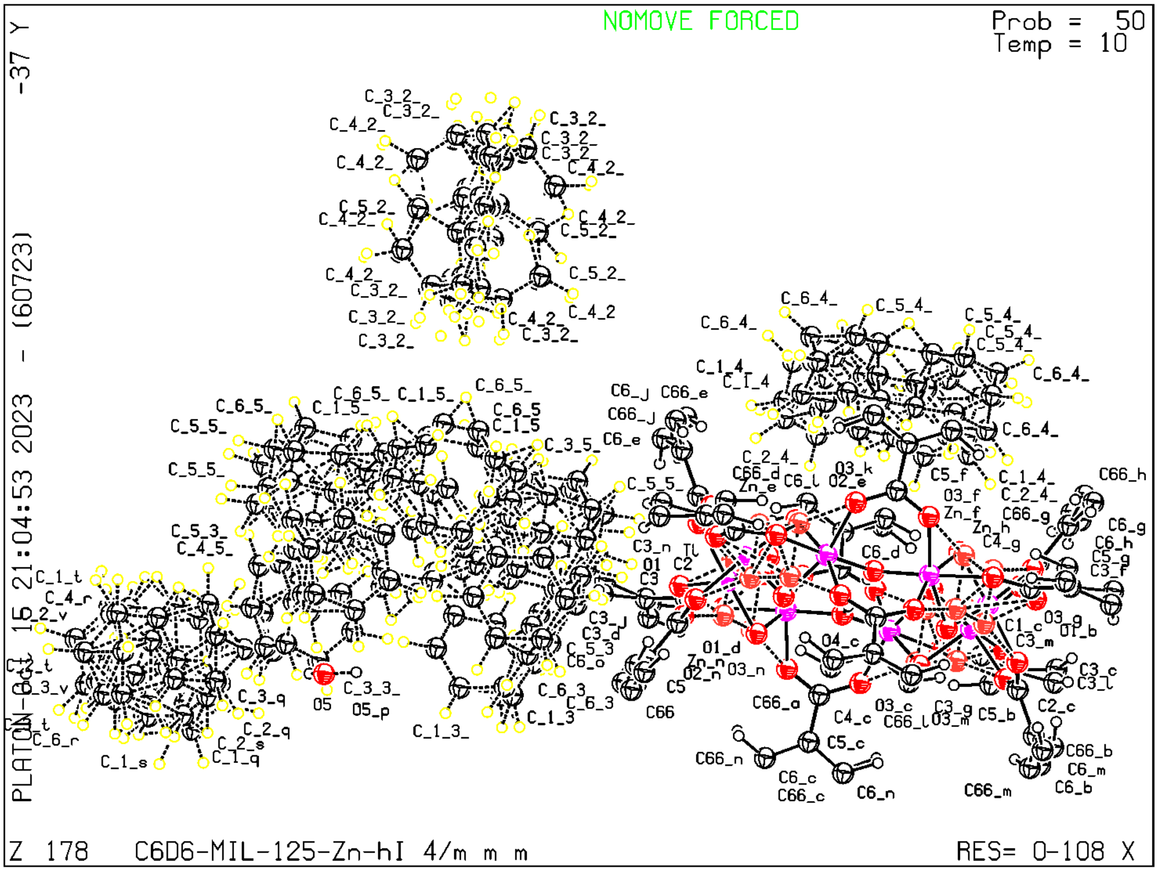

Supplement: Supplementary file 2 — Crystallographic data (11 CIFs) and checkCIF reports. [file 41563_2024_2029_MOESM2_ESM.zip › cifs and check cif reports/C6D6@MIL-125-Zn-high_checkcif.pdf]
