# Supplementary material for: Trace benzene capture by decoration of structural defects in metal–organic framework materials
Source: Nat Mater. 2024 Oct 29;23(11):1531–8. doi: 10.1038/s41563-024-02029-1 (PMC11525167; doi:10.1038/s41563-024-02029-1)

checkCIF/PLATON report

You have not supplied any structure factors. As a result the full set of tests cannot be run.

THIS REPORT IS FOR GUIDANCE ONLY. IF USED AS PART OF A REVIEW PROCEDURE FOR PUBLICATION, IT SHOULD NOT REPLACE THE EXPERTISE OF AN EXPERIENCED CRYSTALLOGRAPHIC REFEREE.

No syntax errors found. CIF dictionary Interpreting this report

Datablock: C6D6-MIL-125-Zn-low

Bond precision: C-C = 0.0055 A Wavelength=0

Cell:	a=18.64607(12)	b=18.64607(12)	c=18.1348(2)
	alpha=90	beta=90	gamma=90
Temperature:	10 K		

	Calculated	Reported
Volume	6305.03(11)	6305.03(11)
Space group	I 4/m m m	I4/mmm
Hall group	-I 4 2	-I 4 2
Moiety formula	C48 H27.48 O34.43 Ti6.95 Zn1.04, 0.024(C96 D96), 0.022(C80 D88)	C56.26 D8.26 H28.52 O35.48 Ti6.95 Zn1.04
Sum formula	C55.90 H28.52 D7.85 O35.48 Ti6.95 Zn1.04	C48 H27.48 O34.43 Ti6.95 Zn1.04, 1.376(C6 D6)
Mr	1684.30	1689.41
Dx,g cm-3	0.887	0.890
Z	2	2
Mu (mm-1)	0.000	0.000
F000	1011.4	0.0
F000'	1679.23	
h,k,lmax		
Nref		
Tmin,Tmax		
Tmin'		

Correction method= Not given

Data completeness= Theta(max)=

R(reflections)= wR2(reflections)=

S = Npar=

The following ALERTS were generated. Each ALERT has the format
test-name_ALERT_alert-type_alert-level.
Click on the hyperlinks for more details of the test.

Alert level A

PLAT770_ALERT_2_A Suspect C-H Bond in CIF: H7 --C_1_3 . 1.31 Ang.

Author Response: The alert could be due to the co-existence of disorder in framework and guest molecules.

PLAT770_ALERT_2_A Suspect C-H Bond in CIF: H7 --C_6_1 . 1.43 Ang.

Author Response: The alert could be due to the co-existence of disorder in framework and guest molecules.

PLAT770_ALERT_2_A Suspect C-H Bond in CIF: H7 --C_6_3 . 1.46 Ang.

Author Response: The alert could be due to the co-existence of disorder in framework and guest molecules.

PLAT770_ALERT_2_A Suspect C-H Bond in CIF: H7 --C_1_3 . 1.46 Ang.

Author Response: The alert could be due to the co-existence of disorder in framework and guest molecules.

PLAT770_ALERT_2_A Suspect C-H Bond in CIF: H4O --C_1_1 . 1.59 Ang.

Author Response: The alert could be due to the co-existence of disorder in framework and guest molecules.

PLAT770_ALERT_2_A Suspect C-H Bond in CIF: H4O --C_1_1 . 1.59 Ang.

Author Response: The alert could be due to the co-existence of disorder in framework and guest molecules.

PLAT770_ALERT_2_A Suspect C-H Bond in CIF: H4O --C_1_1 . 1.59 Ang.

Author Response: The alert could be due to the co-existence of disorder in framework and guest molecules.

PLAT770_ALERT_2_A Suspect C-H Bond in CIF: H4O --C_1_1 . 1.59 Ang.

Author Response: The alert could be due to the co-existence of disorder in framework and guest molecules.

```
PLAT770_ALERT_2_A Suspect C-H Bond in CIF:  H4       --C_3_2     .       1.55 Ang.
```

Author Response: The alert could be due to the co-existence of disorder in framework and guest molecules.

```
PLAT770_ALERT_2_A Suspect C-H Bond in CIF:  H5       --C_4_2     .       1.55 Ang.
```

Author Response: The alert could be due to the co-existence of disorder in framework and guest molecules.

```
PLAT770_ALERT_2_A Suspect C-H Bond in CIF:  H55      --C_4_3     .       1.58 Ang.
```

Author Response: The alert could be due to the co-existence of disorder in framework and guest molecules.

```
PLAT770_ALERT_2_A Suspect C-H Bond in CIF:  C_1_1    --H4O       .       1.59 Ang.
```

Author Response: The alert could be due to the co-existence of disorder in framework and guest molecules.

```
PLAT770_ALERT_2_A Suspect C-H Bond in CIF:  C_6_1    --H7        .       1.43 Ang.
```

Author Response: The alert could be due to the co-existence of disorder in framework and guest molecules.

```
PLAT770_ALERT_2_A Suspect C-H Bond in CIF:  C_3_2    --H4        .       1.55 Ang.
```

Author Response: The alert could be due to the co-existence of disorder in framework and guest molecules.

```
PLAT770_ALERT_2_A Suspect C-H Bond in CIF:  C_4_2    --H5        .       1.55 Ang.
```

Author Response: The alert could be due to the co-existence of disorder in framework and guest molecules.

```
PLAT770_ALERT_2_A Suspect C-H Bond in CIF:  C_1_3    --H7        .       1.31 Ang.
```

Author Response: The alert could be due to the co-existence of disorder in framework and guest molecules.

```
PLAT770_ALERT_2_A Suspect C-H Bond in CIF:  C_1_3    --H7        .       1.46 Ang.
```

Author Response: The alert could be due to the co-existence of disorder in framework and guest molecules.

```
PLAT770_ALERT_2_A Suspect C-H Bond in CIF:  C_4_3    --H55       .       1.58 Ang.
```

Author Response: The alert could be due to the co-existence of disorder in framework and guest molecules.

```
PLAT770_ALERT_2_A Suspect C-H Bond in CIF:  C_6_3    --H7        .       1.46 Ang.
```

Author Response: The alert could be due to the co-existence of disorder in framework and guest molecules.

Alert level B

```
PLAT420_ALERT_2_B D-H Bond Without Acceptor  O4       --H4O      .     Please Check
```

Author Response: The guest molecule benzenen does not have a hydrogen bonding acceptor.

Alert level C

```
CELLK01_ALERT_1_C  Check that the cell measurement temperature is in Kelvin.
            Value of measurement temperature given =    10.000
```

Author Response: The structure is obtained from neutron powder diffraction using cold neutron source at 10K from ISIS WISH beamline.

```
CHEMW01_ALERT_1_C  The ratio of given/expected molecular weight as calculated
            from the _chemical_formula_sum lies outside
            the range 0.99 <> 1.01
            Calculated formula weight =  1671.7751
            Formula weight given      =  1689.4100
```

Author Response: The issues is caused by checkcif algorithm ignoring a unique molecule in the structure with the naming style as X_Y, where X is the element and Y is the internal atom index.

```
CHEMW01_ALERT_1_C  The difference between the given and expected weight for
            compound is greater 1 mass unit. Check that all hydrogen
            atoms have been taken into account.
```

Author Response: The issues is caused by checkcif algorithm ignoring a unique molecule in the structure with the naming style as X_Y, where X is the element and Y is the internal atom index.

```
PLAT041_ALERT_1_C Calc. and Reported SumFormula    Strings  Differ     Please Check
```

Author Response: The issues is caused by checkcif algorithm ignoring a unique molecule in the structure with the naming style as X_Y, where X is the element and Y is the internal atom index.

```
PLAT042_ALERT_1_C Calc. and Reported MoietyFormula Strings  Differ     Please Check
```

Author Response: The issues is caused by checkcif algorithm ignoring a unique molecule in the structure with the naming style as X_Y, where X is the element and Y is the internal atom index.

```
PLAT043_ALERT_1_C Calculated and Reported Mol. Weight Differ by ..       5.11 Check
```

Author Response: The issues is caused by checkcif algorithm ignoring a unique molecule in the structure with the naming style as X_Y, where X is the element and Y is the internal atom index.

```
PLAT077_ALERT_4_C Unitcell Contains Non-integer Number of Atoms ..     Please Check
```

Author Response: The structure contains disordered framework and guest molecules.

```
PLAT601_ALERT_2_C Unit Cell Contains Solvent Accessible VOIDS of .         41 Ang**3
```

Author Response: The void is likely inaccessible to the guest benzene molecule.

Alert level G

```
FORMU01_ALERT_1_G  There is a discrepancy between the atom counts in the
            _chemical_formula_sum and _chemical_formula_moiety. This is
            usually due to the moiety formula being in the wrong format.
            Atom count from _chemical_formula_sum:   C56.25600 H27.48 D8.256 O34.4
            Atom count from _chemical_formula_moiety:C56.26 H28.52 D8.26 O35.48 Ti
FORMU01_ALERT_2_G  There is a discrepancy between the atom counts in the
            _chemical_formula_sum and the formula from the _atom_site* data.
            Atom count from _chemical_formula_sum:C56.25600 H27.48 D8.256 O34.43 T
            Atom count from the _atom_site data:  C56.25601 H28.51599 D8.024 O35.4
CELLZ01_ALERT_1_G Difference between formula and atom_site contents detected.
CELLZ01_ALERT_1_G ALERT: Large difference may be due to a
            symmetry error - see SYMMG tests
           From the CIF: _cell_formula_units_Z    2
           From the CIF: _chemical_formula_sum  C48 H27.48 O34.43 Ti6.95 Zn1.04, 1
```

TEST: Compare cell contents of formula and atom_site data

atom	Z*formula	cif sites	diff
C	96.00	112.51	-16.51
H	54.96	57.03	-2.07
O	68.86	70.95	-2.09
Ti	13.90	13.90	-0.00
Zn	2.08	2.08	0.00
,	2.75	0.00	2.75
(C	12.00	0.00	12.00
D	12.00	16.05	-4.05
)	2.00	0.00	2.00

```
PLAT049_ALERT_1_G Calculated Density Less Than 1.0 gcm-3 .........     0.8872 Check
PLAT300_ALERT_4_G Atom Site Occupancy of C3         Constrained at        0.5 Check
PLAT300_ALERT_4_G Atom Site Occupancy of C6         Constrained at        0.5 Check
PLAT300_ALERT_4_G Atom Site Occupancy of C66        Constrained at        0.5 Check
PLAT300_ALERT_4_G Atom Site Occupancy of H4         Constrained at        0.5 Check
PLAT300_ALERT_4_G Atom Site Occupancy of H5         Constrained at        0.5 Check
PLAT300_ALERT_4_G Atom Site Occupancy of H55        Constrained at        0.5 Check
PLAT301_ALERT_3_G Main Residue  Disorder .............(Resd  1  )        58% Note
PLAT301_ALERT_3_G Main Residue  Disorder .............(Resd  2  )       100% Note
PLAT301_ALERT_3_G Main Residue  Disorder .............(Resd  3  )       100% Note
PLAT302_ALERT_4_G Anion/Solvent/Minor-Residue Disorder (Resd  4  )       100% Note
PLAT302_ALERT_4_G Anion/Solvent/Minor-Residue Disorder (Resd  5  )       100% Note
PLAT302_ALERT_4_G Anion/Solvent/Minor-Residue Disorder (Resd  6  )       100% Note
PLAT304_ALERT_4_G Non-Integer Number of Atoms in ..... (Resd  1  )     117.90 Check
PLAT304_ALERT_4_G Non-Integer Number of Atoms in ..... (Resd  2  )       4.53 Check
PLAT304_ALERT_4_G Non-Integer Number of Atoms in ..... (Resd  3  )       3.73 Check
PLAT304_ALERT_4_G Non-Integer Number of Atoms in ..... (Resd  4  )       1.28 Check
PLAT304_ALERT_4_G Non-Integer Number of Atoms in ..... (Resd  5  )       1.23 Check
PLAT304_ALERT_4_G Non-Integer Number of Atoms in ..... (Resd  6  )       0.07 Check
PLAT304_ALERT_4_G Non-Integer Number of Atoms in ..... (Resd  7  )       0.06 Check
PLAT311_ALERT_2_G Isolated Disordered Oxygen Atom (No H's ?) .....         O5 Check
PLAT432_ALERT_2_G Short Inter X...Y Contact  O1       ..C_4_2    .       2.61 Ang.
                                      -1/2+y,1/2-x,1/2+z  =     26_455 Check
PLAT432_ALERT_2_G Short Inter X...Y Contact  O1       ..C_4_2    .       2.61 Ang.
                                      -1/2+y,1/2-x,1/2-z  =     25_455 Check
PLAT432_ALERT_2_G Short Inter X...Y Contact  O1       ..C_5_2    .       2.82 Ang.
                                      -1/2+y,1/2-x,1/2+z  =     26_455 Check
PLAT432_ALERT_2_G Short Inter X...Y Contact  O1       ..C_5_2    .       2.82 Ang.
                                      -1/2+y,1/2-x,1/2-z  =     25_455 Check
PLAT432_ALERT_2_G Short Inter X...Y Contact  O3       ..C_1_1    .       2.17 Ang.
                                                   y,x,z  =     12_555 Check
PLAT432_ALERT_2_G Short Inter X...Y Contact  O3       ..C_5_2    .       2.27 Ang.
                                      -1/2+y,1/2-x,1/2-z  =     25_455 Check
PLAT432_ALERT_2_G Short Inter X...Y Contact  O3       ..C_4_2    .       2.33 Ang.
                                      -1/2+y,1/2-x,1/2-z  =     25_455 Check
PLAT432_ALERT_2_G Short Inter X...Y Contact  O3       ..C_6_1    .       2.49 Ang.
                                                   y,x,z  =     12_555 Check
PLAT432_ALERT_2_G Short Inter X...Y Contact  O3       ..C_1_1    .       2.61 Ang.
                                                   x,y,z  =      1_555 Check
PLAT432_ALERT_2_G Short Inter X...Y Contact  O3       ..C_6_1    .       2.88 Ang.
                                                   x,y,z  =      1_555 Check
PLAT432_ALERT_2_G Short Inter X...Y Contact  O3       ..C_3      .       2.99 Ang.
                                      -1/2+x,1/2-y,1/2-z  =     29_455 Check
PLAT432_ALERT_2_G Short Inter X...Y Contact  O3       ..C_1_1    .       3.02 Ang.
                                                 y,x,1-z  =     11_556 Check
```

```
PLAT432_ALERT_2_G Short Inter X...Y Contact  C2        ..C3        .       2.45 Ang.
                                               1-x,y,1-z  =      3_656 Check
PLAT432_ALERT_2_G Short Inter X...Y Contact  C2        ..C3        .       2.45 Ang.
                                                 1-x,y,z  =      4_655 Check
PLAT432_ALERT_2_G Short Inter X...Y Contact  C2        ..C3        .       2.45 Ang.
                                                1-x,-y,z  =      2_655 Check
PLAT432_ALERT_2_G Short Inter X...Y Contact  C2        ..C3        .       2.45 Ang.
                                              1-x,-y,1-z  =     16_656 Check
PLAT432_ALERT_2_G Short Inter X...Y Contact  C2        ..C2        .       2.83 Ang.
                                                1-x,-y,z  =      2_655 Check
PLAT432_ALERT_2_G Short Inter X...Y Contact  C2        ..C_2_2     .       3.02 Ang.
                                    -1/2+y,-1/2+x,1/2-z  =     27_445 Check
PLAT432_ALERT_2_G Short Inter X...Y Contact  C2        ..C_2_2     .       3.02 Ang.
                                    -1/2+y,-1/2+x,1/2+z  =     28_445 Check
PLAT432_ALERT_2_G Short Inter X...Y Contact  C2        ..C_2_2     .       3.02 Ang.
                                     -1/2+y,1/2-x,1/2+z  =     26_455 Check
PLAT432_ALERT_2_G Short Inter X...Y Contact  C2        ..C_2_2     .       3.02 Ang.
                                     -1/2+y,1/2-x,1/2-z  =     25_455 Check
PLAT432_ALERT_2_G Short Inter X...Y Contact  C2        ..C_3_2     .       3.14 Ang.
                                     -1/2+y,1/2-x,1/2+z  =     26_455 Check
PLAT432_ALERT_2_G Short Inter X...Y Contact  C2        ..C_3_2     .       3.14 Ang.
                                    -1/2+y,-1/2+x,1/2+z  =     28_445 Check
PLAT432_ALERT_2_G Short Inter X...Y Contact  C2        ..C_3_2     .       3.14 Ang.
                                    -1/2+y,-1/2+x,1/2-z  =     27_445 Check
PLAT432_ALERT_2_G Short Inter X...Y Contact  C2        ..C_3_2     .       3.14 Ang.
                                     -1/2+y,1/2-x,1/2-z  =     25_455 Check
PLAT432_ALERT_2_G Short Inter X...Y Contact  C3        ..C3        .       1.41 Ang.
                                                 1-x,y,z  =      4_655 Check
PLAT432_ALERT_2_G Short Inter X...Y Contact  C3        ..C3        .       1.46 Ang.
                                               1-x,y,1-z  =      3_656 Check
PLAT432_ALERT_2_G Short Inter X...Y Contact  C3        ..C3        .       2.82 Ang.
                                                1-x,-y,z  =      2_655 Check
PLAT432_ALERT_2_G Short Inter X...Y Contact  C3        ..C3        .       2.84 Ang.
                                              1-x,-y,1-z  =     16_656 Check
PLAT432_ALERT_2_G Short Inter X...Y Contact  C4        ..C_1_1     .       2.60 Ang.
                                                   y,x,z  =     12_555 Check
PLAT432_ALERT_2_G Short Inter X...Y Contact  C4        ..C_1_1     .       2.60 Ang.
                                                   x,y,z  =      1_555 Check
PLAT432_ALERT_2_G Short Inter X...Y Contact  C4        ..C_6_1     .       2.63 Ang.
                                                   y,x,z  =     12_555 Check
PLAT432_ALERT_2_G Short Inter X...Y Contact  C4        ..C_6_1     .       2.63 Ang.
                                                   x,y,z  =      1_555 Check
PLAT432_ALERT_2_G Short Inter X...Y Contact  C4        ..C_3       .       2.72 Ang.
                                     1/2-y,-1/2+x,1/2-z  =     23_545 Check
PLAT432_ALERT_2_G Short Inter X...Y Contact  C4        ..C_3       .       2.72 Ang.
                                     -1/2+x,1/2-y,1/2-z  =     29_455 Check
PLAT432_ALERT_2_G Short Inter X...Y Contact  C4        ..C_5_3     .       2.79 Ang.
                                      1/2-y,1/2-x,1/2-z  =     21_555 Check
PLAT432_ALERT_2_G Short Inter X...Y Contact  C4        ..C_5_3     .       2.79 Ang.
                                      1/2-x,1/2-y,1/2-z  =     32_555 Check
PLAT432_ALERT_2_G Short Inter X...Y Contact  C4        ..C_5_2     .       3.04 Ang.
                                     1/2-x,-1/2+y,1/2-z  =     19_545 Check
PLAT432_ALERT_2_G Short Inter X...Y Contact  C4        ..C_5_2     .       3.04 Ang.
                                     -1/2+y,1/2-x,1/2-z  =     25_455 Check
PLAT432_ALERT_2_G Short Inter X...Y Contact  C4        ..C_4_2     .       3.05 Ang.
                                     1/2-x,-1/2+y,1/2-z  =     19_545 Check
PLAT432_ALERT_2_G Short Inter X...Y Contact  C4        ..C_4_2     .       3.05 Ang.
```

```
                                          -1/2+y,1/2-x,1/2-z  =     25_455 Check
PLAT432_ALERT_2_G Short Inter X...Y Contact  C4       ..C_6_3    .       3.16 Ang.
                                           1/2-y,1/2-x,1/2-z  =     21_555 Check
PLAT432_ALERT_2_G Short Inter X...Y Contact  C4       ..C_6_3    .       3.16 Ang.
                                           1/2-x,1/2-y,1/2-z  =     32_555 Check
PLAT432_ALERT_2_G Short Inter X...Y Contact  C5       ..C_5_3    .       2.18 Ang.
                                           1/2-y,1/2-x,1/2-z  =     21_555 Check
PLAT432_ALERT_2_G Short Inter X...Y Contact  C5       ..C_5_3    .       2.18 Ang.
                                           1/2-x,1/2-y,1/2-z  =     32_555 Check
PLAT432_ALERT_2_G Short Inter X...Y Contact  C5       ..C66      .       2.42 Ang.
                                           1/2-x,1/2-y,1/2-z  =     32_555 Check
PLAT432_ALERT_2_G Short Inter X...Y Contact  C5       ..C66      .       2.42 Ang.
                                           1/2-y,1/2-x,1/2-z  =     21_555 Check
PLAT432_ALERT_2_G Short Inter X...Y Contact  C5       ..C6       .       2.42 Ang.
                                           1/2-y,1/2-x,1/2-z  =     21_555 Check
PLAT432_ALERT_2_G Short Inter X...Y Contact  C5       ..C6       .       2.42 Ang.
                                           1/2-x,1/2-y,1/2-z  =     32_555 Check
PLAT432_ALERT_2_G Short Inter X...Y Contact  C5       ..C_3      .       2.74 Ang.
                                          1/2-y,-1/2+x,1/2-z  =     23_545 Check
PLAT432_ALERT_2_G Short Inter X...Y Contact  C5       ..C_3      .       2.74 Ang.
                                          -1/2+x,1/2-y,1/2-z  =     29_455 Check
PLAT432_ALERT_2_G Short Inter X...Y Contact  C5       ..C5       .       2.80 Ang.
                                           1/2-y,1/2-x,1/2-z  =     21_555 Check
PLAT432_ALERT_2_G Short Inter X...Y Contact  C5       ..C_4_3    .       3.00 Ang.
                                           1/2-y,1/2-x,1/2-z  =     21_555 Check
PLAT432_ALERT_2_G Short Inter X...Y Contact  C5       ..C_4_3    .       3.00 Ang.
                                           1/2-x,1/2-y,1/2-z  =     32_555 Check
PLAT432_ALERT_2_G Short Inter X...Y Contact  C5       ..C_5_2    .       3.04 Ang.
                                          1/2-x,-1/2+y,1/2-z  =     19_545 Check
PLAT432_ALERT_2_G Short Inter X...Y Contact  C5       ..C_5_2    .       3.04 Ang.
                                          -1/2+y,1/2-x,1/2-z  =     25_455 Check
PLAT432_ALERT_2_G Short Inter X...Y Contact  C5       ..C_6_3    .       3.11 Ang.
                                           1/2-y,1/2-x,1/2-z  =     21_555 Check
PLAT432_ALERT_2_G Short Inter X...Y Contact  C5       ..C_6_3    .       3.11 Ang.
                                           1/2-x,1/2-y,1/2-z  =     32_555 Check
PLAT432_ALERT_2_G Short Inter X...Y Contact  C5       ..C_4_2    .       3.14 Ang.
                                          -1/2+y,1/2-x,1/2-z  =     25_455 Check
PLAT432_ALERT_2_G Short Inter X...Y Contact  C5       ..C_4_2    .       3.14 Ang.
                                          1/2-x,-1/2+y,1/2-z  =     19_545 Check
PLAT432_ALERT_2_G Short Inter X...Y Contact  C6       ..C66      .       1.38 Ang.
                                           1/2-x,1/2-y,1/2-z  =     32_555 Check
PLAT432_ALERT_2_G Short Inter X...Y Contact  C6       ..C6       .       1.40 Ang.
                                           1/2-y,1/2-x,1/2-z  =     21_555 Check
PLAT432_ALERT_2_G Short Inter X...Y Contact  C6       ..C66      .       2.81 Ang.
                                           1/2-y,1/2-x,1/2-z  =     21_555 Check
PLAT432_ALERT_2_G Short Inter X...Y Contact  C6       ..C6       .       2.82 Ang.
                                           1/2-x,1/2-y,1/2-z  =     32_555 Check
PLAT432_ALERT_2_G Short Inter X...Y Contact  C66      ..C66      .       1.39 Ang.
                                           1/2-y,1/2-x,1/2-z  =     21_555 Check
PLAT432_ALERT_2_G Short Inter X...Y Contact  C66      ..C66      .       2.82 Ang.
                                           1/2-x,1/2-y,1/2-z  =     32_555 Check
PLAT432_ALERT_2_G Short Inter X...Y Contact  C_1_2    ..C_1_2    .       2.73 Ang.
                                          -1/2+y,1/2+x,1/2-z  =     27_455 Check
PLAT720_ALERT_4_G Number of Unusual/Non-Standard Labels .........         48 Note
PLAT764_ALERT_4_G Overcomplete CIF Bond List Detected (Rep/Expd) .       2.63 Ratio
PLAT773_ALERT_2_G Check long C-C Bond in CIF: C6       --C_5_3           2.03 Ang.
PLAT773_ALERT_2_G Check long C-C Bond in CIF: C66      --C_5_3           1.80 Ang.
```

```
PLAT773_ALERT_2_G Check long C-C Bond in CIF: C66      --C_4_3           1.89 Ang.
PLAT773_ALERT_2_G Check long C-C Bond in CIF: C_1      --C_2             1.74 Ang.
PLAT773_ALERT_2_G Check long C-C Bond in CIF: C_1      --C_4             1.94 Ang.
PLAT773_ALERT_2_G Check long C-C Bond in CIF: C_2      --C_1             1.74 Ang.
PLAT773_ALERT_2_G Check long C-C Bond in CIF: C_2      --C_2             1.75 Ang.
PLAT773_ALERT_2_G Check long C-C Bond in CIF: C_4      --C_1             1.94 Ang.
PLAT773_ALERT_2_G Check long C-C Bond in CIF: C_4      --C_6             1.94 Ang.
PLAT773_ALERT_2_G Check long C-C Bond in CIF: C_6      --C_4             1.94 Ang.
PLAT773_ALERT_2_G Check long C-C Bond in CIF: C_1_1    --C_1_1           1.78 Ang.
PLAT773_ALERT_2_G Check long C-C Bond in CIF: C_2_1    --C_6_1           2.06 Ang.
PLAT773_ALERT_2_G Check long C-C Bond in CIF: C_3_1    --C_4_1           1.73 Ang.
PLAT773_ALERT_2_G Check long C-C Bond in CIF: C_3_1    --C_4_1           1.73 Ang.
PLAT773_ALERT_2_G Check long C-C Bond in CIF: C_3_1    --C_4_1           1.79 Ang.
PLAT773_ALERT_2_G Check long C-C Bond in CIF: C_3_1    --C_4_1           1.79 Ang.
PLAT773_ALERT_2_G Check long C-C Bond in CIF: C_3_1    --C_4_1           1.90 Ang.
PLAT773_ALERT_2_G Check long C-C Bond in CIF: C_3_1    --C_4_1           1.91 Ang.
PLAT773_ALERT_2_G Check long C-C Bond in CIF: C_3_1    --C_4_1           1.93 Ang.
PLAT773_ALERT_2_G Check long C-C Bond in CIF: C_3_1    --C_4_1           1.94 Ang.
PLAT773_ALERT_2_G Check long C-C Bond in CIF: C_3_1    --C_4_1           2.04 Ang.
PLAT773_ALERT_2_G Check long C-C Bond in CIF: C_3_1    --C_4_1           2.04 Ang.
PLAT773_ALERT_2_G Check long C-C Bond in CIF: C_4_1    --C_5_1           1.71 Ang.
PLAT773_ALERT_2_G Check long C-C Bond in CIF: C_4_1    --C_3_1           1.73 Ang.
PLAT773_ALERT_2_G Check long C-C Bond in CIF: C_4_1    --C_3_1           1.73 Ang.
PLAT773_ALERT_2_G Check long C-C Bond in CIF: C_4_1    --C_5_1           1.75 Ang.
PLAT773_ALERT_2_G Check long C-C Bond in CIF: C_4_1    --C_3_1           1.79 Ang.
PLAT773_ALERT_2_G Check long C-C Bond in CIF: C_4_1    --C_3_1           1.79 Ang.
PLAT773_ALERT_2_G Check long C-C Bond in CIF: C_4_1    --C_5_1           1.82 Ang.
PLAT773_ALERT_2_G Check long C-C Bond in CIF: C_4_1    --C_3_1           1.90 Ang.
PLAT773_ALERT_2_G Check long C-C Bond in CIF: C_4_1    --C_3_1           1.91 Ang.
PLAT773_ALERT_2_G Check long C-C Bond in CIF: C_4_1    --C_3_1           1.93 Ang.
PLAT773_ALERT_2_G Check long C-C Bond in CIF: C_4_1    --C_3_1           1.94 Ang.
PLAT773_ALERT_2_G Check long C-C Bond in CIF: C_4_1    --C_5_1           1.99 Ang.
PLAT773_ALERT_2_G Check long C-C Bond in CIF: C_4_1    --C_5_1           2.00 Ang.
PLAT773_ALERT_2_G Check long C-C Bond in CIF: C_4_1    --C_3_1           2.04 Ang.
PLAT773_ALERT_2_G Check long C-C Bond in CIF: C_4_1    --C_3_1           2.04 Ang.
PLAT773_ALERT_2_G Check long C-C Bond in CIF: C_5_1    --C_4_1           1.71 Ang.
PLAT773_ALERT_2_G Check long C-C Bond in CIF: C_5_1    --C_4_1           1.75 Ang.
PLAT773_ALERT_2_G Check long C-C Bond in CIF: C_5_1    --C_4_1           1.82 Ang.
PLAT773_ALERT_2_G Check long C-C Bond in CIF: C_5_1    --C_5_1           1.96 Ang.
PLAT773_ALERT_2_G Check long C-C Bond in CIF: C_5_1    --C_5_1           1.96 Ang.
PLAT773_ALERT_2_G Check long C-C Bond in CIF: C_5_1    --C_4_1           1.99 Ang.
PLAT773_ALERT_2_G Check long C-C Bond in CIF: C_5_1    --C_4_1           2.00 Ang.
PLAT773_ALERT_2_G Check long C-C Bond in CIF: C_6_1    --C_6_3           2.02 Ang.
PLAT773_ALERT_2_G Check long C-C Bond in CIF: C_6_1    --C_2_1           2.06 Ang.
PLAT773_ALERT_2_G Check long C-C Bond in CIF: C_1_2    --C_6_2           1.92 Ang.
PLAT773_ALERT_2_G Check long C-C Bond in CIF: C_6_2    --C_1_2           1.92 Ang.
PLAT773_ALERT_2_G Check long C-C Bond in CIF: C_6_2    --C_3_3           1.94 Ang.
PLAT773_ALERT_2_G Check long C-C Bond in CIF: C_1_3    --C_6_3           1.72 Ang.
PLAT773_ALERT_2_G Check long C-C Bond in CIF: C_2_3    --C_6_3           1.82 Ang.
PLAT773_ALERT_2_G Check long C-C Bond in CIF: C_2_3    --C_5_3           1.97 Ang.
PLAT773_ALERT_2_G Check long C-C Bond in CIF: C_3_3    --C_6_2           1.94 Ang.
PLAT773_ALERT_2_G Check long C-C Bond in CIF: C_4_3    --C66             1.89 Ang.
PLAT773_ALERT_2_G Check long C-C Bond in CIF: C_5_3    --C66             1.80 Ang.
PLAT773_ALERT_2_G Check long C-C Bond in CIF: C_5_3    --C_2_3           1.97 Ang.
PLAT773_ALERT_2_G Check long C-C Bond in CIF: C_5_3    --C6              2.03 Ang.
PLAT773_ALERT_2_G Check long C-C Bond in CIF: C_6_3    --C_1_3           1.72 Ang.
PLAT773_ALERT_2_G Check long C-C Bond in CIF: C_6_3    --C_6_3           1.74 Ang.
```

```
PLAT773_ALERT_2_G Check long C-C Bond in CIF: C_6_3    --C_2_3          1.82 Ang.
PLAT773_ALERT_2_G Check long C-C Bond in CIF: C_6_3    --C_6_1          2.02 Ang.
PLAT811_ALERT_5_G No ADDSYM Analysis: Too Many Excluded Atoms ....         ! Info
```

```
 19 ALERT level A = Most likely a serious problem - resolve or explain
  1 ALERT level B = A potentially serious problem, consider carefully
  8 ALERT level C = Check. Ensure it is not caused by an omission or oversight
156 ALERT level G = General information/check it is not something unexpected

 10 ALERT type 1 CIF construction/syntax error, inconsistent or missing data
151 ALERT type 2 Indicator that the structure model may be wrong or deficient
  3 ALERT type 3 Indicator that the structure quality may be low
 19 ALERT type 4 Improvement, methodology, query or suggestion
  1 ALERT type 5 Informative message, check
```

It is advisable to attempt to resolve as many as possible of the alerts in all categories. Often the minor alerts point to easily fixed oversights, errors and omissions in your CIF or refinement strategy, so attention to these fine details can be worthwhile. In order to resolve some of the more serious problems it may be necessary to carry out additional measurements or structure refinements. However, the purpose of your study may justify the reported deviations and the more serious of these should normally be commented upon in the discussion or experimental section of a paper or in the "special_details" fields of the CIF. checkCIF was carefully designed to identify outliers and unusual parameters, but every test has its limitations and alerts that are not important in a particular case may appear. Conversely, the absence of alerts does not guarantee there are no aspects of the results needing attention. It is up to the individual to critically assess their own results and, if necessary, seek expert advice.

Publication of your CIF in IUCr journals

A basic structural check has been run on your CIF. These basic checks will be run on all CIFs submitted for publication in IUCr journals (*Acta Crystallographica*, *Journal of Applied Crystallography*, *Journal of Synchrotron Radiation*); however, if you intend to submit to *Acta Crystallographica Section C* or *E* or *IUCrData*, you should make sure that full publication checks are run on the final version of your CIF prior to submission.

Publication of your CIF in other journals

Please refer to the *Notes for Authors* of the relevant journal for any special instructions relating to CIF submission.

PLATON version of 06/07/2023; check.def file version of 30/06/2023

Datablock C6D6-MIL-125-Zn-low - ellipsoid plot

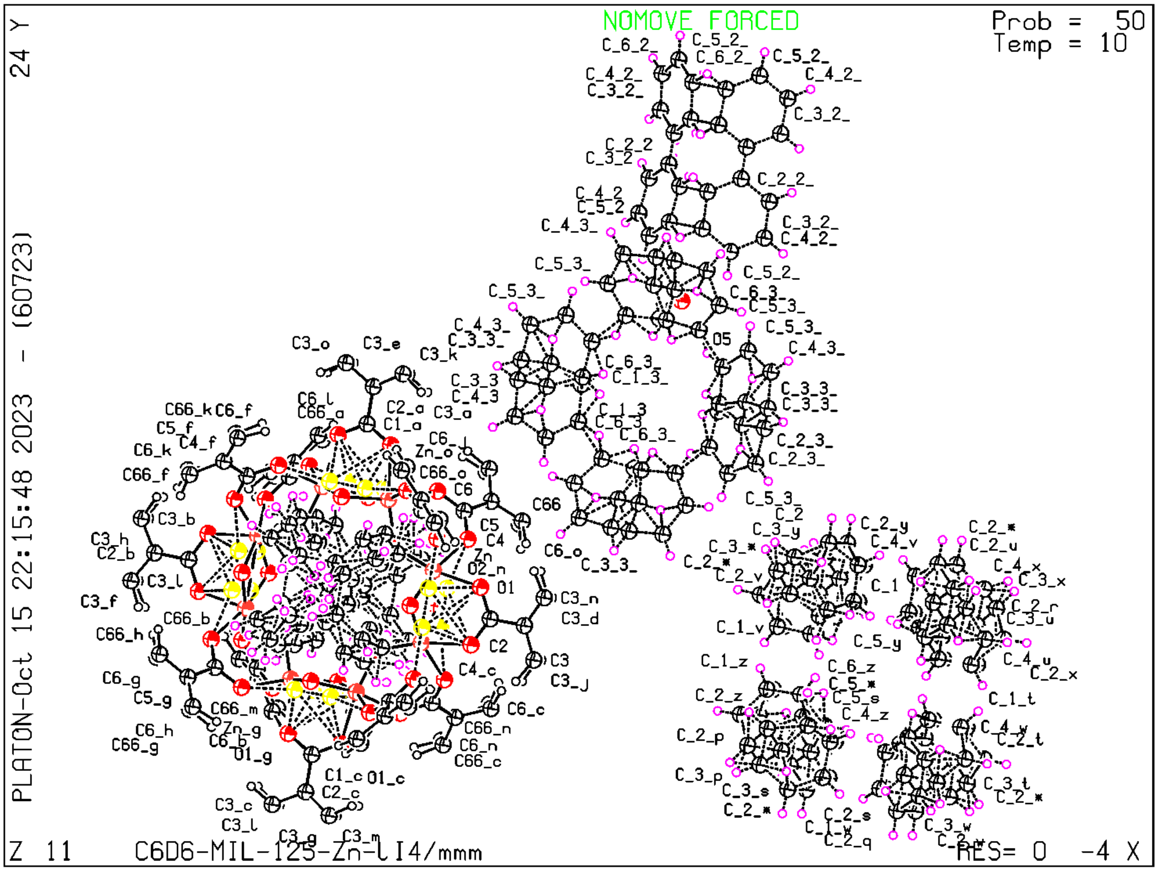

Supplement: Supplementary file 2 — Crystallographic data (11 CIFs) and checkCIF reports. [file 41563_2024_2029_MOESM2_ESM.zip › cifs and check cif reports/C6D6@MIL-125-Zn-low_checkcif.pdf]
