# Supplementary material for: Trace benzene capture by decoration of structural defects in metal–organic framework materials
Source: Nat Mater. 2024 Oct 29;23(11):1531–8. doi: 10.1038/s41563-024-02029-1 (PMC11525167; doi:10.1038/s41563-024-02029-1)

checkCIF/PLATON report

You have not supplied any structure factors. As a result the full set of tests cannot be run.

THIS REPORT IS FOR GUIDANCE ONLY. IF USED AS PART OF A REVIEW PROCEDURE FOR PUBLICATION, IT SHOULD NOT REPLACE THE EXPERTISE OF AN EXPERIENCED CRYSTALLOGRAPHIC REFEREE.

No syntax errors found.   CIF dictionary   Interpreting this report

Datablock: C6D6-MIL-125

```
Bond precision:   C-C = 0.0140 A          Wavelength=0

Cell:              a=18.6015(9)      b=18.6015(9)      c=18.1692(9)
                   alpha=90          beta=90           gamma=90
Temperature:       10 K
```

	Calculated	Reported
Volume	6286.8(7)	6286.8(7)
Space group	I 4/m m m	I4/mmm
Hall group	-I 4 2	-I 4 2
Moiety formula	2(C6 H3.29 D0.45 O4.50 Ti), 0.137(C48 D48), 0.06(C48), 0.069(C2	2(C6 H3.29 D0.45 O4.50 Ti), 1.72(C6 D6) 0.08(C6 H6)
Sum formula	C23.06 H7 D11.07 O9 Ti2	C23.06 H7 D11.07 O9 Ti2
Mr	546.03	546.09
Dx,g cm-3	1.154	1.154
Z	8	8
Mu (mm-1)	0.000	0.000
F000	1972.0	0.0
F000'	2179.34	
h,k,lmax		
Nref		
Tmin,Tmax		
Tmin'		

```
Correction method= Not given

Data completeness=                   Theta(max)=

R(reflections)=                                       wR2(reflections)=
S =                          Npar=
```

The following ALERTS were generated. Each ALERT has the format
test-name_ALERT_alert-type_alert-level.
Click on the hyperlinks for more details of the test.

Alert level A

PLAT770_ALERT_2_A Suspect C-H Bond in CIF: C_2_1 --H_5_1_H . 1.51 Ang.

Author Response: The close contact of this alert could be due to the disordered guest molecules within the framework.

PLAT770_ALERT_2_A Suspect C-H Bond in CIF: C_3_1 --H_3_1_H . 1.46 Ang.

Author Response: The close contact of this alert could be due to the disordered guest molecules within the framework.

PLAT770_ALERT_2_A Suspect C-H Bond in CIF: C_4_1 --H_4_1_H . 1.32 Ang.

Author Response: The close contact of this alert could be due to the disordered guest molecules within the framework.

PLAT770_ALERT_2_A Suspect C-H Bond in CIF: C_4_1 --H_5_1_H . 1.36 Ang.

Author Response: The close contact of this alert could be due to the disordered guest molecules within the framework.

PLAT770_ALERT_2_A Suspect C-H Bond in CIF: C_4_1 --H_4_1_H . 1.37 Ang.

Author Response: The close contact of this alert could be due to the disordered guest molecules within the framework.

PLAT770_ALERT_2_A Suspect C-H Bond in CIF: C_4_1 --H_5_1_H . 1.46 Ang.

Author Response: The close contact of this alert could be due to the disordered guest molecules within the framework.

PLAT770_ALERT_2_A Suspect C-H Bond in CIF: C_4_1 --H_4_1_H . 1.49 Ang.

Author Response: The close contact of this alert could be due to the disordered guest molecules within the framework.

PLAT770_ALERT_2_A Suspect C-H Bond in CIF: C_4_1 --H_4_1_H . 1.54 Ang.

Author Response: The close contact of this alert could be due to the disordered guest molecules within the framework.

```
PLAT770_ALERT_2_A Suspect C-H Bond in CIF:  H_1_1_H  --C_5_2     .       1.48 Ang.
```

Author Response: The close contact of this alert could be due to the disordered guest molecules within the framework.

```
PLAT770_ALERT_2_A Suspect C-H Bond in CIF:  H_2_1_H  --C_5_2     .       1.62 Ang.
```

Author Response: The close contact of this alert could be due to the disordered guest molecules within the framework.

```
PLAT770_ALERT_2_A Suspect C-H Bond in CIF:  H_3_1_H  --C_3_1     .       1.46 Ang.
```

Author Response: The close contact of this alert could be due to the disordered guest molecules within the framework.

```
PLAT770_ALERT_2_A Suspect C-H Bond in CIF:  H_4_1_H  --C_4_1     .       1.32 Ang.
```

Author Response: The close contact of this alert could be due to the disordered guest molecules within the framework.

```
PLAT770_ALERT_2_A Suspect C-H Bond in CIF:  H_4_1_H  --C_4_1     .       1.37 Ang.
```

Author Response: The close contact of this alert could be due to the disordered guest molecules within the framework.

```
PLAT770_ALERT_2_A Suspect C-H Bond in CIF:  H_4_1_H  --C_4_1     .       1.49 Ang.
```

Author Response: The close contact of this alert could be due to the disordered guest molecules within the framework.

```
PLAT770_ALERT_2_A Suspect C-H Bond in CIF:  H_4_1_H  --C_4_1     .       1.54 Ang.
```

Author Response: The close contact of this alert could be due to the disordered guest molecules within the framework.

```
PLAT770_ALERT_2_A Suspect C-H Bond in CIF:  H_5_1_H  --C_4_1     .       1.36 Ang.
```

Author Response: The close contact of this alert could be due to the disordered guest molecules within the framework.

PLAT770_ALERT_2_A Suspect C-H Bond in CIF: H_5_1_H --C_4_1 . 1.46 Ang.

Author Response: The close contact of this alert could be due to the disordered guest molecules within the framework.

PLAT770_ALERT_2_A Suspect C-H Bond in CIF: H_5_1_H --C_2_1 . 1.51 Ang.

Author Response: The close contact of this alert could be due to the disordered guest molecules within the framework.

PLAT770_ALERT_2_A Suspect C-H Bond in CIF: H_6_1_H --C_5_2 . 1.31 Ang.

Author Response: The close contact of this alert could be due to the disordered guest molecules within the framework.

PLAT770_ALERT_2_A Suspect C-H Bond in CIF: C_5_2 --H_6_1_H . 1.31 Ang.

Author Response: The close contact of this alert could be due to the disordered guest molecules within the framework.

PLAT770_ALERT_2_A Suspect C-H Bond in CIF: C_5_2 --H_1_1_H . 1.48 Ang.

Author Response: The close contact of this alert could be due to the disordered guest molecules within the framework.

PLAT770_ALERT_2_A Suspect C-H Bond in CIF: C_5_2 --H_2_1_H . 1.62 Ang.

Author Response: The close contact of this alert could be due to the disordered guest molecules within the framework.

Alert level B

PLAT420_ALERT_2_B D-H Bond Without Acceptor O4 --H4O_D . Please Check

Author Response: The guest molecule benzenen does not have a hydrogen bonding acceptor.

PLAT772_ALERT_2_B Suspect O-H Bond in CIF: O4 --H_3_1_H .. 1.45 Ang.

Author Response: The alert could be due to the co-existence of disorder in framework and guest molecules.

```
PLAT772_ALERT_2_B Suspect O-H Bond in CIF: O4       --H_3_1_H   ..       1.45 Ang.
```

Author Response: The alert could be due to the co-existence of disorder in framework and guest molecules.


```
PLAT772_ALERT_2_B Suspect O-H Bond in CIF: O4       --H_3_1_H   ..       1.45 Ang.
```

Author Response: The alert could be due to the co-existence of disorder in framework and guest molecules.

```
PLAT772_ALERT_2_B Suspect O-H Bond in CIF: O4       --H_3_1_H   ..       1.45 Ang.
```

Author Response: The alert could be due to the co-existence of disorder in framework and guest molecules.

Alert level C

```
CELLK01_ALERT_1_C  Check that the cell measurement temperature is in Kelvin.
            Value of measurement temperature given =    10.000
```

Author Response: The structure is obtained from neutron powder diffraction using cold neutron source at 10K from POWGEN, BL-11A, SNS, ORNL.

```
PLAT042_ALERT_1_C Calc. and Reported MoietyFormula Strings  Differ     Please Check
```

Author Response: This is likely due to the decimal points considers in the calculation.

```
PLAT241_ALERT_2_C High   'MainMol' Ueq as Compared to Neighbors of         Ti Check
```

Author Response: Ti exhibit negative neutron coherent scattering length, thus the thermal parameter is relatively large.

```
PLAT341_ALERT_3_C Low Bond Precision on  C-C Bonds ..............      0.014 Ang.
```

Author Response: Structure are obtained from powder diffraction refinement.

```
PLAT601_ALERT_2_C Unit Cell Contains Solvent Accessible VOIDS of .         95 Ang**3
```

Author Response: The void is unoccupied possibly due to kinetic barriers of the diffusion of guest benzene in the framework.

Alert level G

```
FORMU01_ALERT_1_G  There is a discrepancy between the atom counts in the
            _chemical_formula_sum and _chemical_formula_moiety. This is
            usually due to the moiety formula being in the wrong format.
            Atom count from _chemical_formula_sum:   C23.06 H7 D11.07 O9 Ti2
            Atom count from _chemical_formula_moiety:C12.82559 H7.4056 D1.7256 O9
CELLZ01_ALERT_1_G Difference between formula and atom_site contents detected.
CELLZ01_ALERT_1_G ALERT: check formula stoichiometry or atom site occupancies.
           From the CIF: _cell_formula_units_Z    8
           From the CIF: _chemical_formula_sum  C23.06 H7 D11.07 O9 Ti2
           TEST: Compare cell contents of formula and atom_site data

           atom    Z*formula  cif sites diff
           C        184.48    184.51   -0.03
           H         56.00     55.99    0.01
           D         88.56     88.52    0.04
           O         72.00     72.00    0.00
           Ti        16.00     16.00    0.00
PLAT004_ALERT_5_G Polymeric Structure Found with Maximum Dimension          3 Info
PLAT300_ALERT_4_G Atom Site Occupancy of C3         Constrained at        0.5 Check
PLAT300_ALERT_4_G Atom Site Occupancy of C6         Constrained at        0.5 Check
PLAT300_ALERT_4_G Atom Site Occupancy of C66        Constrained at        0.5 Check
PLAT300_ALERT_4_G Atom Site Occupancy of H4         Constrained at        0.5 Check
PLAT300_ALERT_4_G Atom Site Occupancy of H5         Constrained at        0.5 Check
PLAT300_ALERT_4_G Atom Site Occupancy of H55        Constrained at        0.5 Check
PLAT301_ALERT_3_G Main Residue  Disorder .............(Resd  1  )        21% Note
PLAT301_ALERT_3_G Main Residue  Disorder .............(Resd  2  )       100% Note
PLAT301_ALERT_3_G Main Residue  Disorder .............(Resd  3  )       100% Note
PLAT301_ALERT_3_G Main Residue  Disorder .............(Resd  4  )       100% Note
PLAT301_ALERT_3_G Main Residue  Disorder .............(Resd  5  )       100% Note
PLAT432_ALERT_2_G Short Inter X...Y Contact  O2       ..C_2_1    .       1.96 Ang.
                                      -1/2+x,1/2-y,1/2-z  =     29_455 Check
PLAT432_ALERT_2_G Short Inter X...Y Contact  O2       ..C_2_1    .       1.96 Ang.
                                     -1/2+x,-1/2+y,1/2-z  =     31_445 Check
PLAT432_ALERT_2_G Short Inter X...Y Contact  O2       ..C_3_1    .       2.00 Ang.
                                      -1/2+x,1/2-y,1/2-z  =     29_455 Check
PLAT432_ALERT_2_G Short Inter X...Y Contact  O2       ..C_3_1    .       2.00 Ang.
                                     -1/2+x,-1/2+y,1/2-z  =     31_445 Check
PLAT432_ALERT_2_G Short Inter X...Y Contact  O2       ..C_1_1    .       2.65 Ang.
                                      -1/2+x,1/2-y,1/2-z  =     29_455 Check
PLAT432_ALERT_2_G Short Inter X...Y Contact  O2       ..C_1_1    .       2.65 Ang.
                                     -1/2+x,-1/2+y,1/2-z  =     31_445 Check
PLAT432_ALERT_2_G Short Inter X...Y Contact  O2       ..C_4_1    .       2.70 Ang.
                                     -1/2+x,-1/2+y,1/2-z  =     31_445 Check
PLAT432_ALERT_2_G Short Inter X...Y Contact  O2       ..C_4_1    .       2.70 Ang.
                                      -1/2+x,1/2-y,1/2-z  =     29_455 Check
PLAT432_ALERT_2_G Short Inter X...Y Contact  O2       ..C_4_2    .       2.81 Ang.
                                              1-x,-y,1-z  =     16_656 Check
PLAT432_ALERT_2_G Short Inter X...Y Contact  O2       ..C_4_2    .       2.81 Ang.
                                               1-x,y,1-z  =      3_656 Check
PLAT432_ALERT_2_G Short Inter X...Y Contact  O3       ..C_2_1    .       2.53 Ang.
                                     -1/2+x,-1/2+y,1/2-z  =     31_445 Check
PLAT432_ALERT_2_G Short Inter X...Y Contact  O3       ..C_3_1    .       2.88 Ang.
                                     -1/2+x,-1/2+y,1/2-z  =     31_445 Check
PLAT432_ALERT_2_G Short Inter X...Y Contact  O4       ..C_3_1    .       2.46 Ang.
```

```
                                   -1/2+y,-1/2+x,1/2+z  =     28_445 Check
PLAT432_ALERT_2_G Short Inter X...Y Contact  O4       ..C_3_1    .       2.46 Ang.
                                   -1/2+x,-1/2+y,1/2+z  =     17_445 Check
PLAT432_ALERT_2_G Short Inter X...Y Contact  O4       ..C_3_1    .       2.46 Ang.
                                   -1/2+y,-1/2+x,1/2-z  =     27_445 Check
PLAT432_ALERT_2_G Short Inter X...Y Contact  O4       ..C_3_1    .       2.46 Ang.
                                   -1/2+x,-1/2+y,1/2-z  =     31_445 Check
PLAT432_ALERT_2_G Short Inter X...Y Contact  C2       ..C_3      .       3.08 Ang.
                                          1-x,-1+y,1-z  =      3_646 Check
PLAT432_ALERT_2_G Short Inter X...Y Contact  C2       ..C_3      .       3.08 Ang.
                                             1-x,1-y,z  =      2_665 Check
PLAT432_ALERT_2_G Short Inter X...Y Contact  C2       ..C_3      .       3.08 Ang.
                                            1-x,-1+y,z  =      4_645 Check
PLAT432_ALERT_2_G Short Inter X...Y Contact  C2       ..C_3      .       3.08 Ang.
                                           1-x,1-y,1-z  =     16_666 Check
PLAT432_ALERT_2_G Short Inter X...Y Contact  C4       ..C_2_4    .       2.97 Ang.
                                               y,x,1-z  =     11_556 Check
PLAT432_ALERT_2_G Short Inter X...Y Contact  C4       ..C_2_4    .       2.97 Ang.
                                               x,y,1-z  =     15_556 Check
PLAT432_ALERT_2_G Short Inter X...Y Contact  C4       ..C_2_1    .       2.99 Ang.
                                   -1/2+x,-1/2+y,1/2-z  =     31_445 Check
PLAT432_ALERT_2_G Short Inter X...Y Contact  C4       ..C_2_1    .       2.99 Ang.
                                   -1/2+y,-1/2+x,1/2-z  =     27_445 Check
PLAT432_ALERT_2_G Short Inter X...Y Contact  C4       ..C_3_4    .       3.20 Ang.
                                                 y,x,z  =     12_555 Check
PLAT432_ALERT_2_G Short Inter X...Y Contact  C4       ..C_3_4    .       3.20 Ang.
                                                 x,y,z  =      1_555 Check
PLAT720_ALERT_4_G Number of Unusual/Non-Standard Labels .........         55 Note
PLAT764_ALERT_4_G Overcomplete CIF Bond List Detected (Rep/Expd) .       2.05 Ratio
PLAT773_ALERT_2_G Check long C-C Bond in CIF: C_1      --C_2_2           1.82 Ang.
PLAT773_ALERT_2_G Check long C-C Bond in CIF: C_1      --C_2_2           1.86 Ang.
PLAT773_ALERT_2_G Check long C-C Bond in CIF: C_3      --C_5             1.74 Ang.
PLAT773_ALERT_2_G Check long C-C Bond in CIF: C_4      --C_5             1.72 Ang.
PLAT773_ALERT_2_G Check long C-C Bond in CIF: C_4      --C_5             2.03 Ang.
PLAT773_ALERT_2_G Check long C-C Bond in CIF: C_5      --C_4             1.72 Ang.
PLAT773_ALERT_2_G Check long C-C Bond in CIF: C_5      --C_3             1.74 Ang.
PLAT773_ALERT_2_G Check long C-C Bond in CIF: C_5      --C_6             1.85 Ang.
PLAT773_ALERT_2_G Check long C-C Bond in CIF: C_5      --C_6             2.03 Ang.
PLAT773_ALERT_2_G Check long C-C Bond in CIF: C_5      --C_4             2.03 Ang.
PLAT773_ALERT_2_G Check long C-C Bond in CIF: C_6      --C_5             1.85 Ang.
PLAT773_ALERT_2_G Check long C-C Bond in CIF: C_6      --C_5             2.03 Ang.
PLAT773_ALERT_2_G Check long C-C Bond in CIF: C_2_1    --C_2_1           1.71 Ang.
PLAT773_ALERT_2_G Check long C-C Bond in CIF: C_2_1    --C_4_2           1.85 Ang.
PLAT773_ALERT_2_G Check long C-C Bond in CIF: C_2_1    --C_5_1           1.90 Ang.
PLAT773_ALERT_2_G Check long C-C Bond in CIF: C_3_1    --C_3_1           1.73 Ang.
PLAT773_ALERT_2_G Check long C-C Bond in CIF: C_3_1    --C_3_1           1.85 Ang.
PLAT773_ALERT_2_G Check long C-C Bond in CIF: C_3_1    --C_6_1           1.88 Ang.
PLAT773_ALERT_2_G Check long C-C Bond in CIF: C_4_1    --C_4_1           1.77 Ang.
PLAT773_ALERT_2_G Check long C-C Bond in CIF: C_4_1    --C_4_1           1.77 Ang.
PLAT773_ALERT_2_G Check long C-C Bond in CIF: C_4_1    --C_5_1           1.81 Ang.
PLAT773_ALERT_2_G Check long C-C Bond in CIF: C_4_1    --C_4_1           1.91 Ang.
PLAT773_ALERT_2_G Check long C-C Bond in CIF: C_5_1    --C_6_1           1.71 Ang.
PLAT773_ALERT_2_G Check long C-C Bond in CIF: C_5_1    --C_4_1           1.81 Ang.
PLAT773_ALERT_2_G Check long C-C Bond in CIF: C_5_1    --C_2_1           1.90 Ang.
PLAT773_ALERT_2_G Check long C-C Bond in CIF: C_6_1    --C_5_1           1.71 Ang.
PLAT773_ALERT_2_G Check long C-C Bond in CIF: C_6_1    --C_6_2           1.75 Ang.
PLAT773_ALERT_2_G Check long C-C Bond in CIF: C_6_1    --C_3_1           1.88 Ang.
```

```
PLAT773_ALERT_2_G Check long C-C Bond in CIF: C_1_2    --C_4_4          2.06 Ang.
PLAT773_ALERT_2_G Check long C-C Bond in CIF: C_2_2    --C_1            1.82 Ang.
PLAT773_ALERT_2_G Check long C-C Bond in CIF: C_2_2    --C_1            1.86 Ang.
PLAT773_ALERT_2_G Check long C-C Bond in CIF: C_4_2    --C_2_1          1.85 Ang.
PLAT773_ALERT_2_G Check long C-C Bond in CIF: C_6_2    --C_6_1          1.75 Ang.
PLAT773_ALERT_2_G Check long C-C Bond in CIF: C_1_4    --C_1_4          2.03 Ang.
PLAT773_ALERT_2_G Check long C-C Bond in CIF: C_1_4    --C_3_4          2.04 Ang.
PLAT773_ALERT_2_G Check long C-C Bond in CIF: C_2_4    --C_4_4          1.87 Ang.
PLAT773_ALERT_2_G Check long C-C Bond in CIF: C_2_4    --C_4_4          1.97 Ang.
PLAT773_ALERT_2_G Check long C-C Bond in CIF: C_2_4    --C_6_4          2.04 Ang.
PLAT773_ALERT_2_G Check long C-C Bond in CIF: C_3_4    --C_6_4          1.78 Ang.
PLAT773_ALERT_2_G Check long C-C Bond in CIF: C_3_4    --C_5_4          1.85 Ang.
PLAT773_ALERT_2_G Check long C-C Bond in CIF: C_3_4    --C_3_4          1.96 Ang.
PLAT773_ALERT_2_G Check long C-C Bond in CIF: C_3_4    --C_1_4          2.04 Ang.
PLAT773_ALERT_2_G Check long C-C Bond in CIF: C_4_4    --C_2_4          1.87 Ang.
PLAT773_ALERT_2_G Check long C-C Bond in CIF: C_4_4    --C_2_4          1.97 Ang.
PLAT773_ALERT_2_G Check long C-C Bond in CIF: C_4_4    --C_1_2          2.06 Ang.
PLAT773_ALERT_2_G Check long C-C Bond in CIF: C_5_4    --C_3_4          1.85 Ang.
PLAT773_ALERT_2_G Check long C-C Bond in CIF: C_5_4    --C_6_4          2.03 Ang.
PLAT773_ALERT_2_G Check long C-C Bond in CIF: C_6_4    --C_3_4          1.78 Ang.
PLAT773_ALERT_2_G Check long C-C Bond in CIF: C_6_4    --C_5_4          2.03 Ang.
PLAT773_ALERT_2_G Check long C-C Bond in CIF: C_6_4    --C_2_4          2.04 Ang.
PLAT778_ALERT_2_G Check O..H..X Bond in CIF: H_3_1_H  --O4             1.45 Ang.
PLAT780_ALERT_1_G Coordinates do not Form a Properly Connected Set     Please Do !
PLAT811_ALERT_5_G No ADDSYM Analysis: Too Many Excluded Atoms ....          ! Info
```

---

 22 **ALERT level A** = Most likely a serious problem - resolve or explain
  5 **ALERT level B** = A potentially serious problem, consider carefully
  5 **ALERT level C** = Check. Ensure it is not caused by an omission or oversight
 96 **ALERT level G** = General information/check it is not something unexpected

  6 ALERT type 1 CIF construction/syntax error, inconsistent or missing data
106 ALERT type 2 Indicator that the structure model may be wrong or deficient
  6 ALERT type 3 Indicator that the structure quality may be low
  8 ALERT type 4 Improvement, methodology, query or suggestion
  2 ALERT type 5 Informative message, check

It is advisable to attempt to resolve as many as possible of the alerts in all categories. Often the minor alerts point to easily fixed oversights, errors and omissions in your CIF or refinement strategy, so attention to these fine details can be worthwhile. In order to resolve some of the more serious problems it may be necessary to carry out additional measurements or structure refinements. However, the purpose of your study may justify the reported deviations and the more serious of these should normally be commented upon in the discussion or experimental section of a paper or in the "special_details" fields of the CIF. checkCIF was carefully designed to identify outliers and unusual parameters, but every test has its limitations and alerts that are not important in a particular case may appear. Conversely, the absence of alerts does not guarantee there are no aspects of the results needing attention. It is up to the individual to critically assess their own results and, if necessary, seek expert advice.

Publication of your CIF in IUCr journals

A basic structural check has been run on your CIF. These basic checks will be run on all CIFs submitted for publication in IUCr journals (*Acta Crystallographica*, *Journal of Applied Crystallography*, *Journal of Synchrotron Radiation*); however, if you intend to submit to *Acta Crystallographica Section C* or *E* or *IUCrData*, you should make sure that full publication checks are run on the final version of your CIF prior to submission.

Publication of your CIF in other journals

Please refer to the *Notes for Authors* of the relevant journal for any special instructions relating to CIF submission.

PLATON version of 06/07/2023; check.def file version of 30/06/2023

Datablock C6D6-MIL-125 - ellipsoid plot

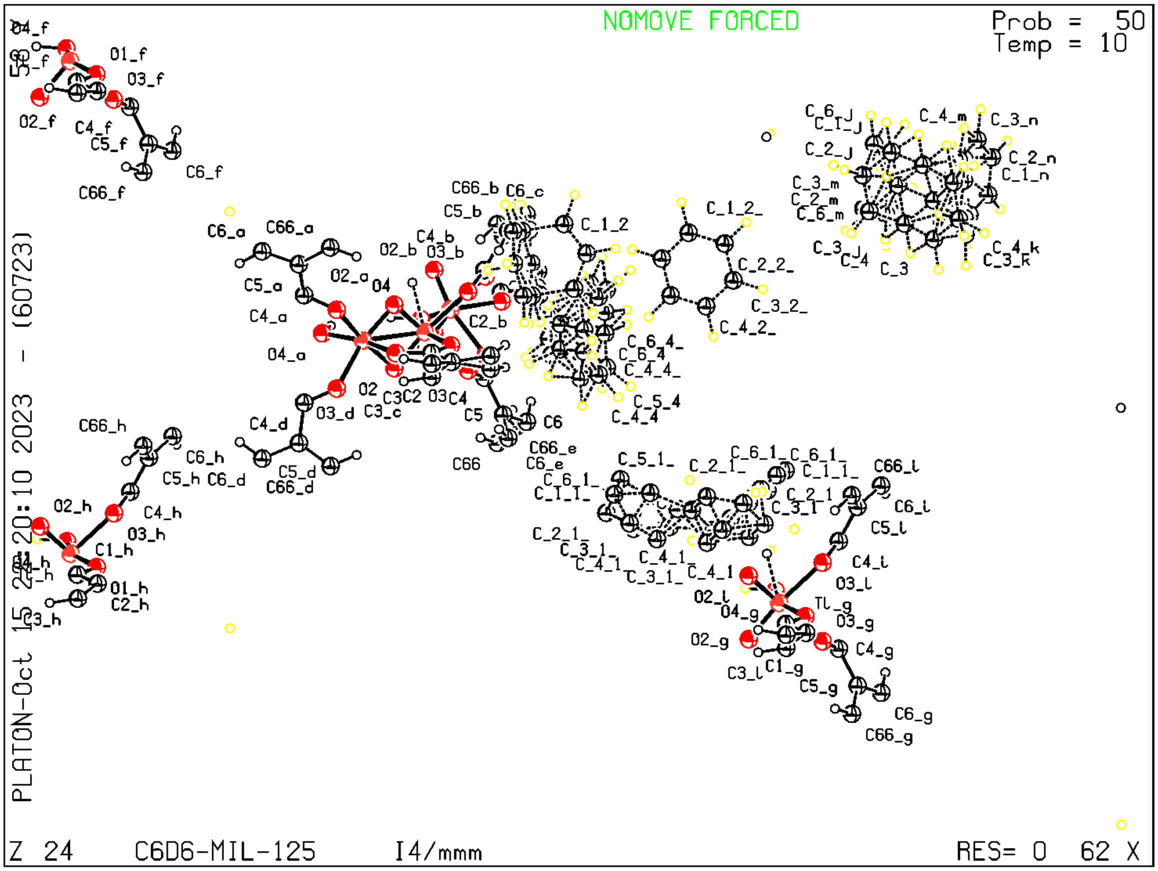

Supplement: Supplementary file 2 — Crystallographic data (11 CIFs) and checkCIF reports. [file 41563_2024_2029_MOESM2_ESM.zip › cifs and check cif reports/C6D6@MIL-125_checkcif.pdf]
