# Supplementary material for: Trace benzene capture by decoration of structural defects in metal–organic framework materials
Source: Nat Mater. 2024 Oct 29;23(11):1531–8. doi: 10.1038/s41563-024-02029-1 (PMC11525167; doi:10.1038/s41563-024-02029-1)

# checkCIF/PLATON report

You have not supplied any structure factors. As a result the full set of tests cannot be run.

THIS REPORT IS FOR GUIDANCE ONLY. IF USED AS PART OF A REVIEW PROCEDURE FOR PUBLICATION, IT SHOULD NOT REPLACE THE EXPERTISE OF AN EXPERIENCED CRYSTALLOGRAPHIC REFEREE.

No syntax errors found. CIF dictionary Interpreting this report

# Datablock: C6H6@MIL-125-Fe

---

| | | | |
|---|---|---|---|
| Bond precision: | C-C = 0.0069 A | | Wavelength=0.82439 |
| Cell: | a=18.65537(5) | b=18.65537(5) | c=18.20263(6) |
| | alpha=90 | beta=90 | gamma=90 |
| Temperature: | 298 K | | |

| | Calculated | Reported |
|---|---|---|
| Volume | 6334.93(4) | 6334.93(4) |
| Space group | I 4/m m m | I4/mmm |
| Hall group | -I 4 2 | -I 4 2 |
| Moiety formula | C3 H1.72 Fe0.05 O2.17 Ti0.45, 0.011(C96 H88), 0.01(C40 H48), 0. | C3 H1.78 Fe0.05 O2.17 Ti0.45, 0.629(C6 H6) |
| Sum formula | C6.69 H5.46 Fe0.05 O2.22 Ti0.45 | C6.77 Fe0.05 H5.55 O2.22 Ti0.45 |
| Mr | 145.79 | 146.85 |
| Dx,g cm-3 | 1.223 | 1.232 |
| Z | 32 | 32 |
| Mu (mm-1) | 0.884 | 0.000 |
| F000 | 2387.2 | 0.0 |
| F000' | 2394.30 | |
| h,k,lmax | 32,32,31 | |
| Nref | 4881 | |
| Tmin,Tmax | | |
| Tmin' | | |

Correction method= Not given

Data completeness= 0.000 Theta(max)=

R(reflections)= wR2(reflections)=

S = Npar=

The following ALERTS were generated. Each ALERT has the format
**test-name_ALERT_alert-type_alert-level.**
Click on the hyperlinks for more details of the test.

### Alert level A

PLAT770_ALERT_2_A Suspect C-H Bond in CIF: C_1 --H_6 . 1.48 Ang.

**Author Response: The close contact alert is due to disorder of guest molecules.**

PLAT770_ALERT_2_A Suspect C-H Bond in CIF: C_1 --H_3 . 1.60 Ang.

**Author Response: The close contact alert is due to disorder of guest molecules.**

PLAT770_ALERT_2_A Suspect C-H Bond in CIF: C_1 --H_6 . 1.62 Ang.

**Author Response: The close contact alert is due to disorder of guest molecules.**

PLAT770_ALERT_2_A Suspect C-H Bond in CIF: C_3 --H_2_3 . 1.42 Ang.

**Author Response: The close contact alert is due to disorder of guest molecules.**

PLAT770_ALERT_2_A Suspect C-H Bond in CIF: C_3 --H_3 . 1.45 Ang.

**Author Response: The close contact alert is due to disorder of guest molecules.**

PLAT770_ALERT_2_A Suspect C-H Bond in CIF: C_3 --H_1 . 1.61 Ang.

**Author Response: The close contact alert is due to disorder of guest molecules.**

PLAT770_ALERT_2_A Suspect C-H Bond in CIF: C_4 --H_5 . 1.31 Ang.

**Author Response: The close contact alert is due to disorder of guest molecules.**

PLAT770_ALERT_2_A Suspect C-H Bond in CIF: C_4 --H_5 . 1.45 Ang.

**Author Response: The close contact alert is due to disorder of guest molecules.**

PLAT770_ALERT_2_A Suspect C-H Bond in CIF: C_4 --H_4 . 1.54 Ang.

**Author Response: The close contact alert is due to disorder of guest molecules.**

PLAT770_ALERT_2_A Suspect C-H Bond in CIF:  C_4      --H_6       .       1.63 Ang.

**Author Response: The close contact alert is due to disorder of guest molecules.**

PLAT770_ALERT_2_A Suspect C-H Bond in CIF:  C_5      --H_5       .       1.32 Ang.

**Author Response: The close contact alert is due to disorder of guest molecules.**

PLAT770_ALERT_2_A Suspect C-H Bond in CIF:  C_6      --H_5       .       1.55 Ang.

**Author Response: The close contact alert is due to disorder of guest molecules.**

PLAT770_ALERT_2_A Suspect C-H Bond in CIF:  C_6      --H_5       .       1.57 Ang.

**Author Response: The close contact alert is due to disorder of guest molecules.**

PLAT770_ALERT_2_A Suspect C-H Bond in CIF:  H_1      --C_2_3     .       1.54 Ang.

**Author Response: The close contact alert is due to disorder of guest molecules.**

PLAT770_ALERT_2_A Suspect C-H Bond in CIF:  H_1      --C_3       .       1.61 Ang.

**Author Response: The close contact alert is due to disorder of guest molecules.**

PLAT770_ALERT_2_A Suspect C-H Bond in CIF:  H_2      --C_3_3     .       1.47 Ang.

**Author Response: The close contact alert is due to disorder of guest molecules.**

PLAT770_ALERT_2_A Suspect C-H Bond in CIF:  H_3      --C_2_3     .       1.43 Ang.

**Author Response: The close contact alert is due to disorder of guest molecules.**

PLAT770_ALERT_2_A Suspect C-H Bond in CIF:  H_3      --C_3       .       1.45 Ang.

**Author Response: The close contact alert is due to disorder of guest molecules.**

PLAT770_ALERT_2_A Suspect C-H Bond in CIF:  H_3      --C_1       .       1.60 Ang.

**Author Response: The close contact alert is due to disorder of guest molecules.**

PLAT770_ALERT_2_A Suspect C-H Bond in CIF:  H_4      --C_4       .       1.54 Ang.

**Author Response: The close contact alert is due to disorder of guest molecules.**

PLAT770_ALERT_2_A Suspect C-H Bond in CIF:  H_5      --C_4       .       1.31 Ang.

**Author Response: The close contact alert is due to disorder of guest molecules.**

PLAT770_ALERT_2_A Suspect C-H Bond in CIF:  H_5      --C_5       .       1.32 Ang.

**Author Response: The close contact alert is due to disorder of guest molecules.**

PLAT770_ALERT_2_A Suspect C-H Bond in CIF:  H_5      --C_4       .       1.45 Ang.

**Author Response: The close contact alert is due to disorder of guest molecules.**

PLAT770_ALERT_2_A Suspect C-H Bond in CIF:  H_5      --C_6       .       1.55 Ang.

**Author Response: The close contact alert is due to disorder of guest molecules.**

PLAT770_ALERT_2_A Suspect C-H Bond in CIF:  H_5      --C_6       .       1.57 Ang.

**Author Response: The close contact alert is due to disorder of guest molecules.**

PLAT770_ALERT_2_A Suspect C-H Bond in CIF:  H_6      --C_1       .       1.48 Ang.

**Author Response: The close contact alert is due to disorder of guest molecules.**

PLAT770_ALERT_2_A Suspect C-H Bond in CIF:  H_6      --C_1       .       1.62 Ang.

**Author Response: The close contact alert is due to disorder of guest molecules.**

PLAT770_ALERT_2_A Suspect C-H Bond in CIF:  H_6      --C_4       .       1.63 Ang.

**Author Response: The close contact alert is due to disorder of guest molecules.**

PLAT770_ALERT_2_A Suspect C-H Bond in CIF:  C_4_1    --H_4_3     .       1.62 Ang.

**Author Response: The close contact alert is due to disorder of guest molecules.**

PLAT770_ALERT_2_A Suspect C-H Bond in CIF:  C_5_1    --H_4_3     .       1.51 Ang.

**Author Response: The close contact alert is due to disorder of guest molecules.**

PLAT770_ALERT_2_A Suspect C-H Bond in CIF:  C_6_1    --H_5_1     .       1.39 Ang.

**Author Response: The close contact alert is due to disorder of guest molecules.**

PLAT770_ALERT_2_A Suspect C-H Bond in CIF:  C_6_1    --H_6_1     .       1.52 Ang.

**Author Response: The close contact alert is due to disorder of guest molecules.**

PLAT770_ALERT_2_A Suspect C-H Bond in CIF:  H_2_1    --C_4_3     .       1.42 Ang.

**Author Response: The close contact alert is due to disorder of guest molecules.**

PLAT770_ALERT_2_A Suspect C-H Bond in CIF:  H_2_1    --C_5_3     .       1.58 Ang.

**Author Response: The close contact alert is due to disorder of guest molecules.**

PLAT770_ALERT_2_A Suspect C-H Bond in CIF:  H_3_1    --C_3_3     .       1.61 Ang.

**Author Response: The close contact alert is due to disorder of guest molecules.**

PLAT770_ALERT_2_A Suspect C-H Bond in CIF:  H_5_1    --C_6_1     .       1.39 Ang.

**Author Response: The close contact alert is due to disorder of guest molecules.**

PLAT770_ALERT_2_A Suspect C-H Bond in CIF:  H_5_1    --C_4_3     .       1.51 Ang.

**Author Response: The close contact alert is due to disorder of guest molecules.**

PLAT770_ALERT_2_A Suspect C-H Bond in CIF:  H_6_1    --C_6_1     .       1.52 Ang.

**Author Response: The close contact alert is due to disorder of guest molecules.**

PLAT770_ALERT_2_A Suspect C-H Bond in CIF:  C_1_2    --H_6_2     .       1.35 Ang.

**Author Response: The close contact alert is due to disorder of guest molecules.**

PLAT770_ALERT_2_A Suspect C-H Bond in CIF: C_2_2 --H_6_2 . 1.52 Ang.

**Author Response: The close contact alert is due to disorder of guest molecules.**

PLAT770_ALERT_2_A Suspect C-H Bond in CIF: C_2_2 --H_1_2 . 1.55 Ang.

**Author Response: The close contact alert is due to disorder of guest molecules.**

PLAT770_ALERT_2_A Suspect C-H Bond in CIF: C_2_2 --H_6_3 . 1.61 Ang.

**Author Response: The close contact alert is due to disorder of guest molecules.**

PLAT770_ALERT_2_A Suspect C-H Bond in CIF: C_3_2 --H_3_2 . 1.34 Ang.

**Author Response: The close contact alert is due to disorder of guest molecules.**

PLAT770_ALERT_2_A Suspect C-H Bond in CIF: C_5_2 --H_5_2 . 1.49 Ang.

**Author Response: The close contact alert is due to disorder of guest molecules.**

PLAT770_ALERT_2_A Suspect C-H Bond in CIF: C_5_2 --H_6_2 . 1.56 Ang.

**Author Response: The close contact alert is due to disorder of guest molecules.**

PLAT770_ALERT_2_A Suspect C-H Bond in CIF: C_5_2 --H_6_2 . 1.58 Ang.

**Author Response: The close contact alert is due to disorder of guest molecules.**

PLAT770_ALERT_2_A Suspect C-H Bond in CIF: C_6_2 --H_6_2 . 1.44 Ang.

**Author Response: The close contact alert is due to disorder of guest molecules.**

PLAT770_ALERT_2_A Suspect C-H Bond in CIF: C_6_2 --H_5_2 . 1.52 Ang.

**Author Response: The close contact alert is due to disorder of guest molecules.**

PLAT770_ALERT_2_A Suspect C-H Bond in CIF: H_1_2 --C_2_2 . 1.55 Ang.

**Author Response: The close contact alert is due to disorder of guest molecules.**

PLAT770_ALERT_2_A Suspect C-H Bond in CIF:  H_3_2    --C_3_2     .       1.34 Ang.

**Author Response: The close contact alert is due to disorder of guest molecules.**

PLAT770_ALERT_2_A Suspect C-H Bond in CIF:  H_5_2    --C_5_2     .       1.49 Ang.

**Author Response: The close contact alert is due to disorder of guest molecules.**

PLAT770_ALERT_2_A Suspect C-H Bond in CIF:  H_5_2    --C_6_2     .       1.52 Ang.

**Author Response: The close contact alert is due to disorder of guest molecules.**

PLAT770_ALERT_2_A Suspect C-H Bond in CIF:  H_6_2    --C_1_2     .       1.35 Ang.

**Author Response: The close contact alert is due to disorder of guest molecules.**

PLAT770_ALERT_2_A Suspect C-H Bond in CIF:  H_6_2    --C_6_2     .       1.44 Ang.

**Author Response: The close contact alert is due to disorder of guest molecules.**

PLAT770_ALERT_2_A Suspect C-H Bond in CIF:  H_6_2    --C_2_2     .       1.52 Ang.

**Author Response: The close contact alert is due to disorder of guest molecules.**

PLAT770_ALERT_2_A Suspect C-H Bond in CIF:  H_6_2    --C_5_2     .       1.56 Ang.

**Author Response: The close contact alert is due to disorder of guest molecules.**

PLAT770_ALERT_2_A Suspect C-H Bond in CIF:  H_6_2    --C_5_2     .       1.58 Ang.

**Author Response: The close contact alert is due to disorder of guest molecules.**

PLAT770_ALERT_2_A Suspect C-H Bond in CIF:  C_2_3    --H_3       .       1.43 Ang.

**Author Response: The close contact alert is due to disorder of guest molecules.**

PLAT770_ALERT_2_A Suspect C-H Bond in CIF:  C_2_3    --H_1       .       1.54 Ang.

**Author Response: The close contact alert is due to disorder of guest molecules.**

PLAT770_ALERT_2_A Suspect C-H Bond in CIF:  C_2_3    --H7        .       1.62 Ang.

**Author Response: The close contact alert is due to disorder of guest molecules.**

PLAT770_ALERT_2_A Suspect C-H Bond in CIF:  C_3_3    --H_2       .       1.47 Ang.

**Author Response: The close contact alert is due to disorder of guest molecules.**

PLAT770_ALERT_2_A Suspect C-H Bond in CIF:  C_3_3    --H_3_1     .       1.61 Ang.

**Author Response: The close contact alert is due to disorder of guest molecules.**

PLAT770_ALERT_2_A Suspect C-H Bond in CIF:  C_4_3    --H_2_1     .       1.42 Ang.

**Author Response: The close contact alert is due to disorder of guest molecules.**

PLAT770_ALERT_2_A Suspect C-H Bond in CIF:  C_4_3    --H_5_1     .       1.51 Ang.

**Author Response: The close contact alert is due to disorder of guest molecules.**

PLAT770_ALERT_2_A Suspect C-H Bond in CIF:  C_5_3    --H_2_1     .       1.58 Ang.

**Author Response: The close contact alert is due to disorder of guest molecules.**

PLAT770_ALERT_2_A Suspect C-H Bond in CIF:  C_5_3    --H_6_3     .       1.60 Ang.

**Author Response: The close contact alert is due to disorder of guest molecules.**

PLAT770_ALERT_2_A Suspect C-H Bond in CIF:  C_5_3    --H_5_3     .       1.62 Ang.

**Author Response: The close contact alert is due to disorder of guest molecules.**

PLAT770_ALERT_2_A Suspect C-H Bond in CIF:  H_2_3    --C_3       .       1.42 Ang.

**Author Response: The close contact alert is due to disorder of guest molecules.**

PLAT770_ALERT_2_A Suspect C-H Bond in CIF:  H_4_3    --C_5_1     .       1.51 Ang.

**Author Response: The close contact alert is due to disorder of guest molecules.**

PLAT770_ALERT_2_A Suspect C-H Bond in CIF: H_4_3 --C_4_1 . 1.62 Ang.

**Author Response: The close contact alert is due to disorder of guest molecules.**

PLAT770_ALERT_2_A Suspect C-H Bond in CIF: H_5_3 --C_5_3 . 1.62 Ang.

**Author Response: The close contact alert is due to disorder of guest molecules.**

PLAT770_ALERT_2_A Suspect C-H Bond in CIF: H_6_3 --C_5_3 . 1.60 Ang.

**Author Response: The close contact alert is due to disorder of guest molecules.**

PLAT770_ALERT_2_A Suspect C-H Bond in CIF: H_6_3 --C_2_2 . 1.61 Ang.

**Author Response: The close contact alert is due to disorder of guest molecules.**

PLAT770_ALERT_2_A Suspect C-H Bond in CIF: H7 --C_2_3 . 1.62 Ang.

**Author Response: The close contact alert is due to disorder of guest molecules.**

---

**Alert level B**

PLAT420_ALERT_2_B D-H Bond Without Acceptor O4 --H4O . Please Check

**Author Response: The guest molecule benzene does not have a hydrogen bonding acceptor.**

---

**Alert level C**

PLAT041_ALERT_1_C Calc. and Reported SumFormula Strings Differ Please Check

**Author Response: The issues is caused by checkcif algorithm ignoring a unique molecule in the structure with the naming style as X_Y, where X is the element and Y is the internal atom index.**

PLAT042_ALERT_1_C Calc. and Reported MoietyFormula Strings Differ Please Check

**Author Response: The issues is caused by checkcif algorithm ignoring a unique molecule in the structure with the naming style as X_Y, where X is the element and Y is the internal atom index.**

PLAT043_ALERT_1_C Calculated and Reported Mol. Weight Differ by .. 1.06 Check

**Author Response: The issues is caused by checkcif algorithm ignoring a unique molecule in the structure with the naming style as X_Y, where X is the element and Y is the internal atom index.**

PLAT077_ALERT_4_C Unitcell Contains Non-integer Number of Atoms .. Please Check

**Author Response: The structure contains disordered framework and guest molecules.**

PLAT244_ALERT_4_C Low 'Solvent' Ueq as Compared to Neighbors of O1 Check

**Author Response: O1 is X-ray heavier compared to neightors and the thermal vibration is less intensive.**

PLAT244_ALERT_4_C Low 'Solvent' Ueq as Compared to Neighbors of O3 Check

**Author Response: O1 is X-ray heavier compared to neightors and the thermal vibration is less intensive.**

PLAT334_ALERT_2_C Small <C-C> Benzene Dist. C2 -C3_c . 1.35 Ang.

**Author Response: Structure are obtained from powder diffraction refinement, and this could be due to insufficient resolution.**

PLAT341_ALERT_3_C Low Bond Precision on C-C Bonds .............. 0.00687 Ang.

**Author Response: Structure are obtained from powder diffraction refinement. Low precision could be due to insufficient resolution.**

---

**Alert level G**

FORMU01_ALERT_1_G There is a discrepancy between the atom counts in the
_chemical_formula_sum and _chemical_formula_moiety. This is
usually due to the moiety formula being in the wrong format.
Atom count from _chemical_formula_sum: C6.77 H5.55 Fe.05 O2.22 Ti0.4
Atom count from _chemical_formula_moiety:C6.774 H5.554 Fe.05 O2.17 Ti0
FORMU01_ALERT_2_G There is a discrepancy between the atom counts in the
_chemical_formula_sum and the formula from the _atom_site* data.
Atom count from _chemical_formula_sum:C6.77 H5.55 Fe.05 O2.22 Ti0.45
Atom count from the _atom_site data: C6.771599 H5.457799 Fe.0543 O2.2
CELLZ01_ALERT_1_G Difference between formula and atom_site contents detected.
CELLZ01_ALERT_1_G WARNING: H atoms missing from atom site list. Is this intentional?
From the CIF: _cell_formula_units_Z 32
From the CIF: _chemical_formula_sum C6.77 Fe0.05 H5.55 O2.22 Ti0.45
TEST: Compare cell contents of formula and atom_site data

```
          atom    Z*formula  cif sites diff
          C        216.64    216.69   -0.05
          Fe         1.60      1.74   -0.14
          H        177.60    174.65    2.95
          O         71.04     71.13   -0.09
          Ti        14.40     14.26    0.14
PLAT004_ALERT_5_G Polymeric Structure Found with Maximum Dimension          3 Info
PLAT092_ALERT_4_G Check: Wavelength Given is not Cu,Ga,Mo,Ag,In Ka    0.82439 Ang.
PLAT143_ALERT_4_G s.u. on c - Axis Small or Missing .............    0.00006 Ang.
PLAT164_ALERT_4_G Nr. of Refined C-H H-Atoms in Heavy-Atom Struct.          2 Note
PLAT301_ALERT_3_G Main Residue  Disorder .............(Resd  1  )        32% Note
PLAT301_ALERT_3_G Main Residue  Disorder .............(Resd  2  )       100% Note
PLAT301_ALERT_3_G Main Residue  Disorder .............(Resd  3  )       100% Note
PLAT301_ALERT_3_G Main Residue  Disorder .............(Resd  4  )       100% Note
PLAT301_ALERT_3_G Main Residue  Disorder .............(Resd  5  )       100% Note
PLAT302_ALERT_4_G Anion/Solvent/Minor-Residue Disorder (Resd  6  )       100% Note
PLAT311_ALERT_2_G Isolated Disordered Oxygen Atom (No H's ?) .....         O5 Check
PLAT411_ALERT_2_G Short Inter H...H Contact  H4       ..H_2_2    .       1.68 Ang.
                                              x,1-y,1-z  =     13_566 Check
PLAT411_ALERT_2_G Short Inter H...H Contact  H4       ..H_2_2    .       1.68 Ang.
                                                x,1-y,z  =     14_565 Check
PLAT411_ALERT_2_G Short Inter H...H Contact  H5       ..H_4_2    .       1.83 Ang.
                                             -1+y,x,1-z  =     11_456 Check
PLAT411_ALERT_2_G Short Inter H...H Contact  H5       ..H_1_3    .       1.85 Ang.
                                    -1/2+x,1/2-y,-1/2+z  =     30_454 Check
PLAT413_ALERT_2_G Short Inter XH3 .. XHn     H4       ..H_1_1    .       1.95 Ang.
                                     1/2-y,-1/2+x,1/2+z  =     24_545 Check
PLAT413_ALERT_2_G Short Inter XH3 .. XHn     H4       ..H_1_1    .       1.95 Ang.
                                     1/2-y,-1/2+x,1/2-z  =     23_545 Check
PLAT432_ALERT_2_G Short Inter X...Y Contact  O1       ..C_3_2    .       2.97 Ang.
                                              x,1-y,1-z  =     13_566 Check
PLAT432_ALERT_2_G Short Inter X...Y Contact  O1       ..C_3_2    .       2.97 Ang.
                                                x,1-y,z  =     14_565 Check
PLAT432_ALERT_2_G Short Inter X...Y Contact  C1       ..C_3_2    .       2.74 Ang.
                                              x,1-y,1-z  =     13_566 Check
PLAT432_ALERT_2_G Short Inter X...Y Contact  C1       ..C_3_2    .       2.74 Ang.
                                               x,-1+y,z  =      1_545 Check
PLAT432_ALERT_2_G Short Inter X...Y Contact  C1       ..C_3_2    .       2.74 Ang.
                                             x,-1+y,1-z  =     15_546 Check
PLAT432_ALERT_2_G Short Inter X...Y Contact  C1       ..C_3_2    .       2.74 Ang.
                                                x,1-y,z  =     14_565 Check
PLAT432_ALERT_2_G Short Inter X...Y Contact  C2       ..C_3_2    .       2.86 Ang.
                                               x,-1+y,z  =      1_545 Check
PLAT432_ALERT_2_G Short Inter X...Y Contact  C2       ..C_3_2    .       2.86 Ang.
                                                x,1-y,z  =     14_565 Check
PLAT432_ALERT_2_G Short Inter X...Y Contact  C2       ..C_3_2    .       2.86 Ang.
                                             x,-1+y,1-z  =     15_546 Check
PLAT432_ALERT_2_G Short Inter X...Y Contact  C2       ..C_3_2    .       2.86 Ang.
                                              x,1-y,1-z  =     13_566 Check
PLAT432_ALERT_2_G Short Inter X...Y Contact  C2       ..C_2_2    .       2.93 Ang.
                                             x,-1+y,1-z  =     15_546 Check
PLAT432_ALERT_2_G Short Inter X...Y Contact  C2       ..C_2_2    .       2.93 Ang.
                                               x,-1+y,z  =      1_545 Check
PLAT432_ALERT_2_G Short Inter X...Y Contact  C2       ..C_2_2    .       2.93 Ang.
                                              x,1-y,1-z  =     13_566 Check
PLAT432_ALERT_2_G Short Inter X...Y Contact  C2       ..C_2_2    .       2.93 Ang.
```

```
                                                   x,1-y,z  =     14_565 Check
PLAT432_ALERT_2_G Short Inter X...Y Contact  C3       ..C_2_2    .       2.66 Ang.
                                                 x,1-y,1-z  =     13_566 Check
PLAT432_ALERT_2_G Short Inter X...Y Contact  C3       ..C_2_2    .       2.66 Ang.
                                                   x,1-y,z  =     14_565 Check
PLAT432_ALERT_2_G Short Inter X...Y Contact  C_3_3    ..C_3_3    .       2.82 Ang.
                                                     y,x,z  =     12_555 Check
PLAT720_ALERT_4_G Number of Unusual/Non-Standard Labels .........          48 Note
PLAT764_ALERT_4_G Overcomplete CIF Bond List Detected (Rep/Expd) .       2.19 Ratio
PLAT773_ALERT_2_G Check long C-C Bond in CIF: C_1      --C_1             2.04 Ang.
PLAT773_ALERT_2_G Check long C-C Bond in CIF: C_2      --C_4             1.98 Ang.
PLAT773_ALERT_2_G Check long C-C Bond in CIF: C_2      --C_3             2.00 Ang.
PLAT773_ALERT_2_G Check long C-C Bond in CIF: C_3      --C_6             1.89 Ang.
PLAT773_ALERT_2_G Check long C-C Bond in CIF: C_3      --C_6             1.97 Ang.
PLAT773_ALERT_2_G Check long C-C Bond in CIF: C_3      --C_2             2.00 Ang.
PLAT773_ALERT_2_G Check long C-C Bond in CIF: C_4      --C_5             1.73 Ang.
PLAT773_ALERT_2_G Check long C-C Bond in CIF: C_4      --C_2             1.98 Ang.
PLAT773_ALERT_2_G Check long C-C Bond in CIF: C_5      --C_4             1.73 Ang.
PLAT773_ALERT_2_G Check long C-C Bond in CIF: C_6      --C_3             1.89 Ang.
PLAT773_ALERT_2_G Check long C-C Bond in CIF: C_6      --C_3             1.97 Ang.
PLAT773_ALERT_2_G Check long C-C Bond in CIF: C_1_1    --C_1_1           1.88 Ang.
PLAT773_ALERT_2_G Check long C-C Bond in CIF: C_1_1    --C_2_1           1.90 Ang.
PLAT773_ALERT_2_G Check long C-C Bond in CIF: C_1_1    --C_6_1           1.95 Ang.
PLAT773_ALERT_2_G Check long C-C Bond in CIF: C_1_1    --C_4_3           2.01 Ang.
PLAT773_ALERT_2_G Check long C-C Bond in CIF: C_1_1    --C_5_3           2.03 Ang.
PLAT773_ALERT_2_G Check long C-C Bond in CIF: C_2_1    --C_6_1           1.80 Ang.
PLAT773_ALERT_2_G Check long C-C Bond in CIF: C_2_1    --C_1_1           1.90 Ang.
PLAT773_ALERT_2_G Check long C-C Bond in CIF: C_3_1    --C_4_3           1.76 Ang.
PLAT773_ALERT_2_G Check long C-C Bond in CIF: C_3_1    --C_4_3           1.90 Ang.
PLAT773_ALERT_2_G Check long C-C Bond in CIF: C_4_1    --C_5_1           1.87 Ang.
PLAT773_ALERT_2_G Check long C-C Bond in CIF: C_5_1    --C_4_1           1.87 Ang.
PLAT773_ALERT_2_G Check long C-C Bond in CIF: C_6_1    --C_4_3           1.73 Ang.
PLAT773_ALERT_2_G Check long C-C Bond in CIF: C_6_1    --C_2_1           1.80 Ang.
PLAT773_ALERT_2_G Check long C-C Bond in CIF: C_6_1    --C_1_1           1.95 Ang.
PLAT773_ALERT_2_G Check long C-C Bond in CIF: C_1_2    --C_4_2           1.89 Ang.
PLAT773_ALERT_2_G Check long C-C Bond in CIF: C_1_2    --C_2_2           1.93 Ang.
PLAT773_ALERT_2_G Check long C-C Bond in CIF: C_1_2    --C_4_2           2.03 Ang.
PLAT773_ALERT_2_G Check long C-C Bond in CIF: C_2_2    --C_5_2           1.93 Ang.
PLAT773_ALERT_2_G Check long C-C Bond in CIF: C_2_2    --C_1_2           1.93 Ang.
PLAT773_ALERT_2_G Check long C-C Bond in CIF: C_2_2    --C_4_2           1.96 Ang.
PLAT773_ALERT_2_G Check long C-C Bond in CIF: C_2_2    --C_6_2           2.04 Ang.
PLAT773_ALERT_2_G Check long C-C Bond in CIF: C_4_2    --C_6_2           1.82 Ang.
PLAT773_ALERT_2_G Check long C-C Bond in CIF: C_4_2    --C_1_2           1.89 Ang.
PLAT773_ALERT_2_G Check long C-C Bond in CIF: C_4_2    --C_2_2           1.96 Ang.
PLAT773_ALERT_2_G Check long C-C Bond in CIF: C_4_2    --C_6_2           2.01 Ang.
PLAT773_ALERT_2_G Check long C-C Bond in CIF: C_4_2    --C_1_2           2.03 Ang.
PLAT773_ALERT_2_G Check long C-C Bond in CIF: C_5_2    --C_5_2           1.87 Ang.
PLAT773_ALERT_2_G Check long C-C Bond in CIF: C_5_2    --C_2_2           1.93 Ang.
PLAT773_ALERT_2_G Check long C-C Bond in CIF: C_5_2    --C_6_2           2.02 Ang.
PLAT773_ALERT_2_G Check long C-C Bond in CIF: C_6_2    --C_4_2           1.82 Ang.
PLAT773_ALERT_2_G Check long C-C Bond in CIF: C_6_2    --C_4_2           2.01 Ang.
PLAT773_ALERT_2_G Check long C-C Bond in CIF: C_6_2    --C_5_2           2.02 Ang.
PLAT773_ALERT_2_G Check long C-C Bond in CIF: C_6_2    --C_2_2           2.04 Ang.
PLAT773_ALERT_2_G Check long C-C Bond in CIF: C_1_3    --C_3_3           1.88 Ang.
PLAT773_ALERT_2_G Check long C-C Bond in CIF: C_2_3    --C_3_3           1.91 Ang.
PLAT773_ALERT_2_G Check long C-C Bond in CIF: C_3_3    --C_1_3           1.88 Ang.
PLAT773_ALERT_2_G Check long C-C Bond in CIF: C_3_3    --C_2_3           1.91 Ang.
```

```
PLAT773_ALERT_2_G Check long C-C Bond in CIF: C_4_3    --C_6_1           1.73 Ang.
PLAT773_ALERT_2_G Check long C-C Bond in CIF: C_4_3    --C_3_1           1.76 Ang.
PLAT773_ALERT_2_G Check long C-C Bond in CIF: C_4_3    --C_3_1           1.90 Ang.
PLAT773_ALERT_2_G Check long C-C Bond in CIF: C_4_3    --C_6_3           1.98 Ang.
PLAT773_ALERT_2_G Check long C-C Bond in CIF: C_4_3    --C_1_1           2.01 Ang.
PLAT773_ALERT_2_G Check long C-C Bond in CIF: C_5_3    --C_1_1           2.03 Ang.
PLAT773_ALERT_2_G Check long C-C Bond in CIF: C_6_3    --C_4_3           1.98 Ang.
PLAT811_ALERT_5_G No ADDSYM Analysis: Too Many Excluded Atoms ....          ! Info
```

---

```
  74 ALERT level A = Most likely a serious problem - resolve or explain
   1 ALERT level B = A potentially serious problem, consider carefully
   8 ALERT level C = Check. Ensure it is not caused by an omission or oversight
  96 ALERT level G = General information/check it is not something unexpected

   6 ALERT type 1 CIF construction/syntax error, inconsistent or missing data
 156 ALERT type 2 Indicator that the structure model may be wrong or deficient
   6 ALERT type 3 Indicator that the structure quality may be low
   9 ALERT type 4 Improvement, methodology, query or suggestion
   2 ALERT type 5 Informative message, check
```

---

---

It is advisable to attempt to resolve as many as possible of the alerts in all categories. Often the minor alerts point to easily fixed oversights, errors and omissions in your CIF or refinement strategy, so attention to these fine details can be worthwhile. In order to resolve some of the more serious problems it may be necessary to carry out additional measurements or structure refinements. However, the purpose of your study may justify the reported deviations and the more serious of these should normally be commented upon in the discussion or experimental section of a paper or in the "special_details" fields of the CIF. checkCIF was carefully designed to identify outliers and unusual parameters, but every test has its limitations and alerts that are not important in a particular case may appear. Conversely, the absence of alerts does not guarantee there are no aspects of the results needing attention. It is up to the individual to critically assess their own results and, if necessary, seek expert advice.

**Publication of your CIF in IUCr journals**

A basic structural check has been run on your CIF. These basic checks will be run on all CIFs submitted for publication in IUCr journals (*Acta Crystallographica*, *Journal of Applied Crystallography*, *Journal of Synchrotron Radiation*); however, if you intend to submit to *Acta Crystallographica Section C* or *E* or *IUCrData*, you should make sure that full publication checks are run on the final version of your CIF prior to submission.

**Publication of your CIF in other journals**

Please refer to the *Notes for Authors* of the relevant journal for any special instructions relating to CIF submission.

---

**PLATON version of 06/07/2023; check.def file version of 30/06/2023**

**Datablock C6H6@MIL-125-Fe** - ellipsoid plot

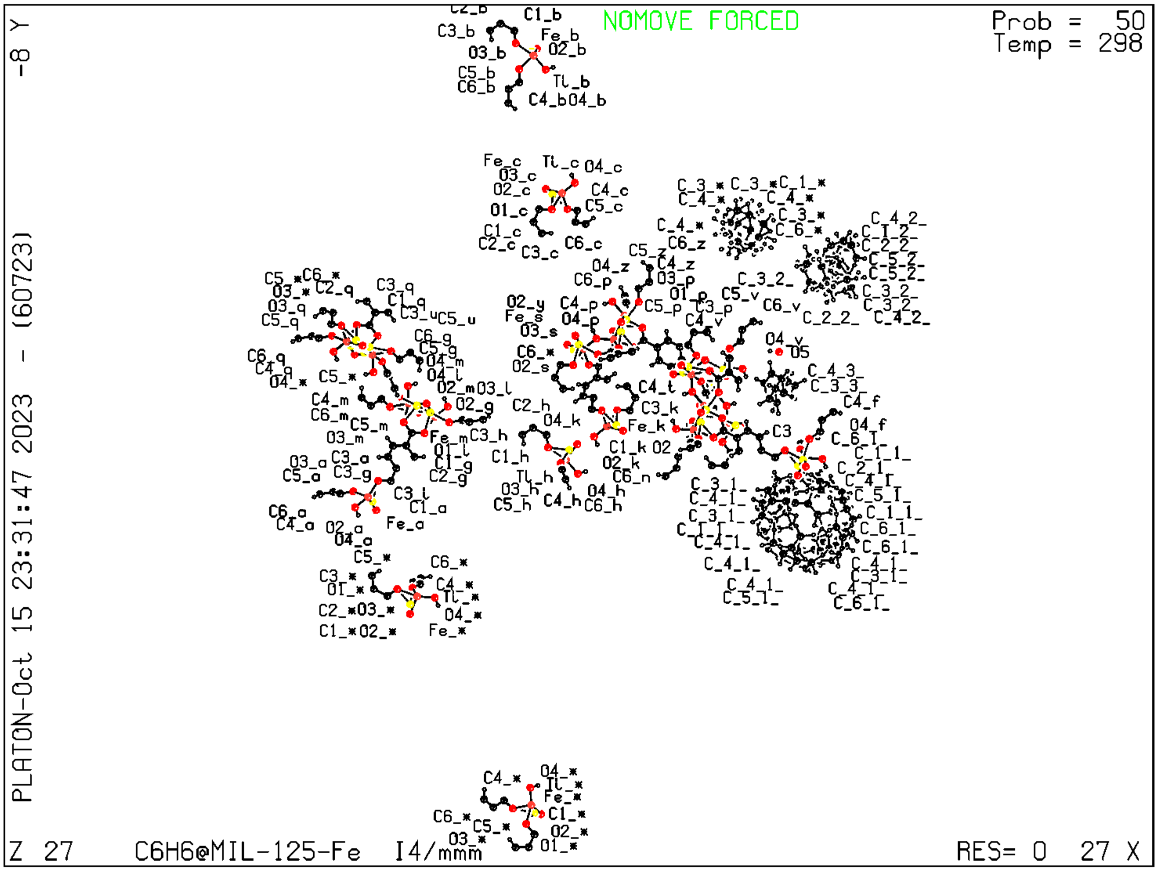

Supplement: Supplementary file 2 — Crystallographic data (11 CIFs) and checkCIF reports. [file 41563_2024_2029_MOESM2_ESM.zip › cifs and check cif reports/C6H6@MIL-125-Fe_checkcif.pdf]
