# Supplementary material for: Trace benzene capture by decoration of structural defects in metal–organic framework materials
Source: Nat Mater. 2024 Oct 29;23(11):1531–8. doi: 10.1038/s41563-024-02029-1 (PMC11525167; doi:10.1038/s41563-024-02029-1)

checkCIF/PLATON report

You have not supplied any structure factors. As a result the full set of tests cannot be run.

THIS REPORT IS FOR GUIDANCE ONLY. IF USED AS PART OF A REVIEW PROCEDURE FOR PUBLICATION, IT SHOULD NOT REPLACE THE EXPERTISE OF AN EXPERIENCED CRYSTALLOGRAPHIC REFEREE.

No syntax errors found. CIF dictionary Interpreting this report

Datablock: C6H6@MIL-125-Ni

Bond precision:	C-C = 0.0069 A		Wavelength=0.82439
Cell:	a=18.65121(5)	b=18.65121(5)	c=18.20217(6)
	alpha=90	beta=90	gamma=90
Temperature:	298 K		

	Calculated	Reported
Volume	6331.95(4)	6331.94(4)
Space group	I 4/m m m	I4/mmm
Hall group	-I 4 2	-I 4 2
Moiety formula	C3 H1.78 Ni0.05 O2.17 Ti0.45, 0.01(C88 H88), 0.021(C48 H48), 0.	C3 H1.78 Ni0.05 O2.22 Ti0.45, 0.58(C6 H6)
Sum formula	C6.31 H5.17 Ni0.05 O2.22 Ti0.45	C6.48 H5.26 Ni0.05 O2.22 Ti0.45
Mr	141.11	143.22
Dx,g cm-3	1.184	1.202
Z	32	32
Mu (mm-1)	0.926	0.000
F000	2309.0	0.0
F000'	2315.95	
h,k,lmax	32,32,31	
Nref	4878	
Tmin,Tmax		
Tmin'		

Correction method= Not given

Data completeness= 0.000 Theta(max)=

R(reflections)= wR2(reflections)=

S = Npar=

The following ALERTS were generated. Each ALERT has the format

test-name_ALERT_alert-type_alert-level.

Click on the hyperlinks for more details of the test.

Alert level A

PLAT770_ALERT_2_A Suspect C-H Bond in CIF: C_1 --H_2 . 1.48 Ang.

Author Response: The close contact alert is due to disorder of guest molecules.

PLAT770_ALERT_2_A Suspect C-H Bond in CIF: C_2 --H_2 . 1.35 Ang.

Author Response: The close contact alert is due to disorder of guest molecules.

PLAT770_ALERT_2_A Suspect C-H Bond in CIF: C_2 --H_6 . 1.36 Ang.

Author Response: The close contact alert is due to disorder of guest molecules.

PLAT770_ALERT_2_A Suspect C-H Bond in CIF: C_2 --H_1 . 1.38 Ang.

Author Response: The close contact alert is due to disorder of guest molecules.

PLAT770_ALERT_2_A Suspect C-H Bond in CIF: C_2 --H_1 . 1.62 Ang.

Author Response: The close contact alert is due to disorder of guest molecules.

PLAT770_ALERT_2_A Suspect C-H Bond in CIF: C_3 --H_1 . 1.40 Ang.

Author Response: The close contact alert is due to disorder of guest molecules.

PLAT770_ALERT_2_A Suspect C-H Bond in CIF: C_3 --H_6 . 1.49 Ang.

Author Response: The close contact alert is due to disorder of guest molecules.

PLAT770_ALERT_2_A Suspect C-H Bond in CIF: C_3 --H_1 . 1.61 Ang.

Author Response: The close contact alert is due to disorder of guest molecules.

PLAT770_ALERT_2_A Suspect C-H Bond in CIF: C_4 --H_1 . 1.52 Ang.

Author Response: The close contact alert is due to disorder of guest molecules.

PLAT770_ALERT_2_A Suspect C-H Bond in CIF: C_5 --H_6 . 1.48 Ang.

Author Response: The close contact alert is due to disorder of guest molecules.

PLAT770_ALERT_2_A Suspect C-H Bond in CIF: C_5 --H_2_1 . 1.56 Ang.

Author Response: The close contact alert is due to disorder of guest molecules.

PLAT770_ALERT_2_A Suspect C-H Bond in CIF: C_6 --H_1 . 1.43 Ang.

Author Response: The close contact alert is due to disorder of guest molecules.

PLAT770_ALERT_2_A Suspect C-H Bond in CIF: H_1 --C_2 . 1.38 Ang.

Author Response: The close contact alert is due to disorder of guest molecules.

PLAT770_ALERT_2_A Suspect C-H Bond in CIF: H_1 --C_3 . 1.40 Ang.

Author Response: The close contact alert is due to disorder of guest molecules.

PLAT770_ALERT_2_A Suspect C-H Bond in CIF: H_1 --C_6 . 1.43 Ang.

Author Response: The close contact alert is due to disorder of guest molecules.

PLAT770_ALERT_2_A Suspect C-H Bond in CIF: H_1 --C_4 . 1.52 Ang.

Author Response: The close contact alert is due to disorder of guest molecules.

PLAT770_ALERT_2_A Suspect C-H Bond in CIF: H_1 --C_3 . 1.61 Ang.

Author Response: The close contact alert is due to disorder of guest molecules.

PLAT770_ALERT_2_A Suspect C-H Bond in CIF: H_1 --C_2 . 1.62 Ang.

Author Response: The close contact alert is due to disorder of guest molecules.

PLAT770_ALERT_2_A Suspect C-H Bond in CIF: H_2 --C_2 . 1.35 Ang.

Author Response: The close contact alert is due to disorder of guest molecules.

PLAT770_ALERT_2_A Suspect C-H Bond in CIF: H_2 --C_1 . 1.48 Ang.

Author Response: The close contact alert is due to disorder of guest molecules.

PLAT770_ALERT_2_A Suspect C-H Bond in CIF: H_6 --C_2 . 1.36 Ang.

Author Response: The close contact alert is due to disorder of guest molecules.

PLAT770_ALERT_2_A Suspect C-H Bond in CIF: H_6 --C_5 . 1.48 Ang.

Author Response: The close contact alert is due to disorder of guest molecules.

PLAT770_ALERT_2_A Suspect C-H Bond in CIF: H_6 --C_3 . 1.49 Ang.

Author Response: The close contact alert is due to disorder of guest molecules.

PLAT770_ALERT_2_A Suspect C-H Bond in CIF: C_1_1 --H_3_3 . 1.42 Ang.

Author Response: The close contact alert is due to disorder of guest molecules.

PLAT770_ALERT_2_A Suspect C-H Bond in CIF: C_1_1 --H_2_1 . 1.60 Ang.

Author Response: The close contact alert is due to disorder of guest molecules.

PLAT770_ALERT_2_A Suspect C-H Bond in CIF: C_1_1 --H_1_1 . 1.62 Ang.

Author Response: The close contact alert is due to disorder of guest molecules.

PLAT770_ALERT_2_A Suspect C-H Bond in CIF: C_4_1 --H_3_2 . 1.39 Ang.

Author Response: The close contact alert is due to disorder of guest molecules.

PLAT770_ALERT_2_A Suspect C-H Bond in CIF: C_4_1 --H_1_2 . 1.49 Ang.

Author Response: The close contact alert is due to disorder of guest molecules.

PLAT770_ALERT_2_A Suspect C-H Bond in CIF: C_4_1 --H_3_2 . 1.55 Ang.

Author Response: The close contact alert is due to disorder of guest molecules.

PLAT770_ALERT_2_A Suspect C-H Bond in CIF: C_5_1 --H_4_3 . 1.60 Ang.

Author Response: The close contact alert is due to disorder of guest molecules.

PLAT770_ALERT_2_A Suspect C-H Bond in CIF: C_5_1 --H_2_2 . 1.61 Ang.

Author Response: The close contact alert is due to disorder of guest molecules.

PLAT770_ALERT_2_A Suspect C-H Bond in CIF: C_6_1 --H_6_3 . 1.41 Ang.

Author Response: The close contact alert is due to disorder of guest molecules.

PLAT770_ALERT_2_A Suspect C-H Bond in CIF: H_1_1 --C_1_1 . 1.62 Ang.

Author Response: The close contact alert is due to disorder of guest molecules.

PLAT770_ALERT_2_A Suspect C-H Bond in CIF: H_2_1 --C_5 . 1.56 Ang.

Author Response: The close contact alert is due to disorder of guest molecules.

PLAT770_ALERT_2_A Suspect C-H Bond in CIF: H_2_1 --C_1_1 . 1.60 Ang.

Author Response: The close contact alert is due to disorder of guest molecules.

PLAT770_ALERT_2_A Suspect C-H Bond in CIF: H_4_1 --C_3_2 . 1.34 Ang.

Author Response: The close contact alert is due to disorder of guest molecules.

PLAT770_ALERT_2_A Suspect C-H Bond in CIF: H_4_1 --C_3_2 . 1.48 Ang.

Author Response: The close contact alert is due to disorder of guest molecules.

PLAT770_ALERT_2_A Suspect C-H Bond in CIF: H_6_1 --C_6_3 . 1.39 Ang.

Author Response: The close contact alert is due to disorder of guest molecules.

PLAT770_ALERT_2_A Suspect C-H Bond in CIF: H_6_1 --C_5_3 . 1.63 Ang.

Author Response: The close contact alert is due to disorder of guest molecules.

```
PLAT770_ALERT_2_A Suspect C-H Bond in CIF:  C_1_2    --H_3_2     .       1.59 Ang.
```

Author Response: The close contact alert is due to disorder of guest molecules.

```
PLAT770_ALERT_2_A Suspect C-H Bond in CIF:  C_1_2    --H_3_2     .       1.60 Ang.
```

Author Response: The close contact alert is due to disorder of guest molecules.

```
PLAT770_ALERT_2_A Suspect C-H Bond in CIF:  C_1_2    --H_6_2     .       1.62 Ang.
```

Author Response: The close contact alert is due to disorder of guest molecules.

```
PLAT770_ALERT_2_A Suspect C-H Bond in CIF:  C_2_2    --H_1_2     .       1.40 Ang.
```

Author Response: The close contact alert is due to disorder of guest molecules.

```
PLAT770_ALERT_2_A Suspect C-H Bond in CIF:  C_2_2    --H_6_2     .       1.55 Ang.
```

Author Response: The close contact alert is due to disorder of guest molecules.

```
PLAT770_ALERT_2_A Suspect C-H Bond in CIF:  C_2_2    --H_6_2     .       1.60 Ang.
```

Author Response: The close contact alert is due to disorder of guest molecules.

```
PLAT770_ALERT_2_A Suspect C-H Bond in CIF:  C_3_2    --H_4_1     .       1.34 Ang.
```

Author Response: The close contact alert is due to disorder of guest molecules.

```
PLAT770_ALERT_2_A Suspect C-H Bond in CIF:  C_3_2    --H_3_2     .       1.48 Ang.
```

Author Response: The close contact alert is due to disorder of guest molecules.

```
PLAT770_ALERT_2_A Suspect C-H Bond in CIF:  C_3_2    --H_4_1     .       1.48 Ang.
```

Author Response: The close contact alert is due to disorder of guest molecules.

```
PLAT770_ALERT_2_A Suspect C-H Bond in CIF:  C_3_2    --H_1_2     .       1.62 Ang.
```

Author Response: The close contact alert is due to disorder of guest molecules.

PLAT770_ALERT_2_A Suspect C-H Bond in CIF: C_4_2 --H_4_2 . 1.36 Ang.

Author Response: The close contact alert is due to disorder of guest molecules.

PLAT770_ALERT_2_A Suspect C-H Bond in CIF: C_4_2 --H_5_2 . 1.42 Ang.

Author Response: The close contact alert is due to disorder of guest molecules.

PLAT770_ALERT_2_A Suspect C-H Bond in CIF: C_4_2 --H_5_2 . 1.51 Ang.

Author Response: The close contact alert is due to disorder of guest molecules.

PLAT770_ALERT_2_A Suspect C-H Bond in CIF: C_4_2 --H_5_2 . 1.59 Ang.

Author Response: The close contact alert is due to disorder of guest molecules.

PLAT770_ALERT_2_A Suspect C-H Bond in CIF: C_4_2 --H_6_2 . 1.62 Ang.

Author Response: The close contact alert is due to disorder of guest molecules.

PLAT770_ALERT_2_A Suspect C-H Bond in CIF: C_6_2 --H_4_2 . 1.59 Ang.

Author Response: The close contact alert is due to disorder of guest molecules.

PLAT770_ALERT_2_A Suspect C-H Bond in CIF: C_6_2 --H_5_2 . 1.62 Ang.

Author Response: The close contact alert is due to disorder of guest molecules.

PLAT770_ALERT_2_A Suspect C-H Bond in CIF: H_1_2 --C_2_2 . 1.40 Ang.

Author Response: The close contact alert is due to disorder of guest molecules.

PLAT770_ALERT_2_A Suspect C-H Bond in CIF: H_1_2 --C_4_1 . 1.49 Ang.

Author Response: The close contact alert is due to disorder of guest molecules.

PLAT770_ALERT_2_A Suspect C-H Bond in CIF: H_1_2 --C_3_2 . 1.62 Ang.

Author Response: The close contact alert is due to disorder of guest molecules.

PLAT770_ALERT_2_A Suspect C-H Bond in CIF: H_2_2 --C_5_1 . 1.61 Ang.

Author Response: The close contact alert is due to disorder of guest molecules.

PLAT770_ALERT_2_A Suspect C-H Bond in CIF: H_3_2 --C_4_1 . 1.39 Ang.

Author Response: The close contact alert is due to disorder of guest molecules.

PLAT770_ALERT_2_A Suspect C-H Bond in CIF: H_3_2 --C_3_2 . 1.48 Ang.

Author Response: The close contact alert is due to disorder of guest molecules.

PLAT770_ALERT_2_A Suspect C-H Bond in CIF: H_3_2 --C_4_1 . 1.55 Ang.

Author Response: The close contact alert is due to disorder of guest molecules.

PLAT770_ALERT_2_A Suspect C-H Bond in CIF: H_3_2 --C_1_2 . 1.59 Ang.

Author Response: The close contact alert is due to disorder of guest molecules.

PLAT770_ALERT_2_A Suspect C-H Bond in CIF: H_3_2 --C_1_2 . 1.60 Ang.

Author Response: The close contact alert is due to disorder of guest molecules.

PLAT770_ALERT_2_A Suspect C-H Bond in CIF: H_4_2 --C_4_2 . 1.36 Ang.

Author Response: The close contact alert is due to disorder of guest molecules.

PLAT770_ALERT_2_A Suspect C-H Bond in CIF: H_4_2 --C_6_2 . 1.59 Ang.

Author Response: The close contact alert is due to disorder of guest molecules.

PLAT770_ALERT_2_A Suspect C-H Bond in CIF: H_5_2 --C_4_2 . 1.42 Ang.

Author Response: The close contact alert is due to disorder of guest molecules.

PLAT770_ALERT_2_A Suspect C-H Bond in CIF: H_5_2 --C_4_2 . 1.51 Ang.

Author Response: The close contact alert is due to disorder of guest molecules.

PLAT770_ALERT_2_A Suspect C-H Bond in CIF: H_5_2 --C_4_2 . 1.59 Ang.

Author Response: The close contact alert is due to disorder of guest molecules.

PLAT770_ALERT_2_A Suspect C-H Bond in CIF: H_5_2 --C_6_2 . 1.62 Ang.

Author Response: The close contact alert is due to disorder of guest molecules.

PLAT770_ALERT_2_A Suspect C-H Bond in CIF: H_6_2 --C_2_2 . 1.55 Ang.

Author Response: The close contact alert is due to disorder of guest molecules.

PLAT770_ALERT_2_A Suspect C-H Bond in CIF: H_6_2 --C_2_2 . 1.60 Ang.

Author Response: The close contact alert is due to disorder of guest molecules.

PLAT770_ALERT_2_A Suspect C-H Bond in CIF: H_6_2 --C_4_2 . 1.62 Ang.

Author Response: The close contact alert is due to disorder of guest molecules.

PLAT770_ALERT_2_A Suspect C-H Bond in CIF: H_6_2 --C_1_2 . 1.62 Ang.

Author Response: The close contact alert is due to disorder of guest molecules.

PLAT770_ALERT_2_A Suspect C-H Bond in CIF: C_1_3 --H_1_3 . 1.44 Ang.

Author Response: The close contact alert is due to disorder of guest molecules.

PLAT770_ALERT_2_A Suspect C-H Bond in CIF: C_3_3 --H_3_3 . 1.51 Ang.

Author Response: The close contact alert is due to disorder of guest molecules.

PLAT770_ALERT_2_A Suspect C-H Bond in CIF: C_5_3 --H_6_1 . 1.63 Ang.

Author Response: The close contact alert is due to disorder of guest molecules.

PLAT770_ALERT_2_A Suspect C-H Bond in CIF: C_6_3 --H_6_1 . 1.39 Ang.

Author Response: The close contact alert is due to disorder of guest molecules.

PLAT770_ALERT_2_A Suspect C-H Bond in CIF: H_1_3 --C_1_3 . 1.44 Ang.

Author Response: The close contact alert is due to disorder of guest molecules.

PLAT770_ALERT_2_A Suspect C-H Bond in CIF: H_3_3 --C_1_1 . 1.42 Ang.

Author Response: The close contact alert is due to disorder of guest molecules.

PLAT770_ALERT_2_A Suspect C-H Bond in CIF: H_3_3 --C_3_3 . 1.51 Ang.

Author Response: The close contact alert is due to disorder of guest molecules.

PLAT770_ALERT_2_A Suspect C-H Bond in CIF: H_4_3 --C_5_1 . 1.60 Ang.

Author Response: The close contact alert is due to disorder of guest molecules.

PLAT770_ALERT_2_A Suspect C-H Bond in CIF: H_6_3 --C_6_1 . 1.41 Ang.

Author Response: The close contact alert is due to disorder of guest molecules.

Alert level B

PLAT043_ALERT_1_B Calculated and Reported Mol. Weight Differ by .. 2.11 Check

Author Response: This is likely due to the decimal points considers in the calculation. The reported strings are consistent with the atom sites information.

PLAT420_ALERT_2_B D-H Bond Without Acceptor O4 --H4O . Please Check

Author Response: The guest molecule benzene does not have a hydrogen bonding acceptor.

Alert level C

PLAT041_ALERT_1_C Calc. and Reported SumFormula Strings Differ Please Check

Author Response: This is likely due to the decimal points considers in the calculation. The reported strings are consistent with the atom sites information.

```
PLAT042_ALERT_1_C Calc. and Reported MoietyFormula Strings  Differ     Please Check
```

Author Response: This is likely due to the decimal points considers in the calculation. The reported strings are consistent with the atom sites information.

```
PLAT044_ALERT_1_C Calculated and Reported Density Dx  Differ by ..     0.0177 Check
```

Author Response: This is likely due to the decimal points considers in the calculation. The reported strings are consistent with the atom sites information.

```
PLAT077_ALERT_4_C Unitcell Contains Non-integer Number of Atoms ..     Please Check
```

Author Response: The structure contains disordered framework and guest molecules.

```
PLAT244_ALERT_4_C Low    'Solvent' Ueq as Compared to Neighbors of         O1 Check
```

Author Response: O1 is X-ray heavier compared to neightors and the thermal vibration is less intensive.

```
PLAT244_ALERT_4_C Low    'Solvent' Ueq as Compared to Neighbors of         O3 Check
```

Author Response: O1 is X-ray heavier compared to neightors and the thermal vibration is less intensive.

```
PLAT341_ALERT_3_C Low Bond Precision on  C-C Bonds ..............    0.00687 Ang.
```

Author Response: Structure are obtained from powder diffraction refinement. Low precision could be due to insufficient resolution.

Alert level G

```
CELLZ01_ALERT_1_G Difference between formula and atom_site contents detected.
CELLZ01_ALERT_1_G ALERT: check formula stoichiometry or atom site occupancies.
           From the CIF: _cell_formula_units_Z   32
           From the CIF: _chemical_formula_sum  C6.48 H5.26 Ni0.05 O2.22 Ti0.45
           TEST: Compare cell contents of formula and atom_site data

           atom    Z*formula  cif sites diff
           C        207.36    207.36    0.00
           H        168.32    168.23    0.09
           Ni         1.60      1.74   -0.14
           O         71.04     71.13   -0.09
           Ti        14.40     14.26    0.14
PLAT004_ALERT_5_G Polymeric Structure Found with Maximum Dimension          3 Info
```

```
PLAT092_ALERT_4_G Check: Wavelength Given is not Cu,Ga,Mo,Ag,In Ka    0.82439 Ang.
PLAT143_ALERT_4_G s.u. on c - Axis Small or Missing .............    0.00006 Ang.
PLAT164_ALERT_4_G Nr. of Refined C-H H-Atoms in Heavy-Atom Struct.          2 Note
PLAT301_ALERT_3_G Main Residue  Disorder .............(Resd  1  )        31% Note
PLAT301_ALERT_3_G Main Residue  Disorder .............(Resd  2  )       100% Note
PLAT301_ALERT_3_G Main Residue  Disorder .............(Resd  3  )       100% Note
PLAT301_ALERT_3_G Main Residue  Disorder .............(Resd  4  )       100% Note
PLAT301_ALERT_3_G Main Residue  Disorder .............(Resd  5  )       100% Note
PLAT302_ALERT_4_G Anion/Solvent/Minor-Residue Disorder (Resd  6  )       100% Note
PLAT311_ALERT_2_G Isolated Disordered Oxygen Atom (No H's ?) .....         O5 Check
PLAT411_ALERT_2_G Short Inter H...H Contact  H4       ..H_1_1    .       2.11 Ang.
                                        -1/2+x,1/2-y,1/2-z  =     29_455 Check
PLAT411_ALERT_2_G Short Inter H...H Contact  H4       ..H_1_1    .       2.11 Ang.
                                        -1/2+x,1/2-y,1/2+z  =     30_455 Check
PLAT411_ALERT_2_G Short Inter H...H Contact  H5       ..H_3_1    .       1.87 Ang.
                                                  -1+x,y,z  =      1_455 Check
PLAT411_ALERT_2_G Short Inter H...H Contact  H5       ..H_3      .       1.94 Ang.
                                      -1/2+y,-1/2+x,-1/2+z  =     28_444 Check
PLAT413_ALERT_2_G Short Inter XH3 .. XHn     H4       ..H_2_3    .       2.02 Ang.
                                               1-y,1-x,1-z  =      5_666 Check
PLAT413_ALERT_2_G Short Inter XH3 .. XHn     H4       ..H_2_3    .       2.02 Ang.
                                                 1-y,1-x,z  =      6_665 Check
PLAT413_ALERT_2_G Short Inter XH3 .. XHn     H4       ..H_5      .       1.79 Ang.
                                        -1/2+x,1/2-y,3/2-z  =     29_456 Check
PLAT413_ALERT_2_G Short Inter XH3 .. XHn     H4       ..H_5      .       1.79 Ang.
                                       -1/2+x,1/2-y,-1/2+z  =     30_454 Check
PLAT432_ALERT_2_G Short Inter X...Y Contact  C1       ..C_4      .       2.92 Ang.
                                      -1/2+x,-1/2+y,-1/2+z  =     17_444 Check
PLAT432_ALERT_2_G Short Inter X...Y Contact  C1       ..C_4      .       2.92 Ang.
                                       -1/2+x,1/2-y,-1/2+z  =     30_454 Check
PLAT432_ALERT_2_G Short Inter X...Y Contact  C1       ..C_4      .       2.92 Ang.
                                        -1/2+x,1/2-y,3/2-z  =     29_456 Check
PLAT432_ALERT_2_G Short Inter X...Y Contact  C1       ..C_4      .       2.92 Ang.
                                       -1/2+x,-1/2+y,3/2-z  =     31_446 Check
PLAT432_ALERT_2_G Short Inter X...Y Contact  C2       ..C_4      .       2.94 Ang.
                                      -1/2+x,-1/2+y,-1/2+z  =     17_444 Check
PLAT432_ALERT_2_G Short Inter X...Y Contact  C2       ..C_4      .       2.94 Ang.
                                       -1/2+x,1/2-y,-1/2+z  =     30_454 Check
PLAT432_ALERT_2_G Short Inter X...Y Contact  C2       ..C_4      .       2.94 Ang.
                                        -1/2+x,1/2-y,3/2-z  =     29_456 Check
PLAT432_ALERT_2_G Short Inter X...Y Contact  C2       ..C_4      .       2.94 Ang.
                                       -1/2+x,-1/2+y,3/2-z  =     31_446 Check
PLAT432_ALERT_2_G Short Inter X...Y Contact  C2       ..C_5      .       3.04 Ang.
                                        -1/2+x,1/2-y,3/2-z  =     29_456 Check
PLAT432_ALERT_2_G Short Inter X...Y Contact  C2       ..C_5      .       3.04 Ang.
                                       -1/2+x,-1/2+y,3/2-z  =     31_446 Check
PLAT432_ALERT_2_G Short Inter X...Y Contact  C2       ..C_5      .       3.04 Ang.
                                       -1/2+x,1/2-y,-1/2+z  =     30_454 Check
PLAT432_ALERT_2_G Short Inter X...Y Contact  C2       ..C_5      .       3.04 Ang.
                                      -1/2+x,-1/2+y,-1/2+z  =     17_444 Check
PLAT432_ALERT_2_G Short Inter X...Y Contact  C3       ..C_5      .       2.75 Ang.
                                        -1/2+x,1/2-y,3/2-z  =     29_456 Check
PLAT432_ALERT_2_G Short Inter X...Y Contact  C3       ..C_5      .       2.75 Ang.
                                       -1/2+x,1/2-y,-1/2+z  =     30_454 Check
PLAT432_ALERT_2_G Short Inter X...Y Contact  C_5_1    ..C_5_1    .       2.95 Ang.
                                                 1-y,1-x,z  =      6_665 Check
PLAT720_ALERT_4_G Number of Unusual/Non-Standard Labels .........         48 Note
```

```
PLAT764_ALERT_4_G Overcomplete CIF Bond List Detected (Rep/Expd) .       2.36 Ratio
PLAT773_ALERT_2_G Check long C-C Bond in CIF: C_1      --C_1             1.72 Ang.
PLAT773_ALERT_2_G Check long C-C Bond in CIF: C_1      --C_2             1.72 Ang.
PLAT773_ALERT_2_G Check long C-C Bond in CIF: C_1      --C_5             1.86 Ang.
PLAT773_ALERT_2_G Check long C-C Bond in CIF: C_1      --C_2             1.89 Ang.
PLAT773_ALERT_2_G Check long C-C Bond in CIF: C_1      --C_4             1.92 Ang.
PLAT773_ALERT_2_G Check long C-C Bond in CIF: C_1      --C_6             2.02 Ang.
PLAT773_ALERT_2_G Check long C-C Bond in CIF: C_1      --C_4             2.04 Ang.
PLAT773_ALERT_2_G Check long C-C Bond in CIF: C_2      --C_1             1.72 Ang.
PLAT773_ALERT_2_G Check long C-C Bond in CIF: C_2      --C_2             1.83 Ang.
PLAT773_ALERT_2_G Check long C-C Bond in CIF: C_2      --C_1             1.89 Ang.
PLAT773_ALERT_2_G Check long C-C Bond in CIF: C_2      --C_5             1.90 Ang.
PLAT773_ALERT_2_G Check long C-C Bond in CIF: C_3      --C_5             1.94 Ang.
PLAT773_ALERT_2_G Check long C-C Bond in CIF: C_4      --C_1             1.92 Ang.
PLAT773_ALERT_2_G Check long C-C Bond in CIF: C_4      --C_1             2.04 Ang.
PLAT773_ALERT_2_G Check long C-C Bond in CIF: C_4      --C_5             2.04 Ang.
PLAT773_ALERT_2_G Check long C-C Bond in CIF: C_5      --C_6             1.71 Ang.
PLAT773_ALERT_2_G Check long C-C Bond in CIF: C_5      --C_1             1.86 Ang.
PLAT773_ALERT_2_G Check long C-C Bond in CIF: C_5      --C_2             1.90 Ang.
PLAT773_ALERT_2_G Check long C-C Bond in CIF: C_5      --C_3             1.94 Ang.
PLAT773_ALERT_2_G Check long C-C Bond in CIF: C_5      --C_4             2.04 Ang.
PLAT773_ALERT_2_G Check long C-C Bond in CIF: C_6      --C_5             1.71 Ang.
PLAT773_ALERT_2_G Check long C-C Bond in CIF: C_6      --C_1             2.02 Ang.
PLAT773_ALERT_2_G Check long C-C Bond in CIF: C_1_1    --C_2_3           2.03 Ang.
PLAT773_ALERT_2_G Check long C-C Bond in CIF: C_2_1    --C_6_1           1.96 Ang.
PLAT773_ALERT_2_G Check long C-C Bond in CIF: C_3_1    --C_5_1           1.84 Ang.
PLAT773_ALERT_2_G Check long C-C Bond in CIF: C_4_1    --C_5_1           1.82 Ang.
PLAT773_ALERT_2_G Check long C-C Bond in CIF: C_5_1    --C_5_3           1.74 Ang.
PLAT773_ALERT_2_G Check long C-C Bond in CIF: C_5_1    --C_4_1           1.82 Ang.
PLAT773_ALERT_2_G Check long C-C Bond in CIF: C_5_1    --C_3_1           1.84 Ang.
PLAT773_ALERT_2_G Check long C-C Bond in CIF: C_5_1    --C_4_3           2.02 Ang.
PLAT773_ALERT_2_G Check long C-C Bond in CIF: C_5_1    --C_4_3           2.06 Ang.
PLAT773_ALERT_2_G Check long C-C Bond in CIF: C_6_1    --C_5_3           1.71 Ang.
PLAT773_ALERT_2_G Check long C-C Bond in CIF: C_6_1    --C_1_3           1.72 Ang.
PLAT773_ALERT_2_G Check long C-C Bond in CIF: C_6_1    --C_4_3           1.82 Ang.
PLAT773_ALERT_2_G Check long C-C Bond in CIF: C_6_1    --C_2_3           1.84 Ang.
PLAT773_ALERT_2_G Check long C-C Bond in CIF: C_6_1    --C_4_3           1.87 Ang.
PLAT773_ALERT_2_G Check long C-C Bond in CIF: C_6_1    --C_3_3           1.88 Ang.
PLAT773_ALERT_2_G Check long C-C Bond in CIF: C_6_1    --C_2_1           1.96 Ang.
PLAT773_ALERT_2_G Check long C-C Bond in CIF: C_1_2    --C_6_2           1.71 Ang.
PLAT773_ALERT_2_G Check long C-C Bond in CIF: C_1_2    --C_6_2           1.73 Ang.
PLAT773_ALERT_2_G Check long C-C Bond in CIF: C_3_2    --C_6_2           1.76 Ang.
PLAT773_ALERT_2_G Check long C-C Bond in CIF: C_4_2    --C_4_2           1.84 Ang.
PLAT773_ALERT_2_G Check long C-C Bond in CIF: C_6_2    --C_1_2           1.71 Ang.
PLAT773_ALERT_2_G Check long C-C Bond in CIF: C_6_2    --C_1_2           1.73 Ang.
PLAT773_ALERT_2_G Check long C-C Bond in CIF: C_6_2    --C_3_2           1.76 Ang.
PLAT773_ALERT_2_G Check long C-C Bond in CIF: C_6_2    --C_6_2           1.85 Ang.
PLAT773_ALERT_2_G Check long C-C Bond in CIF: C_1_3    --C_6_1           1.72 Ang.
PLAT773_ALERT_2_G Check long C-C Bond in CIF: C_1_3    --C_6_3           1.93 Ang.
PLAT773_ALERT_2_G Check long C-C Bond in CIF: C_1_3    --C_2_3           1.94 Ang.
PLAT773_ALERT_2_G Check long C-C Bond in CIF: C_1_3    --C_3_3           2.02 Ang.
PLAT773_ALERT_2_G Check long C-C Bond in CIF: C_2_3    --C_2_3           1.71 Ang.
PLAT773_ALERT_2_G Check long C-C Bond in CIF: C_2_3    --C_6_1           1.84 Ang.
PLAT773_ALERT_2_G Check long C-C Bond in CIF: C_2_3    --C_1_3           1.94 Ang.
PLAT773_ALERT_2_G Check long C-C Bond in CIF: C_2_3    --C_3_3           2.02 Ang.
PLAT773_ALERT_2_G Check long C-C Bond in CIF: C_2_3    --C_1_1           2.03 Ang.
PLAT773_ALERT_2_G Check long C-C Bond in CIF: C_3_3    --C_6_1           1.88 Ang.
```

```
PLAT773_ALERT_2_G Check long C-C Bond in CIF: C_3_3    --C_1_3          2.02 Ang.
PLAT773_ALERT_2_G Check long C-C Bond in CIF: C_3_3    --C_2_3          2.02 Ang.
PLAT773_ALERT_2_G Check long C-C Bond in CIF: C_4_3    --C_6_1          1.82 Ang.
PLAT773_ALERT_2_G Check long C-C Bond in CIF: C_4_3    --C_6_1          1.87 Ang.
PLAT773_ALERT_2_G Check long C-C Bond in CIF: C_4_3    --C_5_1          2.02 Ang.
PLAT773_ALERT_2_G Check long C-C Bond in CIF: C_4_3    --C_5_1          2.06 Ang.
PLAT773_ALERT_2_G Check long C-C Bond in CIF: C_5_3    --C_6_1          1.71 Ang.
PLAT773_ALERT_2_G Check long C-C Bond in CIF: C_5_3    --C_5_1          1.74 Ang.
PLAT773_ALERT_2_G Check long C-C Bond in CIF: C_6_3    --C_1_3          1.93 Ang.
PLAT780_ALERT_1_G Coordinates do not Form a Properly Connected Set     Please Do !
PLAT811_ALERT_5_G No ADDSYM Analysis: Too Many Excluded Atoms ....          ! Info
```

```
  84 ALERT level A = Most likely a serious problem - resolve or explain
   2 ALERT level B = A potentially serious problem, consider carefully
   7 ALERT level C = Check. Ensure it is not caused by an omission or oversight
 105 ALERT level G = General information/check it is not something unexpected

   7 ALERT type 1 CIF construction/syntax error, inconsistent or missing data
 174 ALERT type 2 Indicator that the structure model may be wrong or deficient
   6 ALERT type 3 Indicator that the structure quality may be low
   9 ALERT type 4 Improvement, methodology, query or suggestion
   2 ALERT type 5 Informative message, check
```

It is advisable to attempt to resolve as many as possible of the alerts in all categories. Often the minor alerts point to easily fixed oversights, errors and omissions in your CIF or refinement strategy, so attention to these fine details can be worthwhile. In order to resolve some of the more serious problems it may be necessary to carry out additional measurements or structure refinements. However, the purpose of your study may justify the reported deviations and the more serious of these should normally be commented upon in the discussion or experimental section of a paper or in the "special_details" fields of the CIF. checkCIF was carefully designed to identify outliers and unusual parameters, but every test has its limitations and alerts that are not important in a particular case may appear. Conversely, the absence of alerts does not guarantee there are no aspects of the results needing attention. It is up to the individual to critically assess their own results and, if necessary, seek expert advice.

Publication of your CIF in IUCr journals

A basic structural check has been run on your CIF. These basic checks will be run on all CIFs submitted for publication in IUCr journals (*Acta Crystallographica*, *Journal of Applied Crystallography*, *Journal of Synchrotron Radiation*); however, if you intend to submit to *Acta Crystallographica Section C* or *E* or *IUCrData*, you should make sure that full publication checks are run on the final version of your CIF prior to submission.

Publication of your CIF in other journals

Please refer to the *Notes for Authors* of the relevant journal for any special instructions relating to CIF submission.

PLATON version of 06/07/2023; check.def file version of 30/06/2023

Datablock C6H6@MIL-125-Ni - ellipsoid plot

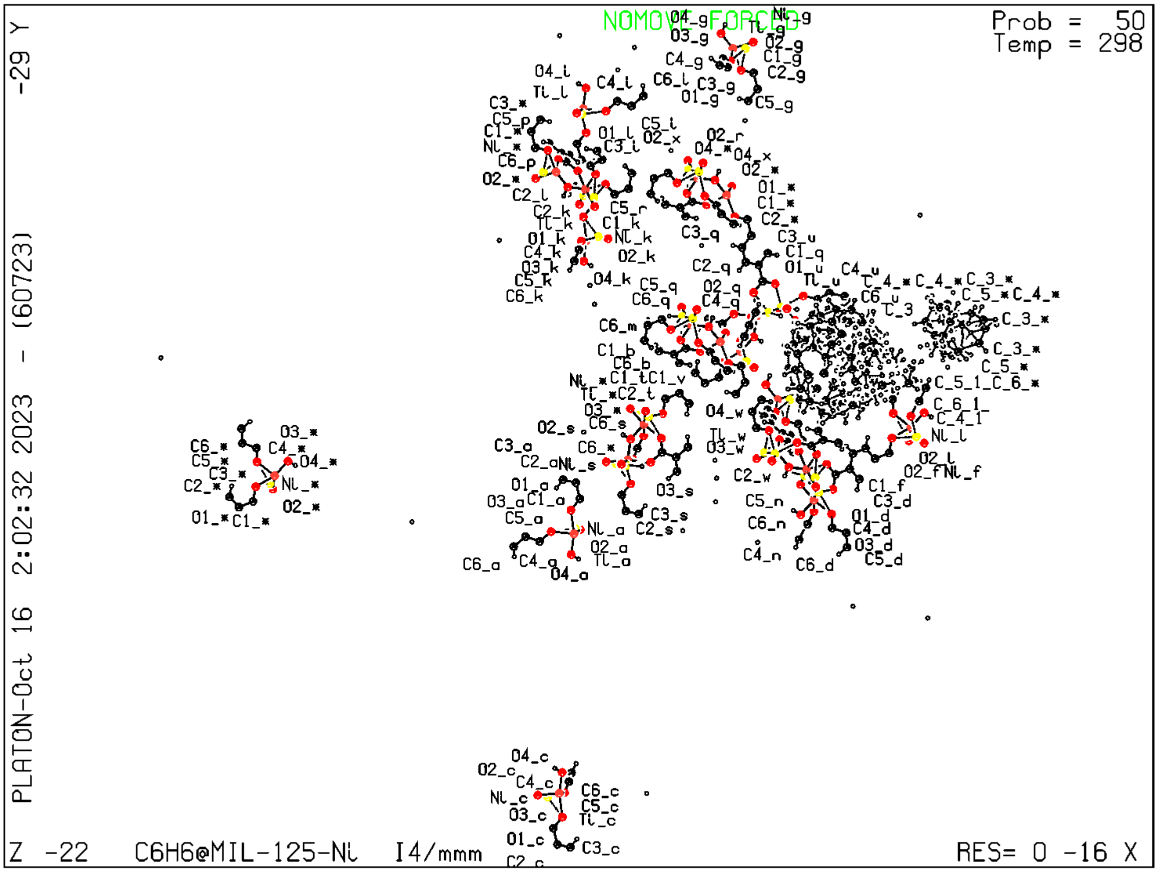

Supplement: Supplementary file 2 — Crystallographic data (11 CIFs) and checkCIF reports. [file 41563_2024_2029_MOESM2_ESM.zip › cifs and check cif reports/C6H6@MIL-125-Ni_checkcif.pdf]
