# Supplementary material for: Trace benzene capture by decoration of structural defects in metal–organic framework materials
Source: Nat Mater. 2024 Oct 29;23(11):1531–8. doi: 10.1038/s41563-024-02029-1 (PMC11525167; doi:10.1038/s41563-024-02029-1)

checkCIF/PLATON report

You have not supplied any structure factors. As a result the full set of tests cannot be run.

THIS REPORT IS FOR GUIDANCE ONLY. IF USED AS PART OF A REVIEW PROCEDURE FOR PUBLICATION, IT SHOULD NOT REPLACE THE EXPERTISE OF AN EXPERIENCED CRYSTALLOGRAPHIC REFEREE.

No syntax errors found. CIF dictionary Interpreting this report

Datablock: MIL-125-defect

Bond precision:	C-C = 0.0065 A	Wavelength=0

Cell:	a=18.61995(14)	b=18.61995(14)	c=18.1534(2)
	alpha=90	beta=90	gamma=90
Temperature:	10 K		

	Calculated	Reported
Volume	6293.83(12)	6293.82(13)
Space group	I 4/m m m	I4/mmm
Hall group	-I 4 2	-I 4 2
Moiety formula	C48 H28.53 O34.73 Ti7.15, 0.382(C8 H32 O8), 0.318(H)	C48 H28.53 O34.73 Ti7.15, 0.382(C8 H32 O8), 0.318(H)
Sum formula	C51.06 H41.08 O37.78 Ti7.15	C51.06 H41.08 O37.78 Ti7.15
Mr	1601.50	1601.49
Dx,g cm-3	0.845	0.845
Z	2	2
Mu (mm-1)	0.000	0.000
F000	761.9	0.0
F000'	1613.89	
h,k,lmax		
Nref		
Tmin,Tmax		
Tmin'		

Correction method= Not given

Data completeness= Theta(max)=

R(reflections)= wR2(reflections)=
S = Npar=

The following ALERTS were generated. Each ALERT has the format
test-name_ALERT_alert-type_alert-level.
Click on the hyperlinks for more details of the test.

Alert level A

```
PLAT602_ALERT_2_A Solvent Accessible VOID(S) in Structure .......          ! Check
```

Author Response: The powder sample is partially desolvated with residual methanol in the pore.

Alert level B

```
PLAT420_ALERT_2_B D-H Bond Without Acceptor  O4       --H4O      .     Please Check
```

Author Response: No hydrogen bonding is formed between the guest and the framework.

Alert level C

```
CELLK01_ALERT_1_C  Check that the cell measurement temperature is in Kelvin.
            Value of measurement temperature given =    10.000
```

Author Response: The structure is obtained from neutron powder diffraction using cold neutron source at 10K from ISIS WISH beamline.

```
PLAT077_ALERT_4_C Unitcell Contains Non-integer Number of Atoms ..     Please Check
```

Author Response: The structure contains disorder.

```
PLAT341_ALERT_3_C Low Bond Precision on  C-C Bonds ..............     0.0065 Ang.
```

Author Response: Structure is obtained from powder diffraction refinement. The low precision is due to limited resoltion.

Alert level G

```
PLAT049_ALERT_1_G Calculated Density Less Than 1.0 gcm-3 .........     0.8451 Check
PLAT300_ALERT_4_G Atom Site Occupancy of C3          Constrained at       0.5 Check
PLAT300_ALERT_4_G Atom Site Occupancy of C6          Constrained at       0.5 Check
PLAT300_ALERT_4_G Atom Site Occupancy of C66         Constrained at       0.5 Check
PLAT300_ALERT_4_G Atom Site Occupancy of H4          Constrained at       0.5 Check
PLAT300_ALERT_4_G Atom Site Occupancy of H5          Constrained at       0.5 Check
PLAT300_ALERT_4_G Atom Site Occupancy of H55         Constrained at       0.5 Check
PLAT301_ALERT_3_G Main Residue  Disorder .............(Resd  1  )       58% Note
PLAT302_ALERT_4_G Anion/Solvent/Minor-Residue Disorder (Resd  2  )      100% Note
```

```
PLAT304_ALERT_4_G Non-Integer Number of Atoms in ..... (Resd  1  )     118.41 Check
PLAT304_ALERT_4_G Non-Integer Number of Atoms in ..... (Resd  2  )       9.17 Check
PLAT304_ALERT_4_G Non-Integer Number of Atoms in ..... (Resd  3  )       0.02 Check
PLAT432_ALERT_2_G Short Inter X...Y Contact  C2       ..C3       .       2.40 Ang.
                                                 1-x,y,z  =      4_655 Check
PLAT432_ALERT_2_G Short Inter X...Y Contact  C2       ..C3       .       2.40 Ang.
                                               1-x,y,1-z  =      3_656 Check
PLAT432_ALERT_2_G Short Inter X...Y Contact  C2       ..C3       .       2.40 Ang.
                                                1-x,-y,z  =      2_655 Check
PLAT432_ALERT_2_G Short Inter X...Y Contact  C2       ..C3       .       2.40 Ang.
                                              1-x,-y,1-z  =     16_656 Check
PLAT432_ALERT_2_G Short Inter X...Y Contact  C2       ..C2       .       2.77 Ang.
                                                1-x,-y,z  =      2_655 Check
PLAT432_ALERT_2_G Short Inter X...Y Contact  C3       ..C3       .       1.39 Ang.
                                                 1-x,y,z  =      4_655 Check
PLAT432_ALERT_2_G Short Inter X...Y Contact  C3       ..C3       .       1.41 Ang.
                                               1-x,y,1-z  =      3_656 Check
PLAT432_ALERT_2_G Short Inter X...Y Contact  C3       ..C3       .       2.76 Ang.
                                                1-x,-y,z  =      2_655 Check
PLAT432_ALERT_2_G Short Inter X...Y Contact  C3       ..C3       .       2.77 Ang.
                                              1-x,-y,1-z  =     16_656 Check
PLAT432_ALERT_2_G Short Inter X...Y Contact  C5       ..C66      .       2.37 Ang.
                                      1/2-x,1/2-y,1/2-z  =     32_555 Check
PLAT432_ALERT_2_G Short Inter X...Y Contact  C5       ..C66      .       2.37 Ang.
                                      1/2-y,1/2-x,1/2-z  =     21_555 Check
PLAT432_ALERT_2_G Short Inter X...Y Contact  C5       ..C6       .       2.37 Ang.
                                      1/2-y,1/2-x,1/2-z  =     21_555 Check
PLAT432_ALERT_2_G Short Inter X...Y Contact  C5       ..C6       .       2.37 Ang.
                                      1/2-x,1/2-y,1/2-z  =     32_555 Check
PLAT432_ALERT_2_G Short Inter X...Y Contact  C5       ..C5       .       2.74 Ang.
                                      1/2-y,1/2-x,1/2-z  =     21_555 Check
PLAT432_ALERT_2_G Short Inter X...Y Contact  C6       ..C66      .       1.35 Ang.
                                      1/2-x,1/2-y,1/2-z  =     32_555 Check
PLAT432_ALERT_2_G Short Inter X...Y Contact  C6       ..C6       .       1.40 Ang.
                                      1/2-y,1/2-x,1/2-z  =     21_555 Check
PLAT432_ALERT_2_G Short Inter X...Y Contact  C6       ..C66      .       2.73 Ang.
                                      1/2-y,1/2-x,1/2-z  =     21_555 Check
PLAT432_ALERT_2_G Short Inter X...Y Contact  C6       ..C6       .       2.76 Ang.
                                      1/2-x,1/2-y,1/2-z  =     32_555 Check
PLAT432_ALERT_2_G Short Inter X...Y Contact  C66      ..C66      .       1.39 Ang.
                                      1/2-y,1/2-x,1/2-z  =     21_555 Check
PLAT432_ALERT_2_G Short Inter X...Y Contact  C66      ..C66      .       2.75 Ang.
                                      1/2-x,1/2-y,1/2-z  =     32_555 Check
PLAT720_ALERT_4_G Number of Unusual/Non-Standard Labels .........          6 Note
PLAT764_ALERT_4_G Overcomplete CIF Bond List Detected (Rep/Expd) .       1.46 Ratio
PLAT811_ALERT_5_G No ADDSYM Analysis: Too Many Excluded Atoms ....          ! Info
```

---

1 **ALERT level A** = Most likely a serious problem - resolve or explain
1 **ALERT level B** = A potentially serious problem, consider carefully
3 **ALERT level C** = Check. Ensure it is not caused by an omission or oversight
35 **ALERT level G** = General information/check it is not something unexpected

2 ALERT type 1 CIF construction/syntax error, inconsistent or missing data

```
22 ALERT type 2 Indicator that the structure model may be wrong or deficient
 2 ALERT type 3 Indicator that the structure quality may be low
13 ALERT type 4 Improvement, methodology, query or suggestion
 1 ALERT type 5 Informative message, check
```

It is advisable to attempt to resolve as many as possible of the alerts in all categories. Often the minor alerts point to easily fixed oversights, errors and omissions in your CIF or refinement strategy, so attention to these fine details can be worthwhile. In order to resolve some of the more serious problems it may be necessary to carry out additional measurements or structure refinements. However, the purpose of your study may justify the reported deviations and the more serious of these should normally be commented upon in the discussion or experimental section of a paper or in the "special_details" fields of the CIF. checkCIF was carefully designed to identify outliers and unusual parameters, but every test has its limitations and alerts that are not important in a particular case may appear. Conversely, the absence of alerts does not guarantee there are no aspects of the results needing attention. It is up to the individual to critically assess their own results and, if necessary, seek expert advice.

Publication of your CIF in IUCr journals

A basic structural check has been run on your CIF. These basic checks will be run on all CIFs submitted for publication in IUCr journals (*Acta Crystallographica*, *Journal of Applied Crystallography*, *Journal of Synchrotron Radiation*); however, if you intend to submit to *Acta Crystallographica Section C* or *E* or *IUCrData*, you should make sure that full publication checks are run on the final version of your CIF prior to submission.

Publication of your CIF in other journals

Please refer to the *Notes for Authors* of the relevant journal for any special instructions relating to CIF submission.

PLATON version of 06/07/2023; check.def file version of 30/06/2023

Datablock MIL-125-defect - ellipsoid plot

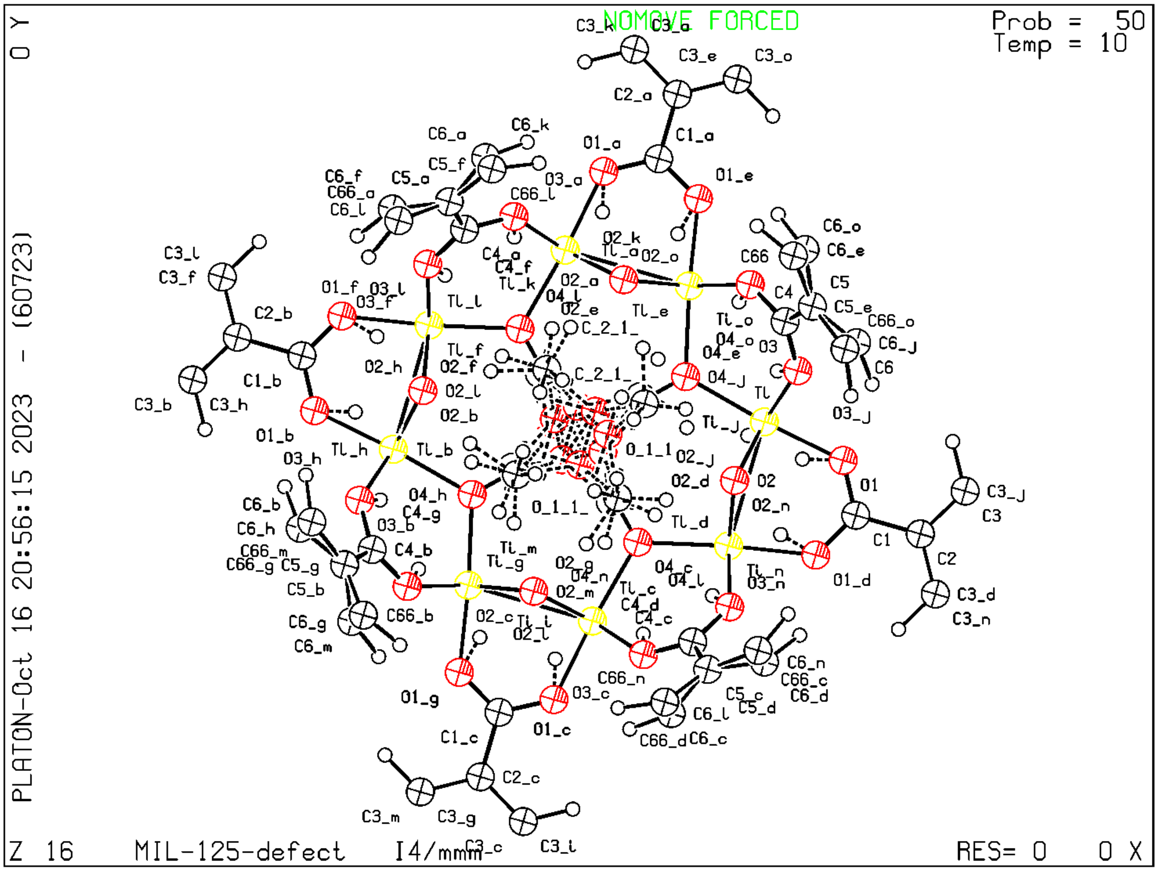

Supplement: Supplementary file 2 — Crystallographic data (11 CIFs) and checkCIF reports. [file 41563_2024_2029_MOESM2_ESM.zip › cifs and check cif reports/MIL-125-defect_checkcif.pdf]
